# A Redox-Active Hydrogen Bond Acceptor Enables Ligand Exchange in a Zinc Complex

**DOI:** 10.1021/acs.inorgchem.5c04839

**Published:** 2025-11-17

**Authors:** Christin N. Gilchrist, Matthias Zeller, John J. Kiernicki

**Affiliations:** † Department of Chemistry, 7470 Drury University, Springfield, Missouri 65802, United States; ‡ H. C. Brown Laboratory, James Tarpo Jr. and Margaret Tarpo Department of Chemistry, 8522 Purdue University, West Lafayette, Indiana 47907, United States

## Abstract

A zinc complex featuring a redox-active hydrogen bond acceptor, ferrocenecarboxylate, was designed to probe how the hydrogen bond strength could influence ligand binding at the metal. Oxidation of the redox-active hydrogen bond acceptor enabled facile ligand substitution with trifluoromethanesulfonate. Control studies confirmed that no substitution was possible without oxidation and that hydrogen bonding interactions were a necessary component for substitution to occur. Importantly, redox-tuning of the hydrogen bond acceptor can be achieved at a mild redox potential.

## Graphical abstract

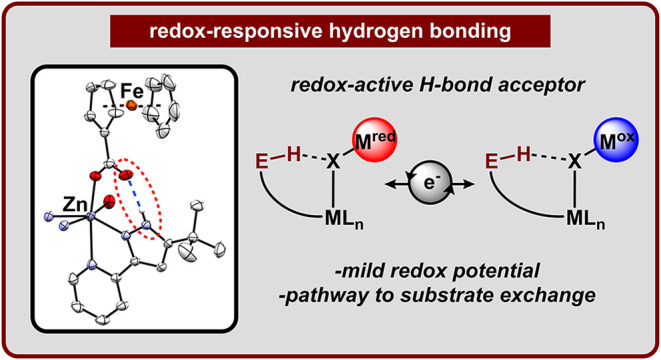

## Introduction

Noncovalent interactions are established to be a key component for substrate capture, orientation, and activation in enzymatic processes.^1^ Within this realm, hydrogen bonds (H-bonds) often garner the most attention and synthetic chemists have routinely implemented them into biomimetic systems to probe key intermediates in challenging bond activation reactions.^2–4^ Despite their appreciated importance, methods to fine-tune H-bond strength remain limited. In biology, H-bonding interactions adjacent to the active site of an enzyme are capable of being modulated through a number of stimuli, including allosteric interactions, further H-bond donors/acceptors, and/or redox-state changes of electronically coupled components.^5–8^ In contrast, synthetic systems where H-bond modulation has been performed generally rely on the Hammett-type redesign of one of the two components of the H-bond.^9–14^ Such an approach often requires new strategies, a new synthetic protocol, and/or new starting materials. A better approach may involve in situ - or late-stage - tuning of a given H-bond within a single molecule. When implemented into coordination complexes, this approach can provide a single platform that contains modular H-bonding to bias equilibrium processes (Figure 1).

**Figure 1:**
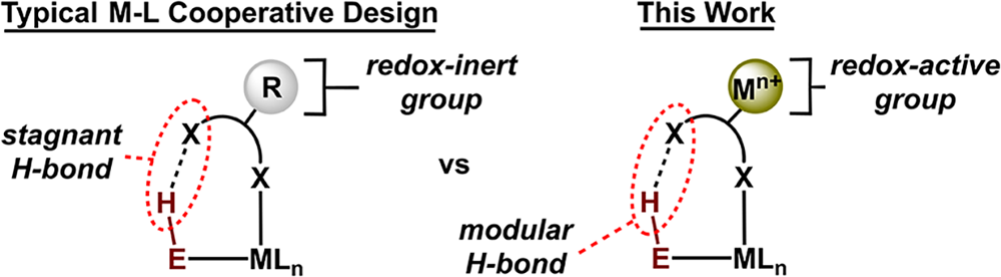
Design of a metal–ligand cooperative system featuring a redox-active H-bond acceptor to promote H-bond tuning.

H-bonds are electrostatic, and thus, an alteration of the partial charge of either the acidic or basic component will influence the bond strength. Therefore, the H-bonding strength can be increased either by increasing the positive charge of the H-bond donor or by increasing the negative charge of the H-bond acceptor. Fundamental studies have been performed in which redox-active moieties have been employed as a method to tune the partial charge of the donor/acceptor components. Rotello and co-workers designed flavin mimics featuring intermolecular H-bonding interactions and found that reduction of the flavin surrogate increased H-bond strength by ∼2–4 kcal/mol.^15,16^ The aforementioned studies, however, relied on redox-active components containing very negative reduction potentials, limiting their compatibility. Redox-active H-bonds, to be broadly applicable, must operate at a mild potential. By attaching ferrocene to an amido-pyridine sensor, Tucker and co-workers were able to demonstrate improved binding affinity for carboxylic acids upon oxidation.^17^ Through utilization of the Fc/Fc^+^ redox couple, assembly properties were able to be tuned at a modest potential (+0.24 V vs Fc/Fc^+^). Mild-potential on/off switches for acidity control have been further extended to boron Lewis acids to enhance the binding affinity of basic small molecules.^18,19^

Key design strategies in metal–ligand scaffolds often incorporate H-bond donor/acceptor sites adjacent to a metal center to modulate activity in ways that primary coordination sphere modification cannot.^20,21^ We hypothesized that redox-active H-bonds could provide an additional control element to such complexes for the purpose of facilitating substrate binding equilibria and promoting ligand exchange reactions via in situ tuning (Figure 2). While sensors based on redox activity are well-established,^22–27^ metal–ligand frameworks featuring redox-active H-bonds represent an underexplored mechanism to change the thermodynamic landscape of acidic/basic interactions.

**Figure 2:**
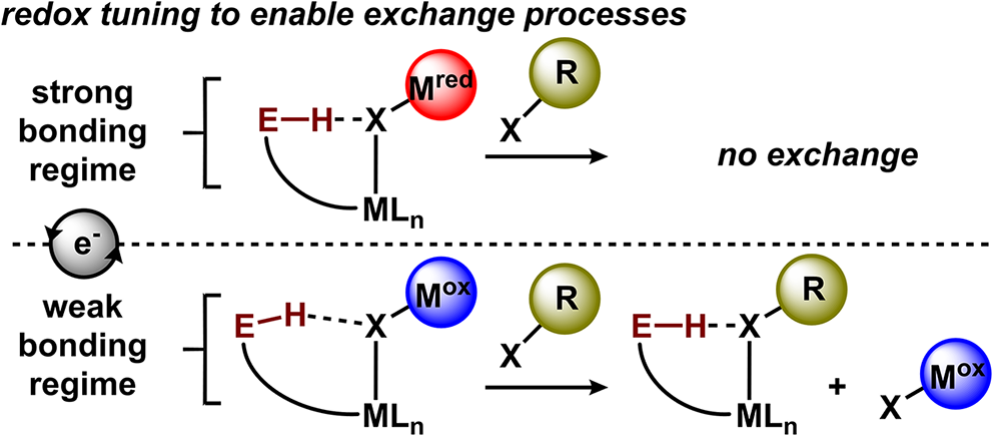
Tuning H-bond strength can promote substrate exchange processes.

## Results and Discussion

To probe control elements imparted by redox-active H-bonds in metal complexes, we initiated our studies with 2-(5-(*tert*-butyl)-1*H*-pyrazol-3-yl)­pyridine^28^ (^H^NN^*t*Bu^) as a redox-innocent H-bond donor chelate and targeted implementation of a redox-active H-bond acceptor. The H-bonding interactions imparted by 2-(pyrazol-3-yl)­pyridine platforms have previously been demonstrated to provide unique avenues for small molecule activation and catalysis.^29–34^ Zinc was chosen for our design to alleviate redox events associated with the central metal. To establish an optimum coordination environment featuring intramolecular H-bonding interactions, we investigated multiple X-type ligands of ZnX_2_ (Figure 3). Treating zinc halides (X = Cl, Br) with ^H^NN^*t*Bu^ exclusively formed four-coordinate complexes of the type (^H^NN^*t*Bu^)­ZnX_2_ (**1-X**), regardless of whether one- or two-equiv of the chelating ligand were employed. Similarly, zinc sources featuring either weakly coordinating anions (NO_3_^–^ or ClO_4_^–^) or thiocyanate anions exclusively produced homoleptic zinc dications, [(^H^NN^*t*Bu^)_3_Zn]­[X]_2_ (**2-X**; X = NO_3_^–^, ClO_4_^–^, 1/2 Zn­(SCN)_4_), regardless of whether 2 or 3 equiv of ^H^NN^*t*Bu^ were used. In contrast, metalation of Zn­(OAc)_2_(H_2_O)_2_ with 2 equiv of ^H^NN^*t*Bu^ afforded (^H^NN^*t*Bu^)_2_Zn­(OAc)_2_ (**3**) as identified by ^1^H NMR spectroscopy.

**Figure 3:**
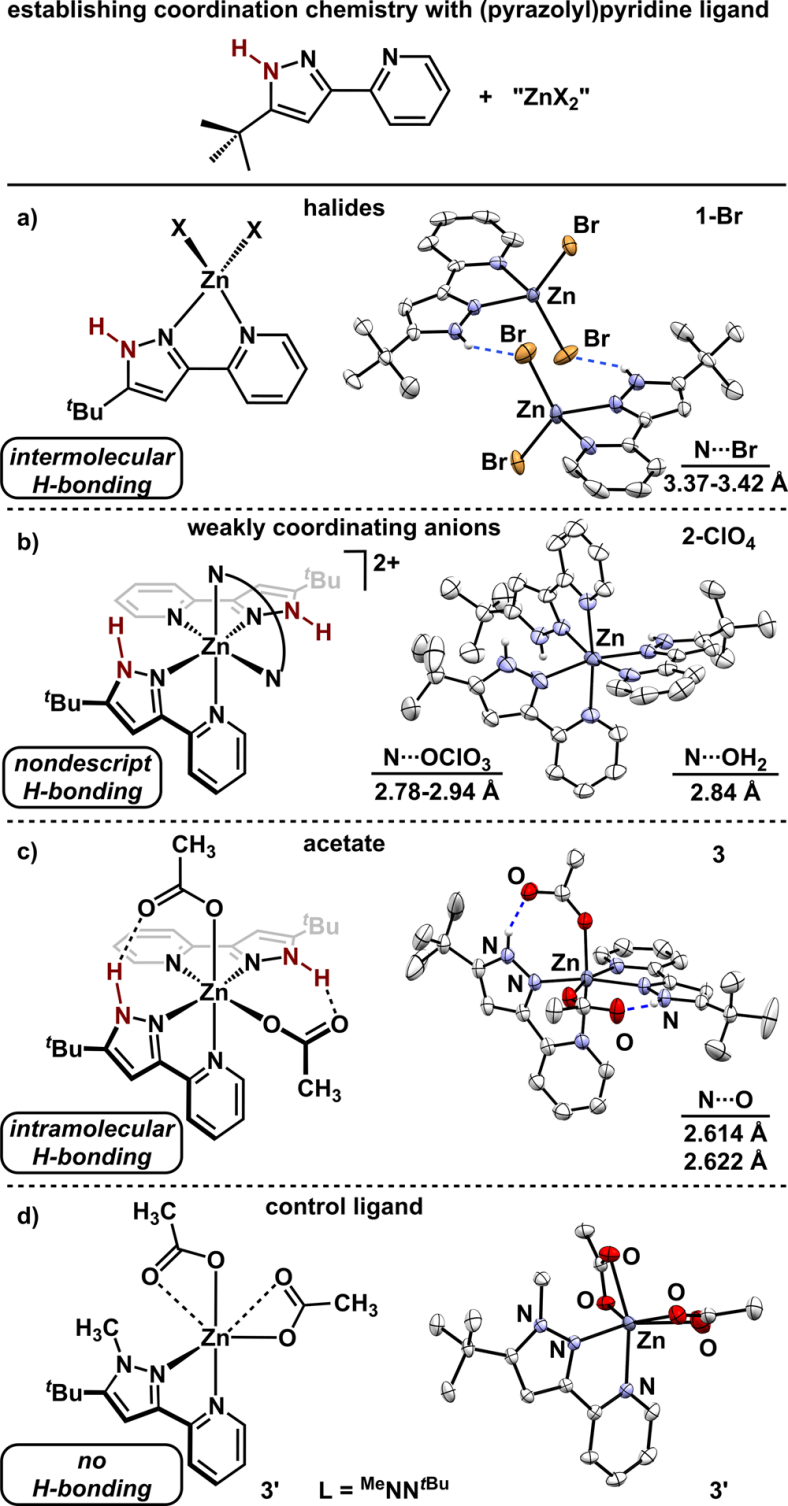
Coordination chemistry of ^H^NN^*t*Bu^ with various zinc sources to generate **1-X** (a), **2-X** (b), and **3** (c). (d) Generation of **3′** using control ligand, ^Me^NN^*t*Bu^. All molecular structures displayed with 50% probability ellipsoids. H atoms not attached to heteroatoms and noncoordinating anions are omitted for clarity.

To interrogate the coordination environment of each set of molecules (**1**, **2**, and **3**) and to probe potential H-bonding interactions, each molecule was investigated by single crystal X-ray diffraction studies. Complexes **1-Cl** and **1-Br** display nearly tetrahedral coordination environments^35^ (τ_4_ = 0.87)^36^ and exclusively contain intermolecular H-bonding interactions.^37^ In each, the pyrazole N–H engages in a weak H-bond with the halide of an adjacent molecule (N···X = 3.146 Å for Cl; 3.395 Å^38^ for Br).^39^ Similar H-bonding interactions have been observed in metal complexes featuring tridentate variants of the ligand, 2,6-bis­(pyrazolyl)­pyridine.^40,41^ These data confirm that complexes **1-X** are not suitable for investigating intramolecular H-bonds. Similarly, homoleptic complexes **2-X**, are unsuitable for studying intramolecular H-bonding interactions and contain solely ill-defined H-bonding interactions. For example, **2-ClO**_**4**_ displays moderate strength H-bonding interactions^39^ to cocrystallized solvent (H_2_O; N···O = 2.840 Å) and to the noncoordinating anion (N···OClO_3_ = 2.78-2.94 Å). Data refinement of single crystals of **3** revealed a pseudo-octahedral coordination environment with cis-acetate ligands (92.53(7)°). Related manganese complexes have been previously reported.^42,43^ For each acetate ligand, one oxygen atom is κ^1^-coordinated to zinc (Zn–O = 2.0510(12), 2.0742(12) Å) while the other oxygen atom engages in a medium strength^39^ intramolecular H-bonding interaction with the pyrazole (N···O_ave_ = 2.614, 2.622 Å). These data suggest that carboxylate X-type ligands may serve as a suitable scaffold for implementing a redox-active H-bond acceptor.

To further understand the H-bonding interactions in **1–3**, each were investigated by infrared spectroscopy. Complexes **1** each display a single, well-defined N–H absorption in the solid state (ATR; **1-Cl** = 3188; **1-Br** = 3219 cm^–1^).^44^ The lower energy absorption observed for **1-Cl** is consistent with a stronger intermolecular H-bonding interaction compared to its bromide counterpart (Δ = 31 cm^–1^).^45^ Homoleptic complexes **2-ClO**_**4**_ and **2-NCS** were each investigated in the solid state (ATR): whereas **2-ClO**_**4**_ displays a single, well-defined N–H stretch (3289 cm^–1^), **2-NCS** displays multiple N–H stretches (3179, 3131 cm^–1^). These observations are consistent with nondescript H-bonding interactions with counteranions and/or cocrystallized solvent molecules as observed in the X-ray structure. In contrast to complexes **1** and **2**, **3** neither displays a clear N–H vibration in the solid state (ATR) nor solution phase (CHCl_3_), but rather, only displays broad features in the N–H region. Ill-defined ν­(N–H) features and nonlinear ^1^H NMR trends have previously been reported for intramolecular H-bonding interactions in related zinc complexes featuring secondary coordination sphere H-bonds.^46^

While each set of molecules, **1**, **2**, and **3**, displays H-bonding interactions, they each possess a vastly different coordination environment at the metal. To investigate the role that the H-bond donor in ^H^NN^*t*Bu^ plays in controlling the coordination environment of the metal, we designed a ligand variant that does not possess the ability to engage in H-bonding. Deprotonation of ^H^NN^*t*Bu^ followed by subsequent alkylation with iodomethane afforded 2-(1-methyl-5-(*tert*-butyl)-1*H*-pyrazol-3-yl)­pyridine (^Me^NN^*t*Bu^). Metalating either 1 or 2 equiv ^Me^NN^*t*Bu^ with Zn­(OAc)_2_(H_2_O)_2_ furnished exclusively (^Me^NN^*t*Bu^)­Zn­(OAc)_2_ (**3′**) regardless of the stoichiometry (Figure 3d). Structures analogous to **3′** have been reported for other group 12 metals with related bidentate ligands, including *N*,*N*,*N’*,*N’*-tetramethylethylenediamine, 2,2′-bipyridine, and 1,10-phenanthroline.^47,48^ The acetate ligands in **3′** are best described as κ^2^ with Zn–O distances ranging 2.0718(11)–2.2785(13) Å. These contrasting metalation results between ^H^NN^*t*Bu^ and ^Me^NN^*t*Bu^ highlight the unique ability of H-bonding interactions to influence the coordination environment at the metal.

The geometric arrangement of **3** suggests that carboxylates are a platform that encourages intramolecular H-bonding interactions. Inspired by the work of Tucker and co-workers where the mild Fc/Fc^+^ couple effected H-bond tuning,^17^ we targeted the replacement of redox-innocent [OAc]^1–^ with redox-active [O_2_CFc]^1–^. Treating a methanol solution of ZnCl_2_ with 2 equiv ^H^NN^*t*Bu^ and 2 equiv potassium ferrocenecarboxylate generated (^H^NN^*t*Bu^)_2_Zn­(O_2_CFc)_2_ (**4**; Fc = C_10_H_9_Fe^II^) as an orange powder in 91% yield (Figure 4). To confirm its structural similarity to **3**, single, X-ray quality crystals that were obtained by diffusing hexane into a toluene solution containing trace methanol were analyzed by diffraction studies. Data refinement revealed a pseudo-octahedral geometry at zinc with *cis*-ferrocenecarboxylate ligands (88.47(6)°). One oxygen of each carboxylate interacts with Zn (Zn–O = 2.0196(14), 2.0452(13) Å) while the other oxygen forms an intramolecular H-bond with a pyrazole N–H (N···O = 2.692, 2.699 Å). These H-bonding distances are slightly longer than those observed for the acetate variant, **3**. To directly assess the role of intramolecular H-bonding in **4**, we synthesized a control complex with ^Me^NN^*t*Bu^ that is incapable of engaging in H-bonding interactions. Under analogous conditions, (^Me^NN^*t*Bu^)­Zn­(O_2_CFc)_2_ (**4′**) was isolated and structurally characterized (Figure 4). Analysis of single X-ray quality crystals obtained on the benchtop revealed the formulation (^Me^NN^*t*Bu^)­Zn­(O_2_CFc)_2_(H_2_O) (**4′-hydrate**). The octahedral zinc hydrate contains one ferrocenecarboxylate that is κ^2^-coordinated (Zn–O: 2.1564(19), 2.2297(18) Å) and a second that is κ^1^-bound. The κ^1^-ferrocenecarboxylate (Zn–O = 2.0902(17) Å) engages in an intramolecular H-bond with Zn-coordinated water. ^1^H NMR and IR spectroscopies of **4′** are consistent with the bound H_2_O ligand being an artifact of crystallization and not being present in bulk isolated samples.

**Figure 4:**
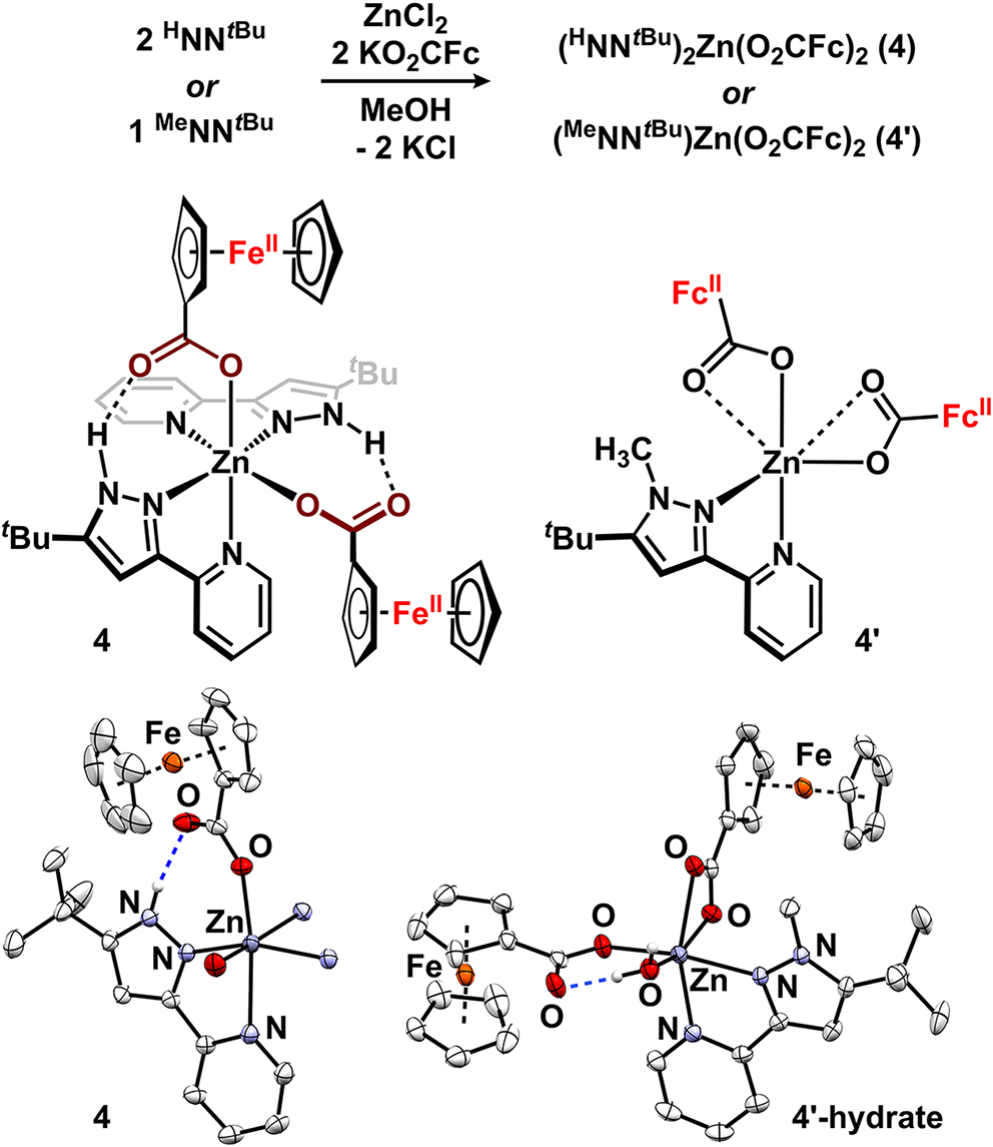
Synthesis of **4** and **4′**. Molecular structures displayed with 50% probability ellipsoids. H atoms not attached to heteroatoms are omitted for improved clarity. The structure of **4** is truncated for improved clarity.

To probe the redox-active component of the H-bond to effect changes in the coordination environment at zinc, the voltammetry of **4** was directly compared to the voltammetry of **4′** (Figure 5a; 0.2 M [Bu_4_N]­[PF_6_], THF; Figures S98 and S99). Cyclic voltammetry of **4** revealed an oxidation wave and a small shoulder on the reverse scan consistent with a chemical equilibrium process.^49^ The redox process is not fully reversible and analysis via square wave (SWV) and differential pulse voltammetries (DPV) suggested the event is composed of two separate oxidations of comparable energy.^50^ These data directly contrast the observations with **4′**: cyclic voltammetry reveals a fully reversible oxidation event while SWV and DPV analyses are consistent with a single two-electron oxidation.^51,52^ The disparate voltammetry results are consistent with **4** undergoing a chemical event upon initial oxidation, whereas **4′** does not undergo a chemical event upon oxidation. Restated: in the absence of a H-bond, bond cleavage does not occur.

**Figure 5:**
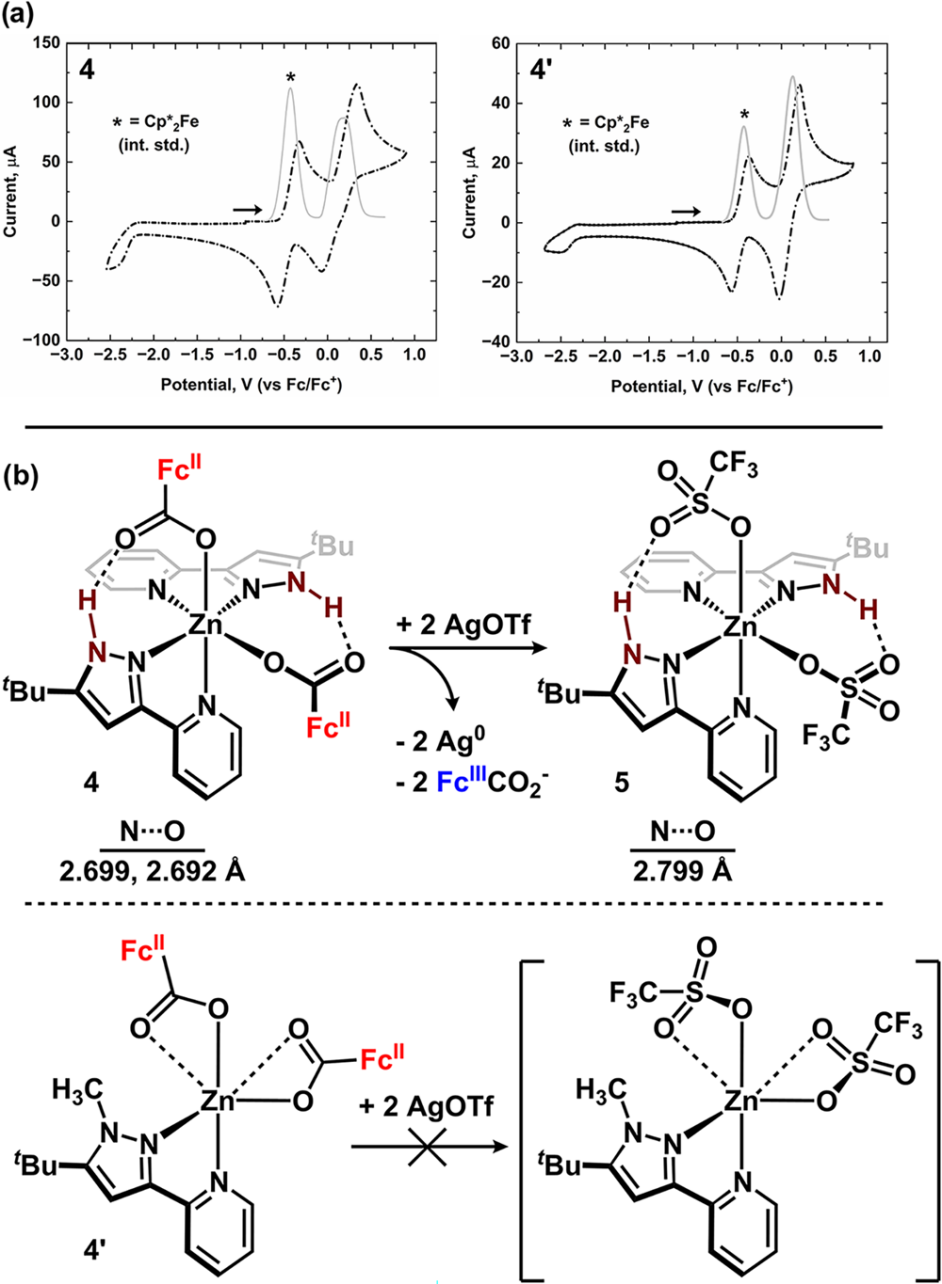
(a) Cyclic voltammograms (0.2 M [Bu_4_N]­[PF_6_], THF, 200 mV/s) of **4** (left) and **4′** (right) overlaid with their square wave voltammograms (gray). The decamethylferrocene internal standard is denoted by the asterisk and the arrow denotes the starting potential. (b) Redox-induced ligand substitution with AgOTf and **4** to generate **5**. The reaction between **4′** and AgOTf does not produce (^Me^NN^*t*Bu^)­Zn­(OTf)_2_.

Electrochemically, we hypothesized that oxidation of the ferrocenecarboxylate ligand in **4** resulted in cleavage of the N–H···O H-bond due to the weakened Lewis basicity of the carboxylate oxygen. This hypothesis is consistent with the irreversible voltammetry and we probed this hypothesis chemically by performing the oxidation in the presence of a competitive X-type ligand. Treating **4** with 2 equiv AgOTf under inert conditions in CH_2_Cl_2_ resulted in a rapid color change from colorless to green (λ_max_ = 633 nm) concomitant with the deposition of silver metal - both consistent with oxidation occurring to form [Fc^III^CO_2_^–^].^53^ ^1^H NMR spectroscopy of the crude mixture revealed the formation of a new diamagnetic species featuring a downfield-shifted resonance at 13.04 ppm, assigned to the pyrazole N–H - a feature not previously observed in **4**. IR analysis of the crude mixture (CH_2_Cl_2_) revealed a strong N–H absorption centered at 3205 cm^–1^. We hypothesized that the new zinc-containing complex was (^H^NN^*t*Bu^)_2_Zn­(OTf)_2_ (**5**) where oxidation of the redox-active H-bond acceptor facilitated ligand exchange by the competitive X-type ligand, trifluoromethanesulfonate (OTf; Figure 5b). (^H^NN^*t*Bu^)_2_Zn­(OTf)_2_ (**5**) was independently synthesized by the addition of 2 equiv ^H^NN^*t*Bu^ to Zn­(OTf)_2_ in CH_2_Cl_2_ under an inert atmosphere. The spectroscopic features of authentic **5** were compared to the in situ generated sample obtained through oxidation and confirmed that the species were identical (Figure S76). Single, X-ray quality crystals of **5** were analyzed by diffraction and revealed an octahedral arrangement at zinc with *cis*-OTf ligands (85.78(8)°). In the solid state, the H-bonding interactions in **5** (2.80 Å) are approximately 0.10 Å longer than those in **4** suggesting an overall weaker binding interaction for [OTf]^1–^.

The chemical oxidation experiment was repeated with **4′** to assess the role that the H-bonding interaction plays in the oxidatively induced ligand substitution reaction (Figure 5b). Treating **4′** with AgOTf (2 equiv) resulted in a gradual color change from colorless to green in analogy to **4** (oxidation to ferrocenium); however, analysis of the crude mixture by ^1^H NMR spectroscopy revealed a paramagnetically broadened spectrum. This finding is inconsistent with the formation of diagmagnetic (^Me^NN^*t*Bu^)­Zn­(OTf)_2_ and instead suggests that [Fc^III^CO_2_^–^] does not dissociate from the metal. Absent the H-bonding interaction, [OTf]^1–^ is unable to displace the ferrocenium carboxylate. Rather, we propose chemical oxidation of **4′** affords the electron transfer product, [(^Me^NN^*t*Bu^)­Zn­(O_2_CFc^III^)_2_]^2+^. This formulation is consistent with the fully reversible voltammetry studies of **4′** that do not allude to a bond-breaking event occurring upon oxidation. Our attempts to isolate (^Me^NN^*t*Bu^)­Zn­(OTf)_2_ for comparative purposes exclusively generated (^Me^NN^*t*Bu^)_2_Zn­(OTf)_2_ (**5′**), regardless of whether 1 or 2 equiv of ^Me^NN^*t*Bu^ were employed in the reaction. Whereas **5** is benchtop stable, **5′** is highly hygroscopic and rapidly degrades to hydrated dications, [(^Me^NN^*t*Bu^)_*x*_Zn­(H_2_O)_*y*_]­[OTf]_2_ (*x* = 1, *y* = 3 or 4; *x* = 2, *y* = 2), when exposed to air, highlighting the driving force that H-bonds provide to induce [OTf]^1–^ binding in **5**.

In the formation of **5** from **4**, ligand substitution by [OTf]^1–^ occurs and is coupled to the oxidation of [Fc^II^CO_2_]^1–^. We performed a series of experiments to assess whether oxidation of the ferrocenecarboxylate ligand in **4** was a requirement for ligand substitution to occur. Treating a CH_2_Cl_2_ solution of **4** with two equiv of a redox-inactive trifluoromethanesulfonate source, [Bu_4_N]­[OTf], was performed as a control reaction (Figure 6a). In situ analysis of the mixture by ^1^H NMR and IR spectroscopies revealed no evidence of the formation of **5** (Figure S78). Similarly, no reaction was observed between **4′** and [Bu_4_N]­[OTf] (2 equiv). Ferrocenyl moieties contain a unique steric profile;^54^ to evaluate whether sterics inhibited [OTf]^1–^ substitution, we assessed a substitution reaction between sterically diminutive **3** and [Bu_4_N]­[OTf]. Similarly, in situ analysis revealed no evidence of [OTf]^1–^ displacing the acetate ligand. These data are consistent with the low-to-modest coordinating ability of [OTf]^1–^,^55^ and, not surprisingly, the reverse reaction between **5** and [Bu_4_N]­[OAc] proceeds to generate **3** (Figure 6b). Through oxidation of [FcCO_2_]^1–^ in **4**, the carboxylate oxygens are rendered less Lewis basic and would therefore have less affinity for H-bonding. We sought to diminish the H-bonding interaction in a redox-inert model by synthesizing a series of benzoate complexes, (^H^NN^*t*Bu^)_2_Zn­(O_2_CAr)_2_ (Ar = *p*-C_6_H_4_X, X = OMe, H, Br; **6-X**), where the Lewis basicity was systematically decreased (ArCO_2_H p*K*_a_ 4.50–3.96).^56^ In separate reactions between each **6-X** and [Bu_4_N]­[OTf], no evidence of the formation of **5** via ligand substitution was obtained (Figure 6a). These data are all consistent with the inability of [OTf]^1–^ to displace the [FcCO_2_]^1–^ ligand in **4** absent oxidation.

**Figure 6:**
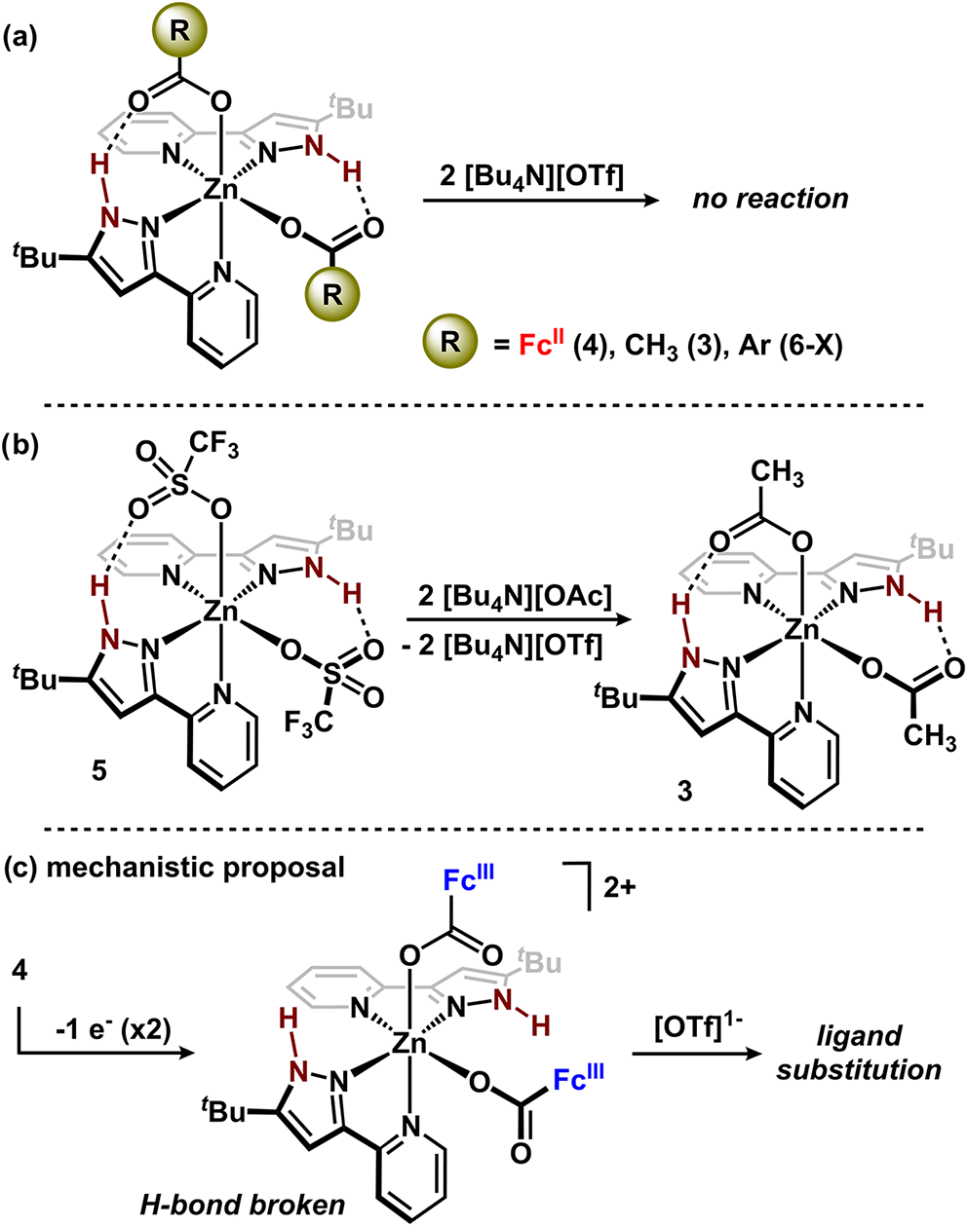
(a) Control reactions to probe requirement of oxidation for ligand substitution to occur. (b) Formation of **3** from **5**. (c) Mechanistic proposal for ligand substitution promoted by redox-controlled H-bonding interactions.

These experimental data support the mechanistic proposal for ligand substitution outlined in Figure 6c. Oxidation of **4** by two single electrons results in H-bond cleavage. Bond breaking upon oxidation is supported by voltammetry studies of **4** in which these events are electrochemically irreversible. H-bond cleavage, rather than Zn–O_2_CFc bond cleavage, is supported by voltammetry studies with **4′** where electrochemical oxidation is fully reversible, suggesting that in the absence of an H-bond, no bond cleavage occurs. Following H-bond cleavage in **4** via oxidation, this species is susceptible to ligand substitution by [OTf]^1–^ to generate **5** and new N–H···O_OTf_ H-bonds. The establishment of new H-bonds in **5** appear to be a driving force for ligand substitution to occur as an analogous species, **5′**, was not obtainable through chemical oxidation of **4′** with AgOTf.

## Conclusion

This example of utilizing redox-active hydrogen bond acceptors, applied to promoting exchange reactions in coordination complexes, complements electrochemical solution assembly studies by Smith and co-workers where redox-active H-bonds controlled assembly between ureas (redox-inert H-bond donor) and quinones (redox-active H-bond acceptor).^57–59^ In this present study, the specific weight attributed to N–H···O bond cleavage versus Zn···O bond weakening to enable ligand substitution is unknown, though the driving force to reform new H-bonds in **5** is a key factor. Importantly, this study establishes that redox-active H-bonding components can be integrated into metal–ligand frameworks, and those with mild redox potentials (∼0.18 V vs Fc/Fc^+^) can perturb the coordination environment at the metal. Here, perturbation enables facile ligand substitution by a competitive Lewis base. Future studies are aimed at redesign strategies to decouple enthalpic contributions of the H-bond versus the Lewis basicity decreases imparted by oxidation.

## Experimental Section

Full synthetic methods for each molecule and instrumentation are detailed in the Supporting Information.

### Synthesis of (^H^NN^tBu^)_2_Zn­(O_2_CFc)_2_ (4)

Open to air, a 20 mL scintillation vial was charged with KO_2_CFc (0.150 g, 0.559 mmol), 6 mL of MeOH, and a stir bar. A separate 20 mL scintillation vial was charged with 2-(5-(*tert*-butyl)-1*H*-pyrazol-3-yl)­pyridine (0.115 g, 0.571 mmol) and 3 mL of MeOH. A third 20 mL scintillation vial was charged with ZnCl_2_ (0.037 g, 0.271) in 1 mL of methanol. While stirring, the solution of 2-(5-(*tert*-butyl)-1*H*-pyrazol-3-yl)­pyridine was added dropwise to the ZnCl_2_ solution, and the mixture stirred for ∼5 min. The solution of KO_2_CFc was then added dropwise, and the mixture was stirred for 22 h. Volatiles were removed and the mixture was redissolved in 3 mL of dichloromethane and filtered. Volatiles were removed, and the resulting solid was washed with 3 × 20 mL of hexane and dried to afford an orange solid (0.231 g, 0.248 mmol, 91%) assigned as (^H^NN^*t*Bu^)_2_Zn­(O_2_CFc)_2_. Single, X-ray quality crystals were obtained by the slow diffusion of hexanes into a toluene solution of (^H^NN^*t*Bu^)_2_Zn­(O_2_CFc)_2_ containing trace MeOH at room temperature. ^1^H NMR (CDCl_3_, 60 MHz, 25 °C): δ = 1.46 (s, 9H, C­(C*H*_3_)_3_), 4.04 (s, 5H, Cp-C*H*), 4.21 (s, 2H, Cp-C*H*), 4.70 (s, 2H, Cp-C*H*), 6.58 (s, 1H, pz-C*H*), 7.16–7.47 (m, 1H, pyr-C*H*), 7.59–7.95 (m, 2H, pyr-C*H*), 8.38 (s, 1H, pyr-C*H*), 12.10 (broad, 1H, N*H*). ^13^C­{^1^H} NMR (CDCl_3_, 15 MHz, 25 °C): δ = 30.55 (C­(*C*H_3_)_3_), 31.95 (*C*(CH_3_)_3_), 69.46 (Cp-*C*H), 70.86 (Cp-C*H*), 98.78 (pz-*C*H), 102.48 (Ar-*C*), 108.06 (Ar-*C*), 119.67 (pyr-*C*H), 123.35 (pyr-*C*H), 138.02 (pyr-*C*H), 148.28 (pyr-*C*H), 157.71 (Ar-*C*), 177.73 (*C*=O). IR (ATR, neat): ν = 2961, 1605, 1466, 1385, 1356, 1346, 1305, 1106, 1001, 988, 776, 720, 694 cm^–1^. IR (CDCl_3_): ν = 2969, 1606, 1574, 1536, 1470, 1389, 1357, 1265 cm^–1^. IR (CH_2_Cl_2_): ν = 2968, 1605, 1573, 1535, 1468, 1387, 1357 cm^–1^. UV Vis (THF, ambient temperature): λ_max_ = 434 nm; ε = 368 ± 4 M^–1^ cm^–1^. MS (monoisotopic mass) of C_46_H_48_N_6_O_4_Fe_2_Zn_1_–(FcCO_2_^–^): Calc. = 695.1775 Da; Obs. = 695.27 Da. MS (monoisotopic mass) of C_46_H_48_N_6_O_4_Fe_2_Zn_1_ (FcCO_2_^–^ + FcCO_2_H): Calc. = 465.1745 Da; Obs. = 465.20 Da.

## Supplementary Material



## Notes

Full experimental procedures and spectroscopic characterization of all species and crystal structure data (PDF)
