# Supplementary material for: A Redox-Active Hydrogen Bond Acceptor Enables Ligand Exchange in a Zinc Complex
Source: Inorg Chem. 2025 Nov 17;64(47):23338–44. doi: 10.1021/acs.inorgchem.5c04839 (PMC12673585; doi:10.1021/acs.inorgchem.5c04839)
Supplement: Supplementary file 1 [file ic5c04839_si_001.pdf]

Supporting Information for:

A Redox-Active Hydrogen Bond Acceptor Enables Ligand Exchange in a Zinc Complex

*Christin N. Gilchrist,[a] Matthias Zeller,[b] and John J. Kiernicki[a]**

[a]Department of Chemistry, Drury University, Springfield, Missouri 65802, United States

[b]H.C. Brown Laboratory, James Tarpo Jr. and Margaret Tarpo Department of Chemistry, Purdue University, West Lafayette, Indiana 47907, United States

Experimental Procedures....S5-S14
Figure S1 ^{1}H NMR spectrum of 2-ethylpicolinate S15
Figure S2 $^{13}C\{^{1}H\}$ NMR spectrum of 2-ethylpicolinate S15
Figure S3 Infrared spectrum (ATR) of 2-ethylpicolinate S16
Figure S4 ^{1}H NMR spectrum of $^{H}NN^{tBu}$ in $CDCl_3$ S16
Figure S5 ^{1}H NMR spectrum of $^{H}NN^{tBu}$ in CH_3OH S17
Figure S6 Infrared spectrum ($CHCl_3$) of $^{H}NN^{tBu}$ S17
Figure S7 Infrared spectrum (ATR) of $^{H}NN^{tBu}$ S18
Figure S8 ^{1}H NMR spectrum of $^{Me}NN^{tBu}$ S18
Figure S9 $^{13}C\{^{1}H\}$ NMR spectrum of $^{Me}NN^{tBu}$ S19
Figure S10 Infrared spectrum (ATR) of $^{Me}NN^{tBu}$ S19
Figure S11 Mass spectrum of $^{Me}NN^{tBu}$ S20
Figure S12 Crude ^{1}H NMR spectrum (CH_3OH) of synthesis of $(^{H}NN^{tBu})ZnCl_2$ (**1-Cl**) S20
Figure S13 ^{1}H NMR spectrum ($CDCl_3$) of $(^{H}NN^{tBu})ZnCl_2$ (**1-Cl**) S21
Figure S14 $^{13}C\{^{1}H\}$ NMR spectrum of $(^{H}NN^{tBu})ZnCl_2$ (**1-Cl**) S21
Figure S15 ^{1}H-^{1}H COSY spectrum of $(^{H}NN^{tBu})ZnCl_2$ (**1-Cl**) S22
Figure S16 Mass spectrum of $(^{H}NN^{tBu})ZnBr_2$ (**1-Br**) S22
Figure S17 Infrared spectra overlay (ATR) of $(^{H}NN^{tBu})ZnX_2$ (**1-X**; X = Br, Cl) S23
Figure S18 ^{1}H NMR spectrum (acetone-d_6) of $[(^{H}NN^{tBu})_3Zn][ClO_4]_2$ (**2-ClO$_4$**) S23
Figure S19 ^{1}H NMR spectrum ($CDCl_3$) of $[(^{H}NN^{tBu})_3Zn][ClO_4]_2$ (**2-ClO$_4$**) S24
Figure S20 $^{13}C\{^{1}H\}$ NMR spectrum of $[(^{H}NN^{tBu})_3Zn][ClO_4]_2$ (**2-ClO$_4$**) S24
Figure S21 ^{1}H-^{1}H COSY spectrum of $[(^{H}NN^{tBu})_3Zn][ClO_4]_2$ (**2-ClO$_4$**) S25
Figure S22 Infrared spectrum (ATR) of $[(^{H}NN^{tBu})_3Zn][ClO_4]_2$ (**2-ClO$_4$**) S25
Figure S23 Infrared spectrum ($CDCl_3$) of $[(^{H}NN^{tBu})_3Zn][ClO_4]_2$ (**2-ClO$_4$**) S26
Figure S24 ^{1}H NMR spectrum of $[(^{H}NN^{tBu})_3Zn][Zn(SCN)_4]$ (**2-SCN**) S26
Figure S25 $^{13}C\{^{1}H\}$ NMR spectrum of $[(^{H}NN^{tBu})_3Zn][Zn(SCN)_4]$ (**2-SCN**) S27
Figure S26 Infrared spectrum (ATR) of $[(^{H}NN^{tBu})_3Zn][Zn(SCN)_4]$ (**2-SCN**) S27
Figure S27 Infrared spectrum ($CDCl_3$) of $[(^{H}NN^{tBu})_3Zn][Zn(SCN)_4]$ (**2-SCN**) S28
Figure S28 ^{1}H NMR spectrum of $(^{H}NN^{tBu})_2Zn(OAc)_2$ (**3**) S28
Figure S29 $^{13}C\{^{1}H\}$ NMR spectrum of $(^{H}NN^{tBu})_2Zn(OAc)_2$ (**3**) S29
Figure S30 ^{1}H-^{1}H COSY spectrum of $(^{H}NN^{tBu})_2Zn(OAc)_2$ (**3**) S29
Figure S31 ^{1}H NMR spectrum ($CDCl_3$) of $(^{H}NN^{tBu})_2Zn(OAc)_2$ (**3**) S30
Figure S32 ^{1}H NMR spectrum (CD_3OD) of $(^{H}NN^{tBu})_2Zn(OAc)_2$ (**3**) S30
Figure S33 Crude ^{1}H NMR spectrum (CH_3OH) of $(^{H}NN^{tBu})_2Zn(OAc)_2$ (**3**) S31
Figure S34 Infrared spectrum (ATR) of $(^{H}NN^{tBu})_2Zn(OAc)_2$ (**3**) S31
Figure S35 Infrared spectrum ($CDCl_3$) of $(^{H}NN^{tBu})_2Zn(OAc)_2$ (**3**) S32
Figure S36 ^{1}H NMR spectrum ($CDCl_3$) of $(^{Me}NN^{tBu})Zn(OAc)_2$ (**3’**) S32
Figure S37 $^{13}C\{^{1}H\}$ NMR spectrum ($CDCl_3$) of $(^{Me}NN^{tBu})Zn(OAc)_2$ (**3’**) S33
Figure S38 Infrared spectrum (ATR) of $(^{Me}NN^{tBu})Zn(OAc)_2$ (**3’**) S33
Figure S39 Mass spectrum of $(^{Me}NN^{tBu})Zn(OAc)_2$ (**3’**) S34
Scheme S1 Attempted metalation with 2-(4-(*tert*-butyl)-pyrazol-1-yl)pyridine S34
Figure S40 ^{1}H NMR spectrum of $(^{H}NN^{tBu})_2Zn(O_2CFc)_2$ (**4**; Fc = ferrocene) S35
Figure S41 $^{13}C\{^{1}H\}$ NMR spectrum of $(^{H}NN^{tBu})_2Zn(O_2CFc)_2$ (**4**; Fc = ferrocene) S35
Figure S42 Infrared spectrum (ATR) of $(^{H}NN^{tBu})_2Zn(O_2CFc)_2$ (**4**; Fc = ferrocene) S36
Figure S43 Infrared spectrum ($CDCl_3$) of $(^{H}NN^{tBu})_2Zn(O_2CFc)_2$ (**4**; Fc = ferrocene) S36
Figure S44 Infrared spectrum (CH_2Cl_2) of $(^{H}NN^{tBu})_2Zn(O_2CFc)_2$ (**4**; Fc = ferrocene) S37
Figure S45 Mass spectrum of $(^{H}NN^{tBu})_2Zn(O_2CFc)_2$ (**4**; Fc = ferrocene) S37
Figure S46 ^{1}H NMR spectrum of $(^{Me}NN^{tBu})Zn(O_2CFc)_2$ (**4’**; Fc = ferrocene) S38
Figure S47 $^{13}C\{^{1}H\}$ NMR spectrum of $(^{Me}NN^{tBu})Zn(O_2CFc)_2$ (**4’**; Fc = ferrocene) S38
Figure S48 Mass spectrum of $(^{Me}NN^{tBu})Zn(O_2CFc)_2$ (**4’**; Fc = ferrocene) S39
Figure S49 Infrared spectrum (ATR) of $(^{Me}NN^{tBu})Zn(O_2CFc)_2$ (**4’**; Fc = ferrocene) S39
Figure S50 Infrared spectrum (CH_2Cl_2) of $(^{Me}NN^{tBu})Zn(O_2CFc)_2$ (**4’**; Fc = ferrocene) S40
Figure S51 ^{1}H NMR spectrum of $(^{H}NN^{tBu})_2Zn(OTf)_2$ (**5**) S40
Figure S52 $^{13}C\{^{1}H\}$ NMR spectrum of $(^{H}NN^{tBu})_2Zn(OTf)_2$ (**5**) S41
Figure S53 ^{19}F NMR spectrum of $(^{H}NN^{tBu})_2Zn(OTf)_2$ (**5**) S41
Figure S54 ^{1}H-^{1}H COSY spectrum of $(^{H}NN^{tBu})_2Zn(OTf)_2$ (**5**) S42
Figure S55 Crude ^{1}H NMR spectrum (CH_3OH) of $(^{H}NN^{tBu})_2Zn(OTf)_2$ (**5**) S42
Figure S56 Infrared spectrum (CH_2Cl_2) of $(^{H}NN^{tBu})_2Zn(OTf)_2$ (**5**) S43

Figure S57 Infrared spectrum ($CHCl_3$) of ($^{H}NN^{tBu}$)$_2$Zn(OTf)$_2$ (**5**) ... S43
Figure S58 Infrared spectrum (ATR) of ($^{H}NN^{tBu}$)$_2$Zn(OTf)$_2$ (**5**) ... S44
Figure S59 Crude ^{1}H NMR spectrum (CH_3OH) of [($^{H}NN^{tBu}$)$_3$Zn][ClO_4]$_2$ (**2-ClO$_4$**) ... S44
Figure S60 Comparison of crude ^{1}H NMR spectra (CH_3OH) for ($^{H}NN^{tBu}$)$_2$Zn(OTf)$_2$ (**5**) and [($^{H}NN^{tBu}$)$_3$Zn][ClO_4]$_2$ (**2-ClO$_4$**) ... S45
Figure S61 Comparison of crude ^{1}H NMR spectra (CH_3OH) for ($^{H}NN^{tBu}$)$_2$Zn(OTf)$_2$ (**5**) and authentic $^{H}NN^{tBu}$ ... S45
Figure S62 Comparison of ^{1}H NMR spectra ($CDCl_3$) for $^{Me}NN^{tBu}$ + Zn(OTf)$_2$ and authentic $^{Me}NN^{tBu}$ ... S46
Figure S63 ^{1}H NMR spectrum of ($^{Me}NN^{tBu}$)$_2$Zn(OTf)$_2$ (**5’**) ... S46
Figure S64 ^{19}F NMR spectrum of ($^{Me}NN^{tBu}$)$_2$Zn(OTf)$_2$ (**5’**) ... S47
Figure S65 Mass spectrum from attempted benchtop synthesis of ($^{Me}NN^{tBu}$)Zn(OTf)$_2$ ... S47
Figure S66 Infrared spectrum (ATR) from attempted benchtop synthesis of ($^{Me}NN^{tBu}$)Zn(OTf)$_2$ ... S48
Figure S67 Infrared spectrum (KBr) of ($^{Me}NN^{tBu}$)$_2$Zn(OTf)$_2$ (**5’**) ... S48
Figure S68 Infrared spectrum (CH_2Cl_2) of ($^{Me}NN^{tBu}$)$_2$Zn(OTf)$_2$ (**5’**) ... S49
Figure S69 Electronic absorption spectra of ($^{H}NN^{tBu}$)$_2$Zn(O_2CFc)$_2$ (**4**; Fc = ferrocene) ... S49
Figure S70 Electronic absorption spectrum of crude reaction between ($^{H}NN^{tBu}$)$_2$Zn(O_2CFc)$_2$ (**4**) and AgOTf ... S50
Figure S71 Electronic absorption spectra of ($^{Me}NN^{tBu}$)Zn(O_2CFc)$_2$ (**4’**; Fc = ferrocene) ... S50
Figure S72 Electronic absorption spectrum of crude reaction between ($^{Me}NN^{tBu}$)Zn(O_2CFc)$_2$ (**4’**) and AgOTf ... S51
Figure S73 Infrared spectrum (CH_2Cl_2) of crude reaction between ($^{Me}NN^{tBu}$)Zn(O_2CFc)$_2$ (**4’**) and AgOTf ... S51
Figure S74 ^{1}H NMR spectrum of crude reaction between ($^{Me}NN^{tBu}$)Zn(O_2CFc)$_2$ (**4’**) and AgOTf ... S52
Figure S75 Overlay of ^{1}H NMR spectra of crude reaction between **4’** and AgOTf vs **5’** ... S52
Figure S76 Infrared spectra overlay (CH_2Cl_2) of crude reaction between ($^{H}NN^{tBu}$)$_2$Zn(O_2CFc)$_2$ (**4**) and AgOTf ... S53
Figure S77 Infrared spectra overlay (KBr/ATR) of crude reaction between ($^{H}NN^{tBu}$)$_2$Zn(O_2CFc)$_2$ (**4**) and AgOTf ... S53
Figure S78 Infrared spectra overlay (CH_2Cl_2) of control reaction between ($^{H}NN^{tBu}$)$_2$Zn(O_2CFc)$_2$ (**4**) and [Bu_4N][OTf] ... S54
Figure S79 Infrared spectra overlay (KBr/ATR) of control reaction between ($^{H}NN^{tBu}$)$_2$Zn(O_2CFc)$_2$ (**4**) and [Bu_4N][OTf] ... S54
Figure S80 Overlay of ^{1}H NMR spectra of **5** + [Bu_4N][OAc] ... S55
Figure S81 Overlay of infrared spectra of **5** + [Bu_4N][OAc] ... S56
Figure S82 Overlay of ^{1}H NMR spectra of **4’** + [Bu_4N][OTf] ... S57
Figure S83 ^{1}H NMR spectrum of ($^{H}NN^{tBu}$)$_2$Zn(O_2CPh)$_2$ (**6-H**) ... S57
Figure S84 ^{13}C{^{1}H} NMR spectrum of ($^{H}NN^{tBu}$)$_2$Zn(O_2CPh)$_2$ (**6-H**) ... S58
Figure S85 Mass spectrum of ($^{H}NN^{tBu}$)$_2$Zn(O_2CPh)$_2$ (**6-H**) ... S58
Figure S86 ^{1}H NMR spectrum of ($^{H}NN^{tBu}$)$_2$Zn(O_2CAr)$_2$ (**6-Br**; Ar = *p*-C_6H_4Br) ... S59
Figure S87 ^{13}C{^{1}H} NMR spectrum of ($^{H}NN^{tBu}$)$_2$Zn(O_2CAr)$_2$ (**6-Br**; Ar = *p*-C_6H_4Br) ... S59
Figure S88 Mass spectrum of ($^{H}NN^{tBu}$)$_2$Zn(O_2CAr)$_2$ (**6-Br**; Ar = *p*-C_6H_4Br) ... S60
Figure S89 ^{1}H NMR spectrum of ($^{H}NN^{tBu}$)$_2$Zn(O_2CAr)$_2$ (**6-OMe**; Ar = *p*-C_6H_4OMe) ... S60
Figure S90 ^{13}C{^{1}H} NMR spectrum of ($^{H}NN^{tBu}$)$_2$Zn(O_2CAr)$_2$ (**6-OMe**; Ar = *p*-C_6H_4OMe) ... S61
Figure S91 Mass spectrum of ($^{H}NN^{tBu}$)$_2$Zn(O_2CAr)$_2$ (**6-OMe**; Ar = *p*-C_6H_4OMe) ... S61
Figure S92 Infrared spectra overlay (ATR) of ($^{H}NN^{tBu}$)$_2$Zn(O_2CAr)$_2$ (**6-X**; Ar = *p*-C_6H_4X; X = Br, H, OMe) ... S62
Figure S93 Infrared spectra overlay (ATR, zoomed) of ($^{H}NN^{tBu}$)$_2$Zn(O_2CAr)$_2$ (**6-X**; Ar = *p*-C_6H_4X; X = Br, H, OMe) ... S62
Figure S94 Infrared spectra overlay (CH_2Cl_2) of ($^{H}NN^{tBu}$)$_2$Zn(O_2CAr)$_2$ (**6-X**; Ar = *p*-C_6H_4X; X = Br, H, OMe) ... S63
Figure S95 Overlay of infrared spectra of **6-Br** + [Bu_4N][OTf] ... S64
Figure S96 Overlay of infrared spectra of **6-H** + [Bu_4N][OTf] ... S65
Figure S97 Overlay of infrared spectra of **6-OMe** + [Bu_4N][OTf] ... S66
Figure S98 Electrochemical experiments with ($^{H}NN^{tBu}$)$_2$Zn(O_2CFc)$_2$ (**4**) ... S67
Figure S99 Electrochemical experiments with ($^{Me}NN^{tBu}$)Zn(O_2CFc)$_2$ (**4’**) ... S68
Crystallographic Details ... S69-S100
Table S1 Experimental parameters for ($^{H}NN^{tBu}$)ZnBr$_2$ (**1-Br**) ... S69
Figure S100 Molecular structure of ($^{H}NN^{tBu}$)ZnBr$_2$ (**1-Br**) ... S70
Table S2 Experimental parameters for ($^{H}NN^{tBu}$)ZnCl$_2$ (**1-Cl**) ... S71
Figure S101 Molecular structure of ($^{H}NN^{tBu}$)ZnCl$_2$ (**1-Cl**) ... S72
Table S3 Experimental parameters for [($^{H}NN^{tBu}$)$_3$Zn][ClO_4]$_2$ (**2-ClO$_4$**) ... S73
Figure S102 Molecular structure of [($^{H}NN^{tBu}$)$_3$Zn][ClO_4]$_2$ (**2-ClO$_4$**) ... S74
Table S4 Experimental parameters for [($^{H}NN^{tBu}$)$_3$Zn][Zn(SCN)$_4$] (**2-SCN**) ... S75
Figure S103 Molecular structure of [($^{H}NN^{tBu}$)$_3$Zn][Zn(SCN)$_4$] (**2-SCN**) ... S76
Table S5 Experimental parameters for ($^{H}NN^{tBu}$)$_2$Zn(OAc)$_2$ (**3**) ... S77
Figure S104 Molecular structure of ($^{H}NN^{tBu}$)$_2$Zn(OAc)$_2$ (**3**) ... S78
Table S6 Experimental parameters for ($^{Me}NN^{tBu}$)Zn(OAc)$_2$ (**3’**) ... S79
Figure S105 Molecular structure of ($^{Me}NN^{tBu}$)Zn(OAc)$_2$ (**3’**) ... S80
Table S7 Experimental parameters for ($^{H}NN^{tBu}$)$_2$Zn(O_2CFc)$_2$ (**4**) ... S81
Figure S106 Molecular structure of ($^{H}NN^{tBu}$)$_2$Zn(O_2CFc)$_2$ (**4**) ... S82

Table S8 Experimental parameters for ($^{Me}NN^{tBu}$)Zn($O_2CFc)_2(H_2O$) (**4’-hydrate**) S83
Figure S107 Molecular structure of ($^{Me}NN^{tBu}$)Zn($O_2CFc)_2(H_2O$) (**4’-hydrate**) S84
Table S9 Experimental parameters for ($^{H}NN^{tBu})_2$Zn(OTf)$_2$ (**5**) S85
Figure S108 Molecular structure of ($^{H}NN^{tBu})_2$Zn(OTf)$_2$ (**5**) S86
Table S10 Experimental parameters for ($^{Me}NN^{tBu})_2$Zn(OTf)$_2$ (**5’**) S87
Figure S109 Molecular structure of ($^{Me}NN^{tBu})_2$Zn(OTf)$_2$ (**5’**) S88
Table S11 Experimental parameters for [($^{Me}NN^{tBu}$)Zn($H_2O)_n$][OTf]$_2$ S89
Figure S110 Molecular structure of [($^{Me}NN^{tBu}$)Zn($H_2O)_n$][OTf]$_2$ S90
Table S12 Experimental parameters for [($^{Me}NN^{tBu})_2$Zn($H_2O)_2$][OTf]$_2$ S91
Figure S111 Molecular structure of [($^{Me}NN^{tBu})_2$Zn($H_2O)_2$][OTf]$_2$ S92
Table S13 Experimental parameters for ($^{H}NN^{tBu})_2$Zn(O_2CAr)$_2$ (**6-OMe**; Ar = *p*-C_6H_4OMe) S93
Figure S112 Molecular structure of ($^{H}NN^{tBu})_2$Zn(O_2CAr)$_2$ (**6-OMe**; Ar = *p*-C_6H_4OMe) S94
Table S14 Experimental parameters for ($^{H}NN^{tBu})_2$Zn(O_2CPh)$_2$ (**6-H**) S95
Figure S113 Molecular structure of ($^{H}NN^{tBu})_2$Zn(O_2CPh)$_2$ (**6-H**) S96
Table S15 Experimental parameters for ($^{H}NN^{tBu})_2$Zn(O_2CAr)$_2$ (**6-Br**; Ar = *p*-C_6H_4Br) S97
Figure S114 Molecular structure of ($^{H}NN^{tBu})_2$Zn(O_2CAr)$_2$ (**6-Br**; Ar = *p*-C_6H_4Br) S98
Table S16 Experimental parameters for (L)Zn_4O(OAc)$_6$ S99
Figure S115 Molecular structure of (L)Zn_4O(OAc)$_6$ S100
Table S17 Metrical parameters for ($^{H}NN^{tBu}$)ZnX_2 (**1-X**; X = Br, Cl) S101
Table S18 Metrical parameters for ($^{R}NN^{tBu})_2$ZnX_2 (**3**, **4**, **4’**, **5**, **5’**, **6-OMe**, **6-H**, **6-Br**) S101
Table S19 Metrical parameters for homoleptic complexes, [($^{H}NN^{tBu})_3$Zn]$^{2+}$ (**2-X**) S101
Table S20 Metrical parameters for zinc acetate complexes S102
Table S21 Metrical parameters for $^{Me}NN^{tBu}$ containing complexes S102
Table S22 Metrical parameters and N-H resonances (^{1}H NMR) for $^{H}NN^{tBu}$ containing complexes S103
References S104

General Considerations. No uncommon hazards are noted in the syntheses. Unless specifically noted, all manipulations were performed open to atmospheric air on the benchtop. Any air- and moisture-sensitive manipulations were performed using standard Schlenk techniques or in an inert atmosphere drybox with an atmosphere of purified nitrogen.

Solvent preparation for sensitive reactions: THF was dried over Na/benzophenone and vacuum transferred; hexane, dichloromethane, and $CDCl_3$ were dried over CaH_2 and vacuum transferred. All solvents for non-sensitive reactions, including chloroform-*d* and DMSO-d_6, were purchased from commercial suppliers and used as received.

$Zn(OAc)_2(H_2O)_2$, $Zn(ClO_4)_2{\cdot}6H_2O$, $Zn(ClO_4)_2{\cdot}6H_2O$, NaSCN, $ZnCl_2$, $ZnBr_2$, $Zn(OTf)_2$, AgOTf, $[Bu_4N][OTf]$, iodomethane, sodium hydride, 1*H*-4-*tert*-butylpyrazole, and 2-bromopyridine were purchased from commercial suppliers and used as received. The following compounds were prepared according to literature procedure: $[Zn(O_2CAr)_2]_n$ (Ar = *p*-C_6H_4X; X = OCH_3, H, Br),[1] ferrocene carboxylic acid,[2] and 2-(5-(*tert*-butyl)-1*H*-pyrazol-3-yl)pyridine.[3]

NMR spectra were recorded on a Varian Vnmrs 700 spectrometer, a Varian MR400 spectrometer, or a Magritek Spinsolve 60 Carbon spectrometer. ^1H and ^{13}C chemical shifts are reported in parts per million (ppm) relative to tetramethylsilane and referenced internally to the residual solvent peak. Multiplicities are reported as follows: singlet (s), doublet (d), triplet (t), quartet (q), pentet (p). Paramagnetic resonances are reported in ppm followed by the full-width at half-max. ^{19}F chemical shifts are reported in ppm relative to $CFCl_3$ and are internally referenced. Infrared spectra were recorded using a PerkinElmer Spectrum 3 FT-IR spectrometer. Samples were either a) diluted into dry KBr and recorded as pellets, 2) collected as neat solids by ATR, or 3) as solutions. Electronic absorption spectra were obtained with a Vernier VSP-UV UV-VIS spectrophotometer. Electrochemical experiments were performed with GAMRY Instruments Interface 1010B. The tetrabutylammonium hexafluorophosphate electrolyte was triply recrystallized from ethyl acetate and dried in vacuo before use. High resolution liquid chromatography mass spectrometry data (HR LC-MS) were obtained on an Agilent 6230 TOF instrument. Low resolution mass spectrometry data (MS) were collected on a Thermo Scientific TSQ Quantis instrument. All samples were collected in positive ion mode.

Single crystals of $(^{H}NN^{tBu})_2Zn(O_2CPh)_2$, $(^{H}NN^{tBu})_2Zn(O_2CAr)_2$ (Ar = *p*-C_6H_4OMe), $(^{H}NN^{tBu})ZnCl_2$, $(^{H}NN^{tBu})_2Zn(O_2CFc)_2$, $(^{Me}NN^{tBu})Zn(O_2CFc)_2(H_2O)$, $(^{H}NN^{tBu})_2Zn(O_2CAr)_2$ (Ar = *p*-C_6H_4Br), $(^{H}NN^{tBu})_2Zn(OAc)_2$, $(^{H}NN^{tBu})ZnBr_2$, $(^{H}NN^{tBu})_2Zn(OTf)_2$, $(^{Me}NN^{tBu})Zn(OAc)_2$, $(^{Me}NN^{tBu})_2Zn(OTf)_2$, $[(^{Me}NN^{tBu})Zn(H_2O)_n][OTf]_2$ (n = 3, 4), and $(L)Zn_4O(OAc)_6$ suitable for X-ray diffraction were coated with poly(isobutylene) oil and quickly transferred to the goniometer head of a Bruker AXS D8 Quest diffractometer with a fixed chi angle, a sealed tube fine-focus X-ray tube, single crystal curved graphite incident beam monochromator, and a Photon2 CMOS area detector. Examination and data collection were performed with Mo Kα radiation (λ = 0.71073 Å). Single crystals of $[(^{H}NN^{tBu})_3Zn][Zn(SCN)_4]$, $[(^{H}NN^{tBu})_3Zn][ClO_4]_2$, and $[(^{Me}NN^{tBu})_2Zn(H_2O)_2][OTf]_2$ suitable for X-ray diffraction were coated with poly(isobutylene) oil and quickly transferred to the goniometer head of a Bruker AXS D8 Quest diffractometer with kappa geometry, an I-µ-S microsource X-ray tube, laterally graded multilayer (Goebel) mirror for monochromatization, a Photon2 or a Photon3 CMOS area detector, and an Oxford Cryosystems low temperature device. Examination and data collection were performed with Cu Kα radiation (λ = 1.54184 Å). Data were collected, reflections were indexed and processed, and the files scaled and corrected for absorption using APEX3, APEX4, SAINT and/or SADABS.[4] For all samples, the space groups were assigned using XPREP within the SHELXTL suite

of programs,[5] and the structures were solved by direct methods using ShelXS-97[6] and refined by full matrix least squares against F^2 with all reflections using Shelxl2018[7] using the graphical interface Shelxle.[8] If not specified otherwise, H atoms attached to carbon atoms were positioned geometrically and constrained to ride on their parent atoms, with carbon hydrogen bond distances of 0.95 Å for an aromatic C-H, 1.00, 0.99 and 0.98 Å for aliphatic C-H, CH_2, and CH_3 moieties, respectively. Methyl H atoms were allowed to rotate but not to tip to best fit the experimental electron density. Depending on data quality, N-H bond distances were either freely refined or restrained to 0.88 Å. $U_{iso}(H)$ values were set to a multiple of $U_{eq}(C)$ with 1.5 for CH_3, and 1.2 for CH_2, C-H, and N-H units, respectively. Additional data collection and refinement details, including description of disorder (where present) can be found with the individual structure descriptions below. Complete crystallographic data, in CIF format, have been deposited with the Cambridge Crystallographic Data Centre. CCDC 2395143-2395152, 2443233-2443234, and 2495156-2495159 contains the supplementary crystallographic data for this paper. These data can be obtained free of charge from The Cambridge Crystallographic Data Centre via www.ccdc.cam.ac.uk/data_request/cif.

Synthesis of 2-ethylpicolinate. This material was synthesized following a modified literature procedure.[9] A 1 L round bottom flask was charged with 2-picolinic acid (20.183g, 163.945 mmol), toluene (200 mL), ethanol (200 mL, 200 proof), and sulfuric acid (6 mL). The flask was fitted with a Dean-Stark trap and a reflux condenser and the solution was refluxed for 5 days. The Dean-Stark trap was periodically drained during this time. The solution was cooled to 0 °C and neutralized with aqueous sodium bicarbonate until gas evolution ceased. The organics were extracted with Et_2O (3 x 200 mL) and dried over $MgSO_4$. Removal of volatiles afforded light yellow oil (13.826 g, 91.460 mmol, 31%) assigned as 2-ethylpicolinate. ^{1}H NMR ($CDCl_3$, 400 MHz, 25 °C): δ = 1.41 (t, *J* = 7.1, 3H, OCH_2CH_3), 4.45 (q, *J* = 7.2, 2H, OCH_2CH_3), 7.44 (t, *J* = 6.1, 1H, pyr-C*H*), 7.81 (dt, *J* = 1.1, 7.8, 1H, pyr-C*H*), 8.10 (d, *J* = 7.9, 1H, pyr-C*H*), 8.73 (d, *J* = 4.5, 1H, pyr-C*H*). ^{13}C{^{1}H} NMR ($CDCl_3$, 176 MHz, 25 °C): δ = 14.39 (OCH_2CH_3), 62.00 (OCH_2CH_3), 125.15 (pyr-*C*H), 126.87 (pyr-*C*H), 137.04 (pyr-*C*H), 148.34 (pyr-*C*), 149.90 (pyr-*C*H), 165.30 (*C*=O). IR (ATR, neat): ν = 3058, 2983, 1737 (shoulder), 1715 (sharp), 1305, 1127 cm^{-1}.

Synthesis of 2-(5-(*tert*-butyl)-1*H*-pyrazol-3-yl)pyridine (^{H}NN^{tBu}). This material was synthesized following a modified literature procedure.[3] An oven dried 250 mL Schlenk flask was charged with sodium hydride (60% dispersion in mineral oil, 1.376 g, 34.400 mmol) and a stir bar. The flask was attached to a vacuum line and ~60 mL of dry THF was condensed into the flask via vacuum transfer. The flask was then attached to a N_2 manifold, cooled to -78 °C, and treated with pinacolone (3.180 g, 31.749 mmol) against a positive flow of N_2 with a pipet. The flask was warmed to room temperature, fitted with a reflux condenser, and heated to 65-70 °C. A separate 100 mL round bottom flask was charged 2-ethylpicolinate (4.000 g, 26.460 mmol), attached to a vacuum line, and ~20 mL of dry THF was condensed into the flask via vacuum transfer. The flask was removed from the vacuum line, sealed with a septum, and sparged with N_2. The solution of 2-ethylpicolinate was added dropwise via syringe to the boiling NaH/pinacolone solution over the course of 10 minutes. *Caution! Vigorous H_2 gas evolution occurs during this step!* The reaction was stirred an additional 3 hr at 65 °C, cooled to room temperature, and the acidity adjusted to pH = 5 with 1 M HCl (aqueous). The organics were extracted with diethyl ether (4 x 15 mL), washed with brine, and dried over $MgSO_4$. Volatiles were removed to afford a viscous yellow/orange oil that was redissolved in 50 mL of ethanol and heated to reflux. A solution of hydrazine (35% in H_2O; 9 mL dissolved in 30 mL ethanol) was added dropwise to the boiling reaction solution over the course of 15 min. The reaction was refluxed for 2 hr, then volatiles were removed in vacuo to afford dark orange oil. The crude material was purified by column chromatography on silica eluting first with 9:1 hexanes:EtOAc, then 100% EtOAc (R_f = 0.64 in

EtOAc). The material obtained was a light yellow viscous semi-solid. Repeated trituration with hexanes afforded a white solid (2.682 g, 13.325 mmol, 50%) assigned as 2-(5-(*tert*-butyl)-1*H*-pyrazol-3-yl)pyridine. MP = 370 - 375 K. Spectroscopic data were consistent with literature. ^{1}H NMR ($CDCl_3$, 400 MHz, 25 °C): δ = 1.38 (s, 9H, C(C*H*$_3$)$_3$), 6.69 (s, 1H, pz-C*H*), 7.21 (broad, 1H, pyr-C*H*), 7.71-7.80 (m, 2H, pyr-C*H*), 8.60 (broad, 1H, pyr-C*H*). ^{1}H NMR (CH_3OH, 400 MHz, 25 °C): δ = 1.33 (s, 9H, C(C*H*$_3$)$_3$), 6.64 (s, 1H, pz-C*H*), 7.27 (t, *J* = 6.8, 1H, pyr-C*H*), 7.80 (dt, *J* = 1.3, 7.5, 1H, pyr-C*H*), 7.89 (broad, 1H, pyr-C*H*), 8.47 (d, *J* = 3.3, 1H, pyr-C*H*). IR (ATR, neat): ν = 3167, 3135, 3105, 3046, 2963, 2864, 1739, 1596, 1567, 1458, 1411, 1362, 1304, 1277, 1245, 1179, 1152, 1131, 1093, 1076, 1003, 988, 974, 832, 784, 714 cm^{-1}. IR ($CHCl_3$): ν = 3456 (N-H) cm^{-1}. Hi-Res LCMS: Calc. for [$C_{12}H_{15}N_3$] + Na = 224.1164. Obs. = 224.1219. Calc. for [$C_{12}H_{15}N_3$] + H = 202.1344. Obs. = 202.1494.

Synthesis of 2-(1-methyl-5-(*tert*-butyl)-1*H*-pyrazol-3-yl)pyridine ($^{Me}NN^{tBu}$). This material was synthesized following a modified literature procedure.[11] An oven dried 200 mL Schlenk flask was charged with sodium hydride (60% dispersion in mineral oil, 0.537 g, 13.425 mmol) and a stir bar. The flask was attached to a Schlenk line and placed under an N_2 atmosphere. Via cannula, ~50 mL of dry THF was added and the flask cooled to 0 °C. *Caution! Vigorous H_2 gas evolution occurs during the next step!* While stirring, 2-(5-(*tert*-butyl)-1*H*-pyrazol-3-yl)pyridine ($^{H}NN^{tBu}$; 2.000 g, 9.937 mmol) was added as a solid in one portion against a flow of N_2 resulting in immediate effervescence. The flask was warmed to RT and iodomethane (5.642 g, 39.749 mmol) was added via syringe. The flask was equipped with a condenser and the solution was refluxed 24 hr. Volatiles were removed in vacuo. The remaining sludge was extracted into dichloromethane (~50 mL), filtered over Celite, and volatiles were removed to afford dark yellow/orange liquid. The material was purified via column chromatography on silica to afford a light yellow liquid that gradually solidifies to an off-white solid (1.623 g, 7.538 mmol, 76%) identified as 2-(1-methyl-5-(*tert*-butyl)-1*H*-pyrazol-3-yl)pyridine. R_f (2:1 hexane:ethyl acetate): 0.50. R_f (1:2 hexane:ethyl acetate): 0.70. The spectroscopic data is consistent with prior reports.[12] ^{1}H NMR ($CDCl_3$, 60 MHz, 25 °C): δ = 1.43 (s, 9H, C(C*H*$_3$)$_3$), 4.04 (s, 3H, N-C*H*$_3$), 6.74 (s, 1H, pz-C*H*), 7.16 (dt, *J* = 1.5, 5.6, 1H, pyr-C*H*), 7.68 (dt, *J* = 1.5, 7.8, 1H, pyr-C*H*), 7.96 (d, *J* = 7.8, 1H, pyr-C*H*), 8.64 (d, *J* = 4.5, 1H, pyr-C*H*). ^{13}C{^{1}H} NMR ($CDCl_3$, 15 MHz, 25 °C): δ = 22.23 (C(*C*H$_3$)$_3$), 30.88 (*C*(CH$_3$)$_3$), 39.33 (N-*C*H$_3$), 101.95 (Ar-C*H*), 119.26 (Ar-C*H*), 121.68 (Ar-C*H*), 136.04 (Ar-C*H*), 148.92 (Ar-C*H*), 152.21 (Ar-C), 152.36 (Ar-C). IR (ATR, neat): ν = 2966, 1737, 1596, 1566, 1542, 1494, 1465, 1407, 1284, 1273, 1251, 1220, 1187, 1146, 1112, 1091, 1047, 1025, 994, 968, 816, 785, 744, 686 cm^{-1}. MS (monoisotopic mass) of $C_{13}H_{17}N_3$+H: Calc. = 216.1501 Da; Obs. = 216.18 Da. MS (monoisotopic mass) of $C_{13}H_{17}N_3$+Na: Calc. = 238.1320 Da; Obs. = 238.16 Da.

Synthesis of ($^{H}NN^{tBu}$)ZnBr$_2$ (1-Br). A 20 mL scintillation vial was charged with $ZnBr_2$ (0.280 g, 1.243 mmol), 6 mL of MeOH, and a stir bar. A separate 20 mL scintillation vial was charged with 2-(5-(*tert*-butyl)-1*H*-pyrazol-3-yl)pyridine (0.250 g, 1.242 mmol) and 6 mL of MeOH. While stirring, the solution of 2-(5-(*tert*-butyl)-1*H*-pyrazol-3-yl)pyridine was added dropwise to the $ZnBr_2$ solution. After 48 hr, Et_2O was added to induce precipitation (~10 mL). The solid was collected and further washed with Et_2O (2 x 20 mL) to afford white powder (0.159 g, 0.373 mmol, 30%) assigned as ($^{H}NN^{tBu}$)ZnBr$_2$. Single, X-ray quality crystals were obtained by diffusing hexanes into a 1,2-dichlorobenzene solution of ($^{H}NN^{tBu}$)ZnBr$_2$ at room temperature. Isolated samples of ($^{H}NN^{tBu}$)ZnBr$_2$ exhibit too poor of solubility in common NMR solvents ($CDCl_3$, dmso-*d*$_6$) for meaningful analysis. IR (ATR): ν = 3219 (N-H), 1610, 1466, 1448, 1299, 1220, 1156, 1005, 892, 790, 719, 692 cm^{-1}. Solubility in $CHCl_3$/CH_2Cl_2 was too poor for solution IR analysis. MS (monoisotopic mass) of [($^{H}NN^{tBu}$)ZnBr$_2$]$_2$ - Br ($C_{24}H_{30}N_6Br_3Zn_2$): Calc. = 766.8665 Da; Obs. = 767.00 Da. MS (monoisotopic mass) of

$[C_{24}H_{30}N_6Br_3Zn_2]^+$ - $ZnBr_2$: Calc. = 545.1007 Da; Obs. = 545.19 Da. MS (monoisotopic mass) of $[C_{24}H_{30}N_6Br_1Zn_1]^+$ - HBr: Calc. = 465.1745 Da; Obs. = 465.23 Da.

Synthesis of ($^{H}NN^{tBu}$)$ZnCl_2$ (1-Cl). A 20 mL scintillation vial was charged with $ZnCl_2$ (0.170 g, 1.247 mmol), 6 mL of MeOH, and a stir bar. A separate 20 mL scintillation vial was charged with 2-(5-(*tert*-butyl)-1*H*-pyrazol-3-yl)pyridine (0.250 g, 1.242 mmol) and 6 mL of MeOH. While stirring, the solution of 2-(5-(*tert*-butyl)-1*H*-pyrazol-3-yl)pyridine was added dropwise to the $ZnCl_2$ solution. After 48 hr, volatiles were removed in vacuo. The crude material was dissolved in minimal CH_2Cl_2 and hexanes were added to induce precipitation. The solid was collected, washed further with hexanes (2 x 20 mL) to afford white powder (0.381 g, 0.746 mmol, 60%) assigned as ($^{H}NN^{tBu}$)$ZnCl_2$. Single, X-ray quality crystals were obtained by diffusing hexanes into an ethyl acetate solution of ($^{H}NN^{tBu}$)$ZnCl_2$ at room temperature. ^{1}H NMR ($CDCl_3$, 700 MHz, 25 °C): δ = 1.46 (s, 9H, C(C*H*$_3$)$_3$), 6.58 (s, 1H, pz-C*H*), 7.42 (t, *J* = 5.8, 1H, pyr-C*H*), 7.79 (d, *J* = 7.8, 1H, pyr-C*H*), 7.97 (d, *J* = 7.5, 1H, pyr-C*H*), 8.26 (broad, 1H, pyr-C*H*), 13.84 (broad, 1H, N-*H*). ^{1}H NMR (crude analysis, CH_3OH, 400 MHz, 25 °C): 1.36 (s, 9H, C(C*H*$_3$)$_3$), 6.92 (s, 1H, pz-C*H*), 7.47 (t, *J* = 5.4, 1H, pyr-C*H*), 8.02-8.10 (m, 2H, pyr-C*H*), 8.13 (broad, 1H, pyr-C*H*). ^{13}C{^{1}H} NMR ($CDCl_3$, 176 MHz, 25 °C): δ = 30.28 (C(*C*H$_3$)$_3$), 31.89 (*C*(CH$_3$)$_3$), 99.51 (pz-*C*H), 121.04 (pyr-*C*H), 124.80 (pyr-*C*H), 140.00 (pyr-*C*H), 147.28 (Ar-*C*), 148.46 (Ar-*C*), 148.54 (pyr-*C*H), 159.31 (Ar-*C*). IR (ATR, neat): ν = 3188 (N-H), 3144, 3107 cm^{-1}. Hi-Res LCMS: Calc. for $[C_{12}H_{15}N_3Cl_2Zn_1]$ – Cl = 300.0246. Obs. = 300.0237.

Synthesis of [($^{H}NN^{tBu}$)$_3$Zn][(ClO_4)$_2$] (2-ClO$_4$). A 20 mL scintillation vial was charged with $Zn(NO_3)_2$▪$6H_2O$ (0.099 g, 0.333 mmol), 4 mL of MeOH, and a stir bar. A separate 20 mL scintillation vial was charged with 2-(5-(*tert*-butyl)-1*H*-pyrazol-3-yl)pyridine (0.200 g, 0.994 mmol) and 2 mL of MeOH. While stirring, the solution of 2-(5-(*tert*-butyl)-1*H*-pyrazol-3-yl)pyridine was added dropwise to the $Zn(NO_3)_2$▪$6H_2O$ solution. After 5 min, sodium perchlorate (0.101 g, 0.824 mmol) in 2 mL of MeOH was added dropwise. After 21 hr, volatiles were removed in vacuo. The material was dissolved in CH_2Cl_2 (~10 mL), filtered, and volatiles were removed. The solid was washed with hexanes (3 x 20 mL) and dried to afford white solid (0.251 g, 0.289 mmol, 87%) assigned as [($^{H}NN^{tBu}$)$_3$Zn][(ClO_4)$_2$]. Single, X-ray quality crystals were obtained by diffusing hexanes into an ethyl acetate solution of [($^{H}NN^{tBu}$)$_3$Zn][(ClO_4)$_2$] at room temperature. ^{1}H NMR (acetone-*d*$_6$, 700 MHz, 25 °C): δ = 1.46 (s, 9H, C(C*H*$_3$)$_3$), 7.27 (s, 1H, pz-C*H*), 7.66 (broad, 1H, pyr-C*H*), 8.25-8.31 (m, 2H, pyr-C*H*), 8.33 (broad, 1H, pyr-C*H*), 12.80 (s, 1H, N-*H*). ^{1}H NMR ($CDCl_3$, 60 MHz, 25 °C): δ = 1.33 (s, 9H, C(C*H*$_3$)$_3$), 6.56 (s, 1H, pz-C*H*), 7.34-7.67 (m, 1H, pyr-C*H*), 7.70-8.22 (m, 3H, pyr-C*H*), 11.70 (s, 1H, N-*H*). ^{1}H NMR (crude analysis, CH_3OH, 400 MHz, 25 °C): 1.34 (s, 9H, C(C*H*$_3$)$_3$), 6.99 (s, 1H, pz-C*H*), 7.54 (broad, 1H, pyr-C*H*), 8.11 (broad, 3H, pyr-C*H*). ^{13}C{^{1}H} NMR (acetone-*d*$_6$, 176 MHz, 25 °C): δ = 30.04 (C(*C*H$_3$)$_3$), 32.36 (*C*(CH$_3$)$_3$), 101.53 (pz-*C*H), 112.84 (pyr-*C*H), 126.80 (pyr-*C*H), 142.53 (pyr-*C*H), 148.19 (Ar-*C*), 148.91 (Ar-*C*), 149.04 (pyr-*C*H), 160.24 (Ar-*C*). IR (ATR, neat): ν = 3289 (N-H), 2971, 1617, 1576, 1560, 1529, 1471, 1447, 1304, 1259, 1211, 1146, 1116, 1062,1038, 1004, 982, 924, 820, 781, 700 cm^{-1}. IR ($CDCl_3$): ν = 3257 (N-H) cm^{-1}. Hi-Res LCMS: Calc. for $[C_{36}H_{45}N_9Zn_1]$ – [$^{H}NN^{tBu}$ + H] = 465.1745. Obs. = 465.1952.

Synthesis of [($^{H}NN^{tBu}$)$_3$Zn][Zn(NCS)$_4$] (2-SCN). A 20 mL scintillation vial was charged with $ZnCl_2$ (0.018 g, 0.132 mmol), 1 mL of MeOH, and a stir bar. A separate 20 mL scintillation vial was charged with 2-(5-(*tert*-butyl)-1*H*-pyrazol-3-yl)pyridine (0.040 g, 0.199 mmol) and 1 mL of MeOH. While stirring, the solution of 2-(5-(*tert*-butyl)-1*H*-pyrazol-3-yl)pyridine was added dropwise to the $ZnCl_2$ solution. After 5 min, sodium thiocyanate (0.022 g, 0.271 mmol) in 1 mL of MeOH was added dropwise. After 20 hr, volatiles were removed in vacuo. The material was dissolved in CH_2Cl_2 (~8 mL), filtered, and volatiles were removed. The solid was washed with diethyl ether (3 x 10 mL) and dried to afford white solid (0.032 g, 0.033 mmol, 50%) assigned as [($^{H}NN^{tBu}$)$_3$Zn][Zn(NCS)$_4$]. Single, X-ray quality crystals were obtained by diffusing hexanes into

a 1,2-dichloroethane solution of [($^{H}NN^{tBu}$)$_3$Zn][Zn(NCS)$_4$] at room temperature. ^{1}H NMR ($CDCl_3$, 60 MHz, 25 °C): δ = 1.42 (s, 9H, C(C*H*$_3$)$_3$), 6.64 (s, 1H, pz-C*H*), 7.53 (t, *J* = 6.6, 1H, pyr-C*H*), 7.72-8.22 (m, 3H, pyr-C*H*), 12.52 (broad, 1H, N-*H*). ^{13}C{^{1}H} NMR ($CDCl_3$, 15 MHz, 25 °C): δ = 30.07 (C(*C*H$_3$)$_3$), 31.75 (*C*(CH$_3$)$_3$), 99.84 (Ar-*C*), 121.44 (Ar-*C*), 126.04 (Ar-C), 140.80 (Ar-C), 147.39 (Ar-C), 147.89 (Ar-*C*), 148.21 (Ar-*C*), 158.97. The S=*C*=N resonance was not observed. IR (ATR): ν = 3179 (N-H), 3131 (N-H), 3070 (N-H), 2075 (NCS) 1444, 782 cm^{-1}. IR ($CDCl_3$): ν = 3421, 3177 (N-H), 3142, 3096, 3068, 2090 (NCS) cm^{-1}.

Synthesis of ($^{H}NN^{tBu}$)$_2$Zn(OAc)$_2$ (3). A 20 mL scintillation vial was charged with Zn(OAc)$_2$(H$_2$O)$_2$ (0.137 g, 0.624 mmol), 6 mL of MeOH, and a stir bar. A separate 20 mL scintillation vial was charged with 2-(5-(*tert*-butyl)-1*H*-pyrazol-3-yl)pyridine (0.250 g, 1.242 mmol) and 6 mL of MeOH. While stirring, the solution of 2-(5-(*tert*-butyl)-1*H*-pyrazol-3-yl)pyridine was added dropwise to the Zn(OAc)$_2$(H$_2$O)$_2$ solution. After 18 hr, volatiles were removed in vacuo. The resulting solid was washed with diethyl ether/hexanes (1:1, 20 mL) and hexanes (2 x 20 mL) and dried to afford white solid (0.327 g, 0.558 mmol, 90%) assigned as ($^{H}NN^{tBu}$)$_2$Zn(OAc)$_2$. Single, X-ray quality crystals were obtained by diffusing hexanes into a benzene solution of ($^{H}NN^{tBu}$)$_2$Zn(OAc)$_2$ at room temperature. ^{1}H NMR ($CDCl_3$, 700 MHz, 25 °C): 1.48 (s, 9H, C(C*H*$_3$)$_3$), 1.83 (s, 3H, O$_2$CC*H*$_3$), 6.54 (s, 1H, pz-C*H*), 7.09 (t, *J* = 6.2, 1H, pyr-C*H*), 7.56 (d, *J* = 7.7, 1H, pyr-C*H*), 7.67 (t, *J* = 7.6, 1H, pyr-C*H*), 7.88 (broad, 1H, pyr-C*H*). ^{1}H NMR ($CDCl_3$, 400 MHz, 25 °C): 1.47 (s, 9H, C(C*H*$_3$)$_3$), 1.84 (s, 3H, O$_2$CC*H*$_3$), 6.54 (s, 1H, pz-C*H*), 7.10 (t, *J* = 6.0, 1H, pyr-C*H*), 7.56 (d, *J* = 7.8, 1H, pyr-C*H*), 7.68 (t, *J* = 7.5, 1H, pyr-C*H*), 7.89 (broad, 1H, pyr-C*H*). ^{1}H NMR (CD_3OD, 400 MHz, 25 °C): 1.39 (s, 9H, C(C*H*$_3$)$_3$), 1.85 (s, 3H, O$_2$CC*H*$_3$), 6.91 (s, 1H, pz-C*H*), 7.41 (t, *J* = 5.3, 1H, pyr-C*H*), 7.95-8.03 (m, 2H, pyr-C*H*), 8.06 (broad, 1H, pyr-C*H*). ^{13}C{^{1}H} NMR ($CDCl_3$, 176 MHz, 25 °C): δ = 24.45 (*C*H$_3$), 30.49 (C(*C*H$_3$)$_3$), 31.77 (*C*(CH$_3$)$_3$), 98.83 (pz-*C*H), 119.61 (pyr-*C*H), 123.44 (pyr-*C*H), 137.74 (pyr-*C*H), 146.66 (Ar-*C*), 148.35 (pyr-*C*H), 148.35 (Ar-*C*), 157.15 (Ar-*C*), 179.22 (*C*=O). IR (ATR, neat): ν = 3106, 2961, 1605, 1574, 1515, 1405, 1302, 1152, 989, 914, 784, 746, 722, 695 cm^{-1}. IR ($CHCl_3$): no tangible absorptions. Hi-Res LCMS: Calc. for [$C_{28}H_{36}N_6O_4Zn_1$] – [HOAc + OAc] = 465.1745. Obs. = 465.1947.

Attempted Synthesis of ($^{Me}NN^{tBu}$)$_2$Zn(OAc)$_2$; Identification of ($^{Me}NN^{tBu}$)Zn(OAc)$_2$ (3'). A 20 mL scintillation vial was charged with Zn(OAc)$_2$(H$_2$O)$_2$ (0.052 g, 0.237 mmol), 4 mL of MeOH, and a stir bar. A separate 20 mL scintillation vial was charged with 2-(1-methyl-5-(*tert*-butyl)-1*H*-pyrazol-3-yl)pyridine ($^{Me}NN^{tBu}$; 0.102 g, 0.474 mmol) and 3 mL of MeOH. While stirring, the solution of 2-(1-methyl-5-(*tert*-butyl)-1*H*-pyrazol-3-yl)pyridine ($^{Me}NN^{tBu}$) was added dropwise to the Zn(OAc)$_2$(H$_2$O)$_2$ solution. The reaction was stirred 1 hr, then volatiles were removed in vacuo. The material was dissolved in 5 mL CH_2Cl_2, filtered, and volatiles removed. The solid was then washed with 2 x 20 mL hexanes and dried to afford off-white powder (0.084 g). ^{1}H NMR analysis revealed a 2:1 ratio of acetate to $^{Me}NN^{tBu}$, consistent with ($^{Me}NN^{tBu}$)Zn(OAc)$_2$. Independent synthesis of ($^{Me}NN^{tBu}$)Zn(OAc)$_2$ and comparison of the two samples spectroscopically confirmed they are identical. The yield of ($^{Me}NN^{tBu}$)Zn(OAc)$_2$ from this reaction was 89%

Authentic Synthesis of ($^{Me}NN^{tBu}$)Zn(OAc)$_2$ (3'). A 20 mL scintillation vial was charged with Zn(OAc)$_2$(H$_2$O)$_2$ (0.228 g, 1.039 mmol), 6 mL of MeOH, and a stir bar. A separate 20 mL scintillation vial was charged with 2-(1-methyl-5-(*tert*-butyl)-1*H*-pyrazol-3-yl)pyridine ($^{Me}NN^{tBu}$; 0.225 g, 1.045 mmol) and 6 mL of MeOH. While stirring, the solution of with 2-(1-methyl-5-(*tert*-butyl)-1*H*-pyrazol-3-yl)pyridine ($^{Me}NN^{tBu}$) was added dropwise to the Zn(OAc)$_2$(H$_2$O)$_2$ solution. The reaction was stirred 1 hr, then volatiles were removed in vacuo. The material was dissolved in 10 mL CH_2Cl_2, filtered, and volatiles removed. The solid was then washed with 2 x 20 mL hexanes and dried to afford off-white powder (0.366 g, 0.918 mmol, 88%) identified as ($^{Me}NN^{tBu}$)Zn(OAc)$_2$. Single, X-ray quality crystals were obtained by diffusing Et_2O into an *o*-$C_6H_4Cl_2$ solution of ($^{Me}NN^{tBu}$)Zn(OAc)$_2$ at room temperature. ^{1}H NMR ($CDCl_3$, 60 MHz, 25 °C): δ = 1.39 (s, 9H,

$C(CH_3)_3$), 2.03 (s, 6H, O_2CCH_3), 4.18 (s, 3H, N-CH_3), 6.54 (s, 1H, pz-C*H*), 7.248.11 (m, 3H, pyr-C*H*), 8.77 (d, *J* = 5.1, 1H, pyr-C*H*). $^{13}C\{^1H\}$ NMR ($CDCl_3$, 15 MHz, 25 °C): δ = 21.91 (O_2CCH_3), 29.32 ($C(CH_3)_3$), 31.74 ($C(CH_3)_3$), 39.96 (N-CH_3), 101.42 (Ar-*C*H), 120.01 (Ar-*C*H), 124.76 (Ar-*C*H), 139.99 (Ar-*C*H), 145.03 (Ar-*C*), 147.36 (Ar-*C*), 149.53 (Ar-*C*H), 156.33 (Ar-*C*), 180.87 (O_2CCH_3). IR (ATR, neat): ν = 2976, 1609, 1582, 1558, 1503, 1478, 1428 (broad), 1368, 1334, 1308, 1290, 1250, 1225, 1170, 1144, 1056, 1022, 1006, 980, 931, 816, 801, 758, 742, 686, 678 cm^{-1}. MS (monoisotopic mass) of $[(^{Me}NN^{tBu})Zn(OAc)_2]_2$ - OAc ($C_{32}H_{43}N_6O_6Zn_2$): Calc. = 735.1827 Da; Obs. = 735.28 Da. MS (monoisotopic mass) of $[C_{32}H_{43}N_6O_6Zn_2]^+$ - $Zn(OAc)_2$: Calc. = 553.2269 Da; Obs. = 553.32 Da.

Synthesis of $(^{H}NN^{tBu})_2Zn(O_2CFc)_2$ (4). A 20 mL scintillation vial was charged with KO_2CFc (0.150 g, 0.559 mmol), 6 mL of MeOH, and a stir bar. A separate 20 mL scintillation vial was charged with 2-(5-(*tert*-butyl)-1*H*-pyrazol-3-yl)pyridine (0.115 g, 0.571 mmol) and 3 mL of MeOH. A third 20 mL scintillation vial was charged with $ZnCl_2$ (0.037 g, 0.271) in 1 mL methanol. While stirring, the solution of 2-(5-(*tert*-butyl)-1*H*-pyrazol-3-yl)pyridine was added dropwise to the $ZnCl_2$ solution and the mixture stirred for ~5 min. The solution of KO_2CFc was then added dropwise and the mixture was stirred for 22 hr. Volatiles were removed and the mixture redissolved in 3 mL dichloromethane and filtered. Volatiles were removed and the resulting solid washed with 3 x 20 mL hexanes and dried to afford orange solid (0.231 g, 0.248 mmol, 91%) assigned as $(^{H}NN^{tBu})_2Zn(O_2CFc)_2$. Single, X-ray quality crystals were obtained by the slow diffusion of hexanes into a toluene solution of $(^{H}NN^{tBu})_2Zn(O_2CFc)_2$ containing trace MeOH at room temperature. ^1H NMR ($CDCl_3$, 60 MHz, 25 °C): δ = 1.46 (s, 9H, $C(CH_3)_3$), 4.04 (s, 5H, Cp-C*H*), 4.21 (s, 2H, Cp-C*H*), 4.70 (s, 2H, Cp-C*H*), 6.58 (s, 1H, pz-C*H*), 7.16-7.47 (m, 1H, pyr-C*H*), 7.59-7.95 (m, 2H, pyr-C*H*), 8.38 (s, 1H, pyr-C*H*), 12.10 (broad, 1H, N*H*). $^{13}C\{^1H\}$ NMR ($CDCl_3$, 15 MHz, 25 °C): δ = 30.55 ($C(CH_3)_3$), 31.95 ($C(CH_3)_3$), 69.46 (Cp-*C*H), 70.86 (Cp-C*H*), 98.78 (pz-*C*H), 102.48 (Ar-*C*), 108.06 (Ar-*C*), 119.67 (pyr-*C*H), 123.35 (pyr-*C*H), 138.02 (pyr-*C*H), 148.28 (pyr-*C*H), 157.71 (Ar-*C*), 177.73 (*C*=O). IR (ATR, neat): ν = 2961, 1605, 1466, 1385, 1356, 1346, 1305, 1106, 1001, 988, 776, 720, 694 cm^{-1}. IR ($CDCl_3$): ν = 2969, 1606, 1574, 1536, 1470, 1389, 1357, 1265 cm^{-1}. IR (CH_2Cl_2): ν = 2968, 1605, 1573, 1535, 1468, 1387, 1357 cm^{-1}. UV Vis (THF, ambient temperature): λ_{max} = 434 nm; ε = 368 +/- 4 $M^{-1}cm^{-1}$. MS (monoisotopic mass) of $C_{46}H_{48}N_6O_4Fe_2Zn_1$ – ($FcCO_2^-$): Calc. = 695.1775 Da; Obs. = 695.27 Da. MS (monoisotopic mass) of $C_{46}H_{48}N_6O_4Fe_2Zn_1$ – ($FcCO_2^-$ + $FcCO_2H$): Calc. = 465.1745 Da; Obs. = 465.20 Da.

Synthesis of $(^{Me}NN^{tBu})Zn(O_2CFc)_2$ (4'). A 20 mL scintillation vial was charged with zinc chloride (0.055 g, 0.404 mmol) and 2 mL CH_3OH. While stirring, a solution of 2-(1-methyl-5-(*tert*-butyl)-1*H*-pyrazol-3-yl)pyridine ($^{Me}NN^{tBu}$; 0.087 g, 0.404 mmol) in CH_3OH (6 mL) was added dropwise. To the stirring mixture, solid potassium ferrocenecarboxylate (0.219 g, 0.817 mmol) was added portion wise. The reaction was stirred for 18 hr, then volatiles were removed in vacuo. The material was extracted with 10 mL CH_2Cl_2, filtered over Celite, and dried. The resulting solid was washed with 3 x 20 mL hexanes and dried to afford light orange solid (0.219 g, 0.296 mmol, 73%) assigned as $(^{Me}NN^{tBu})Zn(O_2CFc)_2$. Single, X-ray quality crystals were obtained by diffusing Et_2O into a C_6H_6 solution of $(^{Me}NN^{tBu})Zn(O_2CFc)_2$ at room temperature, on the benchtop. Data refinement revealed the hydrate, $(^{Me}NN^{tBu})Zn(O_2CFc)_2(H_2O)$. ^1H NMR ($CDCl_3$, 60 MHz, 25 °C): 1.43 (s, 9H, $C(CH_3)_3$), 4.20 (s, 5H, Cp-C*H*), 4.13-4.35 (overlapping, 5H, Cp-C*H* and N-CH_3), 4.82 (m, 2H, Cp-C*H*), 6.60 (s, 1H, pz-C*H*), 7.35-7.55 (m, 1H, pyr-C*H*), 7.63-7.96 (m, 2H, pyr-C*H* x 2), 8.88 (m, 1H, pyr-C*H*). $^{13}C\{^1H\}$ NMR ($CDCl_3$, 15 MHz, 25 °C): δ = 29.49 ($C(CH_3)_3$), 31.74 ($C(CH_3)_3$), 40.19 (N-CH_3), 69.78 (Cp-*C*H), 70.48 (Cp-C*H*), 70.94 (Cp-*C*H), 74.43 (Cp-*C*), 101.73 (pz-*C*H), 120.09 (pyr-*C*H), 124.36 (pyr-*C*H), 139.52 (pyr-*C*H), 149.68 (pyr-*C*H), 180.74 (*C*=O). Three sp^2 carbons (Ar-C) of the $^{Me}NN^{tBu}$ ligand are poorly resolved and not reported. IR (ATR, neat): ν = 2967, 1610, 1574, 1549 (with others overlapping), 1469, 1385, 1358,

1344, 1287, 1251, 1224, 1183, 1157, 1105, 1053, 1021, 1001, 979, 922, 813, 785, 743, 687 cm^{-1}. IR (CH_2Cl_2; inert atmosphere): ν = 2976, 1612, 1590, 1575, 1480, 1391, 1360, 1347, 1224, 1205, 1193, 1183, 1157, 1140, 1106, 1092, 1053, 1031, 1024, 1002, 980, 947, 922, 514, 488 cm^{-1}. UV Vis (THF, ambient temperature): λ_{max} = 439 nm; ε = 434 +/- 3 $M^{-1}cm^{-1}$ $M^{-1}cm^{-1}$. MS (monoisotopic mass) of [$C_{35}H_{35}N_3O_4Zn_1Fe_2$] – e^-: Calc. = 737.0618 Da; Obs. = 737.30 Da. MS (monoisotopic mass) of [$C_{35}H_{35}N_3O_4Zn_1Fe_2$] – $(O_2CFc)^{1-}$ + ($^{Me}NN^{tBu}$): Calc. = 723.2088 Da; Obs. = 723.41 Da. MS (monoisotopic mass) of [$C_{37}H_{43}N_6O_2Zn_1Fe_1$]$^{1+}$ + $Zn(O_2CFc)_2$: Calc. = 1245.1284 Da; Obs. = 1245.36 Da.

Synthesis of ($^{H}NN^{tBu}$)$_2$Zn(OTf)$_2$ (5) from ($^{H}NN^{tBu}$)$_2$Zn(O_2CFc)$_2$ (4). Inside a glovebox, a 20 mL scintillation vial was charged with ($^{H}NN^{tBu}$)$_2$Zn(O_2CFc)$_2$ (0.020 g, 0.022 mmol) and 2 mL dichloromethane. While stirring, AgOTf (0.011 g, 0.043 mmol) was added resulting in a rapid color change from orange to dark green. The solution was stirred 17 hr and the crude mixture investigated by ^{1}H NMR spectroscopy, infrared spectroscopy, and electronic absorption spectroscopy.

Oxidation of ($^{Me}NN^{tBu}$)Zn(O_2CFc)$_2$ (4') with AgOTf. In analogy to the oxidation of ($^{H}NN^{tBu}$)$_2$Zn(O_2CFc)$_2$ with AgOTf to produce ($^{H}NN^{tBu}$)$_2$Zn(OTf)$_2$, a control reaction was performed with ($^{Me}NN^{tBu}$)Zn(O_2CFc)$_2$. Inside of a glovebox, a 20 mL scintillation vial was charged with ($^{Me}NN^{tBu}$)Zn(O_2CFc)$_2$ (0.046 g, 0.062 mmol), 2 mL CH_2Cl_2, and a stir bar. While stirring, solid silver trifluoromethanesulfonate (0.032 g, 0.125 mmol) was added. A gradual color change from light orange to teal/green occurred over the course of 5 min. After 60 min, the mixture was filtered, an aliquot was removed, further diluted with CH_2Cl_2, and analyzed via electronic absorption spectroscopy. The spectrum revealed a strong absorption at 633 nm, consistent with oxidation for ferrocenecarboxylate to ferroceniumcarboxylate. From the remaining solution, a portion was utilized for solution IR spectroscopy. The remaining solution was dried in vacuo to afford green solid. This crude material was directly analyzed by NMR spectroscopy. The material is paramagnetic; ^{1}H NMR resonances attributable to $^{Me}NN^{tBu}$ and $O_2C(C_5H_4)Fe(C_5H_5)$ are not observed. An ^{19}F NMR resonance was not observed. ^{1}H NMR ($CDCl_3$, 60 MHz, 25 °C): 1.40 (78), 3.78 (220), 6.44 (partially obscured by $CDCl_3$ resonance), 7.53 (partially obscured by $CDCl_3$ resonance), 15.97 (700). IR (CH_2Cl_2): ν = 3110, 3062, 2975, 1614, 1574, 1478, 1393, 1363, 1159, 1107, 1055, 1031, 979, 947, 921, 856, 825, 813, 638, 573, 517, 488 cm^{-1}.

Independent Synthesis of ($^{H}NN^{tBu}$)$_2$Zn(OTf)$_2$ (5). A 20 mL scintillation vial was charged with Zn(OTf)$_2$ (0.228 g, 0.627 mmol), 6 mL of MeOH, and a stir bar. A separate 20 mL scintillation vial was charged with 2-(5-(*tert*-butyl)-1*H*-pyrazol-3-yl)pyridine (0.251 g, 1.247 mmol) and 6 mL of MeOH. While stirring, the solution of 2-(5-(*tert*-butyl)-1*H*-pyrazol-3-yl)pyridine was added dropwise to the Zn(OTf)$_2$ solution. After 21 hr, volatiles were removed in vacuo. The resulting solid was washed with diethyl ether/hexanes (1:1, 20 mL) and hexanes (2 x 20 mL) and dried to afford white solid (0.435 g, 0.568 mmol, 90%) assigned as ($^{H}NN^{tBu}$)$_2$Zn(OTf)$_2$. Single, X-ray quality crystals were obtained by diffusing hexanes into an ethyl acetate solution of ($^{H}NN^{tBu}$)$_2$Zn(OTf)$_2$ at room temperature. ^{1}H NMR ($CDCl_3$, 700 MHz, 25 °C): 1.49 (s, 9H, C(C*H*$_3$)$_3$), 6.68 (s, 1H, pz-C*H*), 7.35 (t, *J* = 7.0, 1H, pyr-C*H*), 7.78 (d, *J* = 7.7, 1H, pyr-C*H*), 7.86 (d, *J* = 4.7, 1H, pyr-C*H*), 7.94 (t, *J* = 7.6, 1H, pyr-C*H*), 13.04 (s, 1H, N-*H*). Samples prepared on the benchtop contained an additional resonance identified as H_2O that was unable to be removed through recrystallization, washing, or dynamic vacuum. Samples prepared using glovebox protocol in CH_2Cl_2 did not contain this resonance. ^{1}H NMR (crude analysis, CH_3OH, 400 MHz, 25 °C): 1.34 (s, 9H, C(C*H*$_3$)$_3$), 6.98 (s, 1H, pz-C*H*), 7.51 (broad, 1H, pyr-C*H*), 7.78 (broad, 3H, pyr-C*H*). ^{19}F NMR ($CDCl_3$, 56 MHz, 25 °C): δ = -81.45 (internally referenced to C_6F_6). $^{13}C\{^{1}H\}$ NMR ($CDCl_3$, 176 MHz, 25 °C): δ = 30.04 (C(CH_3)$_3$), 31.90 (*C*(CH_3)$_3$), 99.65 (pz-*C*H), 121.04 (pyr-*C*H), 125.29 (pyr-*C*H), 140.45 (pyr-*C*H), 147.32 (Ar-*C*), 147.42 (Ar-*C*), 147.59 (pyr-*C*H), 159.24 (Ar-*C*). The *C*F_3

resonance was not observed. IR (ATR, neat): ν = 3192 (N-H), 2977, 1612, 1576, 1474, 1448, 1282, 1222, 1172, 1028, 1003, 837, 792 cm^{-1}. IR ($CHCl_3$): 3202 (N-H) cm^{-1}. Hi-Res LCMS: Calc. for [$C_{26}H_{30}F_6N_6O_6S_2Zn_1$] – OTf = 615.1344. Obs. = 615.1361.

Attempted Synthesis of ($^{Me}NN^{tBu}$)Zn(OTf)$_2$ (5'; benchtop). In analogy to ($^{H}NN^{tBu}$)$_2$Zn(OTf)$_2$, a benchtop synthesis of ($^{Me}NN^{tBu}$)Zn(OTf)$_2$ was attempted. A 20 mL scintillation vial was charged with zinc trifluoromethanesulfonate (0.164 g, 0.451 mmol), 6 mL CH_2Cl_2, and a stir bar. While stirring, a solution of 2-(1-methyl-5-(*tert*-butyl)-1*H*-pyrazol-3-yl)pyridine ($^{Me}NN^{tBu}$; 0.097 g, 0.451 mmol) in CH_2Cl_2 (2 mL) was added dropwise. The mixture was stirred 18 hr, filtered over Celite, and dried. The resulting material was washed with 3 x 20 mL hexanes to afford off-white powder (0.236 g). The dried powder was highly hygroscopic, irreversibly becoming an oily semi-solid within minutes of exposure to air. The solubility in $CDCl_3$ is poor, however, ^{1}H NMR resonances observed of the material differ from those of unbound $^{Me}NN^{tBu}$ ligand. Infrared spectroscopy (ATR) reveals an intense, broad absorption consistent with $\nu_{(O-H)}$. Mass spectrometry revealed isotope patterns consistent with [($^{Me}NN^{tBu}$)$_3$Zn]$^{2+}$ and [($^{Me}NN^{tBu}$)$_2$Zn(OTf)]$^{+}$. Single, X-ray quality crystals obtained by slow evaporation of a $CDCl_3$ solution of the material afforded data consistent with [($^{Me}NN^{tBu}$)Zn(H_2O)$_n$][OTf]$_2$ (disordered; n = 3, 4). Single, X-ray quality crystals obtained by diffusing hexanes into an acetone solution of the material afforded data consistent with [($^{Me}NN^{tBu}$)$_2$Zn(H_2O)$_2$][OTf]. These data all suggest that a benchtop synthesis of ($^{Me}NN^{tBu}$)Zn(OTf)$_2$ in analogy to ($^{H}NN^{tBu}$)$_2$Zn(OTf)$_2$ is not possible. ^{1}H NMR ($CDCl_3$, 60 MHz, 25 °C): 1.46 (s, 9H, C(C*H*$_3$)$_3$), 4.02 (s, 3H, N-C*H*$_3$), 6.69 (s, 1H pz-C*H*), 7.31-7.56 (m, 1H, pyr-C*H*), 7.75-8.04 (m, 2H, pyr-C*H*), 8.15-8.38 (m, 1H, pyr-C*H*). The spectrum contains an additional broad resonance at 3.45 ppm attributed to coordinated water. IR (ATR, neat): ν = 3390 (O-H), 1613, 1505, 1461, 1367, 1276, 1237, 1223, 1166, 1026, 979, 785, 762, 744, 688 cm^{-1}. MS (monoisotopic mass) of [$C_{39}H_{51}N_9Zn_1$]$^{2+}$: Calc. = 354.6774 Da; Obs. = 354.82 Da. MS (monoisotopic mass) of [$C_{26}H_{34}N_6Zn_1$(OTf)]$^{+}$: Calc. = 643.1657 Da; Obs. = 643.29 Da.

Attempted Synthesis of ($^{Me}NN^{tBu}$)Zn(OTf)$_2$; isolation of ($^{Me}NN^{tBu}$)$_2$Zn(OTf)$_2$ (5'; inert atmosphere). In a glovebox, a 20 mL scintillation vial was charged with 2-(1-methyl-5-(*tert*-butyl)-1*H*-pyrazol-3-yl)pyridine ($^{Me}NN^{tBu}$; 0.172 g, 0.799 mmol), CH_2Cl_2 (4 mL), and a stir bar. While stirring, solid zinc trifluoromethanesulfonate (0.290 g, 0.798 mmol) was added. The reaction was stirred 16 hr, filtered, and volatiles removed in vacuo. The solid was washed with hexanes (20 mL) and Et_2O (20 mL) to afford white powder (0.282 g). The spectroscopy of this material mirrored that of ($^{Me}NN^{tBu}$)$_2$Zn(OTf)$_2$. Yield of ($^{Me}NN^{tBu}$)$_2$Zn(OTf)$_2$: 0.355 mmol, 89%.

Synthesis of ($^{Me}NN^{tBu}$)$_2$Zn(OTf)$_2$ (5'; inert atmosphere). In a glovebox, a 20 mL scintillation vial was charged with 2-(1-methyl-5-(*tert*-butyl)-1*H*-pyrazol-3-yl)pyridine ($^{Me}NN^{tBu}$; 0.105 g, 0.488 mmol), CH_2Cl_2 (4 mL), and a stir bar. While stirring, solid zinc trifluoromethanesulfonate (0.089 g, 0.245 mmol) was added. The reaction was stirred 16 hr, filtered, and volatiles removed in vacuo. The solid was washed with hexanes (20 mL) and Et_2O (20 mL) to afford white powder (0.173 g, 0.218 mmol, 89%) assigned as ($^{Me}NN^{tBu}$)$_2$Zn(OTf)$_2$. Single, X-ray quality crystals were obtained by diffusing Et_2O into a *o*-$C_6H_4Cl_2$ solution of ($^{Me}NN^{tBu}$)$_2$Zn(OTf)$_2$ at room temperature. ^{1}H NMR ($CDCl_3$, 60 MHz, 25 °C): 1.44 (s, 9H, C(C*H*$_3$)$_3$), 3.98 (s, 3H, N-C*H*$_3$), 6.72 (s, 1H pz-C*H*), 7.36-7.58 (m, 1H, pyr-C*H*), 7.81-8.05 (m, 2H, pyr-C*H*), 8.20-8.37 (m, 1H, pyr-C*H*). ^{19}F NMR ($CDCl_3$, 56 MHz, 25 °C): δ = -81.46 (internally referenced to C_6F_6). IR (CH_2Cl_2): ν = 1613, 1574, 1548, 1505, 1478, 1462, 1436, 1384, 1371, 1366, 1057, 1031, 979, 638 cm^{-1}. IR (KBr): ν = 3141, 3091, 2976, 1614, 1574, 1549, 1506, 1479, 1463, 1438, 1366, 1310, 1235, 1163, 1107, 1058, 1029, 978, 935, 836, 788, 755, 744, 690, 637, 597, 571, 517 cm^{-1}.

Control Reaction between $(^{H}NN^{tBu})_2Zn(O_2CFc)_2$ (4) and $[Bu_4N][OTf]$. Inside a glovebox, a 20 mL scintillation vial charged with $(^{H}NN^{tBu})_2Zn(O_2CFc)_2$ (0.019 g, 0.020 mmol) and 2 mL dichloromethane. While stirring, tetrabutylammonium triflate (0.017 g, 0.043 mmol) was added. The mixture was stirred for 17 hr and no color change was observed. The crude mixture was investigated by ^{1}H NMR spectroscopy and infrared spectroscopy. No reaction was observed.

Control Reaction between $(^{Me}NN^{tBu})Zn(O_2CFc)_2$ (4') and $[Bu_4N][OTf]$. Inside a glovebox, a 20 mL scintillation vial charged with $(^{Me}NN^{tBu})Zn(O_2CFc)_2$ (0.017 g, 0.023 mmol), tetrabutylammonium triflate (0.018 g, 0.046 mmol), and 0.8 mL $CDCl_3$ was added. The mixture was stirred for 15 min. then analyzed via ^{1}H NMR spectroscopy. No reaction was observed.

Control Reaction between $(^{H}NN^{tBu})_2Zn(OTf)_2$ (5) and $[Bu_4N][OAc]$. Inside a glovebox, a 20 mL scintillation vial charged with $(^{H}NN^{tBu})_2Zn(OTf)_2$ (0.027 g, 0.035 mmol), 1.2 mL $CDCl_3$, and a stir bar. The compound, $(^{H}NN^{tBu})_2Zn(OTf)_2$, has minimal solubility in $CDCl_3$. While the slurry stirred, tetrabutylammonium acetate (0.021 g, 0.070 mmol) was added as a solid. All materials immediately dissolved upon addition of tetrabutylammonium acetate. The reaction was stirred for 16 hr, then the crude $CDCl_3$ solution was analyzed via ^{1}H NMR spectroscopy and infrared spectroscopy (solution, $CDCl_3$). ^{1}H NMR spectroscopy confirmed a reaction had occurred. Infrared spectroscopy revealed absorptions consistent with $(^{H}NN^{tBu})_2Zn(OAc)_2$ as the major product with minor unreacted $(^{H}NN^{tBu})_2Zn(OTf)_2$.

Synthesis of $(^{H}NN^{tBu})_2Zn(O_2CAr)_2$ (6-X; Ar = *p*-$C_6H_4OCH_3$, C_6H_5, *p*-C_6H_4Br). A 20 mL scintillation vial was charged with $[Zn(O_2CAr)_2]_n$ (Ar = *p*-$C_6H_4OCH_3$: 0.205 g, 0.608 mmol; Ar = C_6H_5: 0.187 g, 0.608 mmol; Ar = *p*-C_6H_4Br: 0.236 g, 0.611 mmol), 12 mL of MeOH, and a stir bar. A separate 20 mL scintillation vial was charged with 2-(5-(*tert*-butyl)-1*H*-pyrazol-3-yl)pyridine (0.250 g, 1.242 mmol) and 6 mL of MeOH. While stirring, the solution of 2-(5-(*tert*-butyl)-1*H*-pyrazol-3-yl)pyridine was added dropwise over 10 min to the $[Zn(O_2CAr)_2]_n$ slurry. After 18 hr, the mixture was filtered and volatiles were removed. The material was dissolved in dichloromethane, transferred to a scintillation vial, and dried. The resulting solid was washed with 3 x 20 mL hexanes and dried to afford white solid assigned as $(^{H}NN^{tBu})_2Zn(O_2CAr)_2$. **For *p*-$C_6H_4OCH_3$**: Yield = 0.404 g, 0.521 mmol, 86%. Single, X-ray quality crystals were obtained by the slow diffusion of hexanes into a toluene solution of $(^{H}NN^{tBu})_2Zn(O_2CAr)_2$ (Ar = *p*-$C_6H_4OCH_3$) at room temperature. ^{1}H NMR ($CDCl_3$, 60 MHz, 25 °C): δ = 1.51 (s, 9H, C(CH_3)$_3$), 3.74 (s, 3H, OCH_3), 6.59 (s, 1H, pz-C*H*), 6.65 (d, *J* = 8.7, 2H, Ar-C*H*), 6.90-7.21 (m, 1H, pyr-C*H*), 7.51-7.68 (m, 2H, pyr-C*H*), 7.79 (d, *J* = 8.7, 2H, Ar-C*H*), 8.03 (d, *J* = 4.4, 1H, pyr-C*H*). $^{13}C\{^{1}H\}$ NMR ($CDCl_3$, 15 MHz, 25 °C): δ = 30.56 (C(CH_3)$_3$), 31.87 (*C*(CH_3)$_3$), 55.27 (O*C*H_3), 98.74 (pz-*C*H), 112.47 (Ar-*C*H), 119.60 (Ar-*C*), 123.35 (Ar-*C*), 129.96 (Ar-*C*), 131.72 (Ar-*C*H), 137.67 (pyr-*C*H), 146.71 (Ar-*C*), 148.00 (pyr-*C*H), 148.56 (Ar-*C*), 157.50 (Ar-*C*), 161.30 (Ar-*C*), 173.42 (*C*=O). IR (ATR, neat): ν = 2963, 1603, 1380, 1248, 1168, 989, 783, 696 cm^{-1}. MS (monoisotopic mass) of $(^{H}NN^{tBu})_2Zn(O_2CAr)_2$ - $ArCO_2^-$ ($C_{32}H_{37}N_6O_3Zn_1$): Calc. = 617.2219 Da; Obs. = 617.32 Da. MS (monoisotopic mass) of $[C_{32}H_{37}N_6O_3Zn_1]^+$ - $ArCO_2H$: Calc. = 465.1745 Da; Obs. = 465.25 Da. MS (monoisotopic mass) of $[C_{32}H_{37}N_6O_3Zn_1]^+$ - OMe + H: Calc. = 587.2113 Da; Obs. = 587.28 Da. **For C_6H_5**: Yield = 0.391 g, 0.546 mmol, 90%. Single, X-ray quality crystals were obtained by the slow diffusion of hexanes into a toluene solution of $(^{H}NN^{tBu})_2Zn(O_2CAr)_2$ (Ar = C_6H_5) at room temperature. ^{1}H NMR ($CDCl_3$, 60 MHz, 25 °C): δ = 1.56 (s, 9H, C(CH_3)$_3$), 6.59 (s, 1H, pz-C*H*), 6.87-7.36 (m, 4H, Ar/pyr-C*H*), 7.46-7.68 (m, 2H, Ar/pyr-C*H*), 7.70-8.03 (m, 3H, Ar/pyr-C*H*), 11.17 (broad, 1H, N*H*). $^{13}C\{^{1}H\}$ NMR ($CDCl_3$, 15 MHz, 25 °C): δ = 30.50 (C(CH_3)$_3$), 31.85 (*C*(CH_3)$_3$), 98.68 (pz-*C*H), 119.68 (Ar-*C*H), 123.57 (Ar-*C*H), 127.33 (Ph-*C*H), 129.89 (Ph-*C*H), 130.09 (Ar-*C*H), 137.14 (Ar-*C*), 137.95 (pyr-*C*H), 146.69 (Ar-*C*), 147.80 (Ar-*C*H), 148.27 (Ar-*C*), 157.29 (Ar-*C*), 173.54 (*C*=O). IR (ATR, neat): ν = 2966, 1609, 1538, 1516, 1381, 828, 715, 672 cm^{-1}. MS (monoisotopic mass) of

($(^{H}NN^{tBu})_2Zn(O_2CPh)_2$ - $PhCO_2^-$ - $PhCO_2$ ($C_{24}H_{29}N_6Zn_1$): Calc. = 465.1745 Da; Obs. = 465.25 Da. **For *p*-C_6H_4Br**: Yield = 0.304 g, 0.348 mmol, 64%. Single, X-ray quality crystals were obtained by concentrating a dichloromethane solution of $(^{H}NN^{tBu})_2Zn(O_2CAr)_2$ (Ar = *p*-C_6H_4Br) via slow evaporation at room temperature followed by storage at 0 °C. ^{1}H NMR ($CDCl_3$, 60 MHz, 25 °C): δ = 1.55 (s, 9H, C(CH_3)$_3$), 6.61 (s, 1H, pz-C*H*), 6.89-7.10 (m, 1H, pyr-C*H*), 7.23 (d, *J* = 8.6, 2H, Ar-C*H*), 7.64 (d, *J* = 8.6, 2H, Ar-C*H*), 7.46-7.73 (m, 2H, Ar/pyr-C*H*), 7.84 (d, *J* = 4.7, 1H, pyr-C*H*). $^{13}C\{^1H\}$ NMR ($CDCl_3$, 15 MHz, 25 °C): δ = 30.48 (C(*C*H$_3$)$_3$), 31.82 (*C*(CH$_3$)$_3$), 98.69 (pz-*C*H), 119.67 (Ar-*C*H), 123.52 (Ar-*C*H), 124.60 (Ar-*C*), 130.34 (Ph-*C*H), 131.55 (Ph-*C*H), 136.38 (Ar-*C*), 137.97 (Ar-*C*H), 146.61 (Ar-*C*), 147.66 (Ar-*C*H), 148.22 (Ar-*C*), 157.43 (Ar-*C*), 172.44 (*C*=O). IR (ATR, neat): ν = 2965, 1607, 1588, 1538, 1519, 1384, 770 cm^{-1}. MS (monoisotopic mass) of $C_{38}H_{38}N_6O_4Br_2Zn_1$ – ($ArCO_2^-$): Calc. = 665.1218 Da; Obs. = 665.21 Da. MS (monoisotopic mass) of $C_{46}H_{48}N_6O_4Br_2Zn_1$ – ($ArCO_2^-$ + $ArCO_2H$): Calc. = 465.1745 Da; Obs. = 465.20 Da.

Control Reaction between $(^{H}NN^{tBu})_2Zn(O_2CAr)_2$ (6-Br) and $[Bu_4N][OTf]$. Inside a glovebox, a 20 mL scintillation vial charged with $(^{H}NN^{tBu})_2Zn(O_2CAr)_2$ (**6-Br**; 0.020 g, 0.023 mmol), tetrabutylammonium triflate (0.018 g, 0.046 mmol), and 1 mL CH_2Cl_2 was added. The mixture was stirred for 30 min. The crude mixture was then investigated by infrared spectroscopy (CH_2Cl_2). No reaction was observed.

Control Reaction between $(^{H}NN^{tBu})_2Zn(O_2CAr)_2$ (6-H) and $[Bu_4N][OTf]$. Inside a glovebox, a 20 mL scintillation vial charged with $(^{H}NN^{tBu})_2Zn(O_2CAr)_2$ (**6-H**; 0.018 g, 0.026 mmol), and 1 mL CH_2Cl_2. While stirring, tetrabutylammonium triflate (0.020 g, 0.051 mmol) was added. The mixture was stirred for 30 min. The crude mixture was then investigated by infrared spectroscopy (CH_2Cl_2). No reaction was observed.

Control Reaction between $(^{H}NN^{tBu})_2Zn(O_2CAr)_2$ (6-OMe) and $[Bu_4N][OTf]$. Inside a glovebox, a 20 mL scintillation vial charged with $(^{H}NN^{tBu})_2Zn(O_2CAr)_2$ (**6-OMe**; 0.020 g, 0.023 mmol), and 1 mL CH_2Cl_2. While stirring, tetrabutylammonium triflate (0.020 g, 0.051 mmol) was added. The mixture was stirred for 30 min. The crude mixture was then investigated by infrared spectroscopy (CH_2Cl_2). No reaction was observed.

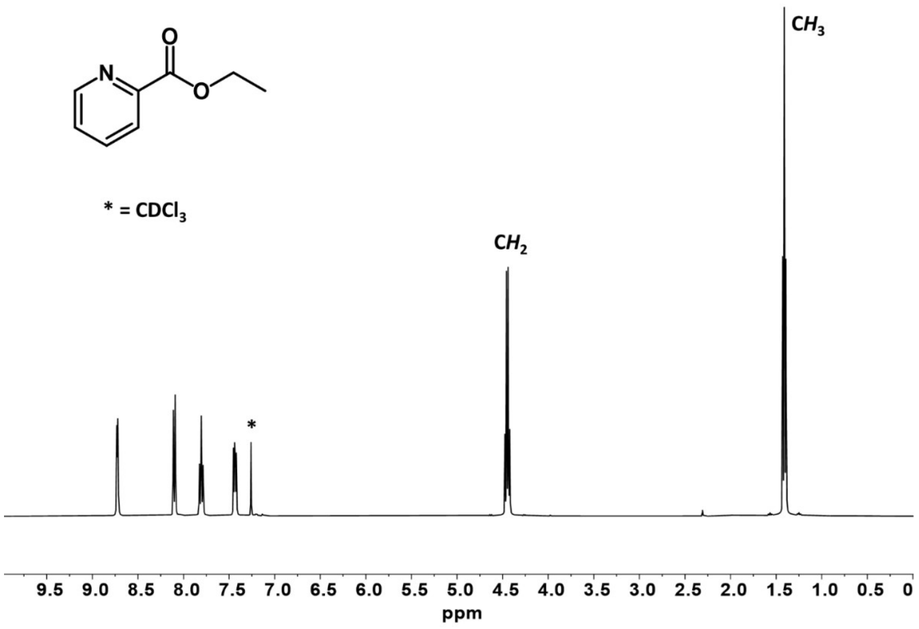

Figure S1 ^{1}H NMR spectrum ($CDCl_3$, 25 °C, 400 MHz) of 2-ethylpicolinate.

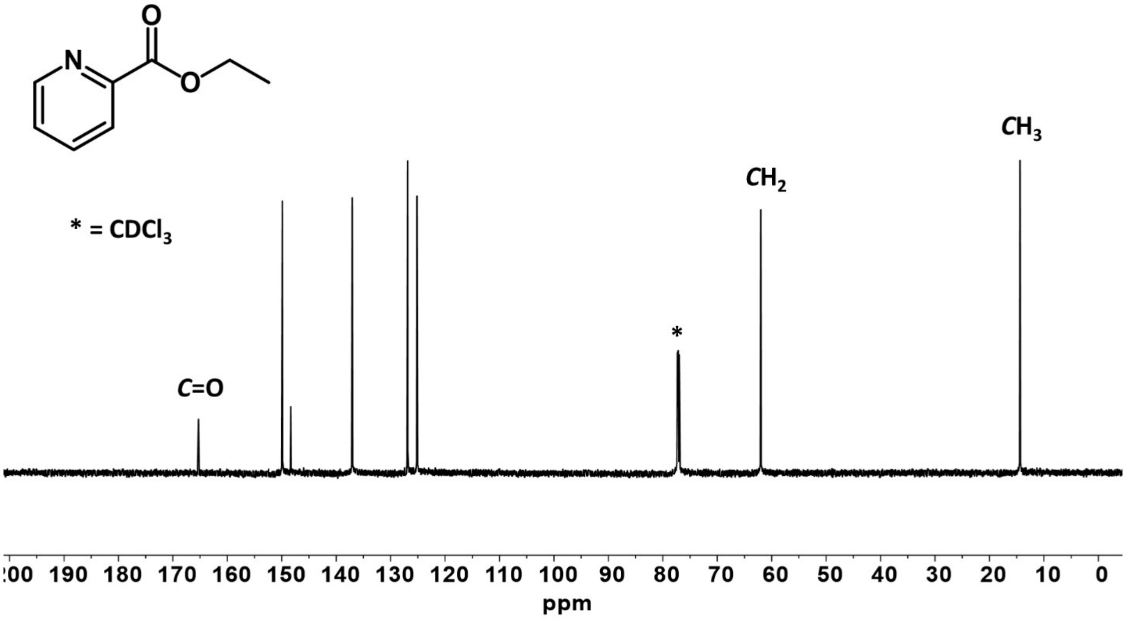

Figure S2 $^{13}C\{^{1}H\}$ NMR spectrum ($CDCl_3$, 25 °C, 176 MHz) of 2-ethylpicolinate.

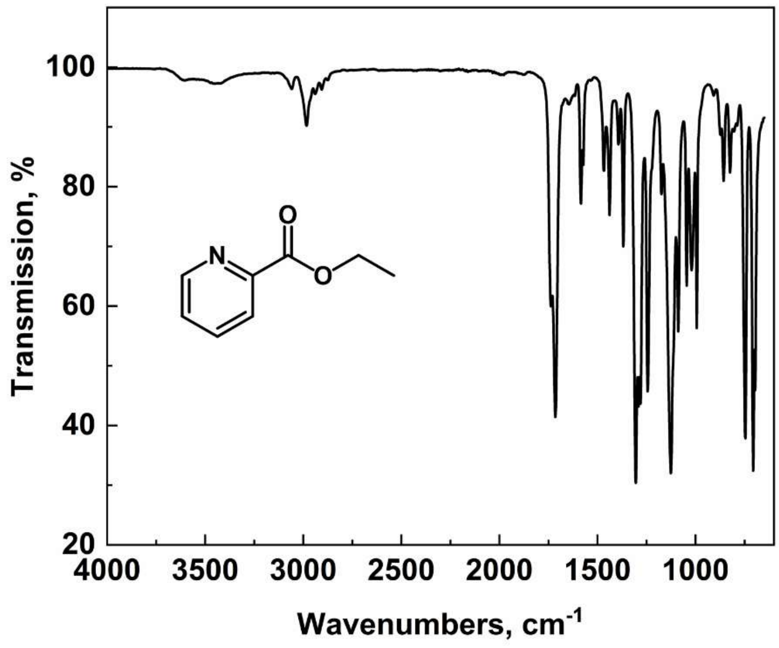

Figure S3 Infrared spectrum (ATR, ambient temperature) of 2-ethylpicolinate.

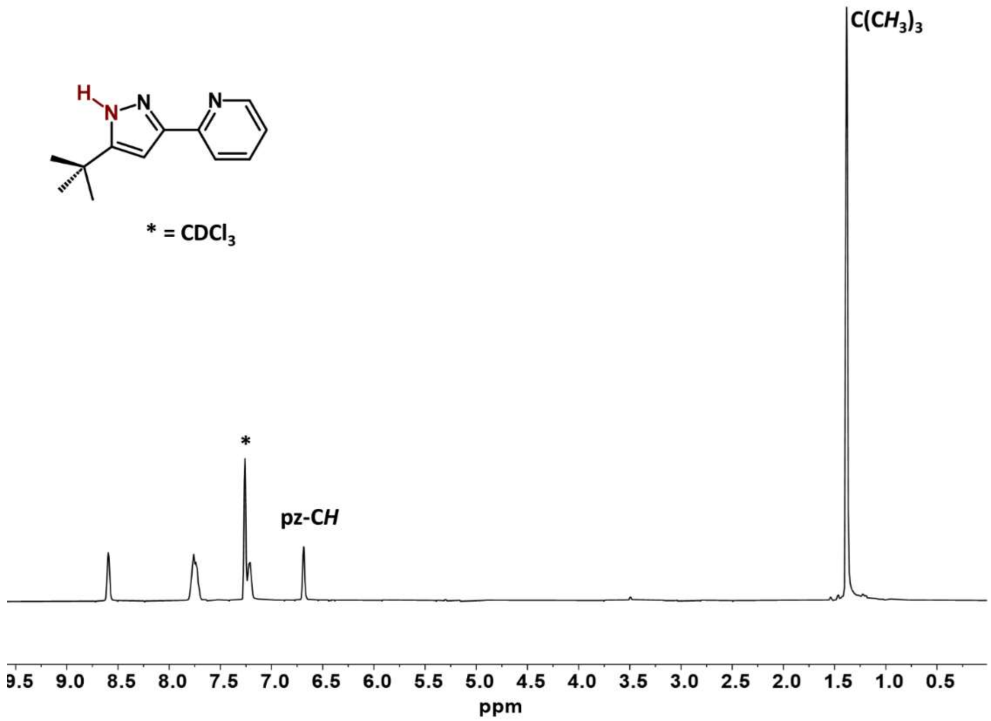

Figure S4 ^{1}H NMR spectrum ($CDCl_3$, 25 ^{o}C, 400 MHz) of $^{H}NN^{tBu}$.

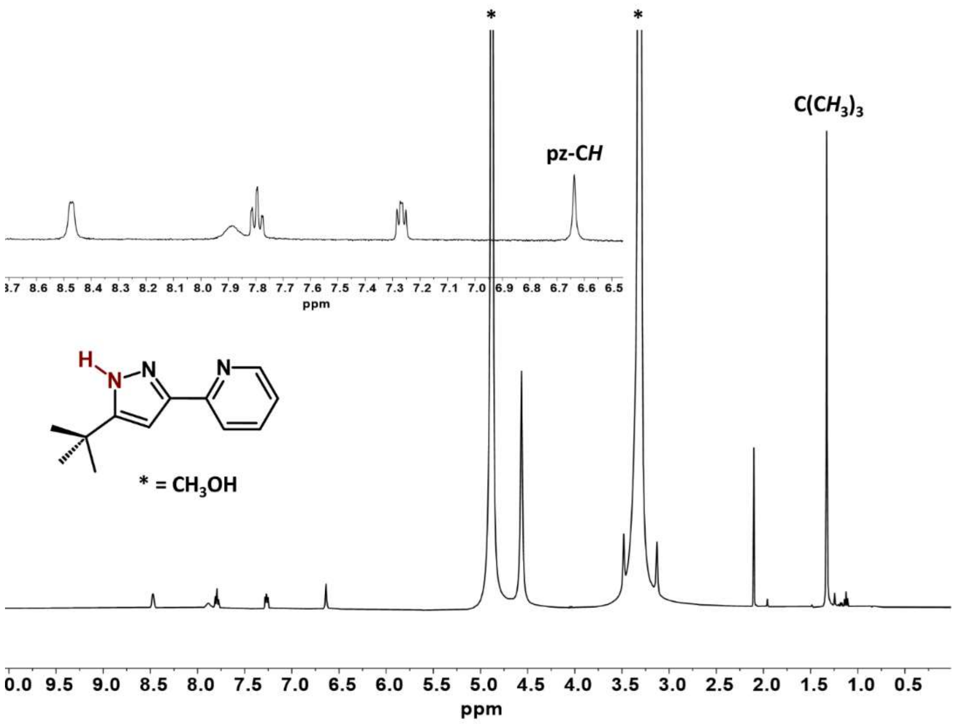

Figure S5 ^{1}H NMR spectrum (CH_3OH, 25 °C, 400 MHz) of $^{H}NN^{tBu}$.

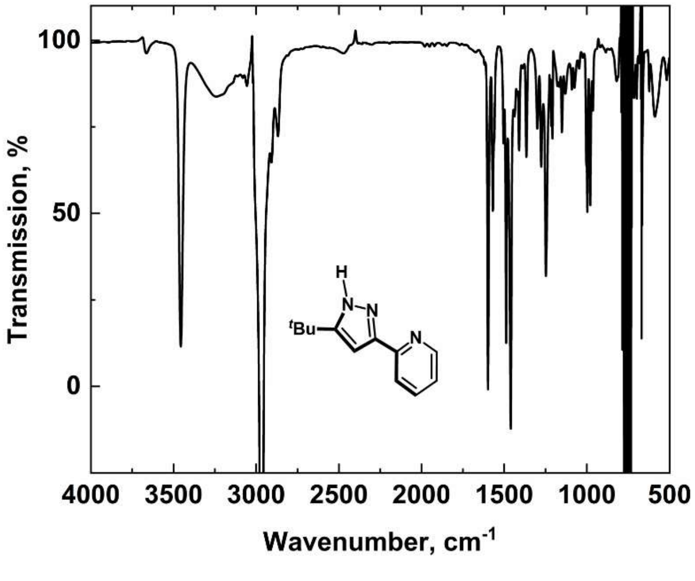

Figure S6 Infrared spectrum ($CHCl_3$, 25 °C) of $^{H}NN^{tBu}$.

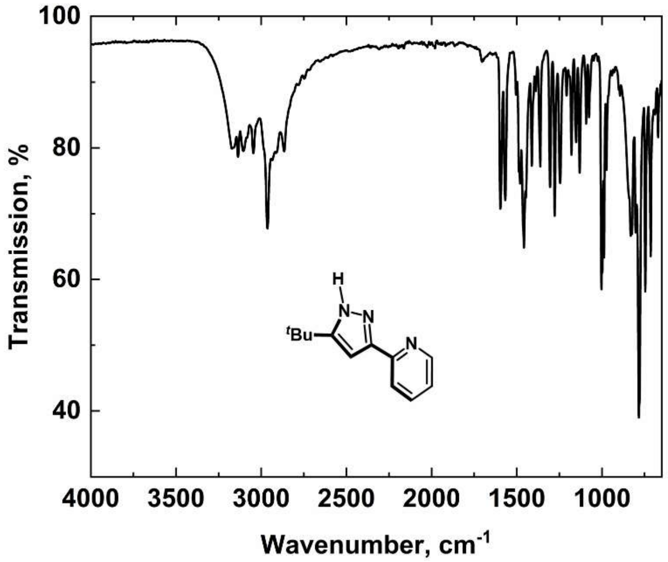

Figure S7 Infrared spectrum (ATR, neat, 25 °C) of $^{H}NN^{tBu}$.

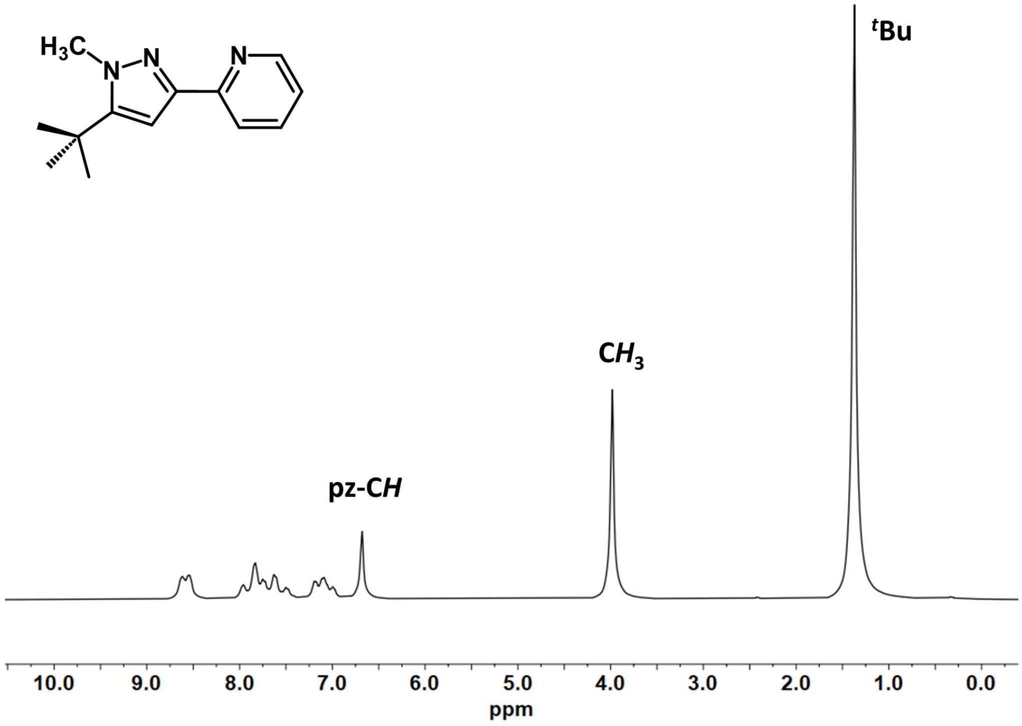

Figure S8 ^{1}H NMR spectrum ($CDCl_3$, 60 MHz, 25 °C) of 2-(1-methyl-5-(*tert*-butyl)-1*H*-pyrazol-3-yl)pyridine ($^{Me}NN^{tBu}$).

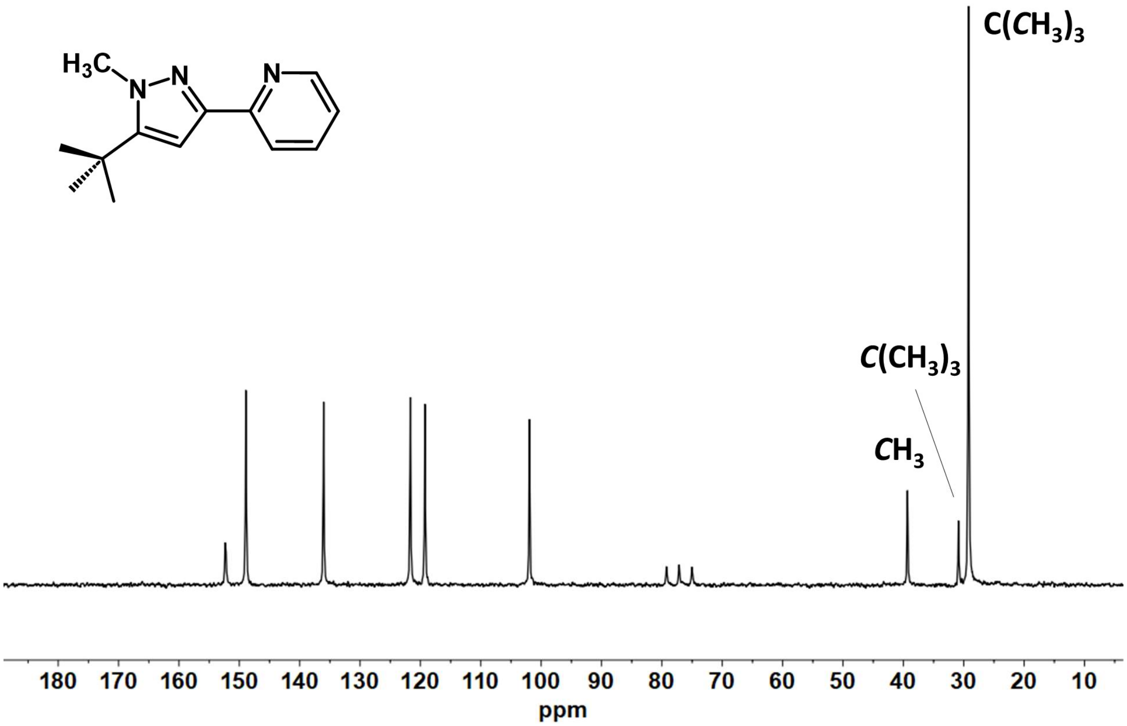

Figure S9 $^{13}C\{^{1}H\}$ NMR spectrum ($CDCl_3$, 15 MHz, 25 °C) of 2-(1-methyl-5-(*tert*-butyl)-1*H*-pyrazol-3-yl)pyridine ($^{Me}NN^{tBu}$).

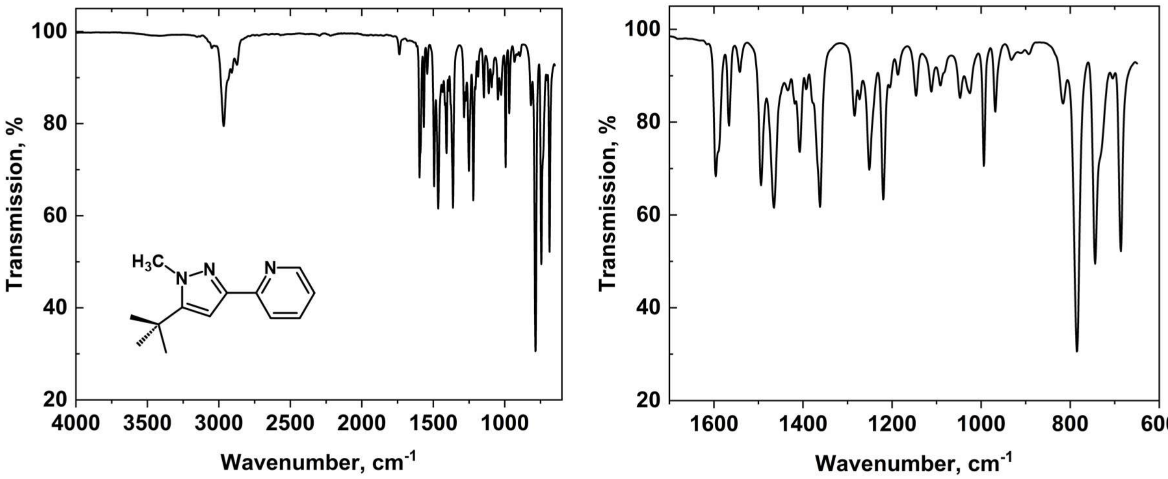

Figure S10 Infrared spectrum (ATR, neat, 25 °C) of 2-(1-methyl-5-(*tert*-butyl)-1*H*-pyrazol-3-yl)pyridine ($^{Me}NN^{tBu}$). Left: full spectrum; Right: low energy region.

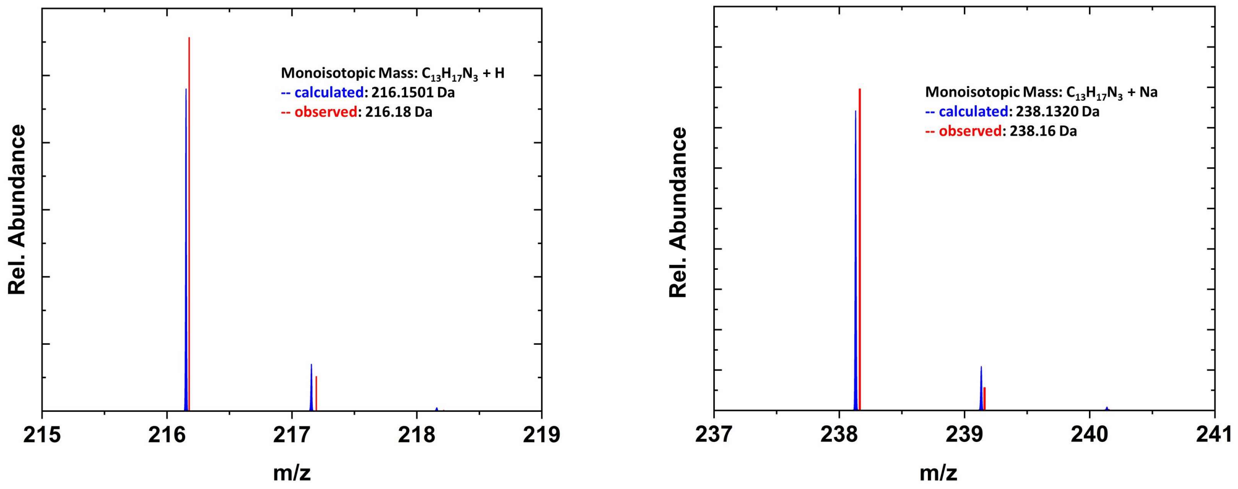

Figure S11 Mass spectrum of 2-(1-methyl-5-(*tert*-butyl)-1*H*-pyrazol-3-yl)pyridine ($^{Me}NN^{tBu}$). Left: M+H; Right: M+Na.

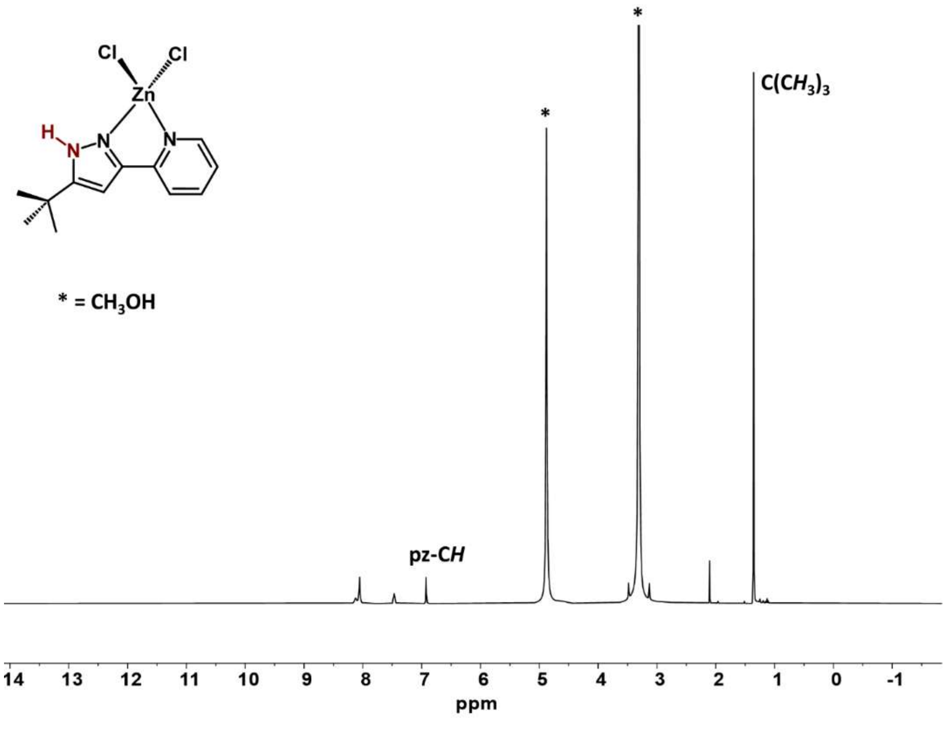

Figure S12 Crude ^{1}H NMR spectrum (CH_3OH, 25 °C, 400 MHz) of the reaction between $ZnCl_2$ and $^{H}NN^{tBu}$.

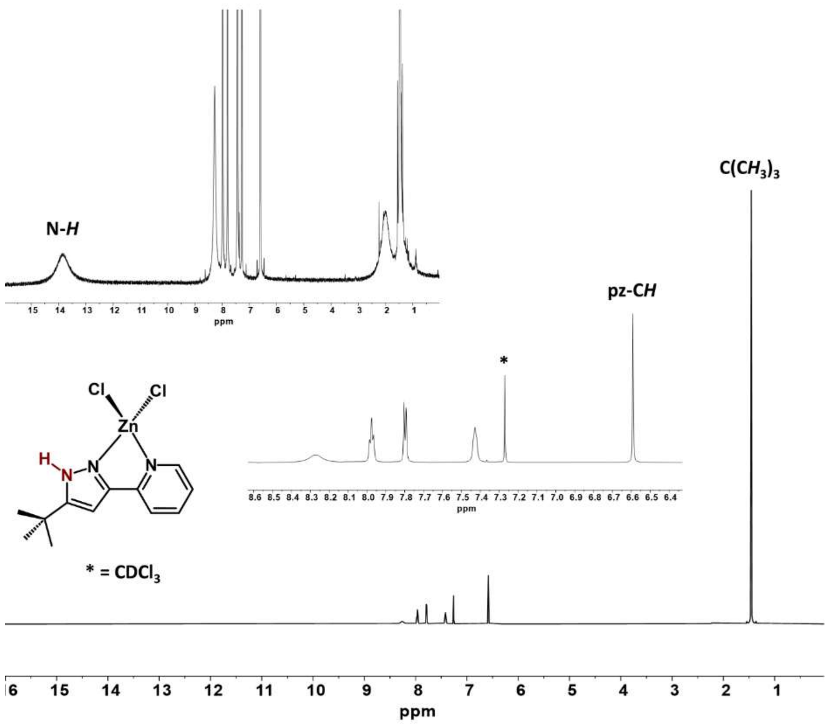

Figure S13 ^{1}H NMR spectrum ($CDCl_3$, 25 °C, 700 MHz) of ($^{H}NN^{tBu}$)$ZnCl_2$ (**1-Cl**). Insets highlight the N-H and aromatic region.

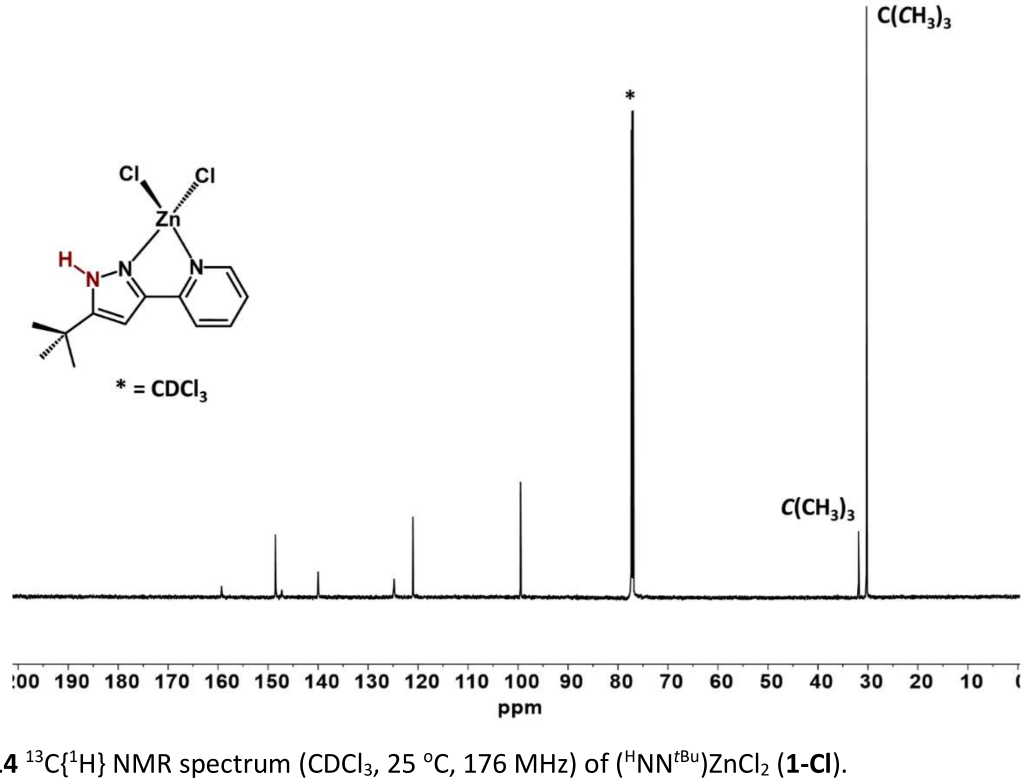

Figure S14 $^{13}C\{^{1}H\}$ NMR spectrum ($CDCl_3$, 25 °C, 176 MHz) of ($^{H}NN^{tBu}$)$ZnCl_2$ (**1-Cl**).

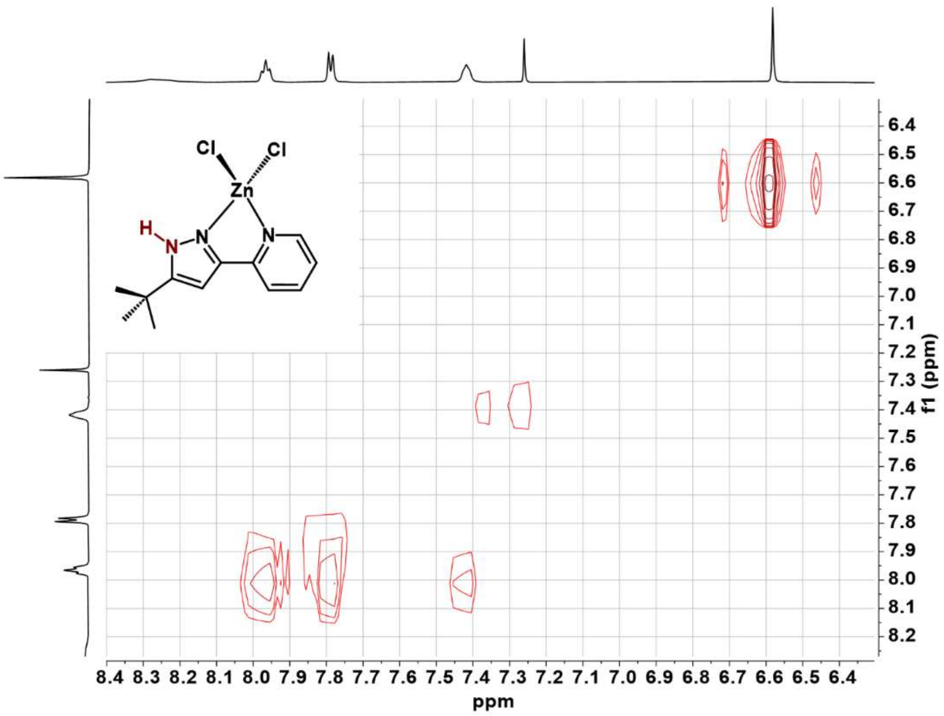

Figure S15 ^1H-^1H COSY spectrum ($CDCl_3$, 25 °C, 700 MHz) of ($^{H}NN^{tBu}$)$ZnCl_2$ (**1-Cl**).

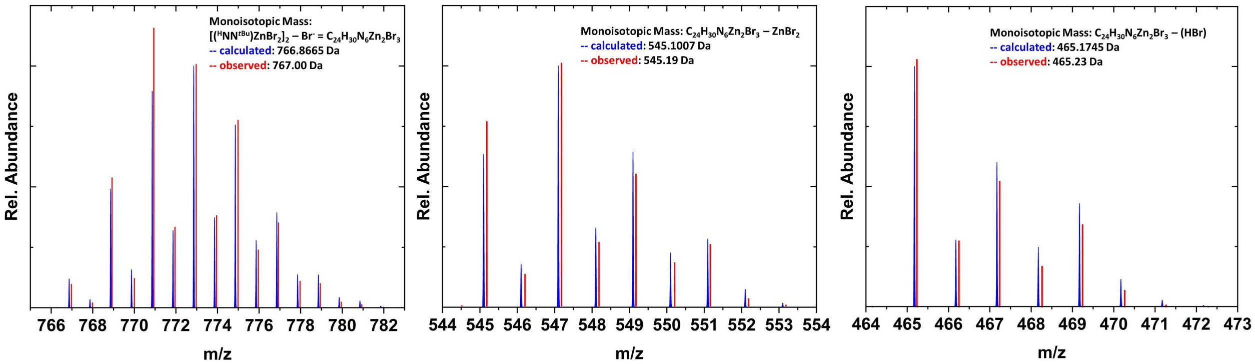

Figure S16 Mass spectrum of ($^{H}NN^{tBu}$)$ZnBr_2$ (**1-Br**). Left: [($^{H}NN^{tBu}$)$ZnBr_2$]$_2$ - Br^- = $[C_{24}H_{30}N_6Zn_2Br_3]^+$; Middle: $[C_{24}H_{30}N_6Zn_2Br_3]^+$ - $ZnBr_2$ = $[C_{24}H_{30}N_6Zn_1Br_1]^+$; Right: $[C_{24}H_{30}N_6Zn_1Br_1]^+$ - (HBr).

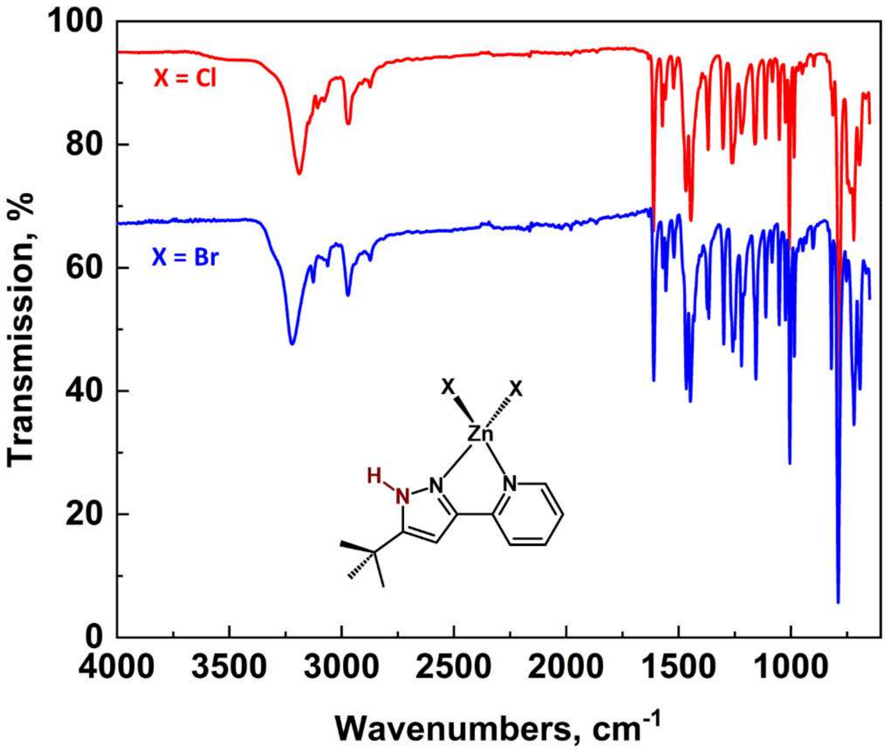

Figure S17 Infrared spectra (ATR, ambient temperature) of ($^{H}NN^{tBu}$)ZnX_2 (**1-X**; red, X = Cl; blue, X = Br).

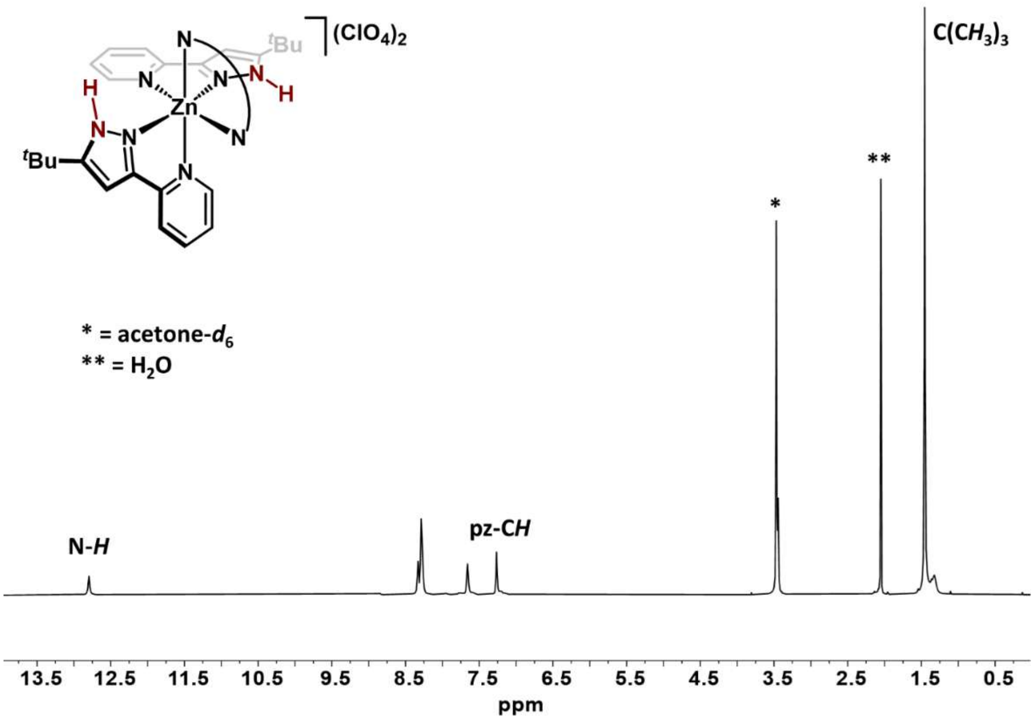

Figure S18 ^{1}H NMR spectrum (acetone-d_6, 25 °C, 700 MHz) of [($^{H}NN^{tBu}$)$_3$Zn][ClO_4]$_2$ (**2-ClO₄**).

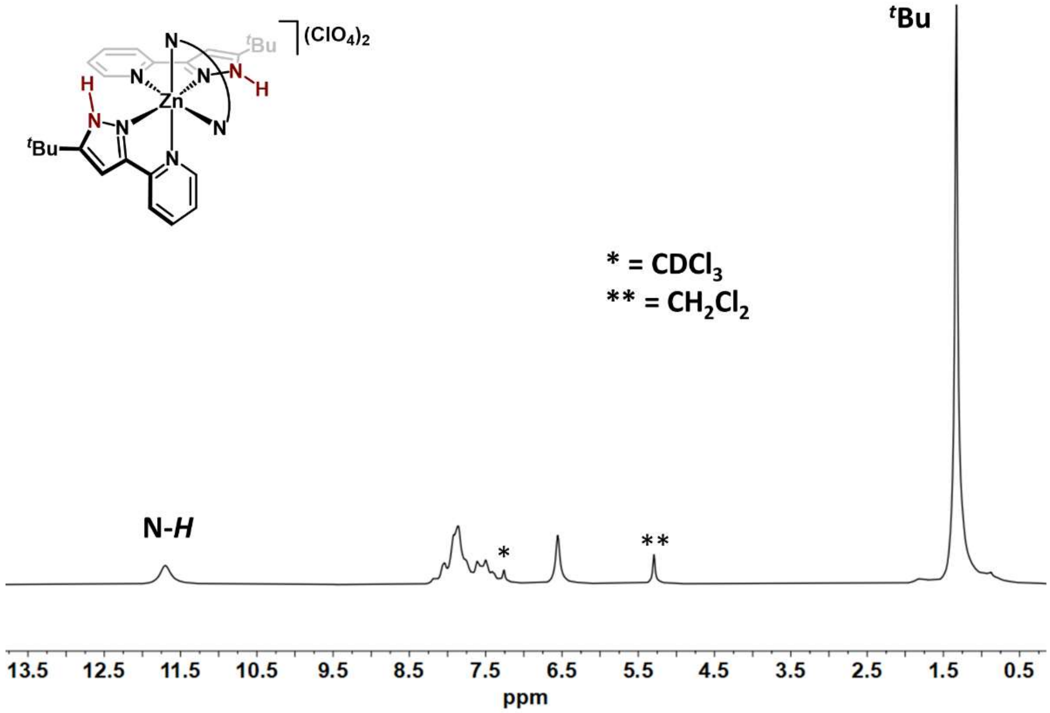

Figure S19 ^{1}H NMR spectrum ($CDCl_3$, 25 °C, 60 MHz) of $[(^{H}NN^{tBu})_3Zn][ClO_4]_2$ (**2-ClO$_4$**).

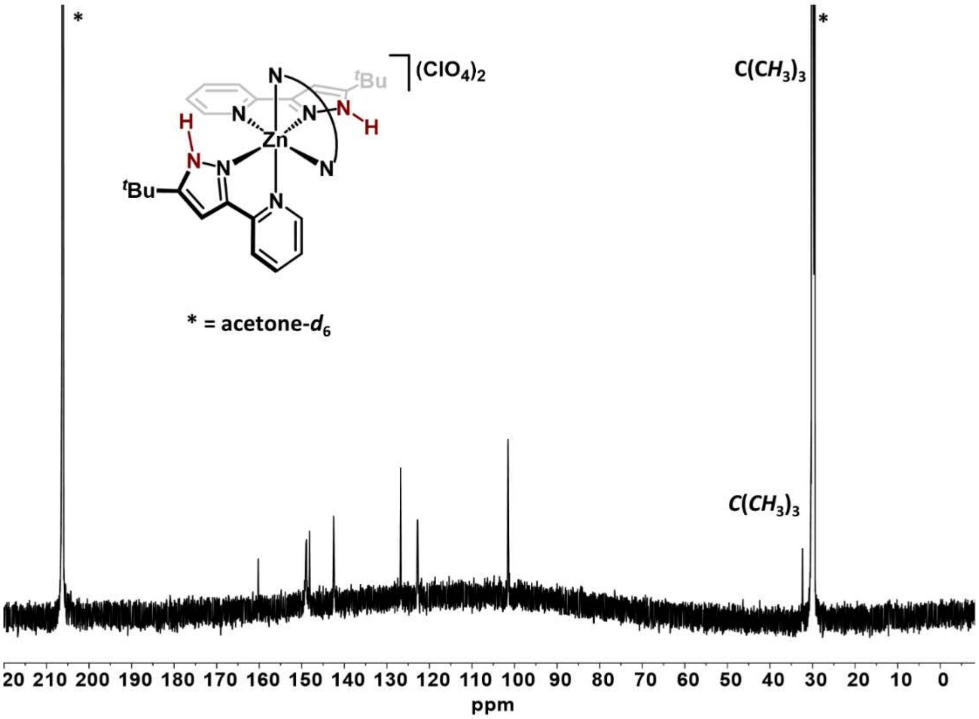

Figure S20 $^{13}C\{^{1}H\}$ NMR spectrum (acetone-d_6, 25 °C, 176 MHz) of $[(^{H}NN^{tBu})_3Zn][ClO_4]_2$ (**2-ClO$_4$**).

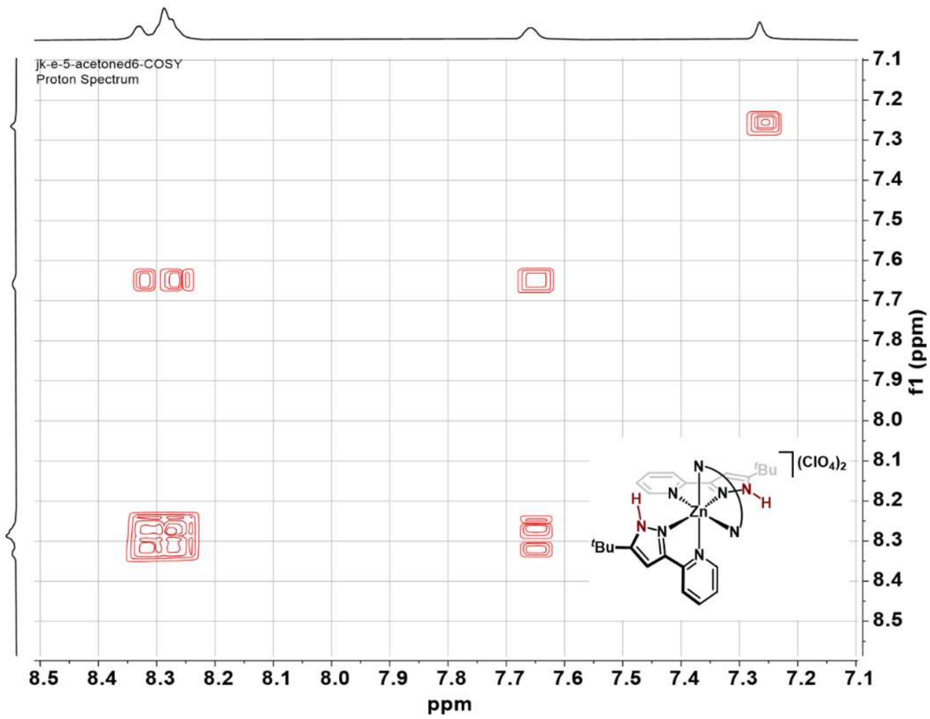

Figure S21 ^1H-^1H COSY spectrum (acetone-d_6, 25 °C, 700 MHz) of $[(^{H}NN^{tBu})_3Zn][ClO_4]_2$ (**2-ClO₄**).

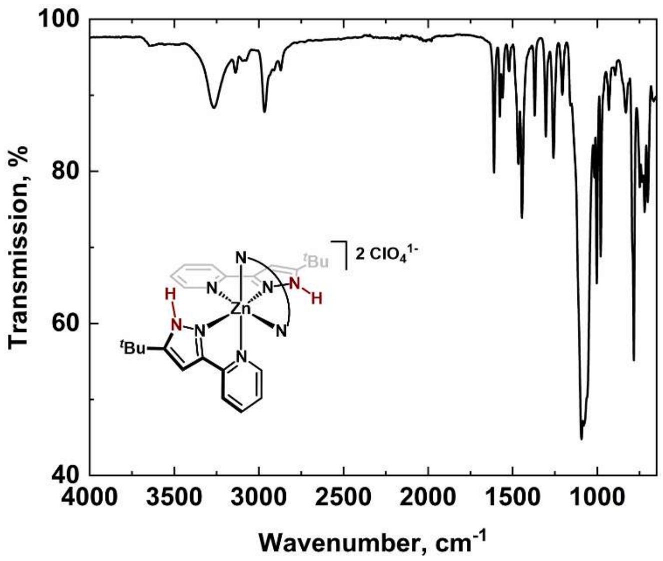

Figure S22 Infrared spectrum (ATR, 25 °C, neat) of $[(^{H}NN^{tBu})_3Zn][ClO_4]_2$ (**2-ClO₄**).

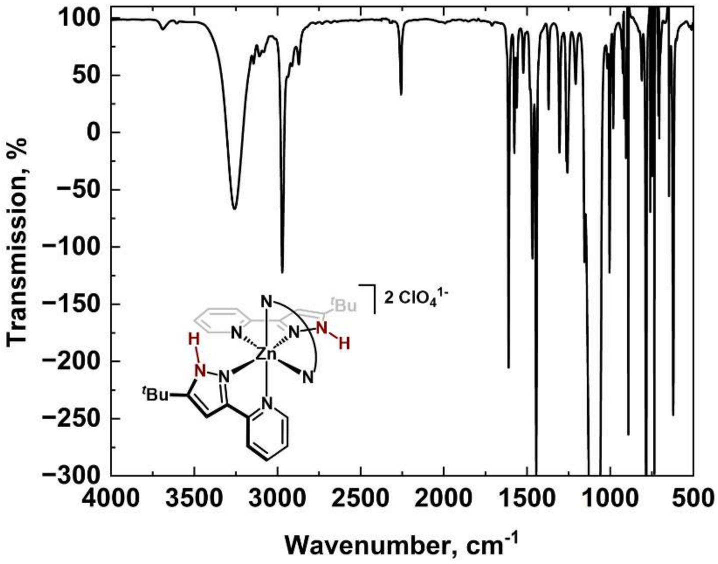

Figure S23 Infrared spectrum ($CDCl_3$, 25 °C) of $[(^{H}NN^{tBu})_3Zn][ClO_4]_2$ (**2-ClO₄**).

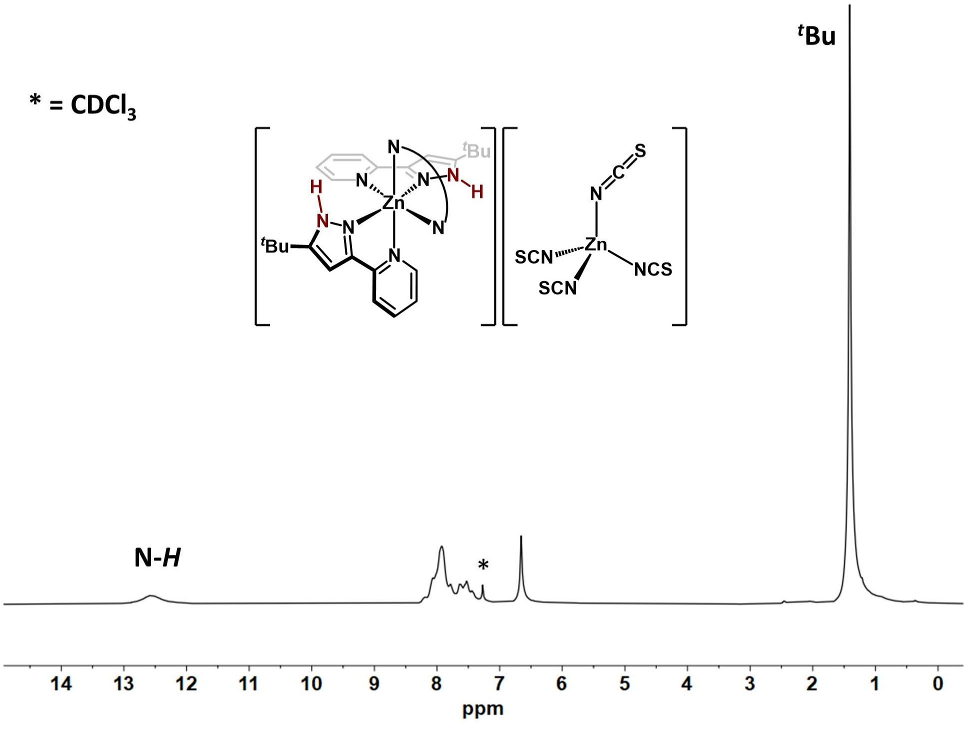

Figure S24 ^{1}H NMR spectrum ($CDCl_3$, 25 °C, 60 MHz) of $[(^{H}NN^{tBu})_3Zn][Zn(SCN)_4]$ (**2-SCN**).

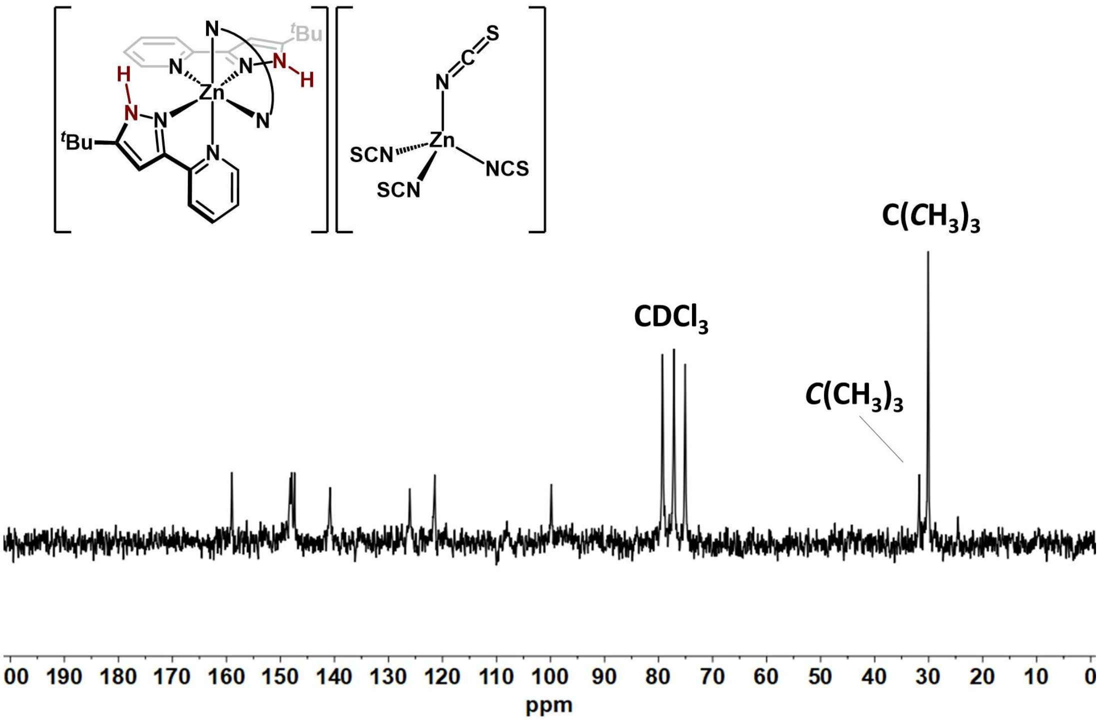

Figure S25 $^{13}C\{^1H\}$ NMR spectrum ($CDCl_3$, 25 °C, 15 MHz) of $[(^{H}NN^{tBu})_3Zn][Zn(SCN)_4]$ (**2-SCN**).

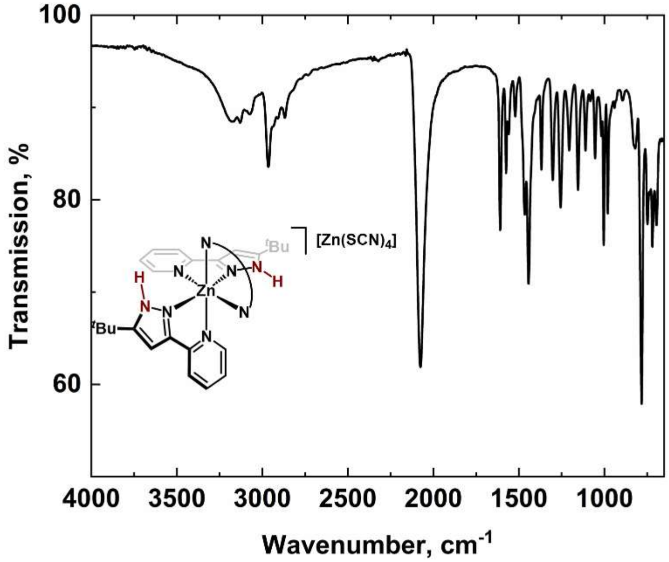

Figure S26 Infrared spectrum (ATR, 25 °C, neat) of $[(^{H}NN^{tBu})_3Zn][Zn(SCN)_4]$ (**2-SCN**).

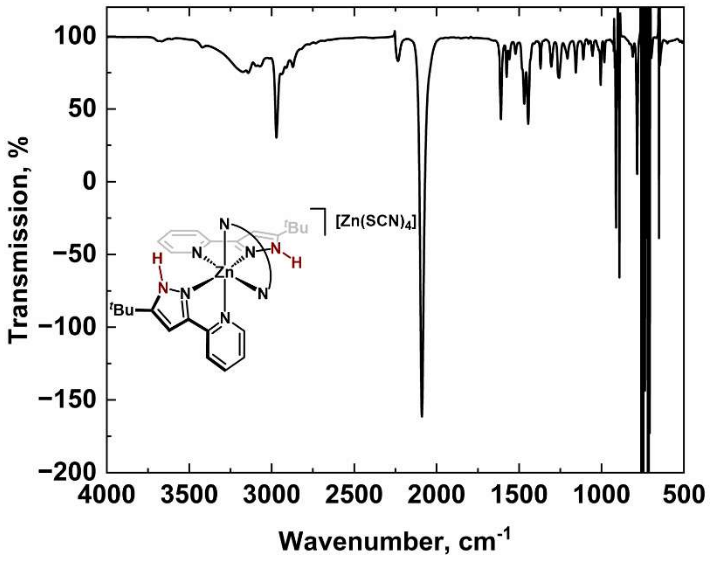

Figure S27 Infrared spectrum ($CDCl_3$, 25 °C) of [($^{H}NN^{tBu}$)$_3$Zn][Zn(SCN)$_4$] (**2-SCN**).

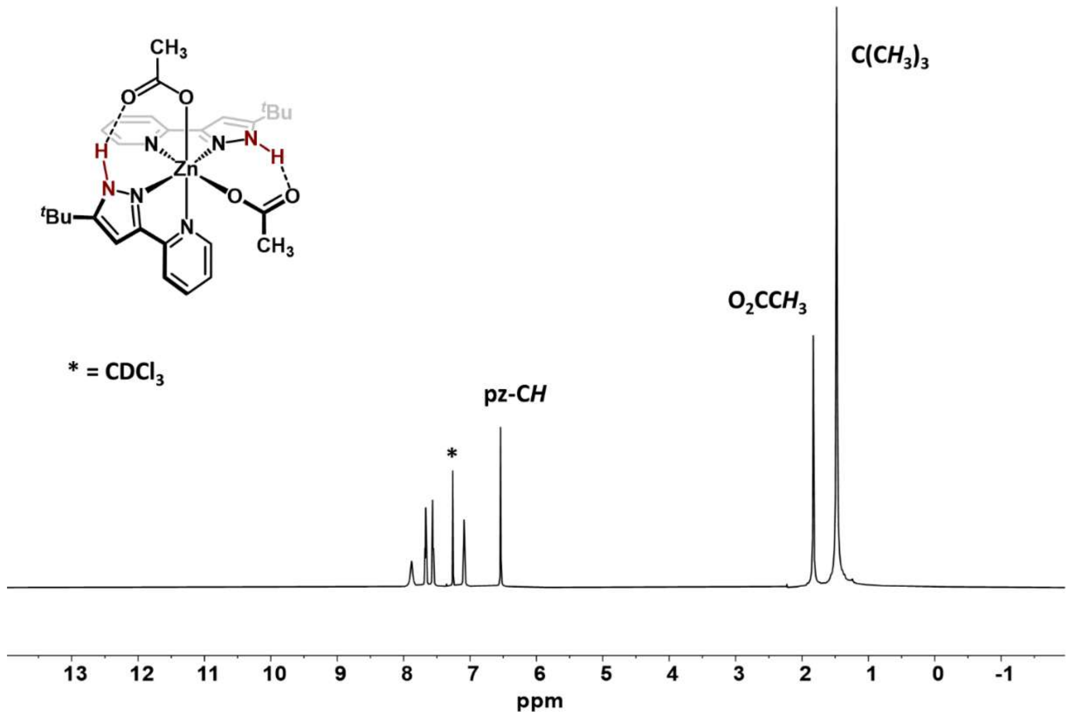

Figure S28 ^{1}H NMR spectrum ($CDCl_3$, 25 °C, 700 MHz) of ($^{H}NN^{tBu}$)$_2$Zn(OAc)$_2$ (**3**).

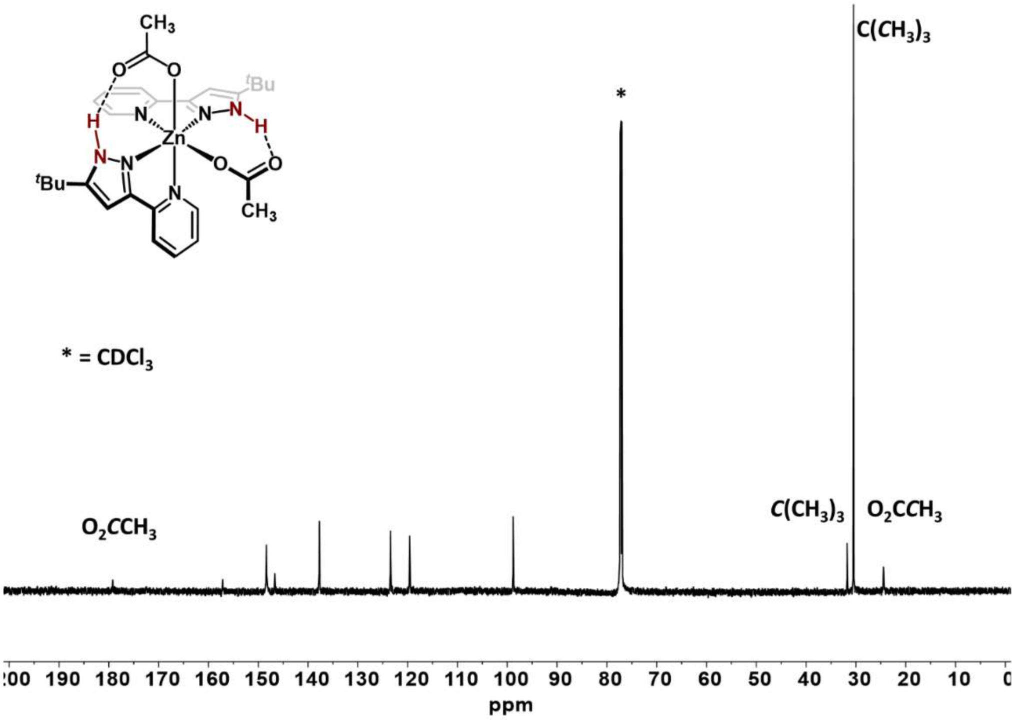

Figure S29 $^{13}C\{^{1}H\}$ NMR spectrum ($CDCl_3$, 25 °C, 176 MHz) of $(^{H}NN^{tBu})_2Zn(OAc)_2$ (**3**).

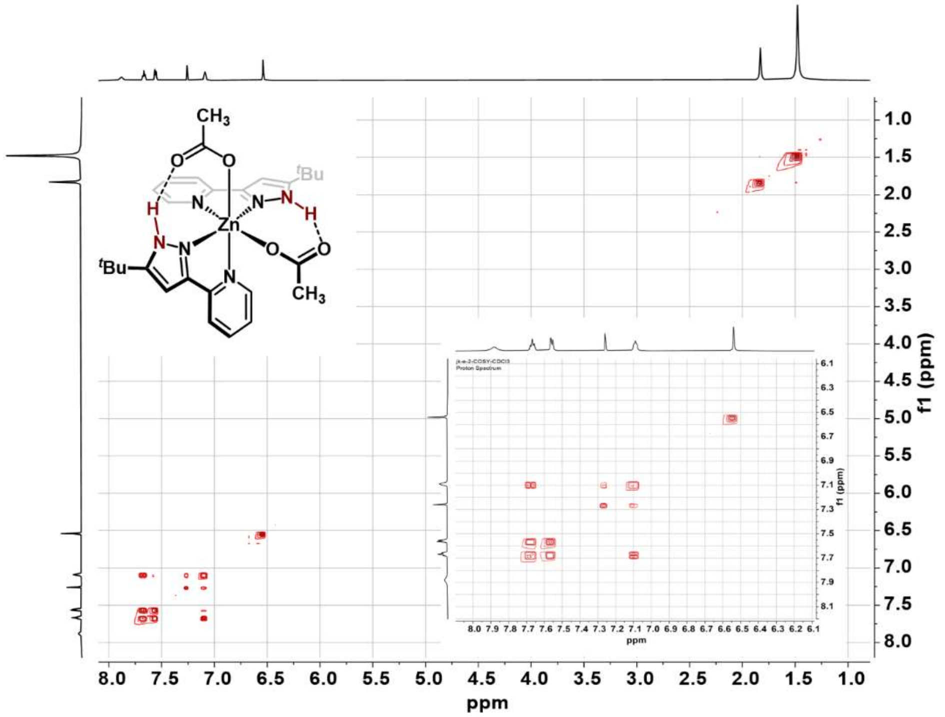

Figure S30 ^{1}H-^{1}H COSY spectrum ($CDCl_3$, 25 °C, 700 MHz) of $(^{H}NN^{tBu})_2Zn(OAc)_2$ (**3**).

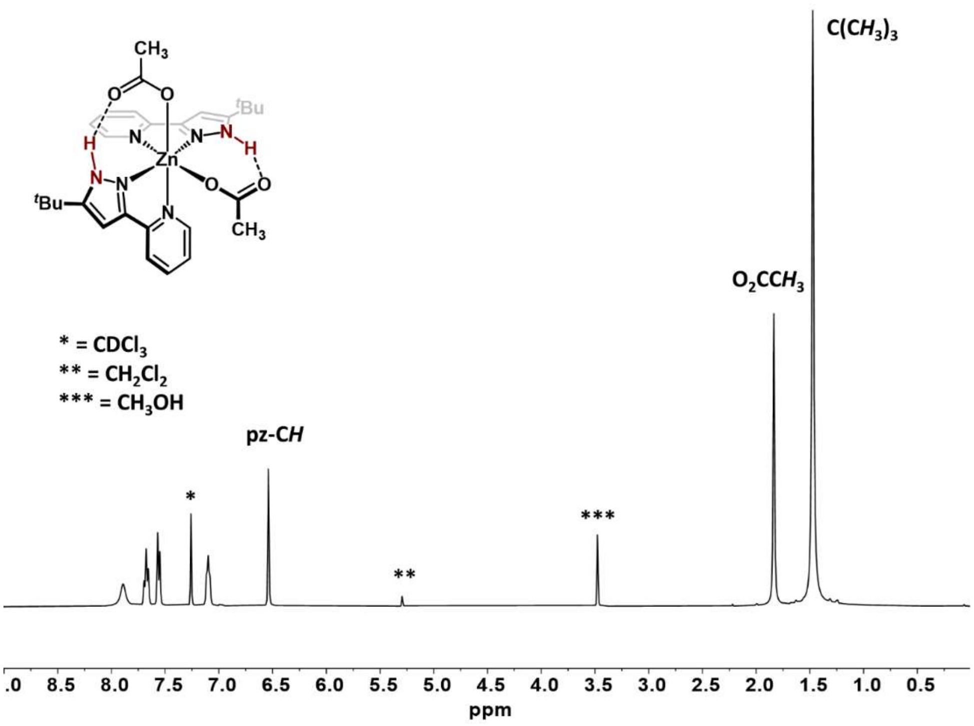

Figure S31 ^{1}H NMR spectrum ($CDCl_3$, 25 °C, 400 MHz) of $(^{H}NN^{tBu})_2Zn(OAc)_2$ (**3**).

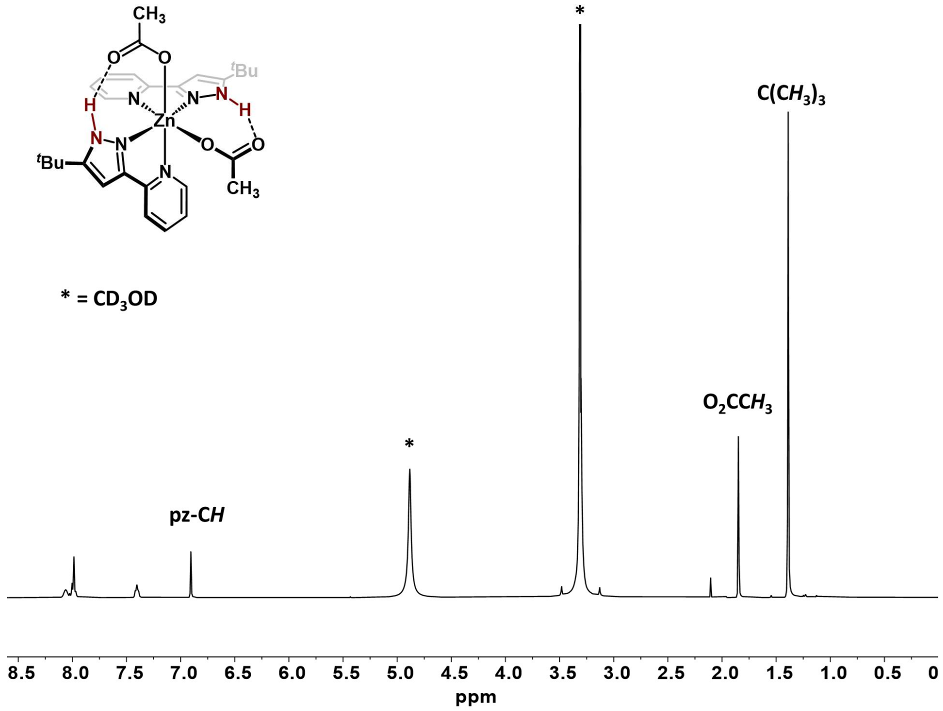

Figure S32 ^{1}H NMR spectrum (CD_3OD, 25 °C, 400 MHz) of $(^{H}NN^{tBu})_2Zn(OAc)_2$ (**3**).

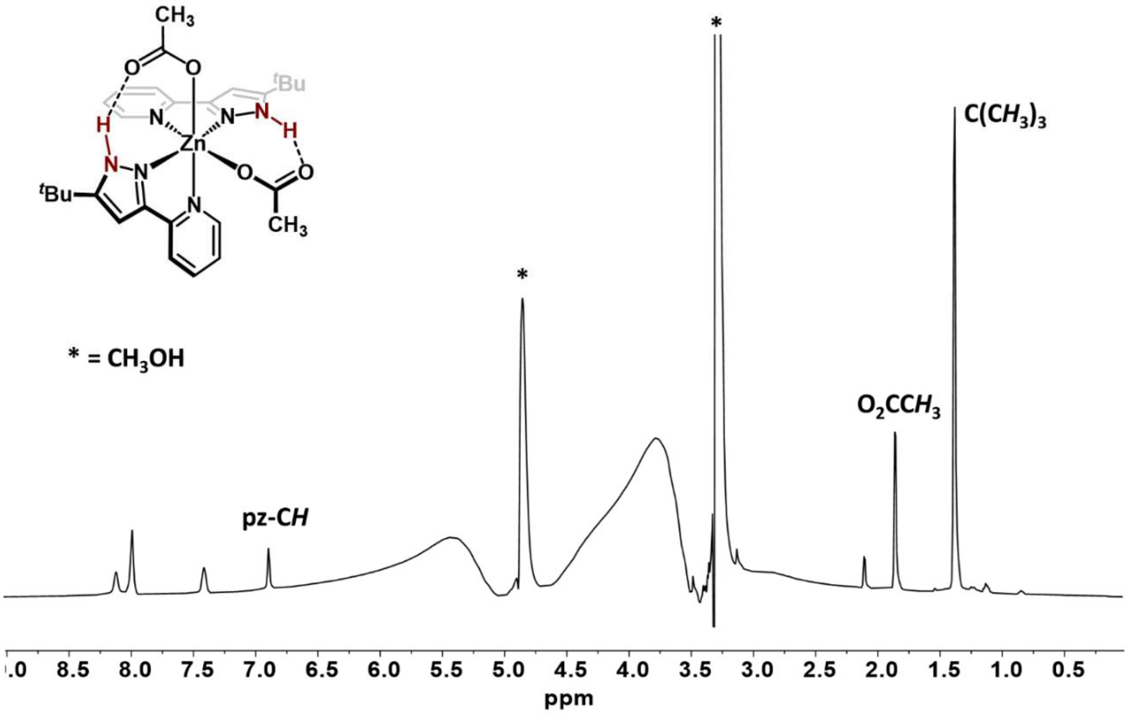

Figure S33 Crude ^{1}H NMR spectrum (CH_3OH, 25 °C, 400 MHz) of the reaction between $Zn(OAc)_2(H_2O)_2$ and $^{H}NN^{tBu}$. Note: the resonances near 3.3 and 4.8 ppm are the result of using CH_3OH.

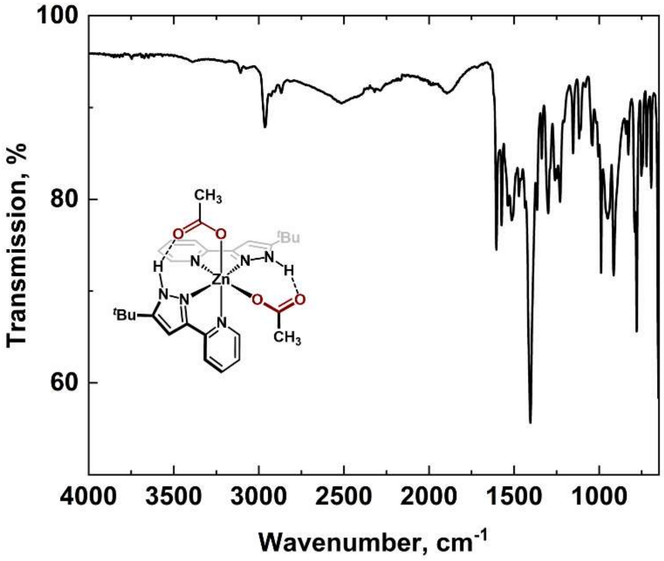

Figure S34 Infrared spectrum (ATR, 25 °C, neat) of $(^{H}NN^{tBu})_2Zn(OAc)_2$ (**3**).

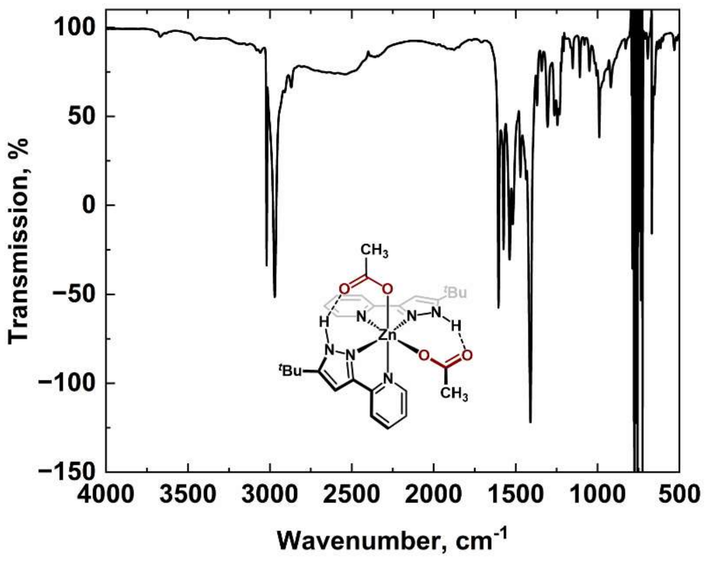

Figure S35 Infrared spectrum ($CHCl_3$, 25 °C) of $(^{H}NN^{tBu})_2Zn(OAc)_2$ (**3**).

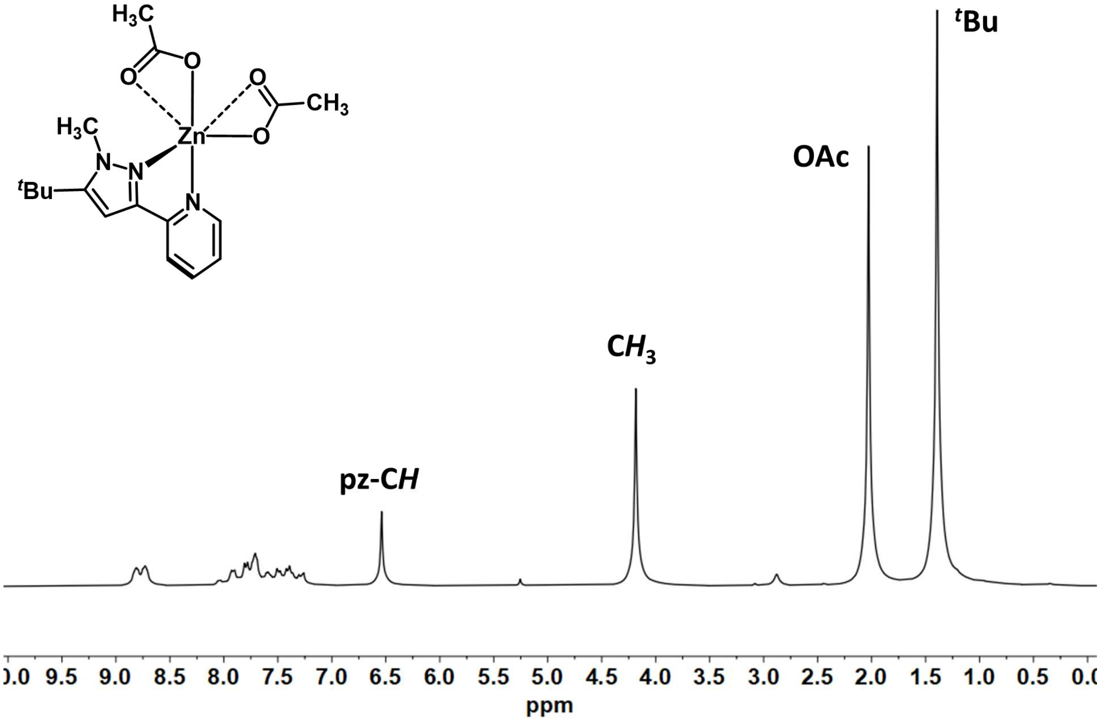

Figure S36 ^{1}H NMR spectrum ($CDCl_3$, 25 °C, 60 MHz) of $(^{Me}NN^{tBu})Zn(OAc)_2$ (**3’**).

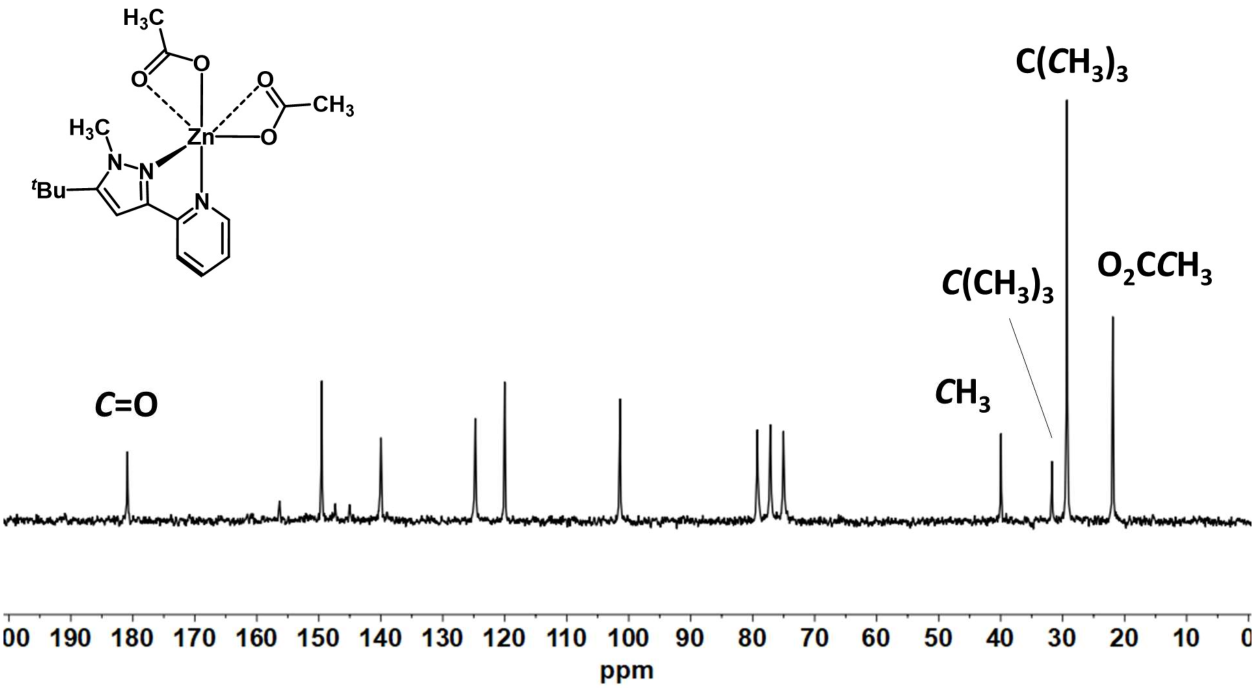

Figure S37 ^{13}C{^{1}H} NMR spectrum ($CDCl_3$, 25 °C, 15 MHz) of (^{Me}NN^{tBu})Zn(OAc)$_2$ (**3'**).

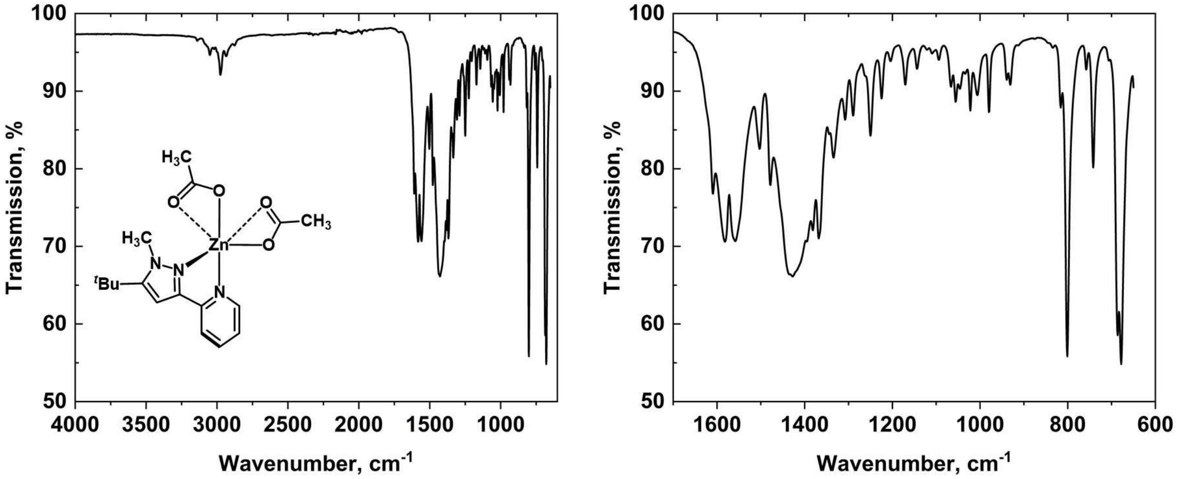

Figure S38 Infrared spectrum (ATR, neat, 25 °C) of (^{Me}NN^{tBu})Zn(OAc)$_2$ (**3'**). Left: full spectrum; Right: low energy region.

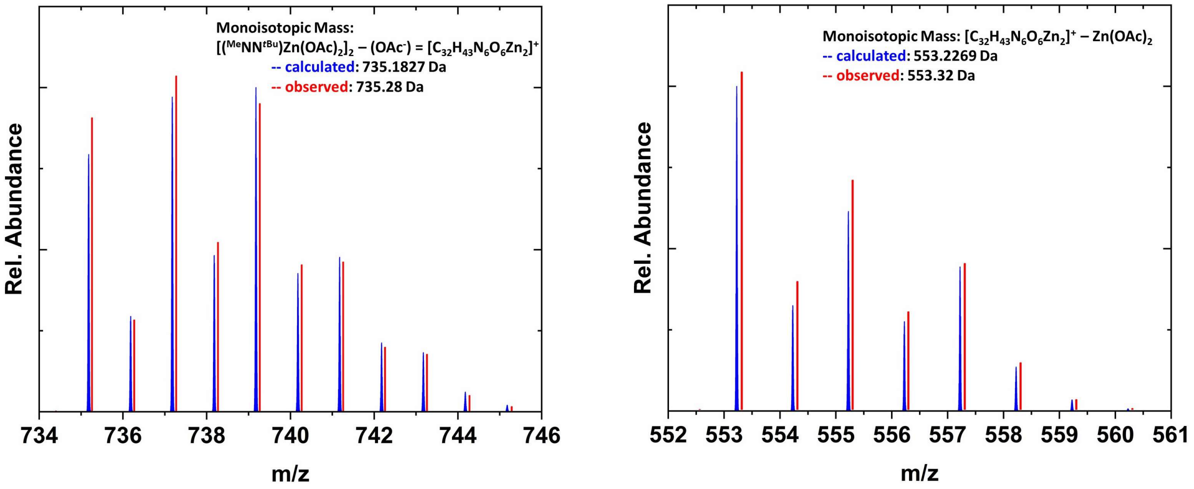

Figure S39 Mass spectrum of (^{Me}NN^{tBu})Zn(OAc)$_2$ (**3'**). Left: [(^{H}NN^{tBu})Zn(OAc)$_2$]$_2$ – (OAc$^-$) = $[C_{32}H_{43}N_6O_6Zn_2]^+$; Right: $[C_{32}H_{43}N_6O_6Zn_2]^+$ - Zn(OAc)$_2$ = $[C_{28}H_{37}N_6O_2Zn_1]^+$.

Scheme S1. Attempted metalation with 2-(4-(*tert*-butyl)-pyrazol-1-yl)pyridine.

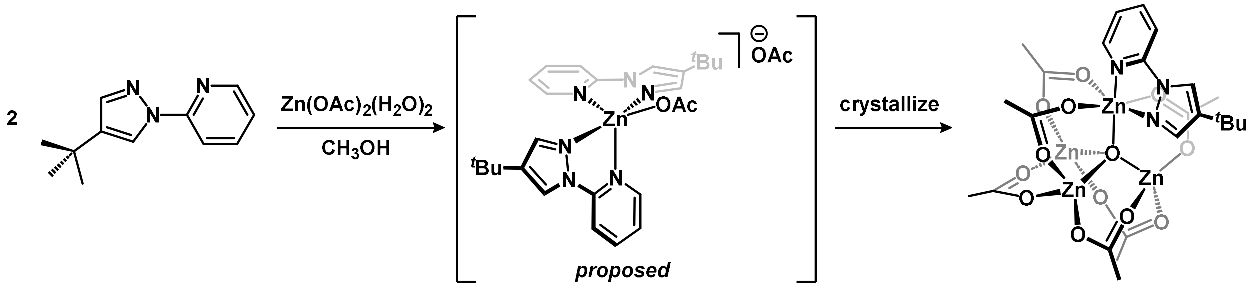

The constitutional isomer of ^{H}NN^{tBu}, 2-(4-(*tert*-butyl)-pyrazol-1-yl)pyridine (L), was synthesized by Cu(I)-mediated cross coupling of 2-bromopyridine and 1H-4-*tert*-butylpyrazole following established protocol.[13] The ligand was metalated with Zn(OAc)$_2$(H$_2$O)$_2$ under analogous protocol to **3** and **3'** as shown above. Multiple attempts to crystallize the product of this reaction only resulted in X-ray quality crystals of (L)Zn$_4$O(OAc)$_6$ – a presumed minor decomposition product from prolonged storage in solution open to ambient conditions. Because of this, full characterization of these molecules was not performed. The X-ray structure of (L)Zn$_4$O(OAc)$_6$ is provided below.

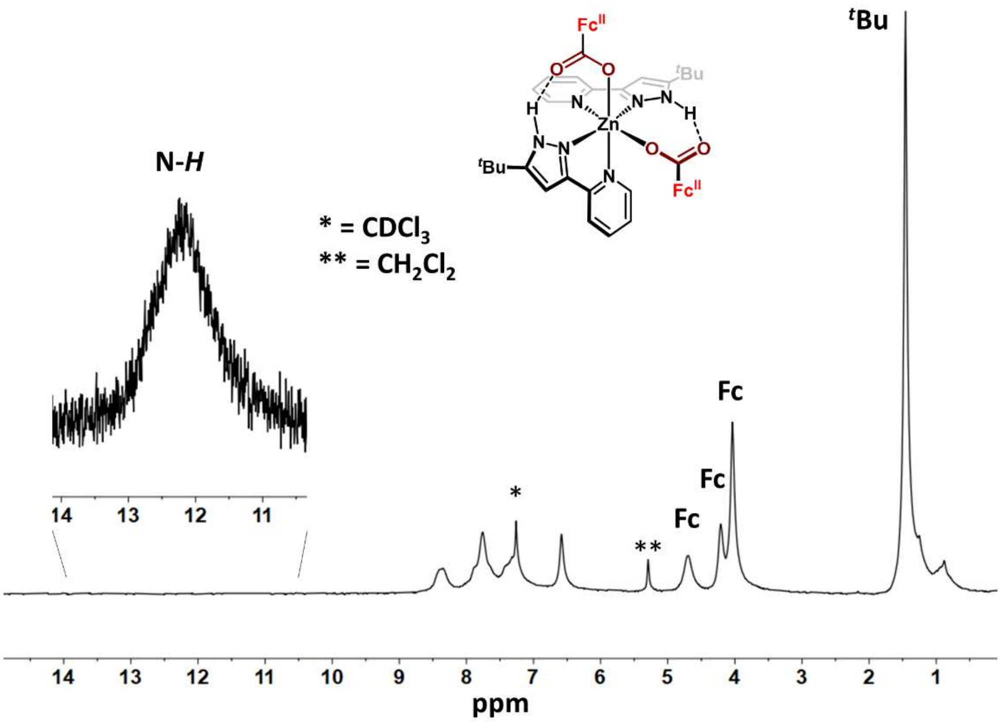

Figure S40 ^{1}H NMR spectrum ($CDCl_3$, 25 ^{o}C, 60 MHz) of $(^{H}NN^{tBu})_2Zn(O_2CFc)_2$ (**4**).

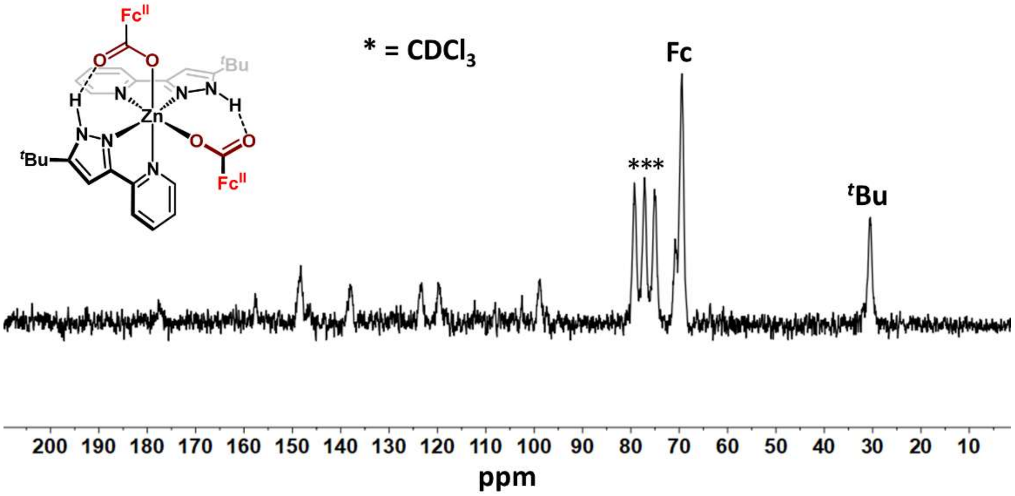

Figure S41 $^{13}C\{^{1}H\}$ NMR spectrum ($CDCl_3$, 25 ^{o}C, 15 MHz) of $(^{H}NN^{tBu})_2Zn(O_2CFc)_2$ (**4**).

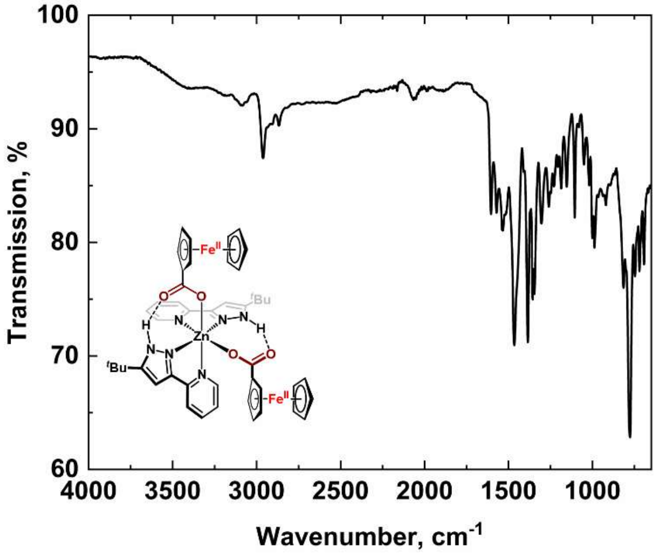

Figure S42 Infrared spectrum (ATR, 25 °C, neat) of $(^{H}NN^{tBu})_2Zn(O_2CFc)_2$ (**4**).

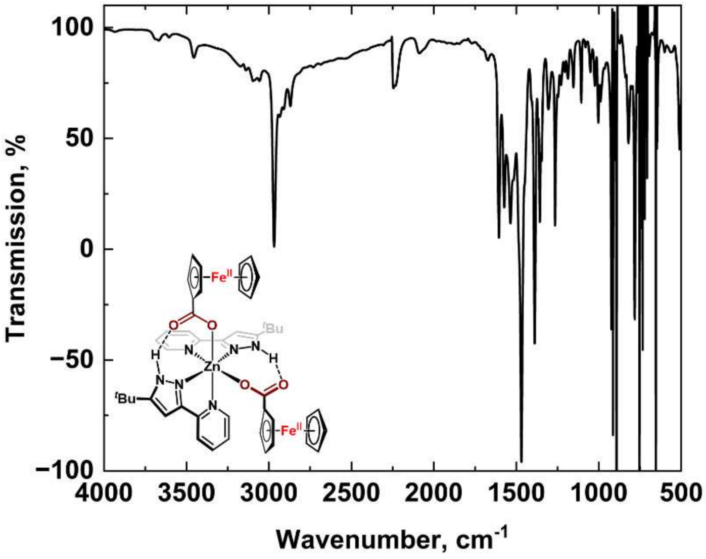

Figure S43 Infrared spectrum ($CDCl_3$, 25 °C) of $(^{H}NN^{tBu})_2Zn(O_2CFc)_2$ (**4**).

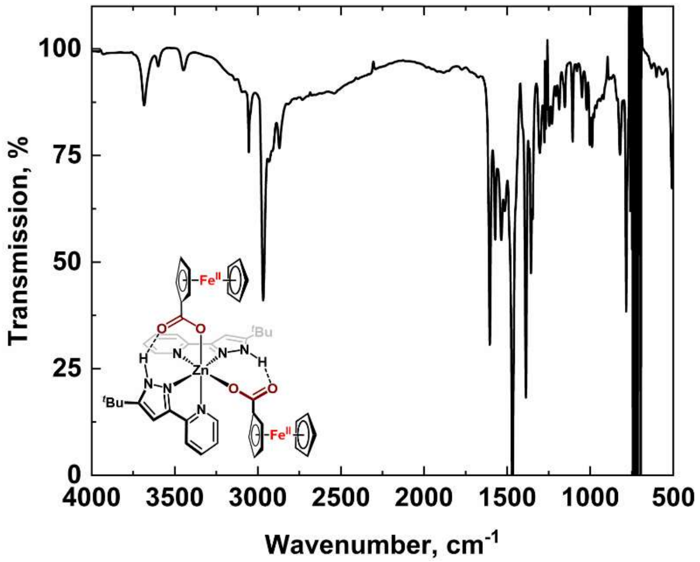

Figure S44 Infrared spectrum (CH_2Cl_2, 25 °C) of ($^{H}NN^{tBu}$)$_2$Zn(O_2CFc)$_2$ (**4**).

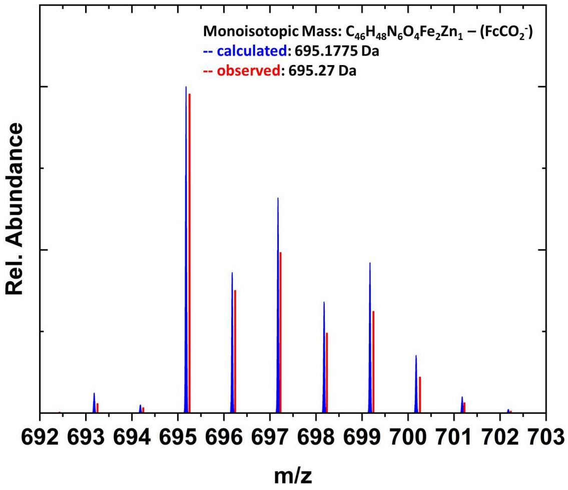

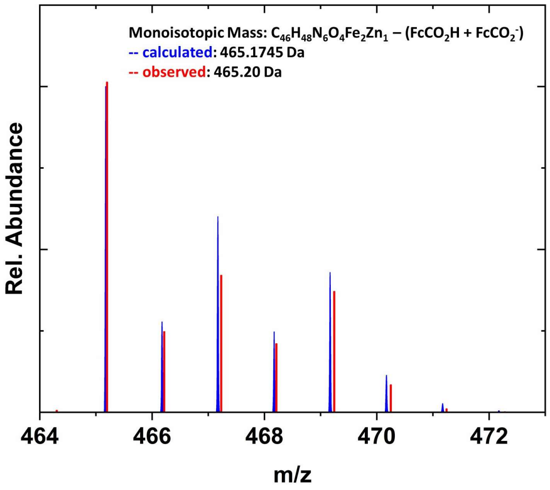

Figure S45 Mass spectrum of ($^{H}NN^{tBu}$)$_2$Zn(O_2CFc)$_2$ (**4**). Left: M - $FcCO_2^-$; Right: M - ($FcCO_2^-$ + $FcCO_2H$).

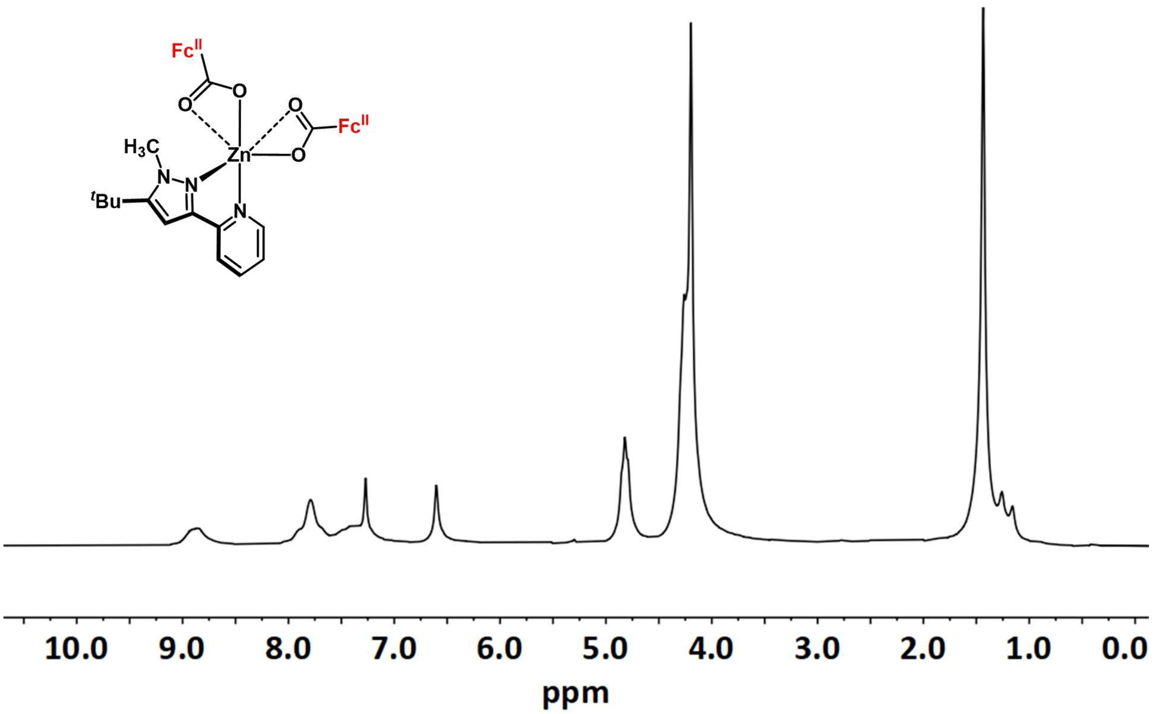

Figure S46 ^{1}H NMR spectrum ($CDCl_3$, 25 °C, 60 MHz) of ($^{Me}NN^{tBu}$)$Zn(O_2CFc)_2$ (**4'**).

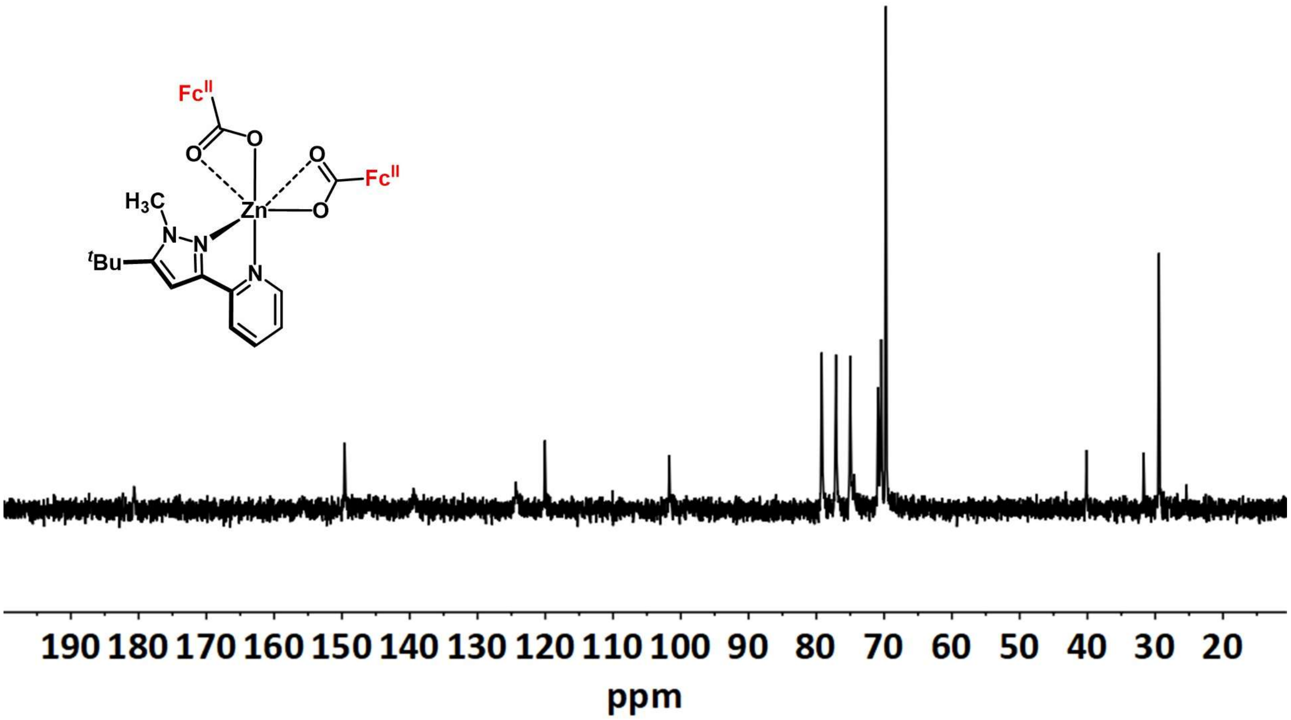

Figure S47 ^{13}C NMR spectrum ($CDCl_3$, 25 °C, 15 MHz) of ($^{Me}NN^{tBu}$)$Zn(O_2CFc)_2$ (**4'**).

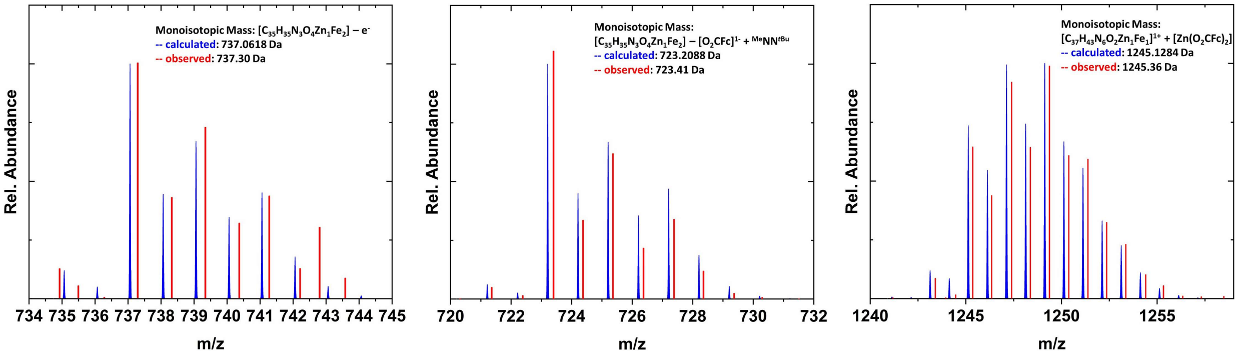

Figure S48 Mass spectrum of ($^{Me}NN^{tBu}$)$Zn(O_2CFc)_2$ (**4'**). Left: M – e^-; Middle: M - $(ArCO_2)^-$ + $^{Me}NN^{tBu}$; Right: $[M(ArCO_2)(^{Me}NN^{tBu})]^-$ + $Zn(O_2CFc)_2$.

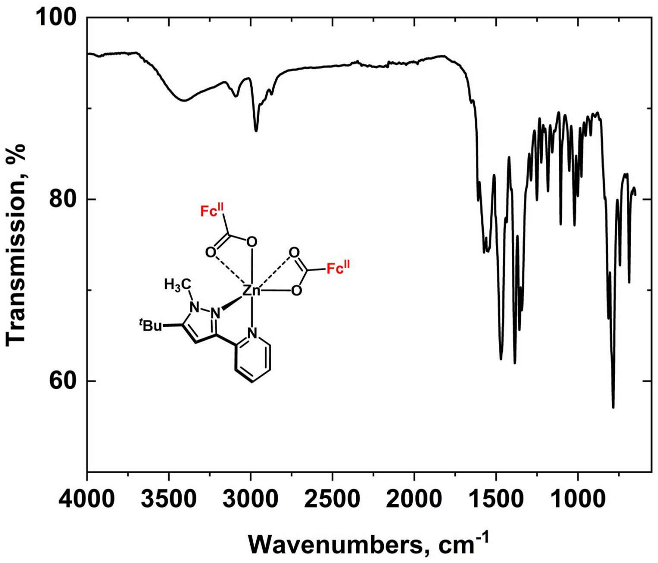

Figure S49 Infrared spectrum (ATR, 25 °C, neat) of ($^{Me}NN^{tBu}$)$Zn(O_2CFc)_2$ (**4'**). This spectrum was obtained open to air.

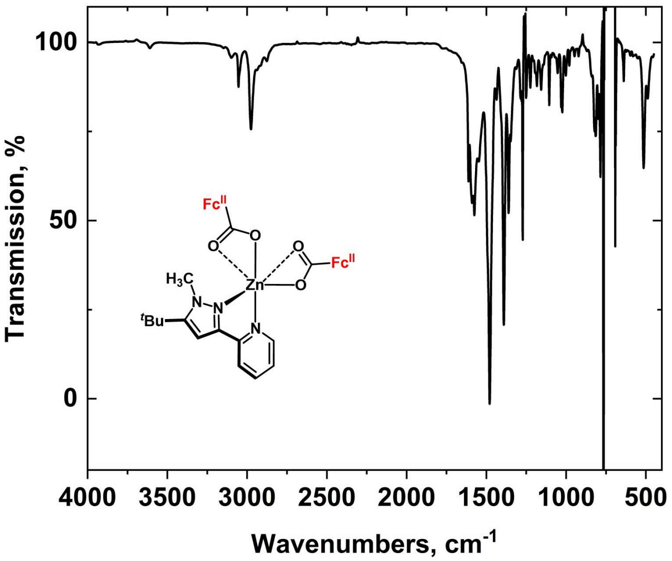

Figure S50 Infrared spectrum (CH_2Cl_2) of ($^{Me}NN^{tBu}$)Zn(O_2CFc)$_2$ (**4'**). This spectrum was obtained under an inert atmosphere.

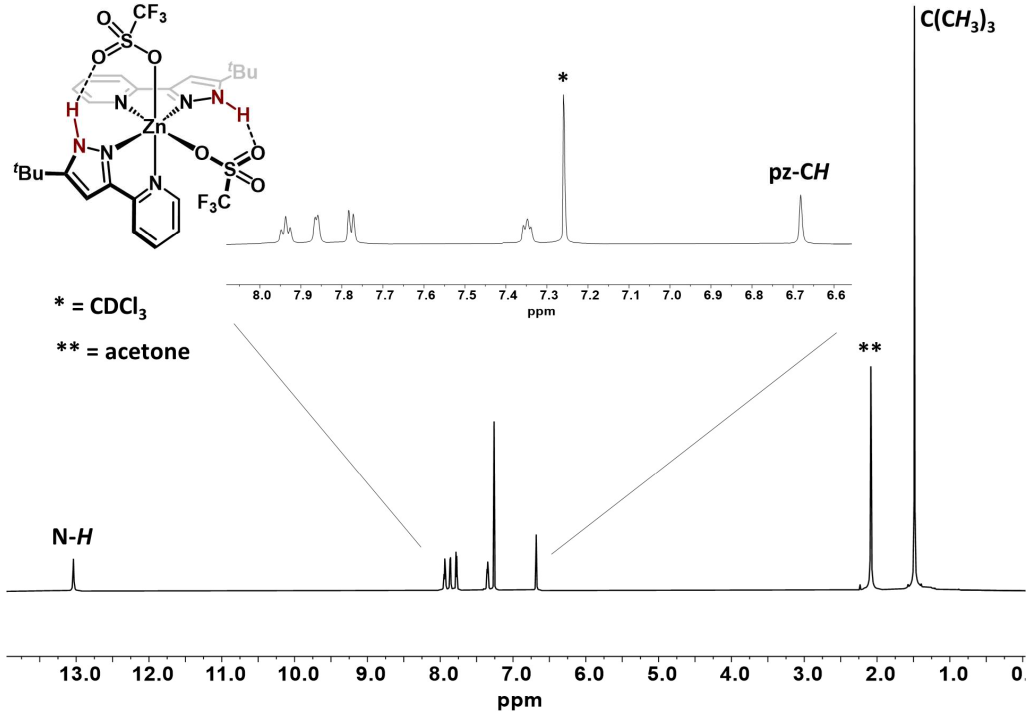

Figure S51 ^{1}H NMR spectrum ($CDCl_3$, 25 °C, 700 MHz) of ($^{H}NN^{tBu}$)$_2$Zn(OTf)$_2$ (**5**). Inset highlights the aromatic region.

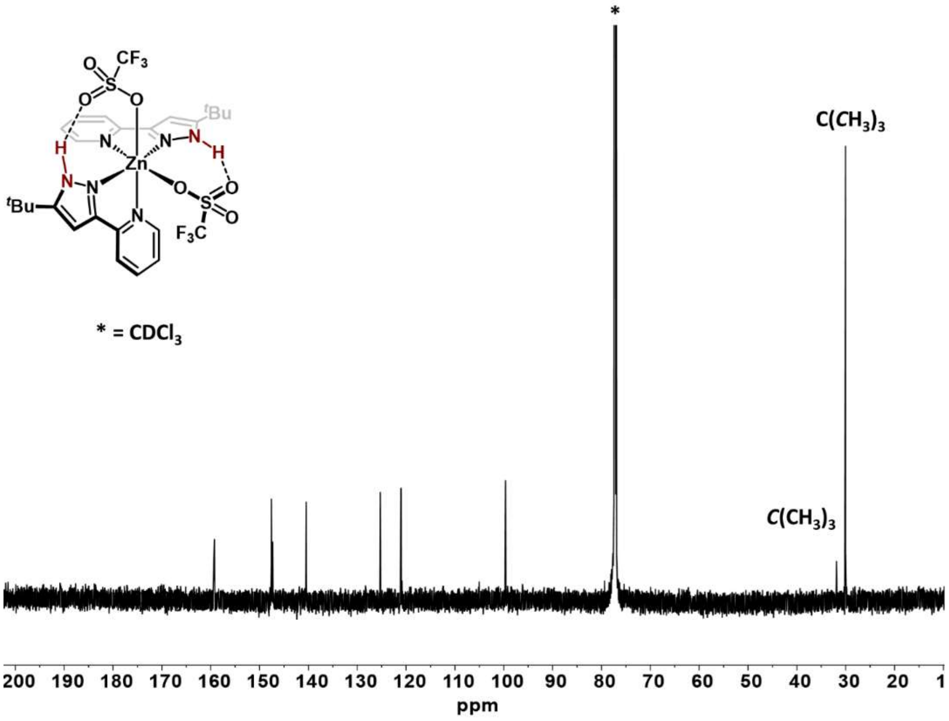

Figure S52 $^{13}C\{^{1}H\}$ NMR spectrum ($CDCl_3$, 25 °C, 176 MHz) of $(^{H}NN^{tBu})_2Zn(OTf)_2$ (**5**).

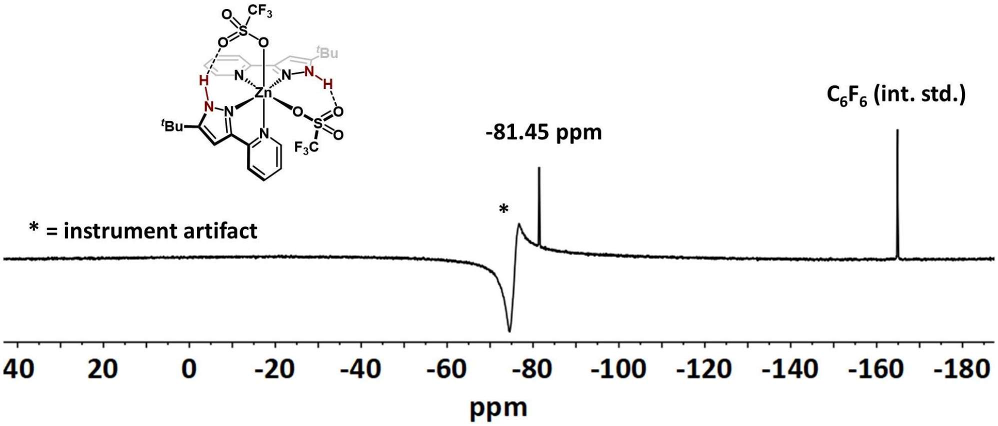

Figure S53 ^{19}F NMR spectrum ($CDCl_3$, 25 °C, 56 MHz) of $(^{H}NN^{tBu})_2Zn(OTf)_2$ (**5**).

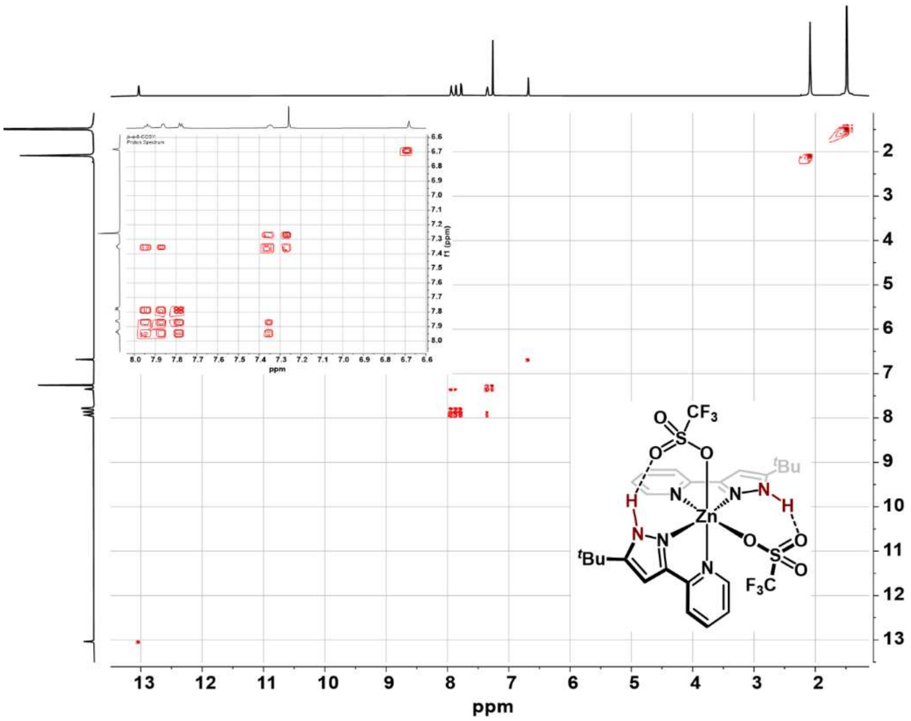

Figure S54 ^{1}H-^{1}H COSY spectrum ($CDCl_3$, 25 °C, 700 MHz) of ($^{H}NN^{tBu}$)$_2$Zn(OTf)$_2$ (**5**).

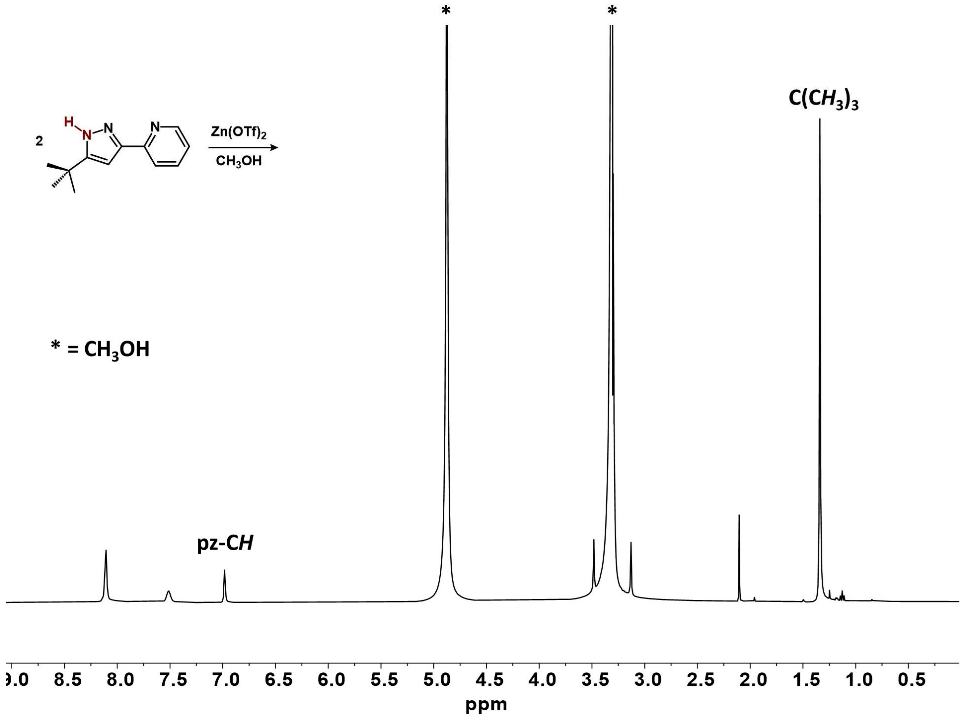

Figure S55 Crude ^{1}H NMR spectrum (CH_3OH, 25 °C, 400 MHz) of the reaction between Zn(OTf)$_2$ and $^{H}NN^{tBu}$.

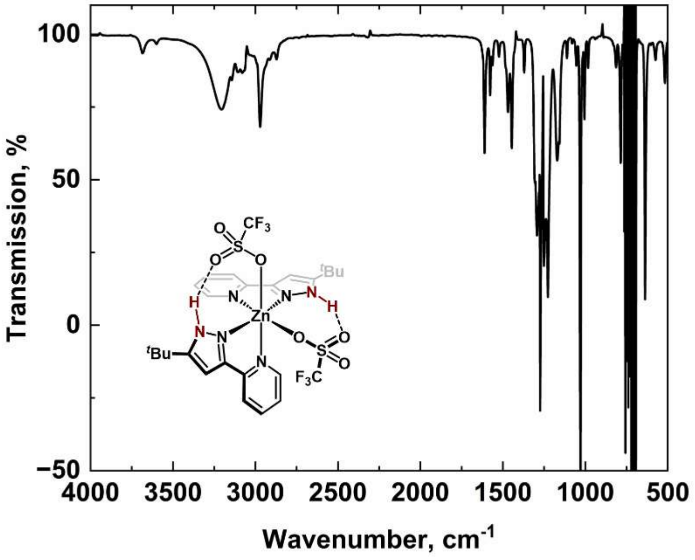

Figure S56 Infrared spectrum (CH_2Cl_2, 25 °C) of ($^{H}NN^{tBu}$)$_2$Zn(OTf)$_2$ (**5**).

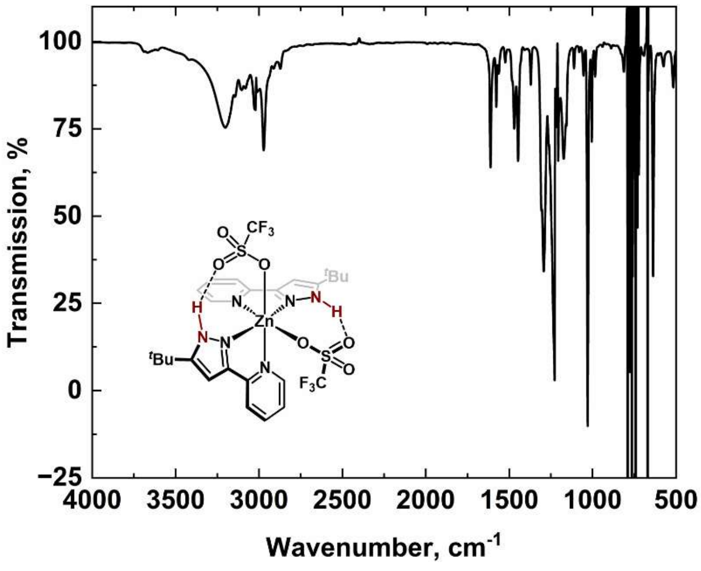

Figure S57 Infrared spectrum ($CHCl_3$, 25 °C) of ($^{H}NN^{tBu}$)$_2$Zn(OTf)$_2$ (**5**).

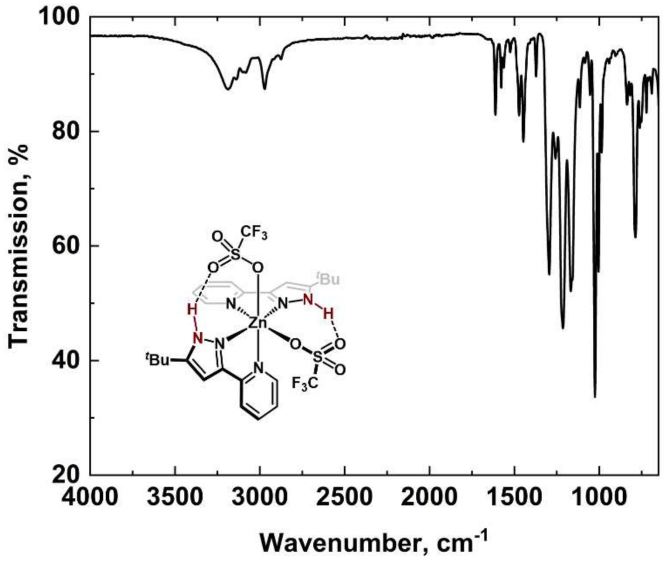

Figure S58 Infrared spectrum (ATR, 25 °C, neat) of $(^{H}NN^{tBu})_2Zn(OTf)_2$ (**5**).

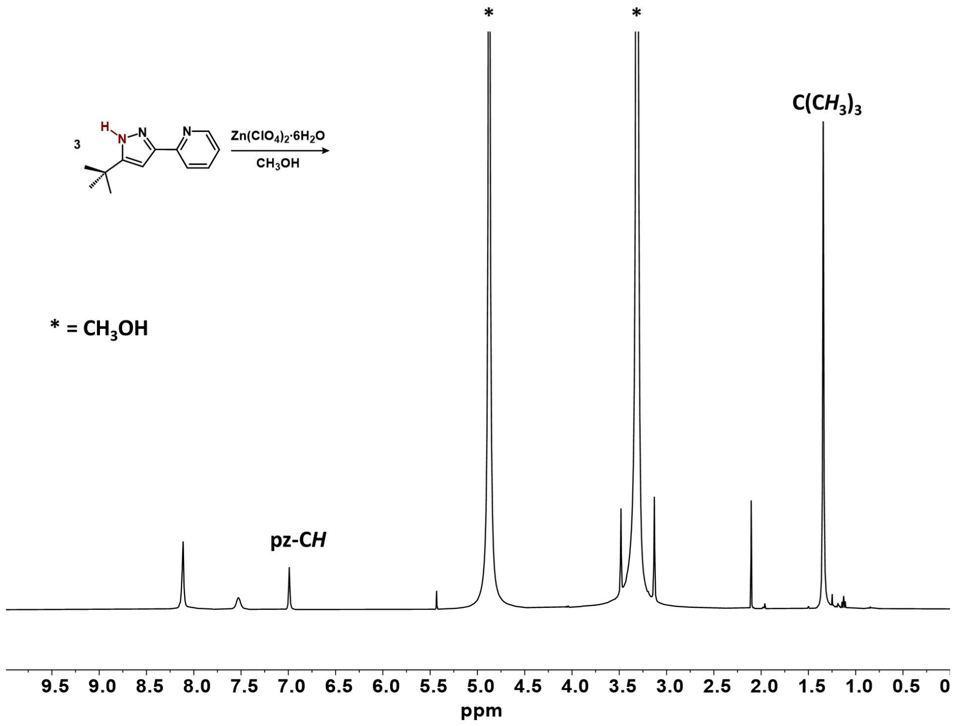

Figure S59 Crude ^{1}H NMR spectrum (CH_3OH, 25 °C, 400 MHz) of the reaction between $Zn(ClO_4)_2{\cdot}6H_2O$ and $^{H}NN^{tBu}$.

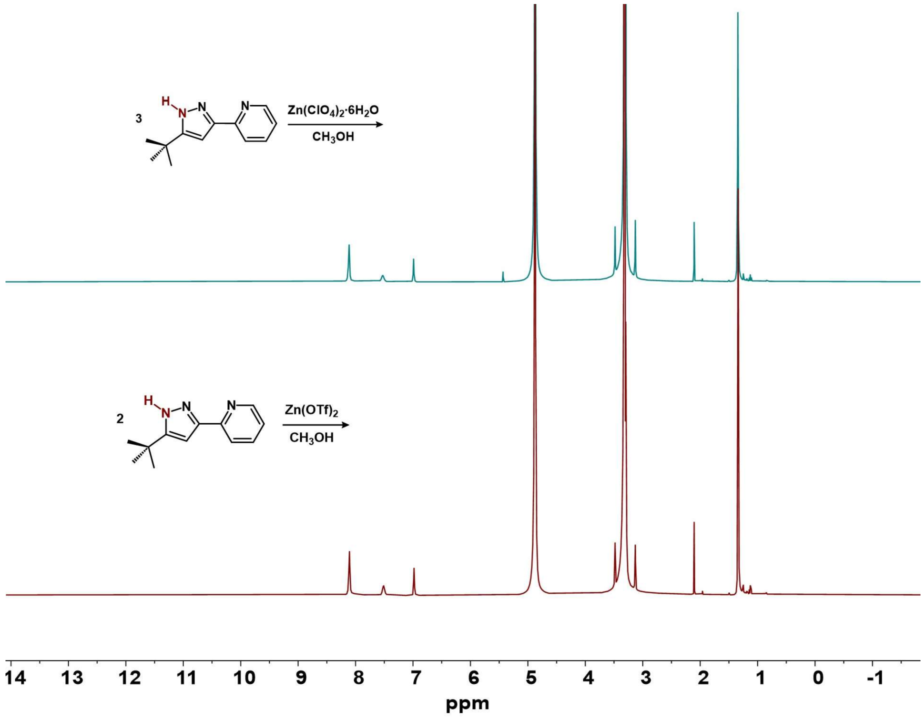

Figure S60 Crude ^{1}H NMR spectra (CH_3OH, 25 °C, 400 MHz) overlay. Top: reaction between $Zn(ClO_4)_2{\cdot}6H_2O$ and $^{H}NN^{tBu}$. Bottom: reaction between $Zn(OTf)_2$ and $^{H}NN^{tBu}$.

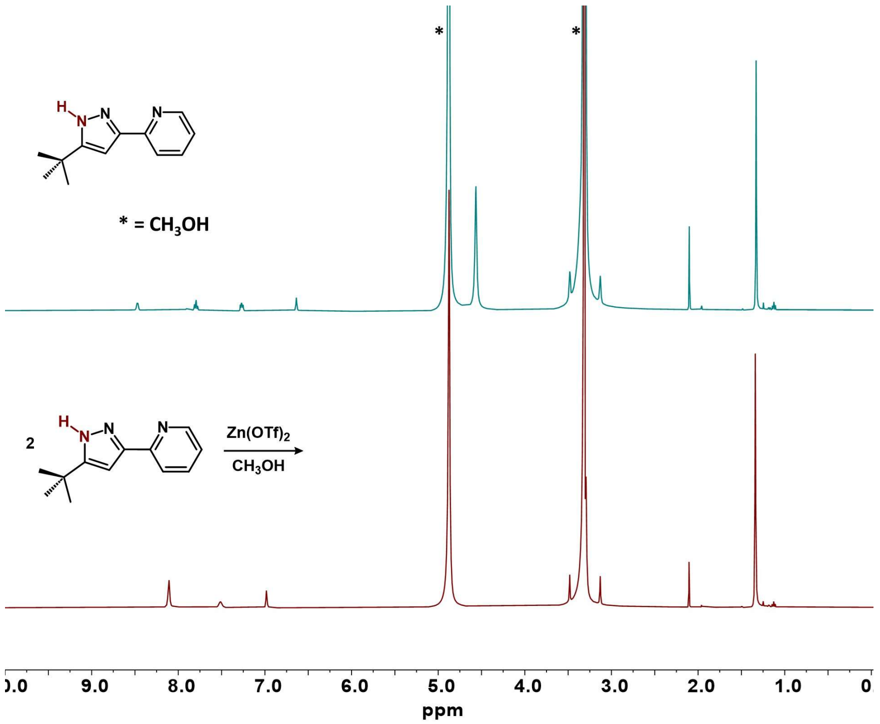

Figure S61 ^{1}H NMR spectra (CH_3OH, 25 °C, 400 MHz) overlay. Top: spectrum of $^{H}NN^{tBu}$ ligand. Bottom: crude reaction between $Zn(OTf)_2$ and $^{H}NN^{tBu}$.

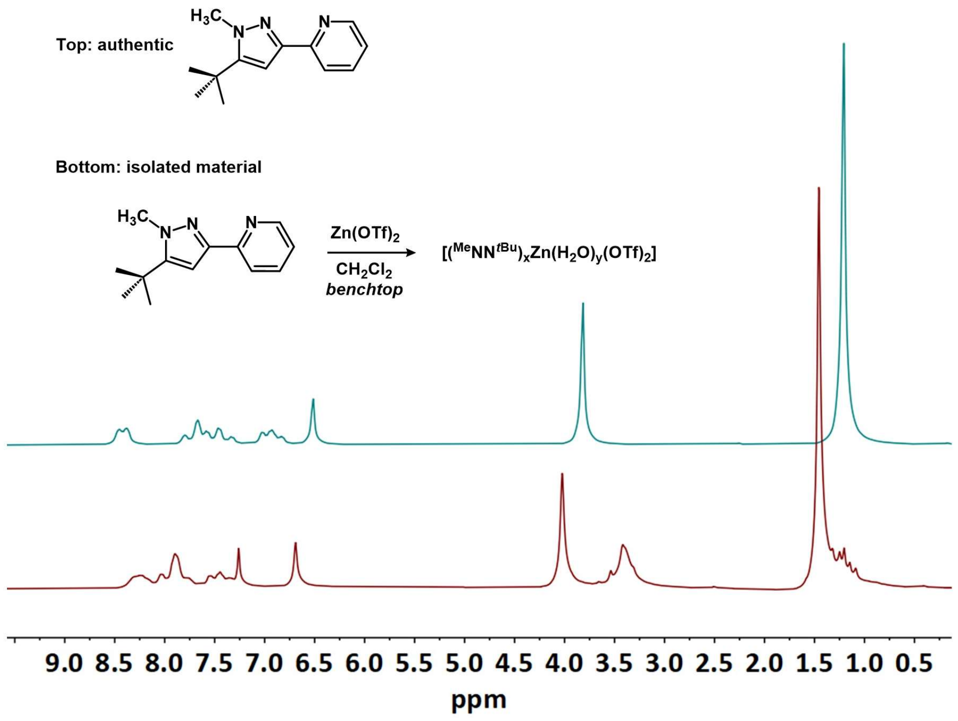

Figure S62 ^{1}H NMR comparison ($CDCl_3$, 25 °C, 60 MHz). Top: authentic sample of free ligand, $^{Me}NN^{tBu}$. Bottom: isolated material from benchtop reaction between $^{Me}NN^{tBu}$ and $Zn(OTf)_2$.

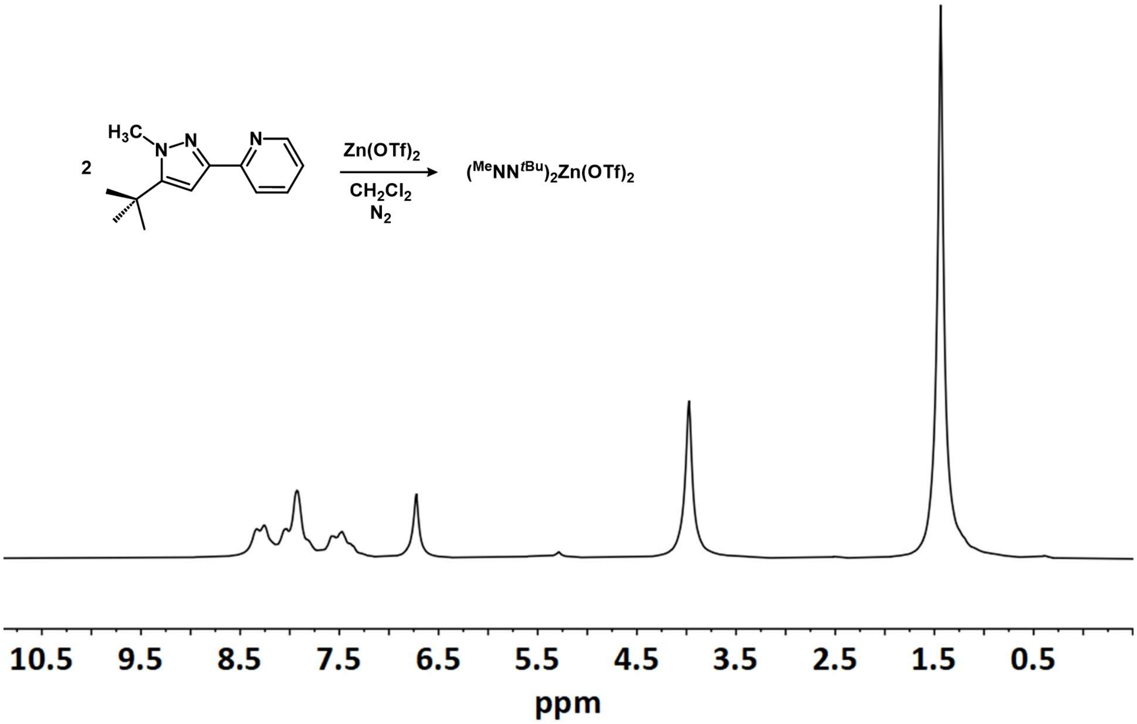

Figure S63 ^{1}H NMR ($CDCl_3$, 25 °C, 60 MHz) of $(^{Me}NN^{tBu})_2Zn(OTf)_2$ (**5’**) obtained under inert atmosphere.

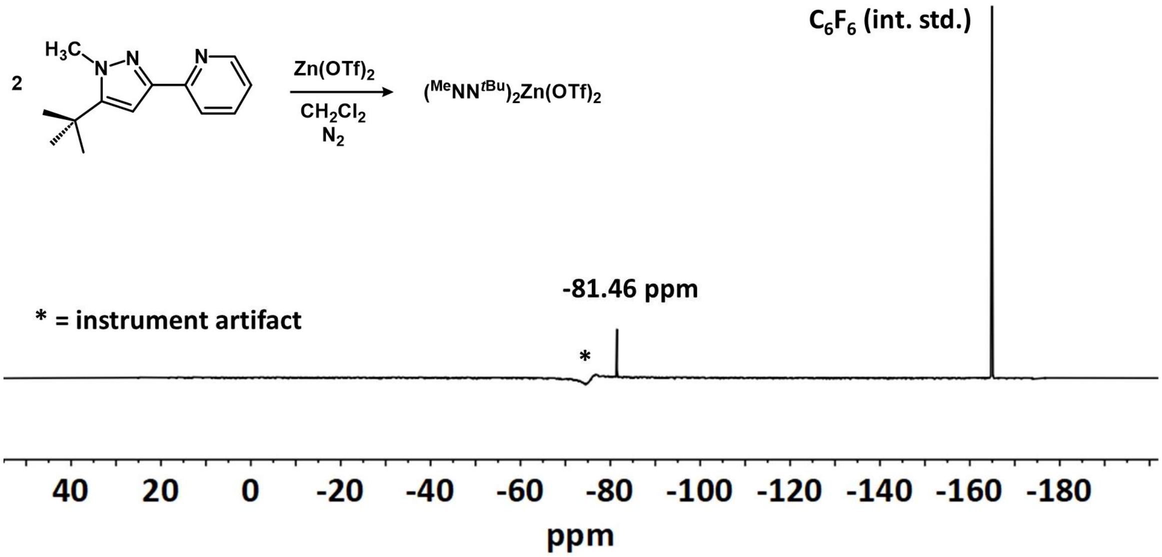

Figure S64 ^{19}F NMR spectrum ($CDCl_3$, 25 °C, 56 MHz) of $(^{Me}NN^{tBu})_2Zn(OTf)_2$ (**5’**).

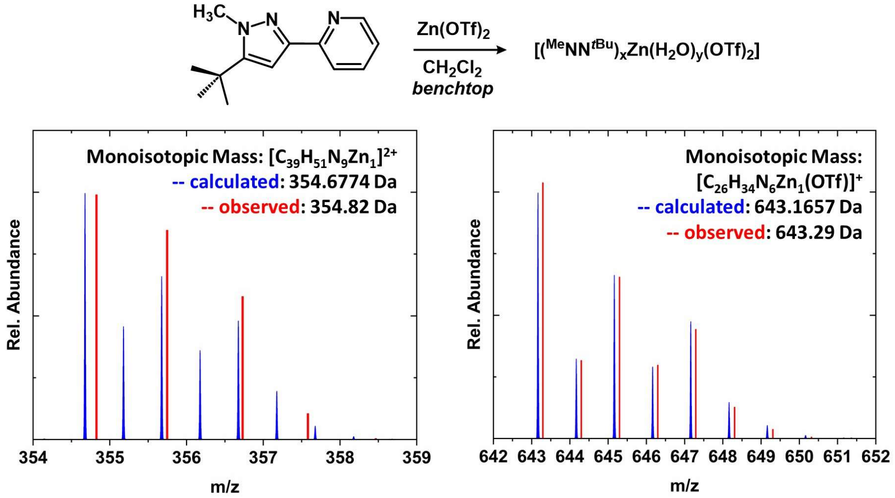

Figure S65 Mass spectrum obtained from attempted benchtop synthesis of $(^{Me}NN^{tBu})Zn(OTf)_2$. Left: $[(^{Me}NN^{tBu})_3Zn]^{2+}$. Right: $[(^{Me}NN^{tBu})_2Zn(OTf)]^{1+}$.

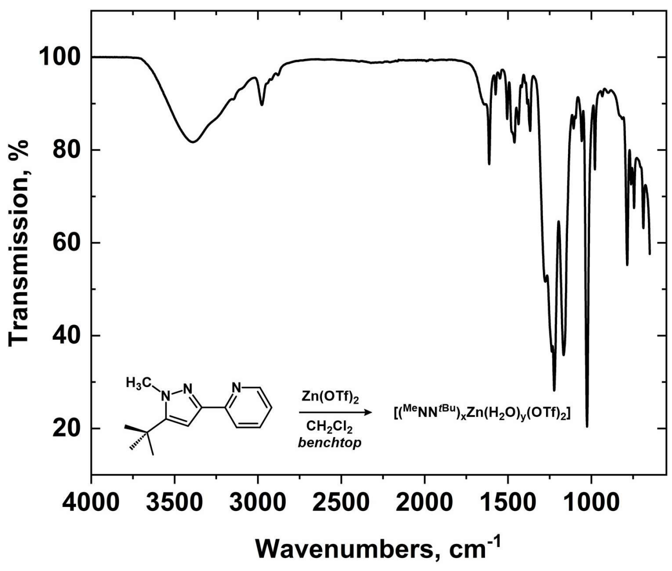

Figure S66 Infrared spectrum (ATR) obtained from attempted benchtop synthesis of ($^{Me}NN^{tBu}$)Zn(OTf)$_2$. The hygroscopic material displays a strong O-H absorption.

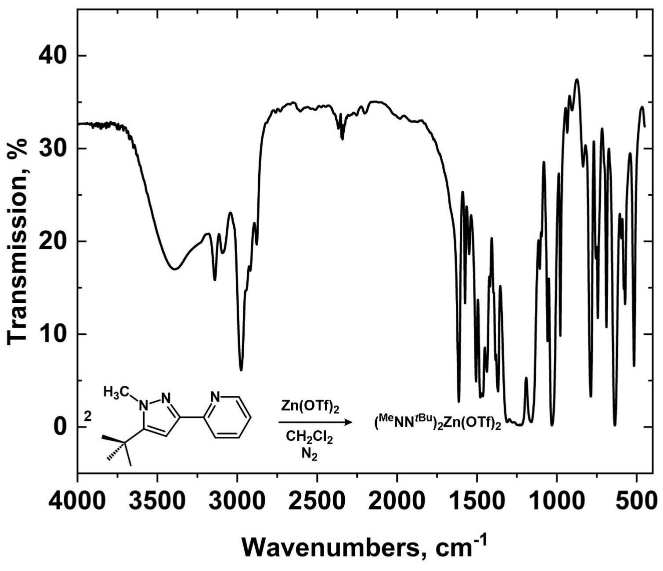

Figure S67 Infrared spectrum (KBr) of ($^{Me}NN^{tBu}$)$_2$Zn(OTf)$_2$ (**5'**) obtained under inert atmosphere.

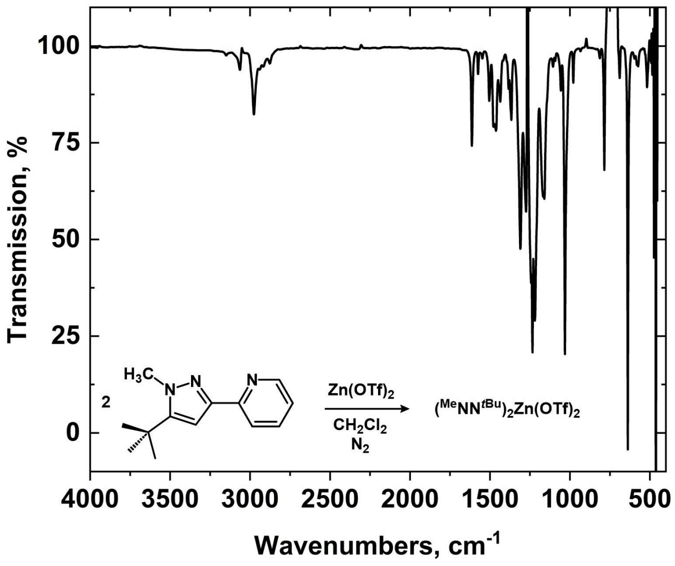

Figure S68 Infrared spectrum (CH_2Cl_2) of $(^{Me}NN^{tBu})_2Zn(OTf)_2$ (**5’**) obtained under inert atmosphere.

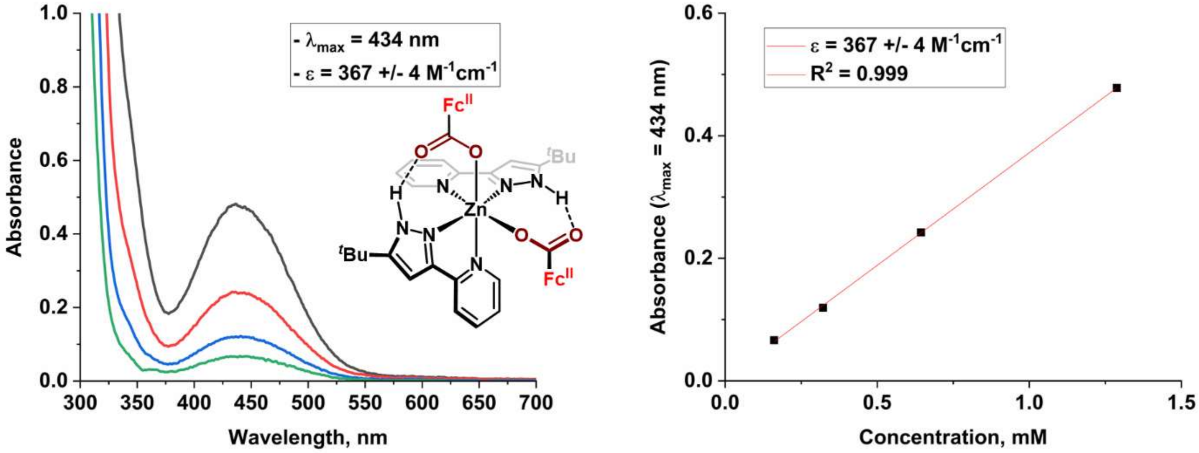

Figure S69 Electronic absorption spectra of $(^{H}NN^{tBu})_2Zn(O_2CFc)_2$ (**4**) in THF at room temperature. Concentration for Beer’s Law analysis: 0.161 mM, 0.323 mM, 0.645 mM, and 1.290 mM.

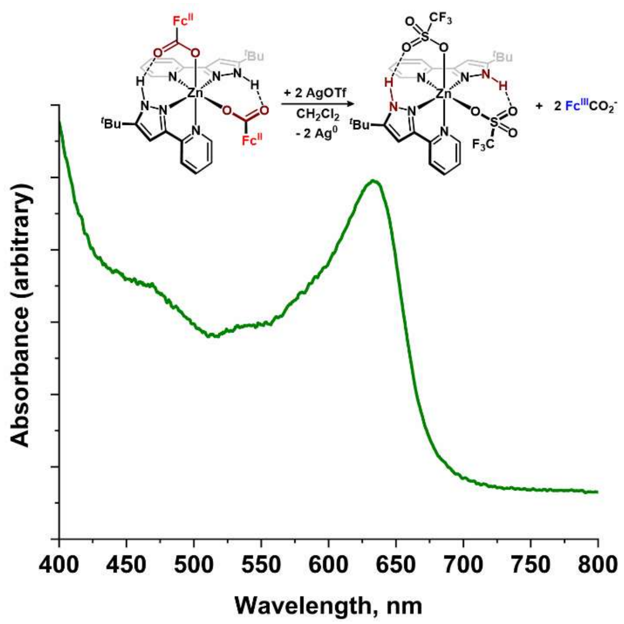

Figure S70 Electronic absorption spectrum of crude reaction between ($^{H}NN^{tBu}$)$_2$Zn(O_2CFc)$_2$ (**4**) and AgOTf in CH_2Cl_2 at room temperature. The absorption spectrum is consistent with literature reports for the formation of [$Fc^{III}CO_2$]$^{1-}$.[10]

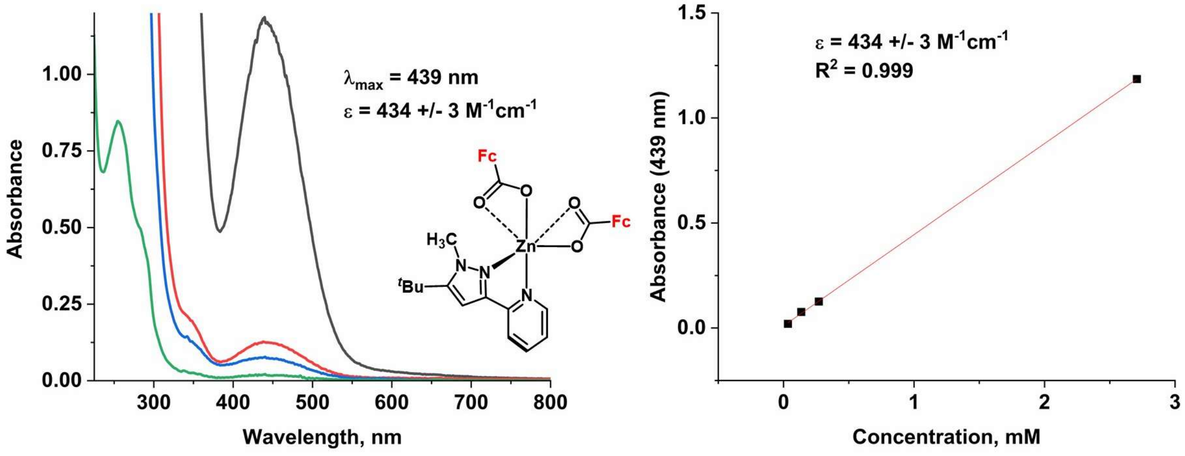

Figure S71 Electronic absorption spectra of ($^{Me}NN^{tBu}$)Zn(O_2CFc)$_2$ (**4'**) in THF at room temperature. Concentration for Beer's Law analysis: 2.707 mM, 0.271 mM, 0.135 mM, and 0.034 mM.

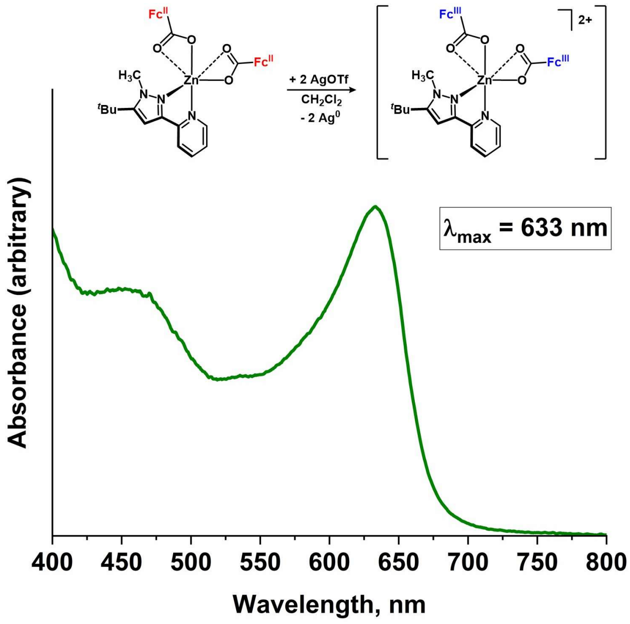

Figure S72 Electronic absorption spectrum of crude reaction between (^{Me}NN^{tBu})Zn($O_2CFc)_2$ (**4'**) and AgOTf in CH_2Cl_2 at room temperature. The absorption spectrum is consistent with literature reports for the formation of $[Fc^{III}CO_2]^{1-}$.[10]

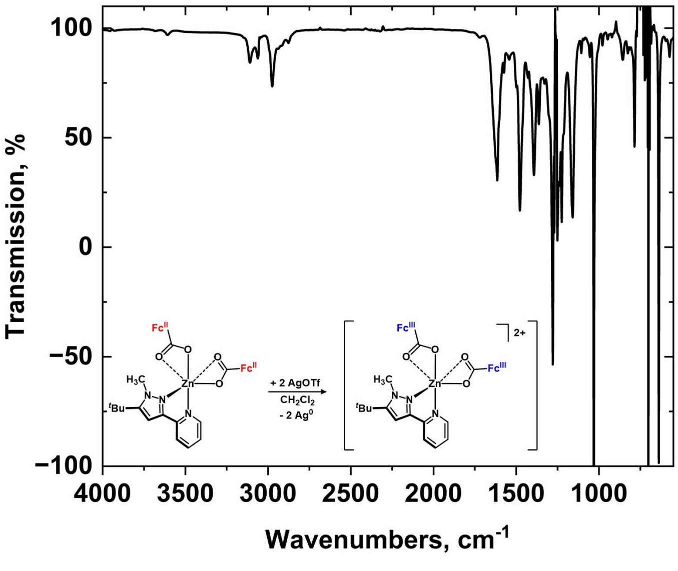

Figure S73 Infrared spectrum (CH_2Cl_2) of crude reaction between (^{Me}NN^{tBu})Zn($O_2CFc)_2$ (**4'**) and AgOTf. This spectrum was obtained under an inert atmosphere.

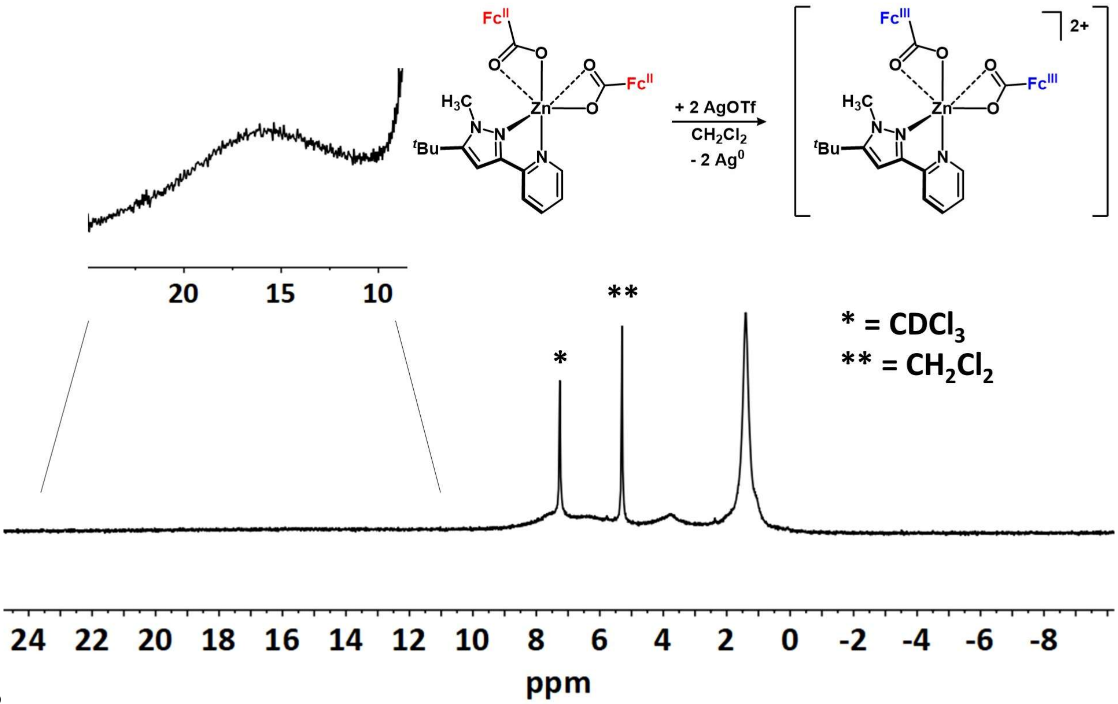

3

Figure S74 ^{1}H NMR spectrum ($CDCl_3$, 25 °C, 60 MHz) of crude reaction between ($^{Me}NN^{tBu}$)Zn(O_2CFc)$_2$ (**4’**) and AgOTf. This spectrum was obtained under an inert atmosphere.

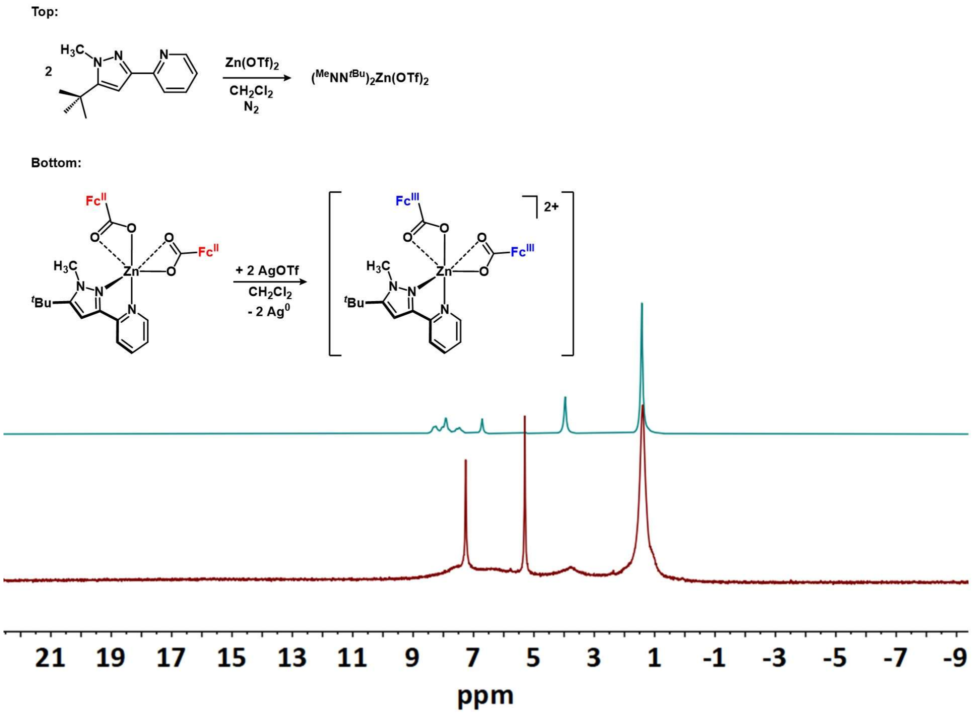

Figure S75 ^{1}H NMR comparison ($CDCl_3$, 25 °C, 60 MHz). Bottom: crude reaction between ($^{Me}NN^{tBu}$)Zn(O_2CFc)$_2$ (**4’**) and AgOTf. Top: Authentic sample of ($^{Me}NN^{tBu}$)$_2$Zn(OTf)$_2$ (**5’**). Both spectra were obtained under an inert atmosphere.

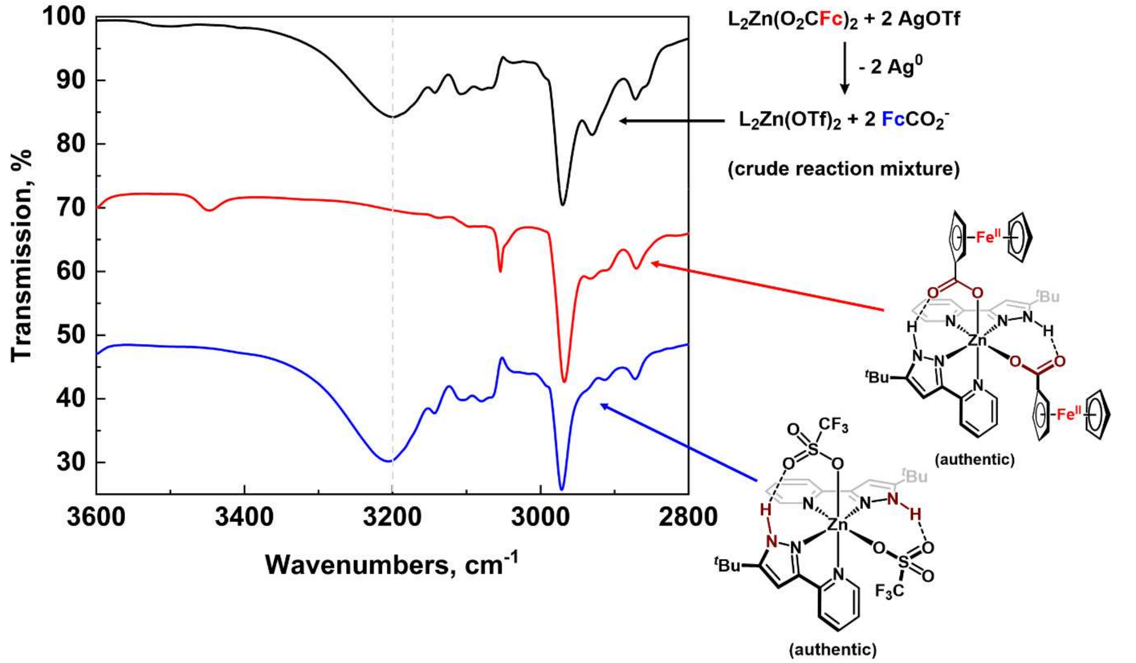

Figure S76 Overlay of infrared spectra (CH_2Cl_2, 25 °C). Top (black): crude reaction between $(^{H}NN^{tBu})_2Zn(O_2CFc)_2$ (**4**) and AgOTf (2 equiv.). Middle (red): authentic sample of $(^{H}NN^{tBu})_2Zn(O_2CFc)_2$ (**4**). Bottom (blue): authentic sample of $(^{H}NN^{tBu})_2Zn(OTf)_2$ (**5**).

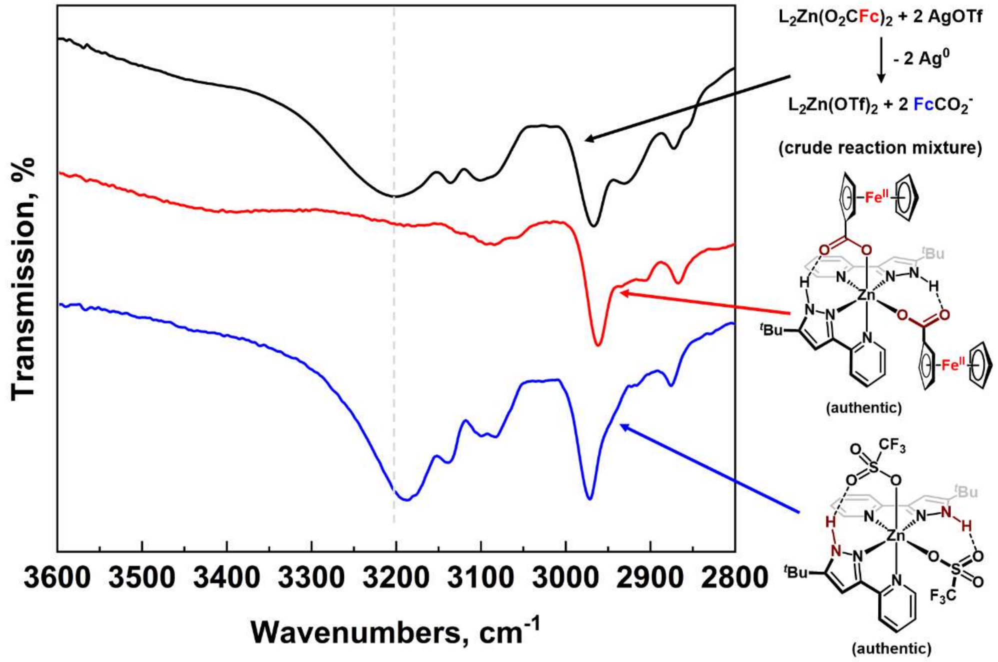

Figure S77 Overlay of infrared spectra. Top (black, KBr): crude reaction between $(^{H}NN^{tBu})_2Zn(O_2CFc)_2$ (**4**) and AgOTf (2 equiv.). Middle (red, ATR): authentic sample of $(^{H}NN^{tBu})_2Zn(O_2CFc)_2$ (**4**). Bottom (blue, ATR): authentic sample of $(^{H}NN^{tBu})_2Zn(OTf)_2$ (**5**).

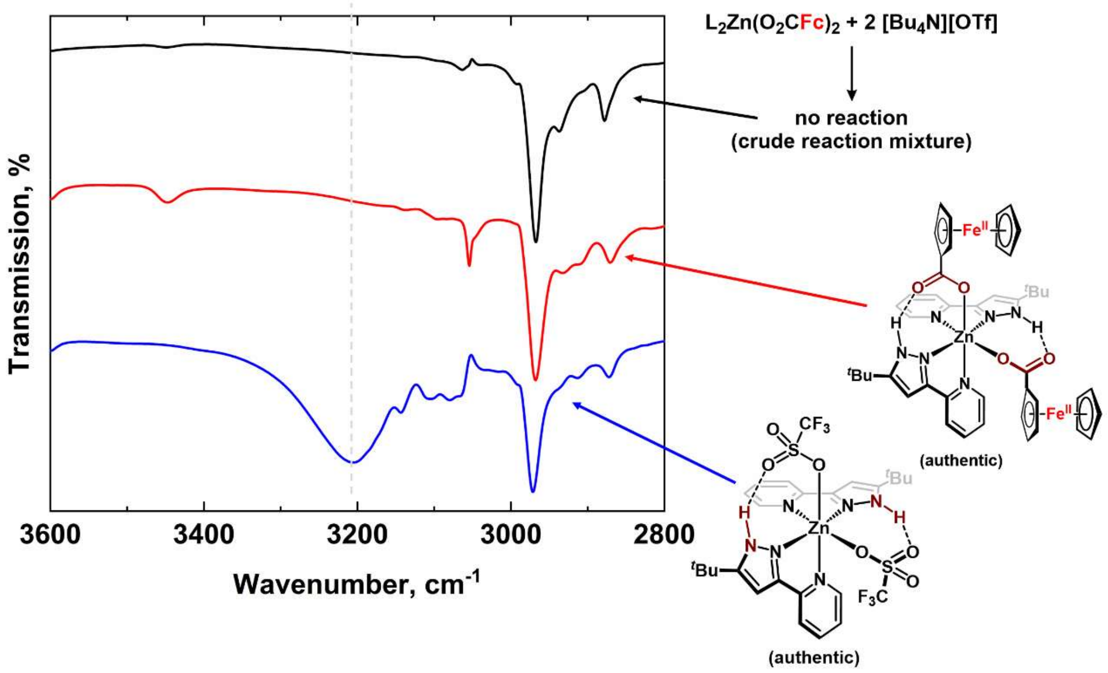

Figure S78 Overlay of infrared spectra (CH_2Cl_2, 25 °C). Top (black): crude reaction between ($^{H}NN^{tBu}$)$_2$Zn(O_2CFc)$_2$ (**4**) and [Bu_4N][OTf] (2 equiv.). Middle (red): authentic sample of ($^{H}NN^{tBu}$)$_2$Zn(O_2CFc)$_2$ (**4**). Bottom (blue): authentic sample of ($^{H}NN^{tBu}$)$_2$Zn(OTf)$_2$ (**5**).

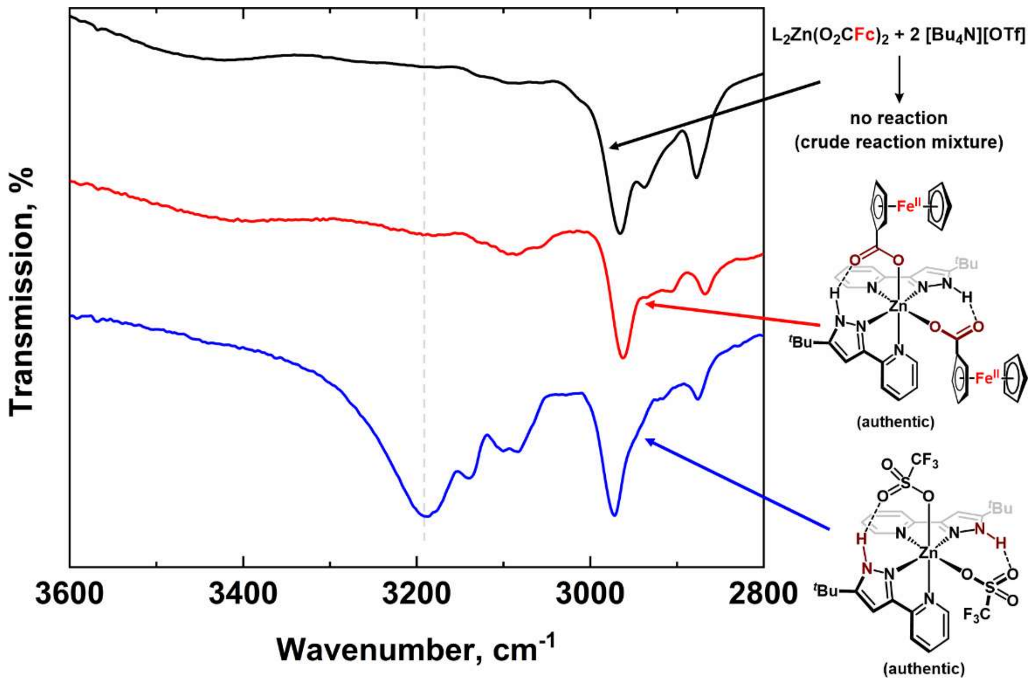

Figure S79 Overlay of infrared spectra. Top (black, KBr): crude reaction between ($^{H}NN^{tBu}$)$_2$Zn(O_2CFc)$_2$ (**4**) and [Bu_4N][OTf] (2 equiv.). Middle (red, ATR): authentic sample of ($^{H}NN^{tBu}$)$_2$Zn(O_2CFc)$_2$ (**4**). Bottom (blue, ATR): authentic sample of ($^{H}NN^{tBu}$)$_2$Zn(OTf)$_2$ (**5**).

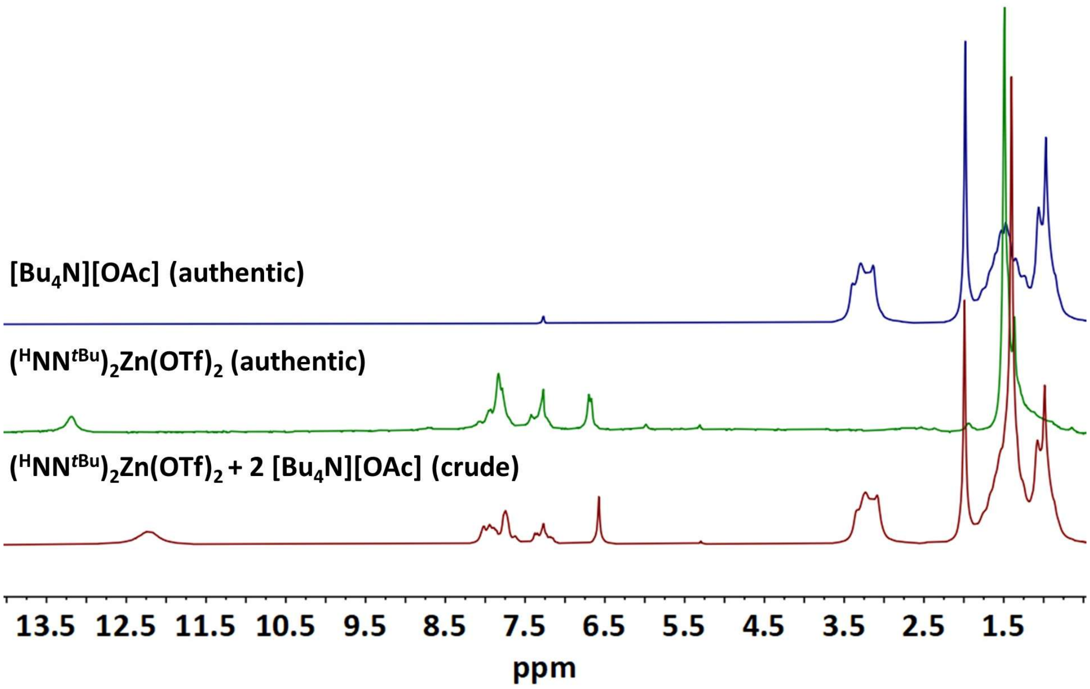

Figure S80 Crude ^{1}H NMR spectra ($CDCl_3$, 25 °C, 60 MHz). Bottom: reaction between $(^{H}NN^{tBu})_2Zn(OTf)_2$ (**5**) and two equiv. $[Bu_4N][OAc]$. Middle and top: authentic spectra of $(^{H}NN^{tBu})_2Zn(OTf)_2$ (**5**) and $[Bu_4N][OAc]$, respectively.

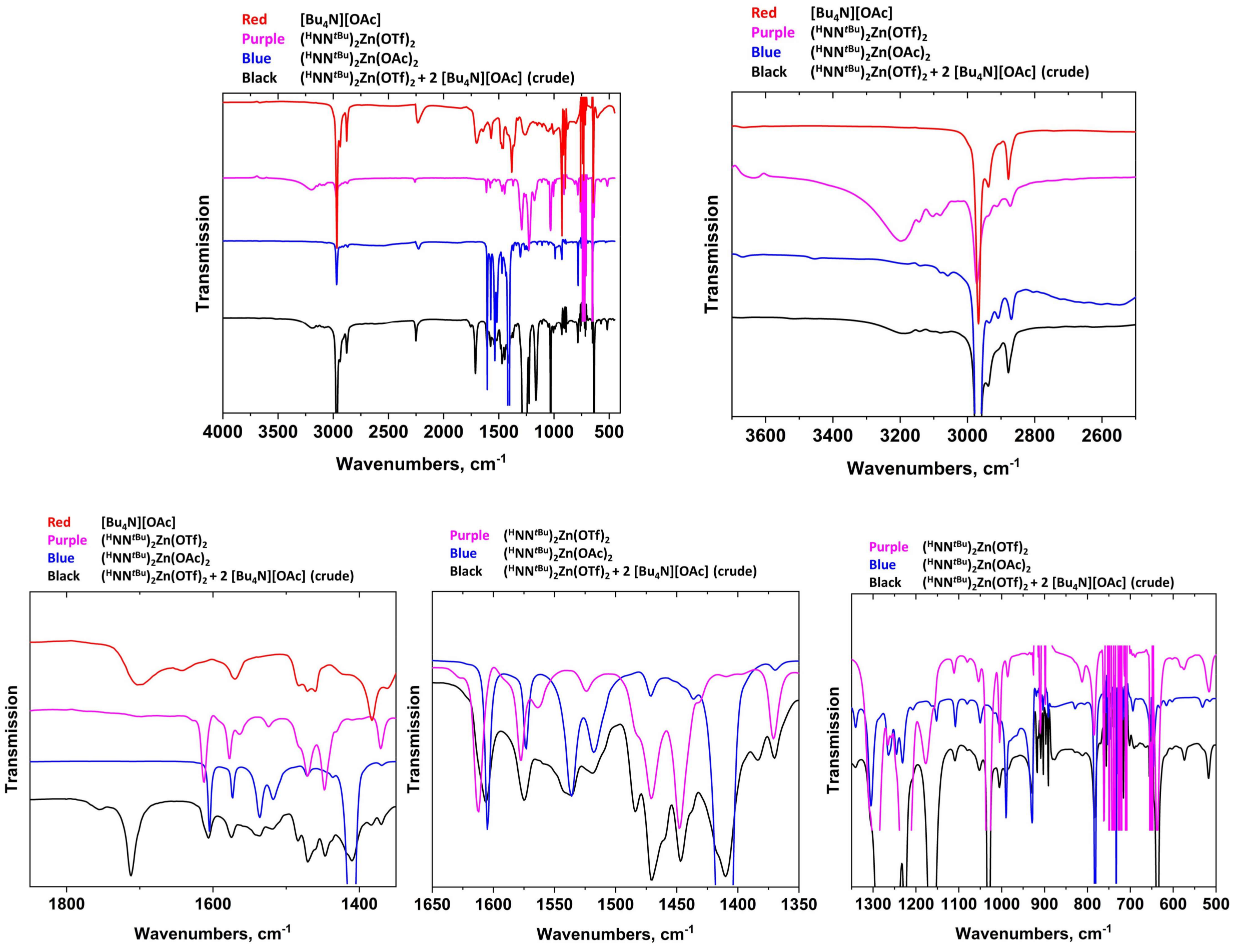

Figure S81 Overlay of infrared spectra ($CDCl_3$, 25 °C) highlighting different regions. Black: crude reaction between ($^{H}NN^{tBu}$)$_2$Zn(OTf)$_2$ (**5**) and [Bu$_4$N][OAc] (2 equiv.). Blue: authentic sample of ($^{H}NN^{tBu}$)$_2$Zn(OAc)$_2$ (**3**). Purple: authentic sample of ($^{H}NN^{tBu}$)$_2$Zn(OTf)$_2$ (**5**). Red: authentic sample of [Bu$_4$N][OTf].

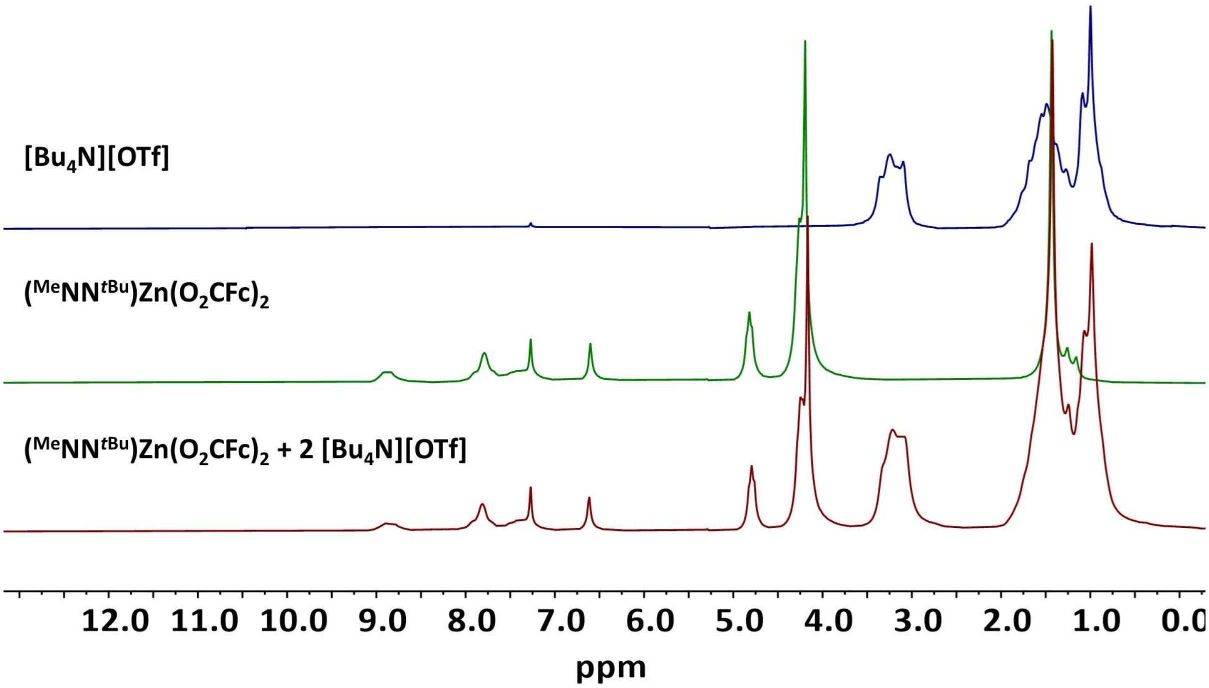

Figure S82 Crude ^{1}H NMR spectra (CDCl$_3$, 25 °C, 60 MHz). Bottom: reaction between (^{Me}NN^{tBu})Zn(O$_2$CFc)$_2$ (**4'**) and two equiv. [Bu$_4$N][OTf]. No reaction is observed. Middle and top: authentic spectra of (^{Me}NN^{tBu})Zn(O$_2$CFc)$_2$ (**4'**) and [Bu$_4$N][OTf], respectively.

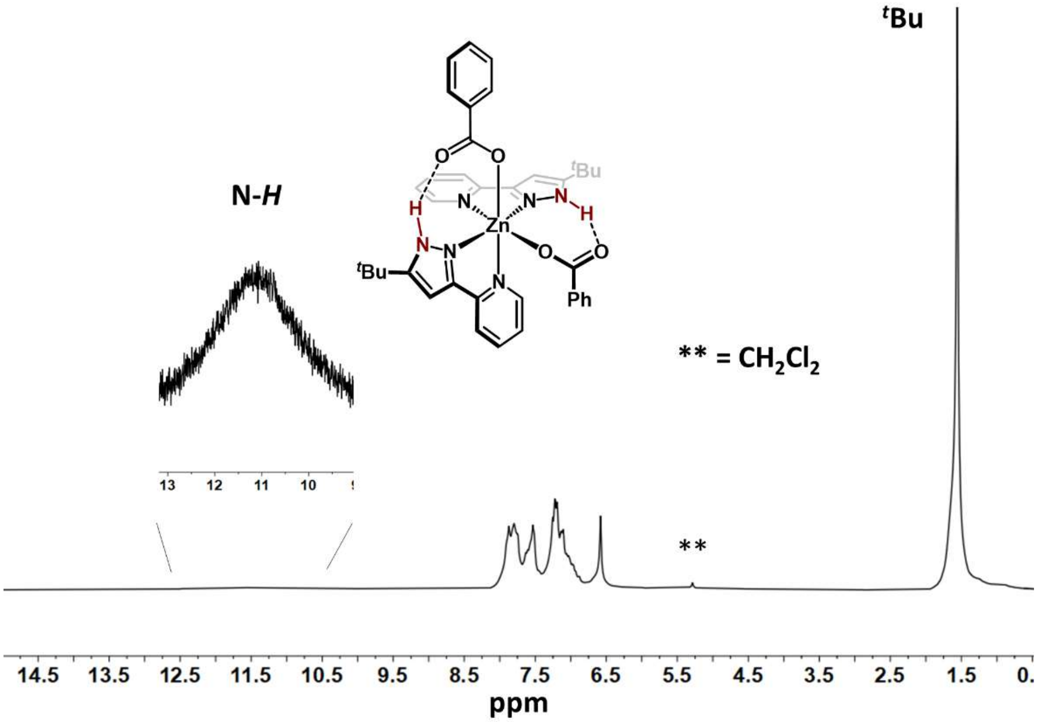

Figure S83 ^{1}H NMR spectrum (CDCl$_3$, 25 °C, 60 MHz) of (^{H}NN^{tBu})$_2$Zn(O$_2$CPh)$_2$ (**6-H**).

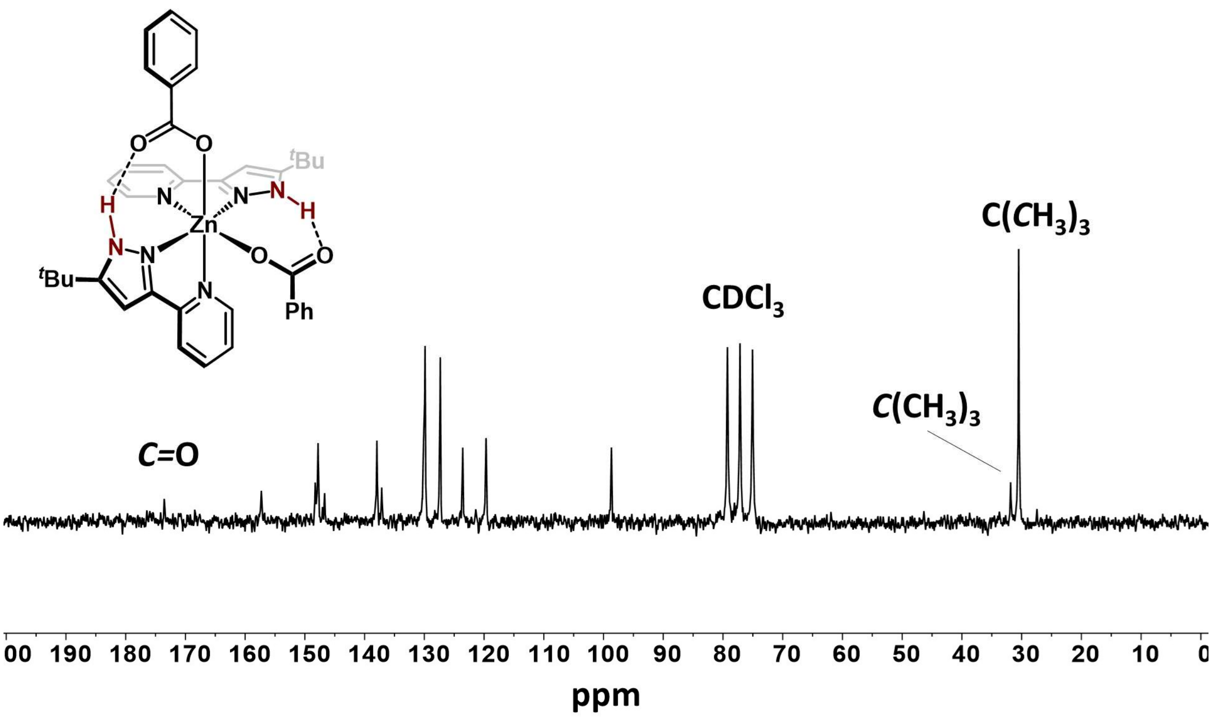

Figure S84 $^{13}C\{^{1}H\}$ NMR spectrum ($CDCl_3$, 25 °C, 15 MHz) of $(^{H}NN^{tBu})_2Zn(O_2CPh)_2$ (**6-H**).

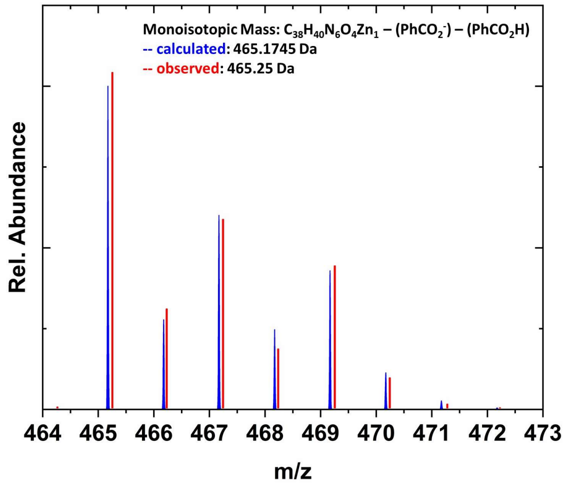

Figure S85 Mass spectrum of $(^{H}NN^{tBu})_2Zn(O_2CPh)_2$ (**6-H**): M - ($ArCO_2^-$ + $ArCO_2H$).

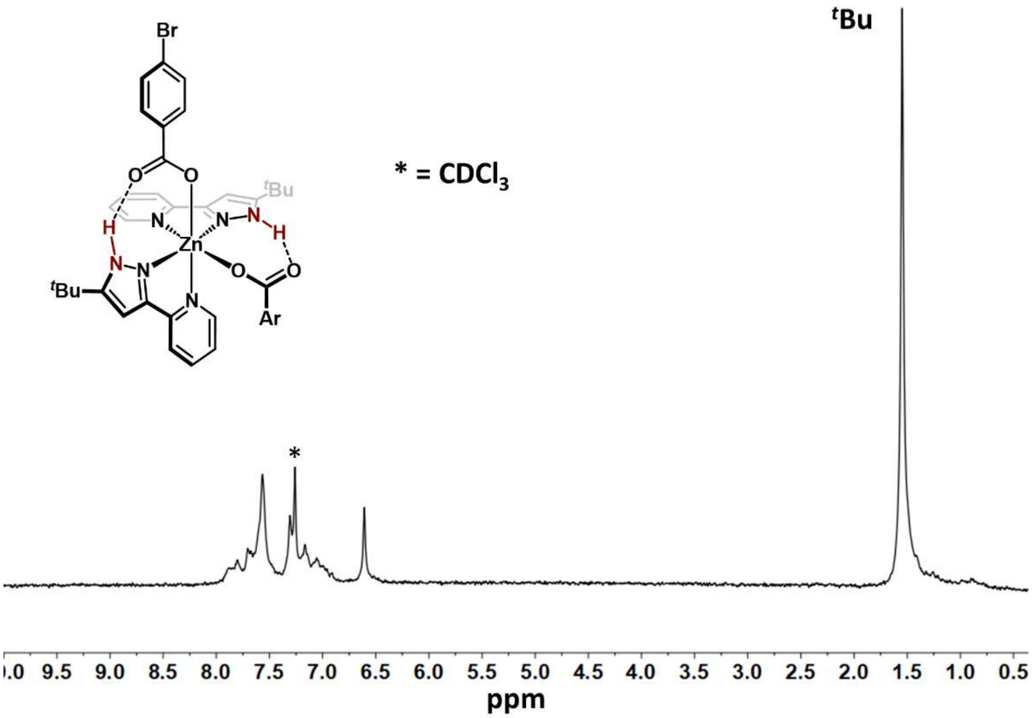

Figure S86 ^{1}H NMR spectrum ($CDCl_3$, 25 °C, 60 MHz) of $(^{H}NN^{tBu})_2Zn(O_2CAr)_2$ (**6-Br**; Ar = *p*-C_6H_4Br).

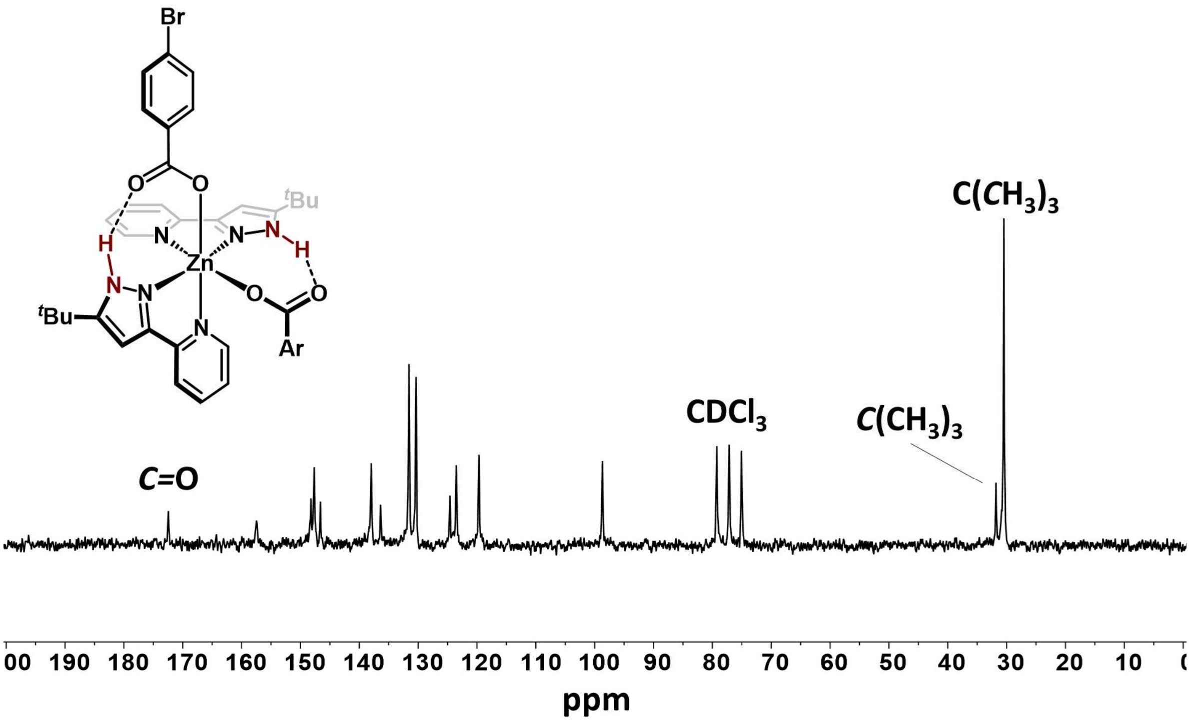

Figure S87 $^{13}C\{^{1}H\}$ NMR spectrum ($CDCl_3$, 25 °C, 15 MHz) of $(^{H}NN^{tBu})_2Zn(O_2CAr)_2$ (**6-Br**; Ar = *p*-C_6H_4Br).

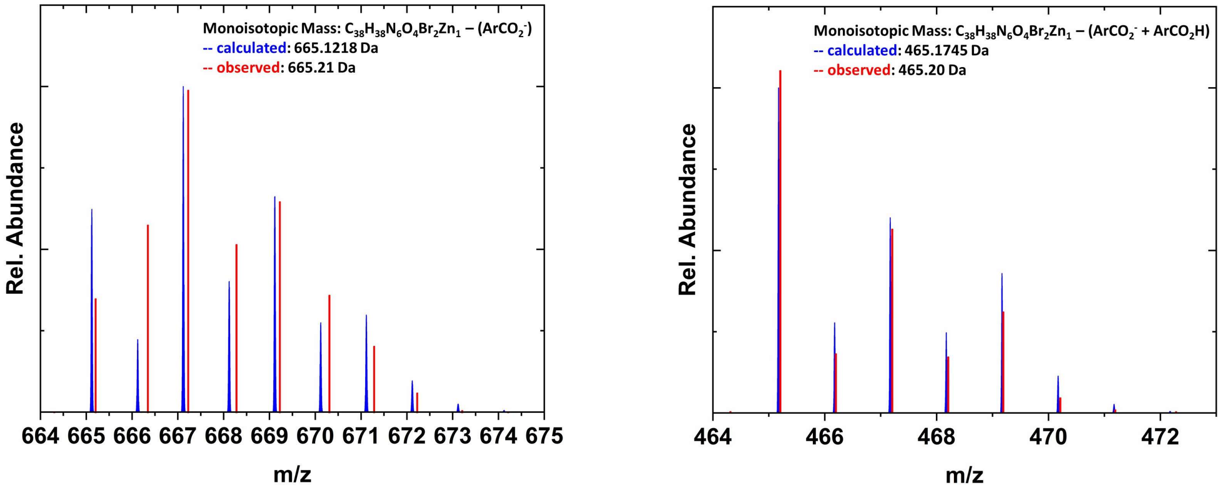

Figure S88 Mass spectrum of $(^{H}NN^{tBu})_2Zn(O_2CAr)_2$ (**6-Br**; Ar = *p*-C_6H_4Br). Left: M - $ArCO_2^-$; Right: M - ($ArCO_2^-$ + $ArCO_2H$).

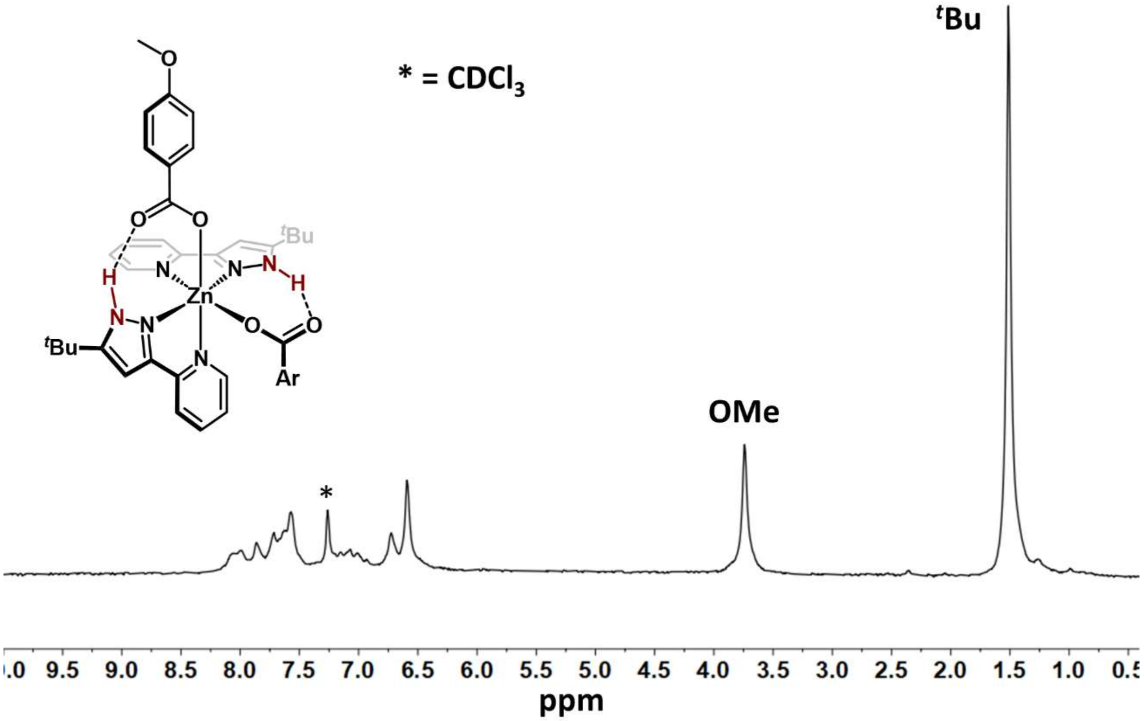

Figure S89 ^1H NMR spectrum ($CDCl_3$, 25 °C, 60 MHz) of $(^{H}NN^{tBu})_2Zn(O_2CAr)_2$ (**6-OMe**; Ar = *p*-C_6H_4OMe).

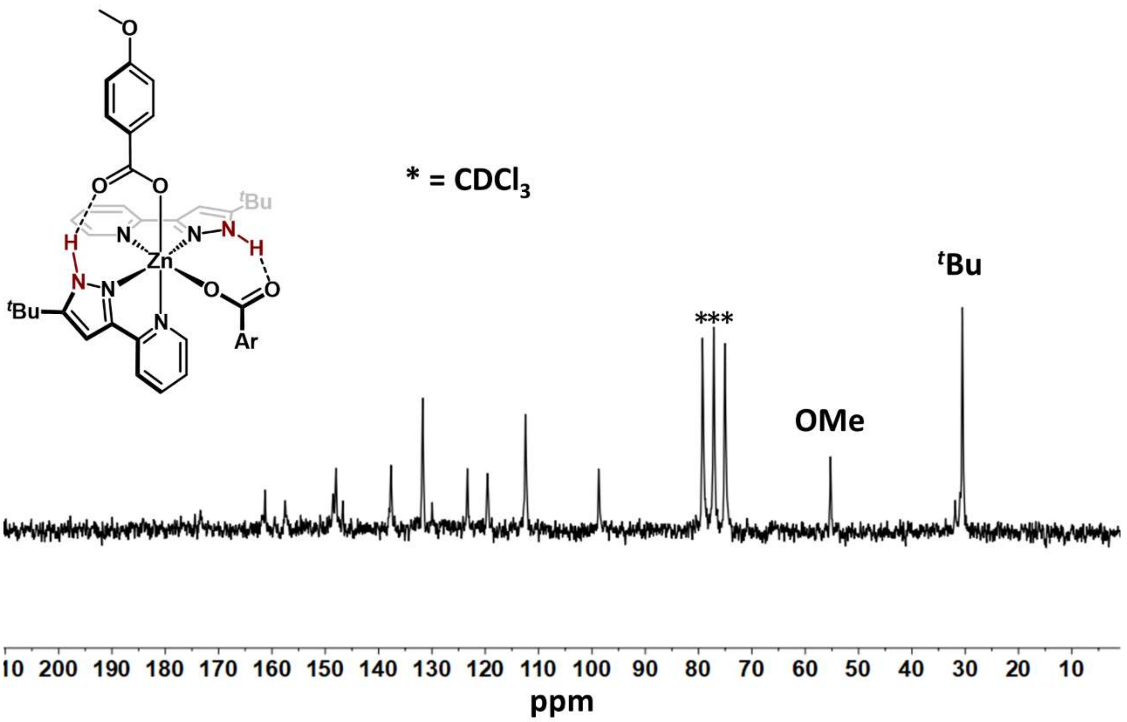

Figure S90 $^{13}C\{^1H\}$ NMR spectrum ($CDCl_3$, 25 °C, 15 MHz) of $(^{H}NN^{tBu})_2Zn(O_2CAr)_2$ (**6-OMe**; Ar = *p*-C_6H_4OMe).

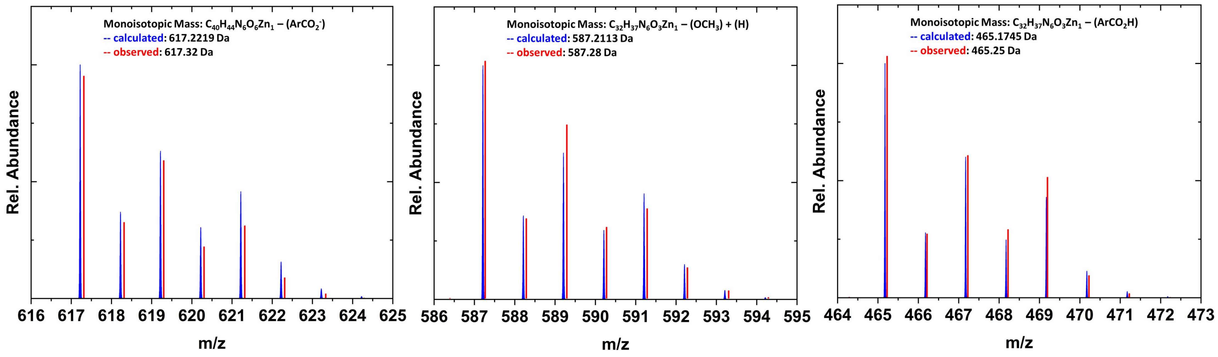

Figure S91 Mass spectrum of $(^{H}NN^{tBu})_2Zn(O_2CAr)_2$ (**6-OMe**; Ar = *p*-C_6H_4OMe). Left: M - $ArCO_2^-$; Middle: M - ($ArCO_2^-$ + OCH_3) + H; Right: M - ($ArCO_2^-$ + $ArCO_2H$).

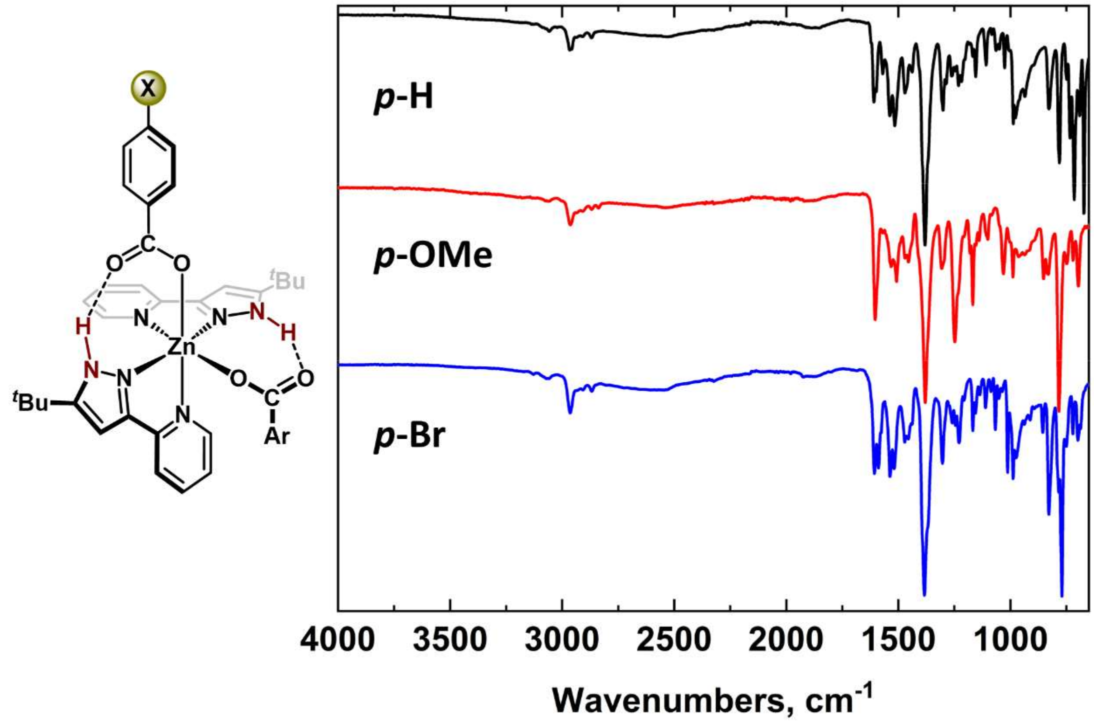

Figure S92 Infrared spectra (ATR, ambient temperature) of $(^{H}NN^{tBu})_2Zn(O_2CAr)_2$ (**6-X**; Ar = *p*-C_6H_4X; black, X = H; red, X = OMe; blue, X = Br).

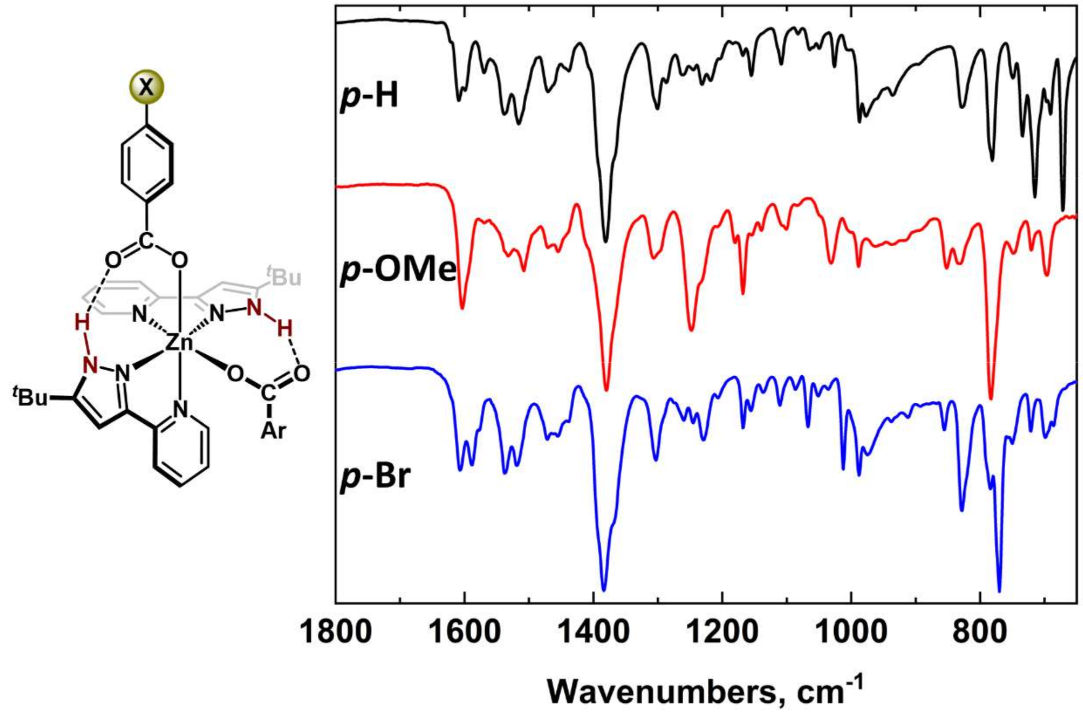

Figure S93 Infrared spectra (ATR, ambient temperature) of $(^{H}NN^{tBu})_2Zn(O_2CAr)_2$ (**6-X**; Ar = *p*-C_6H_4X; black, X = H; red, X = OMe; blue, X = Br) emphasizing the lower energy region.

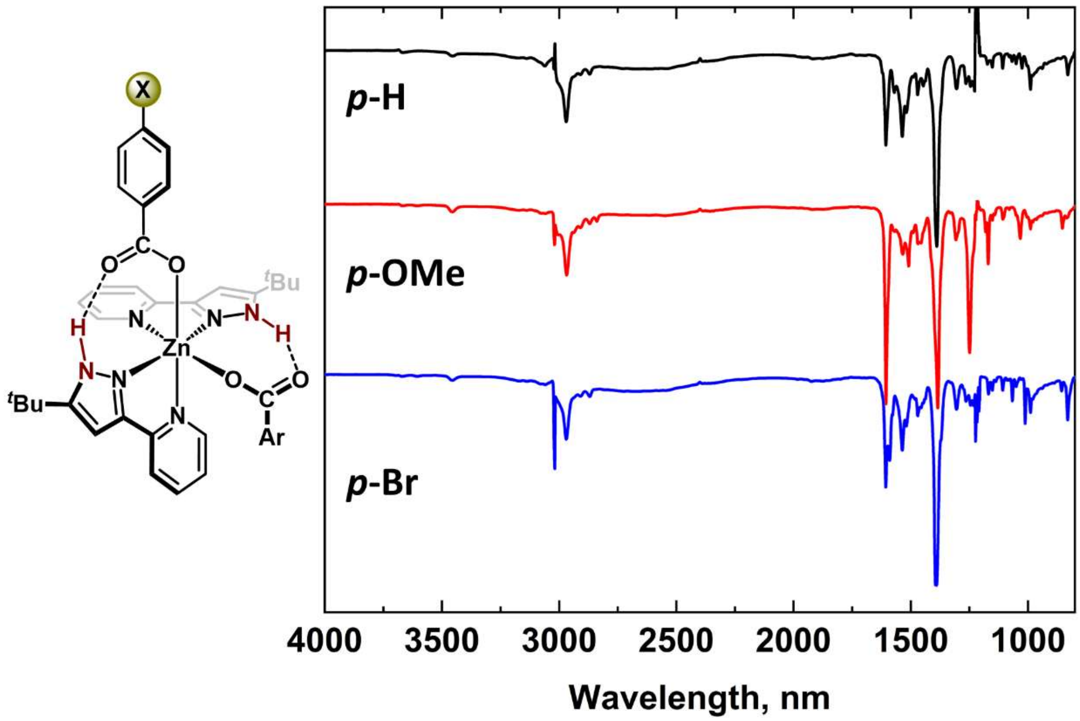

Figure S94 Infrared spectra (CH_2Cl_2, ambient temperature) of $(^{H}NN^{tBu})_2Zn(O_2CAr)_2$ (**6-X**; Ar = *p*-C_6H_4X; black, X = H; red, X = OMe; blue, X = Br).

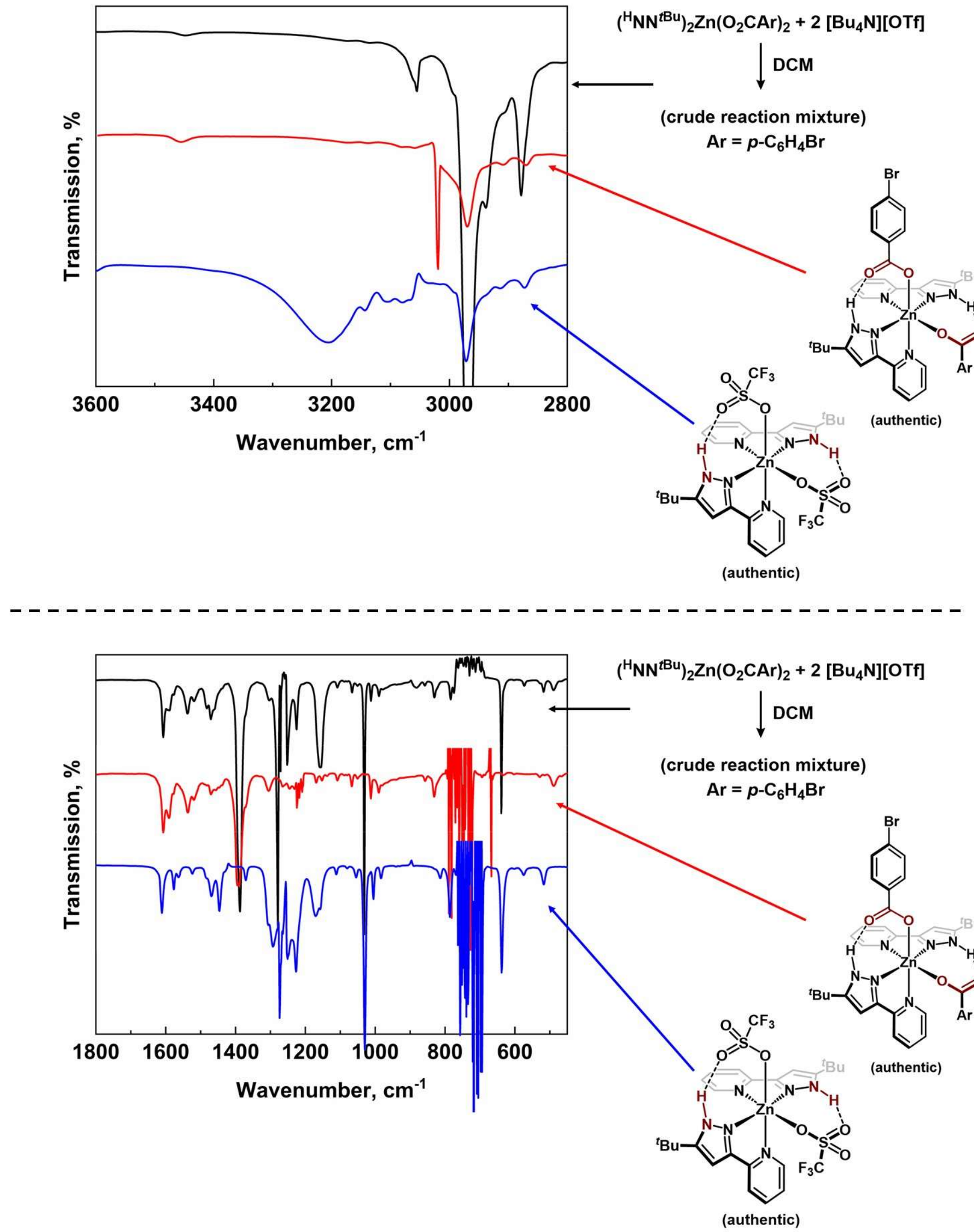

Figure S95 Overlay of infrared spectra (CH_2Cl_2, 25 °C). Top: high energy region. Bottom: low energy region. Black: crude reaction between $(^{H}NN^{tBu})_2Zn(O_2CAr)_2$ (**6-Br**) and $[Bu_4N][OTf]$ (2 equiv.). Middle (red): authentic sample of $(^{H}NN^{tBu})_2Zn(O_2CAr)_2$ (**6-Br**). Bottom (blue): authentic sample of $(^{H}NN^{tBu})_2Zn(OTf)_2$ (**5**).

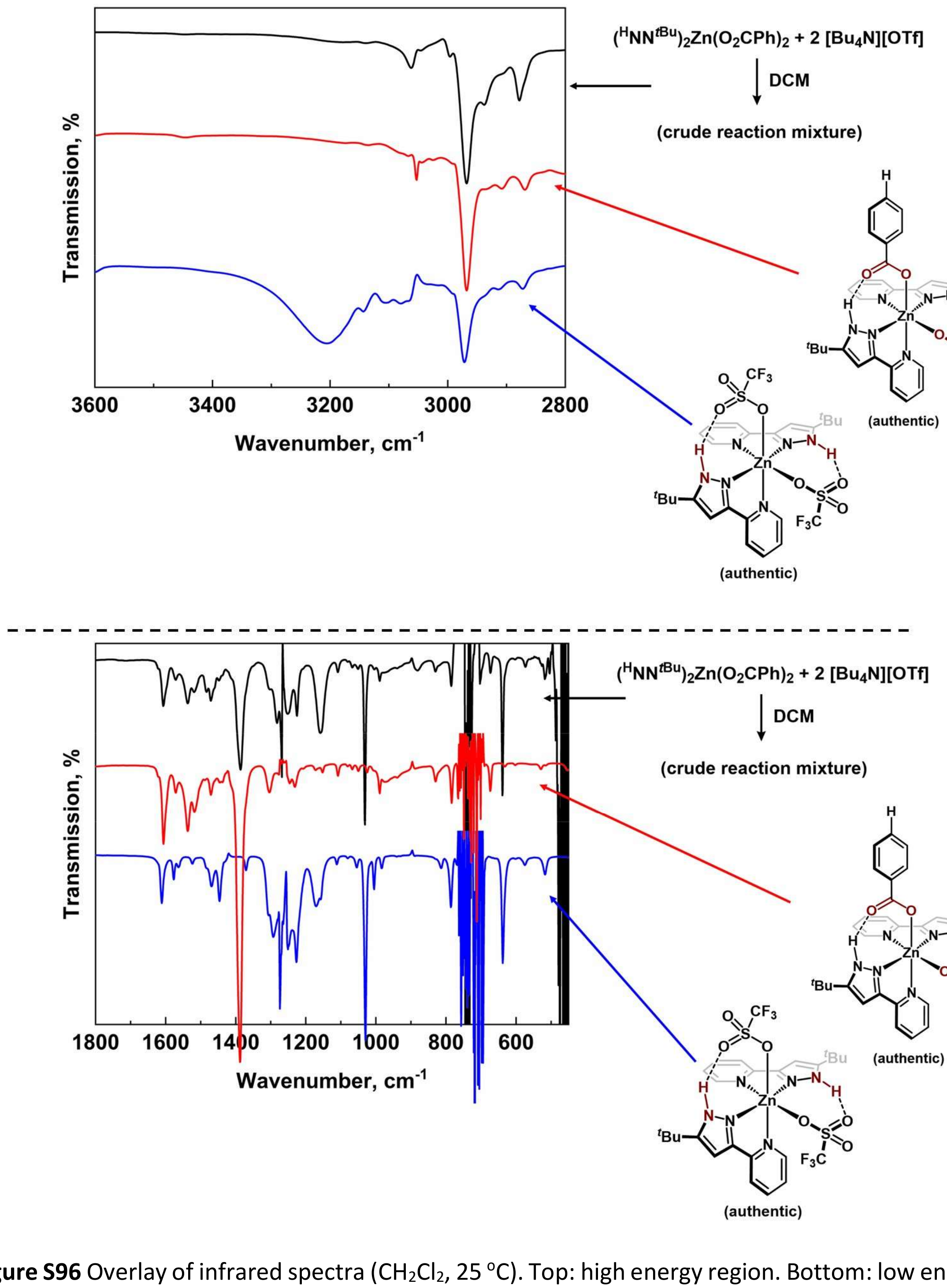

Figure S96 Overlay of infrared spectra (CH_2Cl_2, 25 °C). Top: high energy region. Bottom: low energy region. Black: crude reaction between $(^{H}NN^{tBu})_2Zn(O_2CAr)_2$ (**6-H**) and $[Bu_4N][OTf]$ (2 equiv.). Middle (red): authentic sample of $(^{H}NN^{tBu})_2Zn(O_2CAr)_2$ (**6-H**). Bottom (blue): authentic sample of $(^{H}NN^{tBu})_2Zn(OTf)_2$ (**5**).

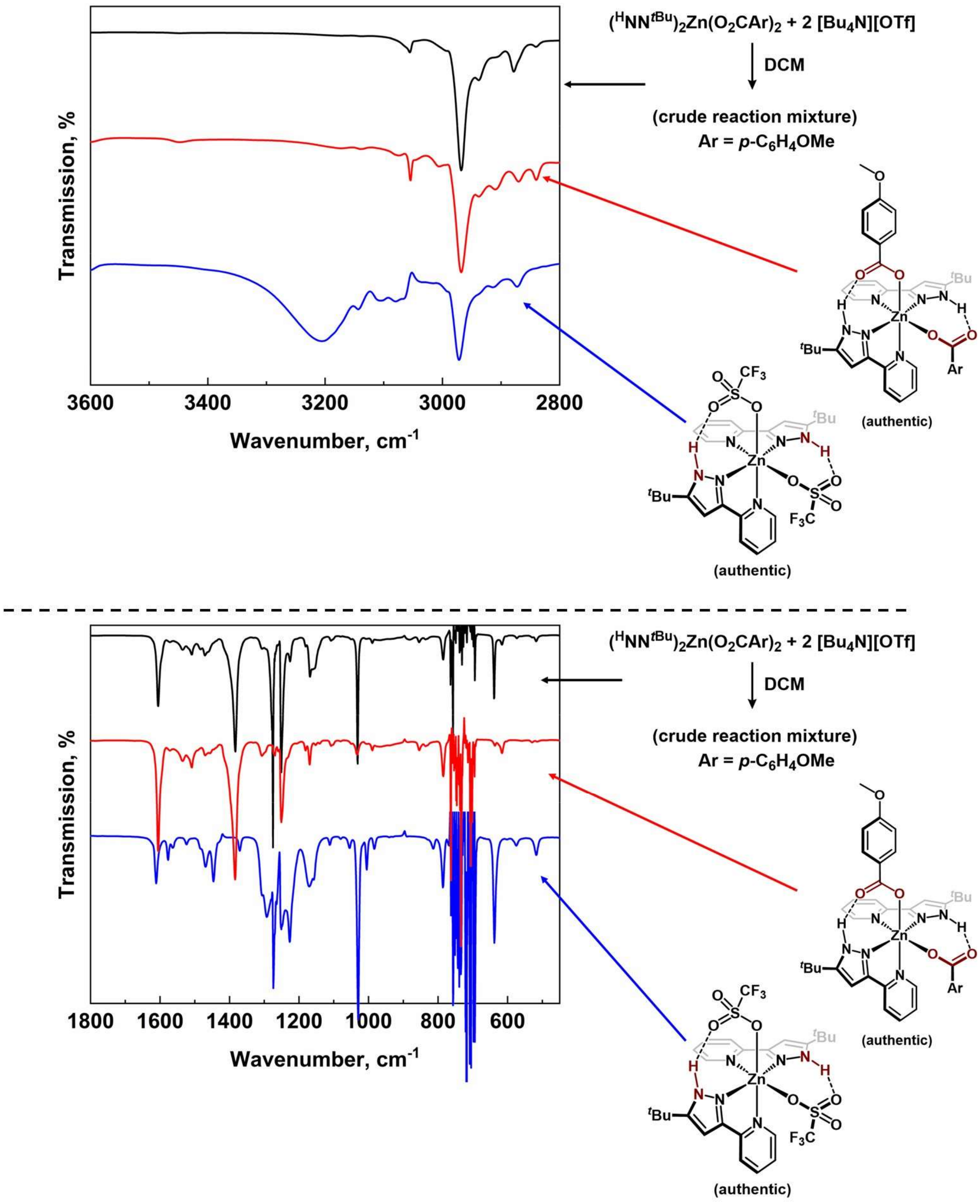

Figure S97 Overlay of infrared spectra (CH_2Cl_2, 25 °C). Top: high energy region. Bottom: low energy region. Black: crude reaction between $(^{H}NN^{tBu})_2Zn(O_2CAr)_2$ (**6-OMe**) and $[Bu_4N][OTf]$ (2 equiv.). Middle (red): authentic sample of $(^{H}NN^{tBu})_2Zn(O_2CAr)_2$ (**6-OMe**). Bottom (blue): authentic sample of $(^{H}NN^{tBu})_2Zn(OTf)_2$ (**5**).

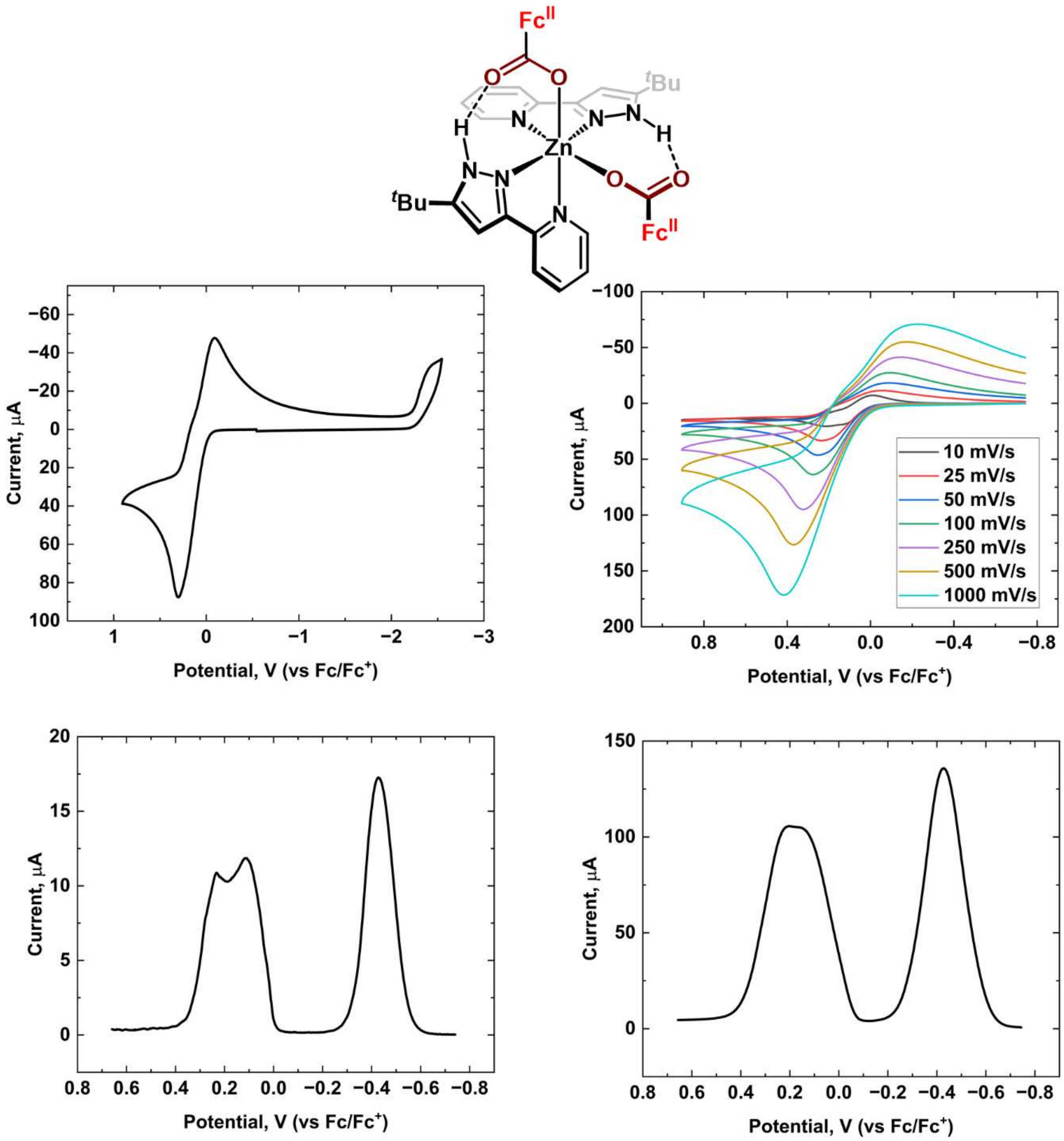

Figure S98 Electrochemical data for ($^{H}NN^{tBu}$)$_2$Zn(O_2CFc)$_2$ (**4**; 3.2 mM). All experiments were performed in THF under an N_2 atmosphere with 0.2 M [Bu_4N][PF_6] supporting electrolyte. Working electrode: glassy carbon. Counter electrode: Pt wire. Reference electrode: Ag wire. Top left: Cyclic voltammogram scanning anodically from -540 mV (initial) at 200 mV/s. Top right: scan rate dependence. Bottom left: differential pulse voltammogram. The redox event at -427 mV is the decamethylferrocene internal standard. Bottom right: square wave voltammogram. The redox event at -427 mV is the decamethylferrocene internal standard. At 200 mV/s: decamethylferrocene ΔE_p = 250 mV; **4** ΔE_p = 410 mV.

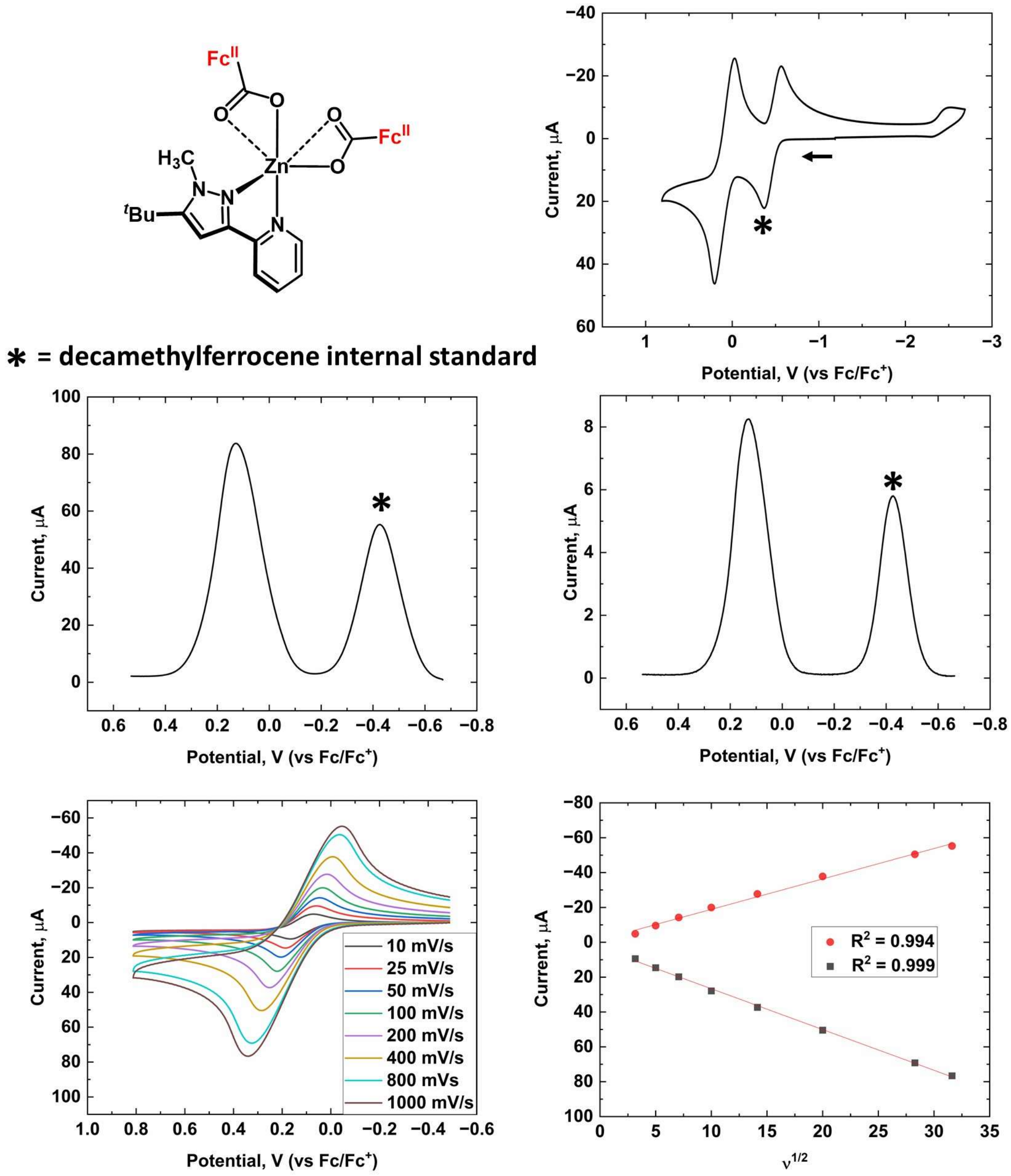

Figure S99 Electrochemical data for ($^{Me}NN^{tBu}$)Zn(O_2CFc)$_2$ (**4'**; 0.9 mM). All experiments were performed in THF under an N_2 atmosphere with 0.2 M [Bu_4N][PF_6] supporting electrolyte. Working electrode: glassy carbon. Counter electrode: Pt wire. Reference electrode: Ag wire. Top right: Cyclic voltammogram scanning anodically from -1.1 V (initial) at 200 mV/s. The reversible redox event at -427 mV is the decamethylferrocene internal standard. Middle left: square wave voltammogram. The redox event at -427 mV is the decamethylferrocene internal standard. Middle right: Differential pulse voltammogram. The redox event at -427 mV is the decamethylferrocene internal standard. Bottom left: scan rate dependence. Bottom right: plot of current vs square root of the scan rate. At 200 mV/s: decamethylferrocene ΔE_p = 200 mV; **4'** ΔE_p = 230 mV.

Compound: $(^{H}NN^{tBu})ZnBr_2$ (**1-Br**)

Local Name: JK-1-32

CCDC Number: 2395151

Table S1. Crystallographic details for $(^{H}NN^{tBu})ZnBr_2$ (**1-Br**)

**Crystal data**	
Chemical formula	$C_{12}H_{15}Br_2N_3Zn$
M_r	426.46
Crystal system, space group	Monoclinic, $P2_1/c$
Temperature (K)	150
a, *b*, *c* (Å)	26.434 (7), 11.094 (3), 15.654 (4)
β (°)	98.197 (10)
V (Å^3)	4544 (2)
Z	12
Radiation type	Mo *K*α
μ (mm^{-1})	6.88
Crystal size (mm)	0.32 × 0.28 × 0.22
**Data collection**	
Diffractometer	Bruker AXS D8 Quest diffractometer with PhotonII charge-integrating pixel array detector (CPAD)
Absorption correction	Multi-scan *SADABS* 2016/2: Krause, L., Herbst-Irmer, R., Sheldrick G.M. & Stalke D. (2015). J. Appl. Cryst. 48, 3-10.
T_{min}, T_{max}	0.592, 0.746
No. of measured, independent and observed [$I > 2\sigma(I)$] reflections	90367, 11281, 9473
R_{int}	0.038
$(\sin \theta/\lambda)_{max}$ (Å^{-1})	0.667
**Refinement**	
$R[F^2 > 2\sigma(F^2)]$, $wR(F^2)$, S	0.047, 0.120, 1.04
No. of reflections	11281
No. of parameters	536
No. of restraints	120
H-atom treatment	H-atom parameters constrained
	$w = 1/[s^2(F_o^2) + (0.0444P)^2 + 23.0632P]$ where $P = (F_o^2 + 2F_c^2)/3$
$\Delta\rho_{max}$, $\Delta\rho_{min}$ (e Å^{-3})	2.66, -1.21

Computer programs: Apex4 v2021.10-RC6 (Bruker, 2021), *SAINT* V8.40B (Bruker, 2020), *SHELXT* 2014/5 (Sheldrick, 2014), *SHELXL2018*/3 (Sheldrick, 2015, 2018), SHELXLE Rev1183 (Hübschle *et al.*, 2011).

Refinement Details:

The structure is commensurately modulated with a three-fold pseudo-translation along the a-c diagonal. Larger than usual residuals are found at the positions of the bromide and zinc created by one or two-third translations along [1 0 -1]. Possible disorder associated with the pseudo-translation is not resolved for the C and N atoms and was thus ignored.

One of three tert-butyl groups (that of residue 1) was refined as disordered by rotation. The two disordered moieties were restrained to have similar geometries as another not disordered tert-butyl group (of residue 2). U_{ij} components of ADPs for disordered atoms closer to each other than 2.0 Å were restrained to be similar. Subject to these conditions the occupancy ratio refined to 0.632(15) to 0.368(15).

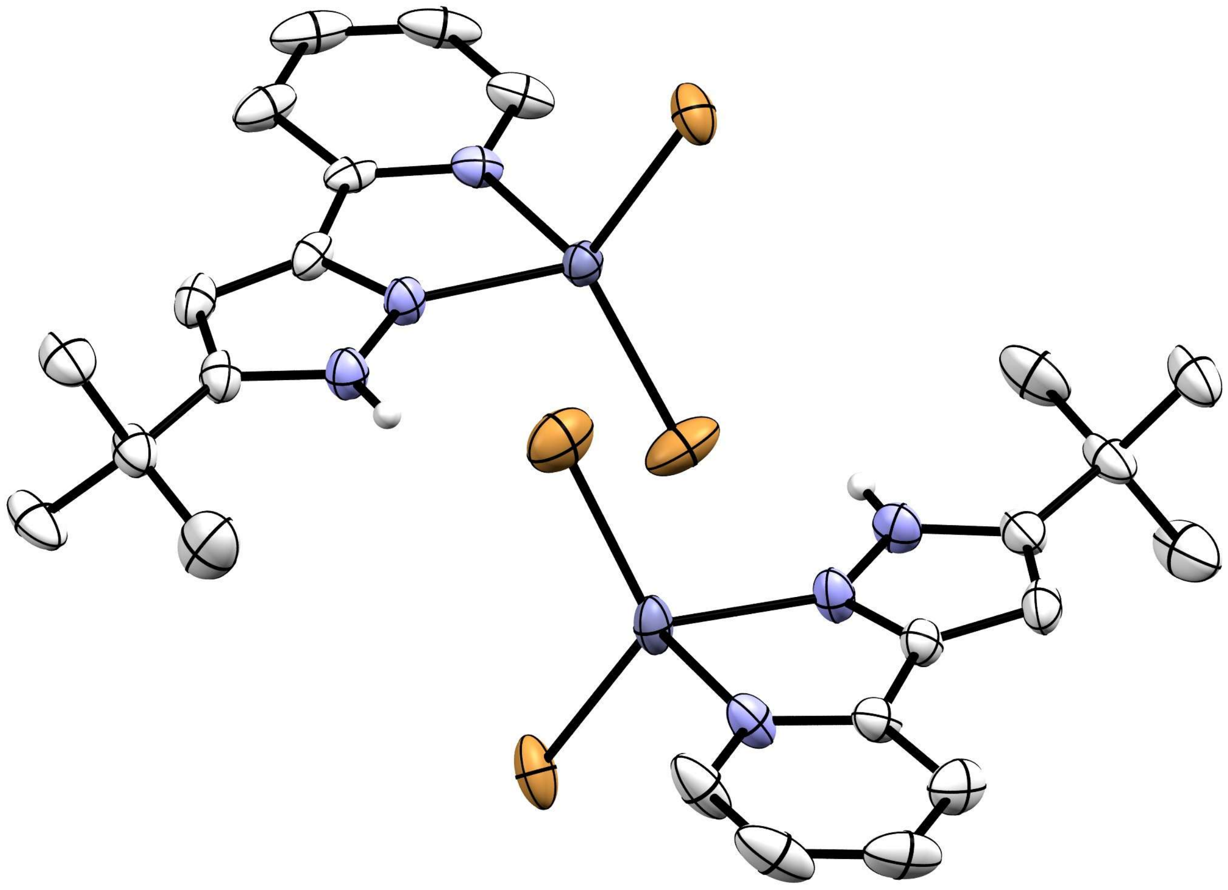

Figure S100. Two of the three crystallographically independent molecules of ($^{H}NN^{tBu}$)$ZnBr_2$ (**1-Br**) per unit cell displayed to highlight intermolecular hydrogen bonding interactions. Ellipsoids are displayed with 50% probability ellipsoids. Hydrogen atoms not connected to nitrogen are omitted for clarity.

Compound: (^{H}NN^{tBu})$ZnCl_2$ (**1-Cl**)
Local Name: JK-1-12 // JK-E-3
CCDC Number: 2395146

Table S2. Crystallographic details for (^{H}NN^{tBu})$ZnCl_2$ (**1-Cl**)

**Crystal data**	
Chemical formula	$C_{12}H_{15}Cl_2N_3Zn{\cdot}0.5(C_4H_8O_2)$
M_r	381.59
Crystal system, space group	Triclinic, $P\overline{1}$
Temperature (K)	150
a, *b*, *c* (Å)	9.3720 (5), 9.9149 (5), 10.5545 (4)
α, β, γ (°)	66.255 (2), 76.759 (2), 77.982 (2)
V (Å^3)	866.42 (7)
Z	2
Radiation type	Mo *K*α
μ (mm^{-1})	1.73
Crystal size (mm)	0.52 × 0.46 × 0.35
**Data collection**	
Diffractometer	Bruker AXS D8 Quest diffractometer with PhotonII charge-integrating pixel array detector (CPAD)
Absorption correction	Multi-scan *SADABS* 2016/2: Krause, L., Herbst-Irmer, R., Sheldrick G.M. & Stalke D., J. Appl. Cryst. 48 (2015) 3-10
T_{min}, T_{max}	0.612, 0.747
No. of measured, independent and observed [$I > 2\sigma(I)$] reflections	29089, 6346, 4949
R_{int}	0.038
$(\sin \theta/\lambda)_{max}$ (Å^{-1})	0.770
**Refinement**	
$R[F^2 > 2\sigma(F^2)]$, $wR(F^2)$, S	0.043, 0.128, 1.07
No. of reflections	6346
No. of parameters	222
No. of restraints	34
H-atom treatment	H-atom parameters constrained
$\Delta\rho_{max}$, $\Delta\rho_{min}$ (e Å^{-3})	0.70, -0.66

Computer programs: Apex3 v2018.7-2 (Bruker, 2018), *SAINT* V8.38A (Bruker, 2018), *SHELXS97* (Sheldrick, 2008), *SHELXL2018*/3 (Sheldrick, 2015, 2018), SHELXLE Rev1030 (Hübschle *et al.*, 2011).

Refinement Details:

An ethyl acetate molecule is disordered around an inversion center. C-C and C-O single bonds were restrained to target values of 1.53(2) and 1.43(2) Å, respectively. U_{ij} components of ADPs for disordered atoms closer to each other than 2.0 Å were restrained to be similar.

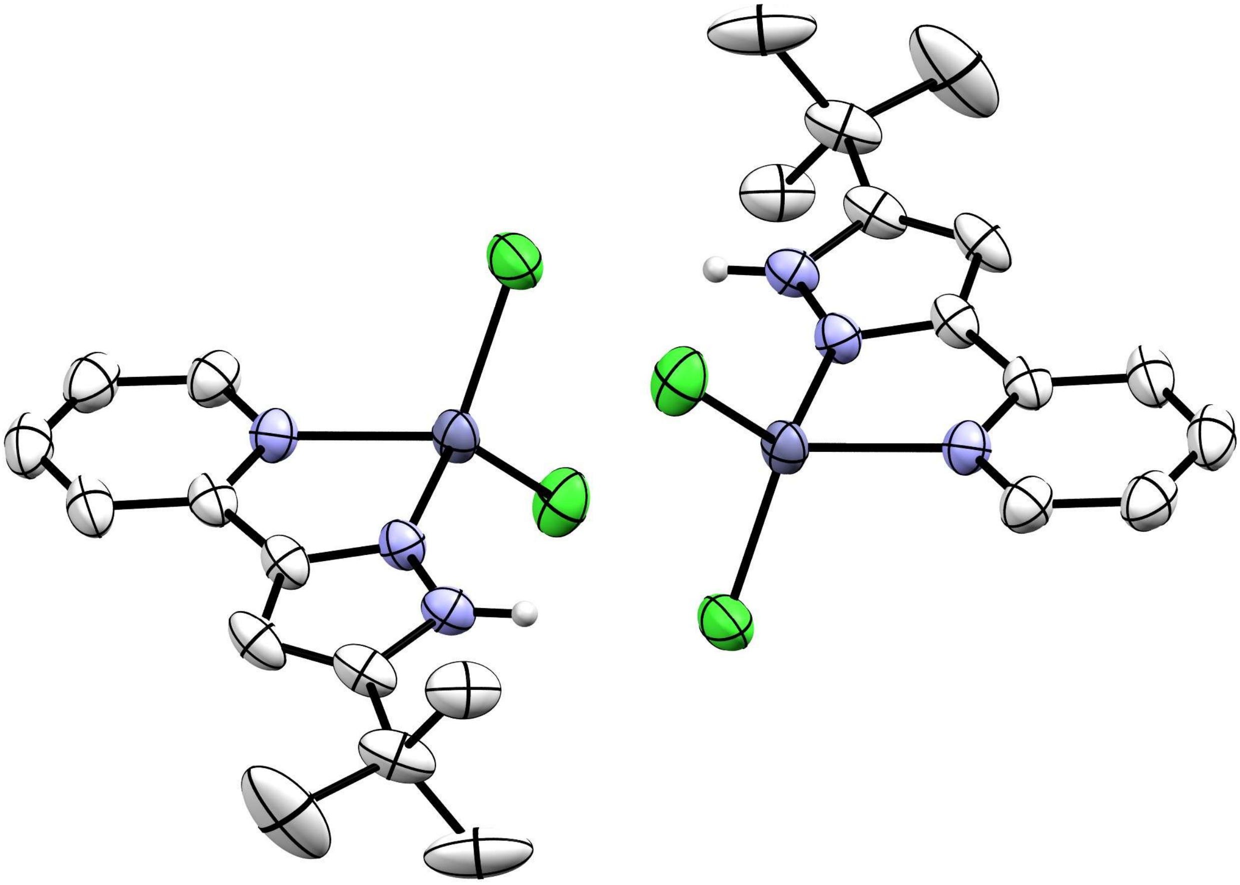

Figure S101. Extended structure of two crystallographically identical molecules of ($^{H}NN^{tBu}$)$ZnCl_2$ (**1-Cl**) to highlight intermolecular hydrogen bonding interactions. Ellipsoids are displayed with 50% probability ellipsoids. Hydrogen atoms not connected to nitrogen are omitted for clarity. Co-crystallized solvent (ethyl acetate) is also omitted.

Compound: $[(^{H}NN^{tBu})_3Zn][(ClO_4)_2]$ (**2-ClO₄**)

Local Name: JK-1-14 // JK-E-5

CCDC Number: 2395145

Table S3. Crystallographic details for $[(^{H}NN^{tBu})_3Zn][(ClO_4)_2]$ (**2-ClO$_4$**)

**Crystal data**	
Chemical formula	$2(C_{36}H_{45}N_9Zn)\cdot C_6H_{14}\cdot 4(ClO_4)\cdot 1.14(H_2O)$
M_r	1842.86
Crystal system, space group	Monoclinic, $P2_1/c$
Temperature (K)	150
a, b, c (Å)	10.1218 (7), 26.723 (2), 16.3647 (9)
β (°)	97.540 (4)
V (Å^3)	4388.2 (5)
Z	2
Radiation type	Cu $K\alpha$
μ (mm^{-1})	2.41
Crystal size (mm)	0.11 × 0.10 × 0.01
**Data collection**	
Diffractometer	Bruker AXS D8 Quest diffractometer with PhotonIII_C14 charge-integrating pixel array detector (CPAD)
Absorption correction	Multi-scan *SADABS* 2016/2: Krause, L., Herbst-Irmer, R., Sheldrick G.M. & Stalke D., J. Appl. Cryst. 48 (2015) 3-10
T_{min}, T_{max}	0.586, 0.754
No. of measured, independent and observed [$I > 2\sigma(I)$] reflections	39387, 9252, 5720
R_{int}	0.119
$(\sin \theta/\lambda)_{max}$ (Å^{-1})	0.638
**Refinement**	
$R[F^2 > 2\sigma(F^2)]$, $wR(F^2)$, S	0.080, 0.187, 1.05
No. of reflections	9252
No. of parameters	716
No. of restraints	889
H-atom treatment	H atoms treated by a mixture of independent and constrained refinement
	$w = 1/[s^2(F_o^2) + (0.0322P)^2 + 11.5996P]$ where $P = (F_o^2 + 2F_c^2)/3$
$\Delta\rho_{max}$, $\Delta\rho_{min}$ (e Å^{-3})	0.81, -0.64

Computer programs: Apex3 v2019.1-0 (Bruker, 2019), *SAINT* V8.40A (Bruker, 2019), *SHELXS97* (Sheldrick, 2008), *SHELXL2018*/3 (Sheldrick, 2015, 2018), SHELXLE Rev1030 (Hübschle *et al.*, 2011).

Refinement Details:

A hexane molecule is disordered around an inversion center by a slight shift of the hexane center off the inversion center. C-C bond distances were restrained to target distances of 1.53(2) Å (CH_3-CH_2) and 1.51(2) Å (CH_2-CH_2), and 1,3 C...C distances were restrained to at least 2.50(2) Å. U_{ij} components of ADPs for disordered atoms closer to each other than 2.0 Å were restrained to be similar and atoms were restrained to be close to isotropic.

The presence of a partially occupied water molecule induces disorder for the two perchlorate anions. All perchlorate Cl-O and O...O distances were restrained to be each similar to each other. Water H atom positions were refined and O-H and H...H distances were restrained to 0.84(2) and 1.36(2) Å, respectively. The position of water H atom H9A was further restrained based on hydrogen bonding considerations (2.35(2) Å distance to O6). U_{ij} components of ADPs for disordered atoms closer to each other than 2.0 Å were restrained to be similar. Subject to these conditions the occupancy ratio refined to 0.570(7) to 0.430(7) in favor of water present.

A *tert*-butyl group was refined as disordered. The two disordered moieties were restrained to have similar geometries. U_{ij} components of ADPs for disordered atoms closer to each other than 2.0 Å were restrained to be similar. Subject to these conditions the occupancy ratio refined to 0.800(11) to 0.200(11).

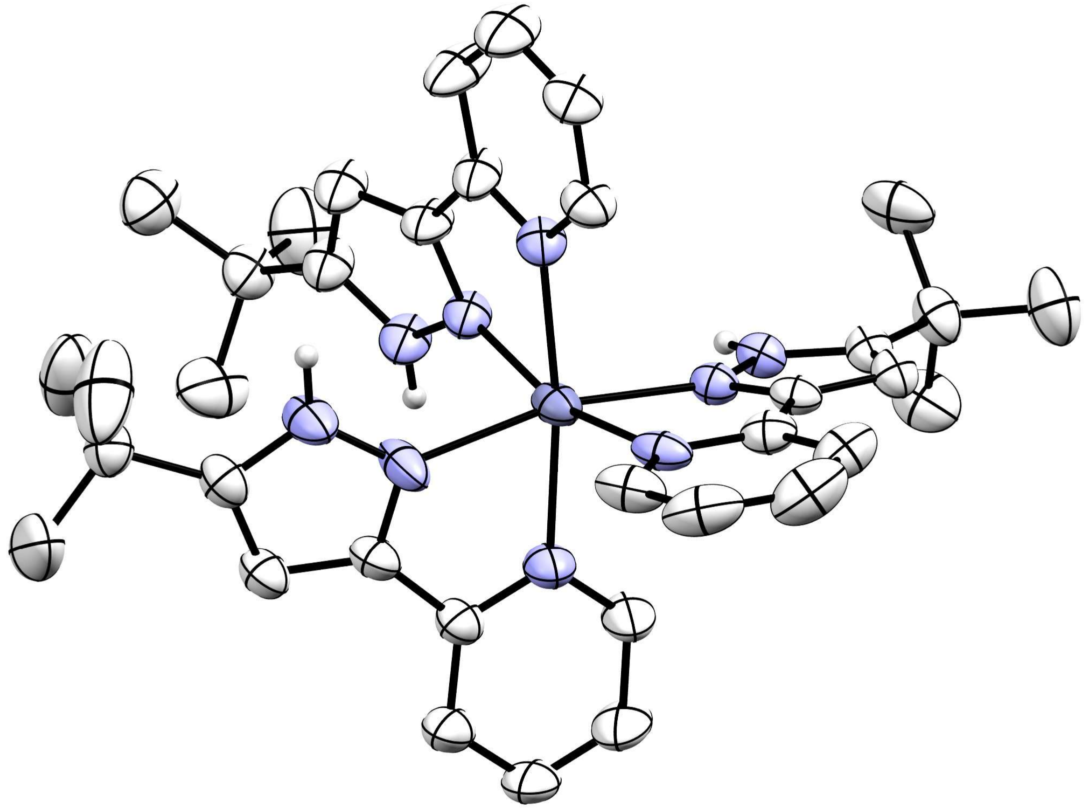

Figure S102. Molecular structure of the cationic portion of $[(^{H}NN^{tBu})_3Zn][(ClO_4)_2]$ (**2-ClO₄**) displayed with 50% probability ellipsoids. Hydrogen atoms not connected to nitrogen are omitted for clarity. Co-crystallized solvents (H_2O) and disordered moieties are omitted to improve clarity.

Compound: $[(^{H}NN^{tBu})_3Zn][Zn(NCS)_3X]$ X = NCS, Cl (**2-SCN**)
Local Name: GC-27
CCDC Number: 2395143
Table S4. Crystallographic details for $[(^{H}NN^{tBu})_3Zn][Zn(NCS)_3X]$ X = NCS, Cl (**2-SCN**)

**Crystal data**	
Chemical formula	$C_{36}H_{45}N_9Zn{\cdot}C_{3.31}Cl_{0.69}N_{3.31}S_{3.31}Zn{\cdot}0.44(C_6H_{14}){\cdot}0.87(C_2H_4Cl_2)$
M_r	1075.34
Crystal system, space group	Monoclinic, *Cc*
Temperature (K)	150
a, *b*, *c* (Å)	11.766 (3), 23.140 (8), 20.793 (7)
β (°)	105.774 (14)
V (Å^3)	5448 (3)
Z	4
Radiation type	Cu *K*α
μ (mm^{-1})	3.69
Crystal size (mm)	0.12 × 0.11 × 0.05
**Data collection**	
Diffractometer	Bruker AXS D8 Quest
Absorption correction	Multi-scan *SADABS* 2016/2: Krause, L., Herbst-Irmer, R., Sheldrick G.M. & Stalke D., J. Appl. Cryst. 48 (2015) 3-10
T_{min}, T_{max}	0.624, 0.754
No. of measured, independent and observed [$I > 2\sigma(I)$] reflections	27920, 9978, 7069
R_{int}	0.063
$(\sin \theta/\lambda)_{max}$ (Å^{-1})	0.641
**Refinement**	
$R[F^2 > 2\sigma(F^2)]$, $wR(F^2)$, S	0.070, 0.206, 1.05
No. of reflections	9978
No. of parameters	760
No. of restraints	541
H-atom treatment	H-atom parameters constrained
$\Delta\rho_{max}$, $\Delta\rho_{min}$ (e Å^{-3})	0.67, -0.69
Absolute structure	Refined as a 2-component inversion twin.
Absolute structure parameter	0.06 (4)

Computer programs: Apex4 v2022.10-0 (Bruker, 2022), *SAINT* V8.40B (Bruker, 2020), *SHELXT* (Sheldrick, 2015b), *SHELXL2019*/2 (Sheldrick, 2015a, 2019), SHELXLE Rev1541 (Hübschle *et al.*, 2011).

Refinement Details:

Refined as a 2-component inversion twin.

The anion is disordered with one of the thiocyanate ligands partially replaced by a chlorido moiety. The disordered thiocyanate was restrained to have a similar geometry as one of the not disordered thiocyanates. The four Fe-N distances were restrained to be similar in length. U_{ij} components of ADPs for the zinc atom and disordered atoms closer to each other than 2.0 Å were restrained to be similar. Subject to these conditions the occupancy ratio refined to 0.688(15) to 0.312(15).

A solvate pocket is disordered. The disorder is correlated with the thiocyanate/chloride disorder, but occupancy rates do not match exactly. The solvate disorder also induces disorder to one of the *tert*-butyl groups and neighboring atoms of the cation. Occupancy rates were not forced to exactly match.

The two disordered moieties of the cation were restrained to have similar geometries as another equivalent, not-disordered moiety. Atoms N2 and N2B were constrained to have identical ADPs. U_{ij} components of ADPs for disordered atoms closer to each other than 2.0 Å were restrained to be similar. Subject to these conditions the occupancy ratio refined to 0.704(18) to 0.296(18).

The solvate pocket was refined as occupied by two moieties of 1,2-dichloroethane and one moiety of hexane. Equivalent bonds of the two dichloroethane molecules were restrained to be similar in length. The C-C bond length of one moiety was restrained to a target value of 1.53(2) Å. The bond lengths and angles of the hexane molecule were restrained to expected target values. U_{ij} components of ADPs for disordered atoms closer to each other than 2.0 Å were restrained to be similar. Subject to these conditions the occupancy ratio refined to 0.552(13) and 0.318(12) for the dichloroethane molecules and 0.44(2) for the hexane molecule.

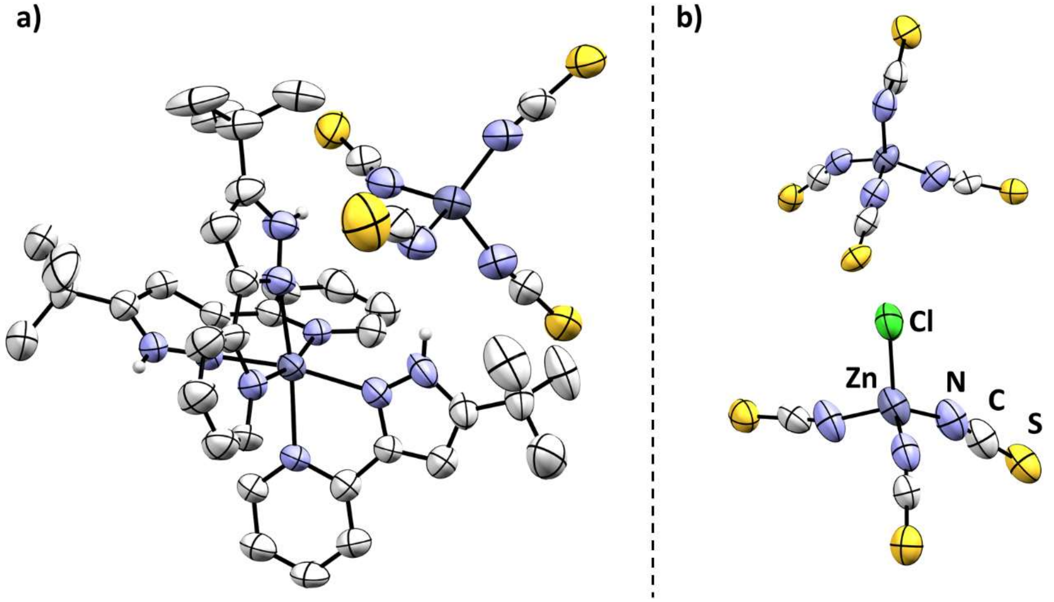

Figure S103. A) The major moiety of the molecular structure of $[(^{H}NN^{tBu})_3Zn][Zn(NCS)_3X]$ X = NCS, Cl (**2-SCN**) displayed with 50% probability ellipsoids. Hydrogen atoms not connected to nitrogen are omitted for clarity. B) The major $[Zn(NCS)_4]^{2-}$ (top) and minor $[Zn(NCS)_3Cl]^{2-}$ (bottom) moieties of the counter anion.

Compound: $(^{H}NN^{tBu})_2Zn(OAc)_2$ (**3**)
Local Name: JK-1-11 // JK-E-2
CCDC Number: 2395152
Table S5. Crystallographic details for $(^{H}NN^{tBu})_2Zn(OAc)_2$ (**3**)

**Crystal data**	
Chemical formula	$C_{28}H_{36}N_6O_4Zn·0.5(C_6H_{14})$
M_r	629.08
Crystal system, space group	Monoclinic, *C*2/*c*
Temperature (K)	150
a, *b*, *c* (Å)	15.2862 (7), 27.2387 (14), 17.0445 (7)
β (°)	115.450 (2)
V (Å^3)	6408.2 (5)
Z	8
Radiation type	Mo *K*α
μ (mm^{-1})	0.81
Crystal size (mm)	0.43 × 0.33 × 0.10
**Data collection**	
Diffractometer	Bruker AXS D8 Quest diffractometer with PhotonII charge-integrating pixel array detector (CPAD)
Absorption correction	Multi-scan *SADABS* 2016/2: Krause, L., Herbst-Irmer, R., Sheldrick G.M. & Stalke D., J. Appl. Cryst. 48 (2015) 3-10
T_{min}, T_{max}	0.659, 0.747
No. of measured, independent and observed [$I > 2\sigma(I)$] reflections	55729, 11986, 7938
R_{int}	0.047
$(\sin \theta/\lambda)_{max}$ (Å^{-1})	0.770
**Refinement**	
$R[F^2 > 2\sigma(F^2)]$, $wR(F^2)$, S	0.042, 0.123, 1.03
No. of reflections	11986
No. of parameters	502
No. of restraints	288
H-atom treatment	H-atom parameters constrained
$\Delta\rho_{max}$, $\Delta\rho_{min}$ (e Å^{-3})	0.44, -0.46

Computer programs: Apex3 v2018.7-2 (Bruker, 2018), *SAINT* V8.38A (Bruker, 2018), *SHELXS97* (Sheldrick, 2008), *SHELXL2018*/3 (Sheldrick, 2015, 2018), SHELXLE Rev1030 (Hübschle *et al.*, 2011).

Refinement Details:

A hexane molecule is located on or around an inversion center, being disordered over three independent sites. The major site is symmetric with respect to the inversion center. The other two moieties are slightly shifted and disordered around the inversion center. C-C bond distances were restrained to target values of 1.53(2) Å for CH_3-CH_2 and 1.51(2) Å for CH_2-CH_2. All 1,3 C...C distances were restrained to be similar. U_{ij} components of ADPs for disordered atoms closer to each other than 2.0 Å were restrained to be similar. Subject to these conditions the occupancy rates refined to 0.532(3) for the inversion symmetric moiety, and to two times 0.1184(14) and two times 0.1157(14) for the other two moieties.

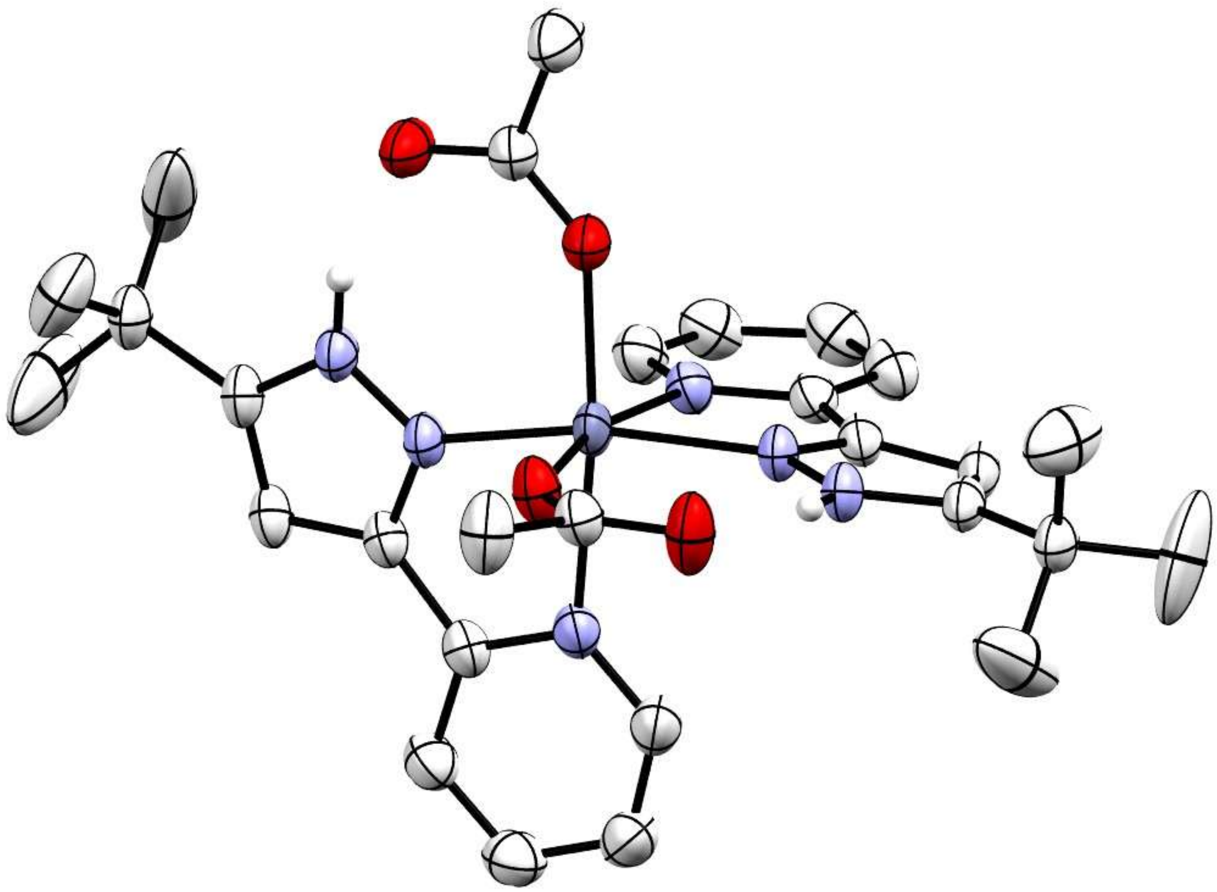

Figure S104. Molecular structure of ($^{H}NN^{tBu}$)$_2$Zn(OAc)$_2$ (**3**) displayed with 50% probability ellipsoids. Hydrogen atoms not connected to nitrogen are omitted for clarity. Disordered solvent (hexane) is omitted for clarity.

Compound: (^{Me}NN^{tBu})Zn(OAc)$_2$ (**3'**)
Local Name: JK-1-68
CCDC Number: 2443234

Table S6. Crystallographic details for (^{Me}NN^{tBu})Zn(OAc)$_2$ (**3'**)

**Crystal data**	
Chemical formula	$C_{17}H_{23}N_3O_4Zn$
M_r	398.75
Crystal system, space group	Monoclinic, $P2_1/n$
Temperature (K)	150
a, *b*, *c* (Å)	10.1943 (5), 10.5344 (6), 17.2321 (9)
β (°)	98.880 (2)
V (Å^3)	1828.39 (17)
Z	4
Radiation type	Mo *K*α
μ (mm^{-1})	1.37
Crystal size (mm)	0.45 × 0.45 × 0.31
**Data collection**	
Diffractometer	Bruker AXS D8 Quest
Absorption correction	Multi-scan *SADABS* 2016/2: Krause, L., Herbst-Irmer, R., Sheldrick G.M. & Stalke D., J. Appl. Cryst. 48 (2015) 3-10
T_{min}, T_{max}	0.614, 0.747
No. of measured, independent and observed [$I > 2\sigma(I)$] reflections	56510, 6997, 5947
R_{int}	0.038
$(\sin \theta/\lambda)_{max}$ (Å^{-1})	0.771
**Refinement**	
$R[F^2 > 2\sigma(F^2)]$, $wR(F^2)$, S	0.031, 0.088, 1.06
No. of reflections	6997
No. of parameters	233
H-atom treatment	H-atom parameters constrained
$\Delta\rho_{max}$, $\Delta\rho_{min}$ (e Å^{-3})	0.48, -0.60

Computer programs: Apex5 v2023.9-2 (Bruker, 2023), *SAINT* V8.40B (Bruker, 2020), *SHELXT* (Sheldrick, 2015b), *SHELXL2019*/2 (Sheldrick, 2015a, 2019), SHELXLE Rev1703 (Hübschle *et al.*, 2011).

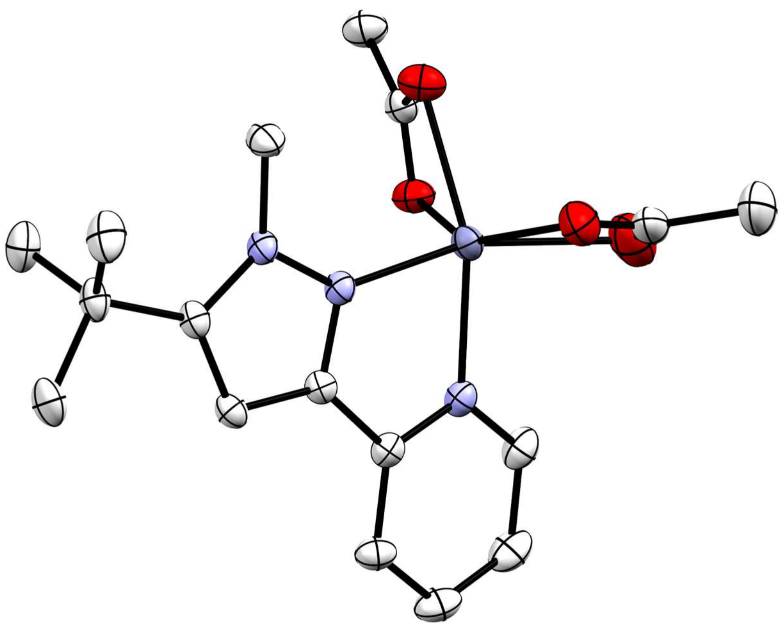

Figure S105. Molecular structure of ($^{Me}NN^{tBu}$)Zn(OAc)$_2$ (**3'**) displayed with 50% probability ellipsoids. All hydrogen atoms are omitted for clarity.

Compound: $(^{H}NN^{tBu})_2Zn(O_2CFc)_2$ (**4**)
Local Name: JK-1-35
CCDC Number: 2395149

Table S7. Crystallographic details for $(^{H}NN^{tBu})_2Zn(O_2CFc)_2$ (**4**)

**Crystal data**	
Chemical formula	$C_{46}H_{48}Fe_2N_6O_4Zn{\cdot}CH_4O{\cdot}0.253(H_2O)$
M_r	962.57
Crystal system, space group	Monoclinic, $P2_1/c$
Temperature (K)	150
a, *b*, *c* (Å)	19.4935 (7), 21.4475 (6), 11.0027 (4)
β (°)	99.004 (2)
V (Å^3)	4543.4 (3)
Z	4
Radiation type	Mo $K\alpha$
μ (mm^{-1})	1.21
Crystal size (mm)	0.43 × 0.38 × 0.26
**Data collection**	
Diffractometer	Bruker AXS D8 Quest diffractometer with PhotonII charge-integrating pixel array detector (CPAD)
Absorption correction	Multi-scan *SADABS* 2016/2: Krause, L., Herbst-Irmer, R., Sheldrick G.M. & Stalke D., J. Appl. Cryst. 48 (2015) 3-10
T_{min}, T_{max}	0.667, 0.747
No. of measured, independent and observed [$I > 2\sigma(I)$] reflections	103436, 17307, 12878
R_{int}	0.049
$(\sin \theta/\lambda)_{max}$ (Å^{-1})	0.770
**Refinement**	
$R[F^2 > 2\sigma(F^2)]$, $wR(F^2)$, S	0.041, 0.112, 1.04
No. of reflections	17307
No. of parameters	702
No. of restraints	433
H-atom treatment	H atoms treated by a mixture of independent and constrained refinement
$\Delta\rho_{max}$, $\Delta\rho_{min}$ (e Å^{-3})	1.11, -0.67

Computer programs: Apex4 v2022.10-RC10 (Bruker, 2022), *SAINT* V8.40B (Bruker, 2020), *SHELXT* (Sheldrick, 2015), *SHELXL2018*/3 (Sheldrick, 2015, 2018), SHELXLE Rev1275 (Hübschle *et al.*, 2011).

Refinement Details:

A *tert*-butyl group and a cyclopentadienyl ring were refined as disordered by rotation. The disordered moieties were restrained to have similar geometries as the other non-disordered *tert*-butyl group and a cyclopentadienyl ring in the structure. U_{ij} components of ADPs for disordered atoms closer to each other than 2.0 Å were restrained to be similar. For the cyclopentadienyl ring, the atoms were also restrained to be close to isotropic. Subject to these conditions the occupancy ratio refined to 0.760(6) to 0.240(6) for the *tert*-butyl group, and to 0.64(2) to 0.36(2) for the cyclopentadienyl ring.

Positions of N-bound H-atoms were refined and N-H bond distances were restrained to a target value of 0.87(2) Å.

A solvate methanol molecule, H-bonded to a carboxylate oxygen atom, was refined as disordered over three positions. Associated with the disorder is a partially occupied water molecule. The methanol C-O distances were restrained to be similar. U_{ij} components of ADPs for disordered atoms closer to each other than 2.0 Å were restrained to be similar. Water H atom positions were initially refined and O-H and H...H distances were restrained to 0.84(2) and 1.36(2) Å, respectively, while a damping factor was applied. Water and hydroxyl H atom positions were further restrained based on hydrogen bonding considerations. In the final refinement cycles, the water H-atoms were constrained to ride on their carrier oxygen atom and the damping factor was removed. Subject to these conditions the methanol occupancy rates refined to 0.424(3), 0.446(3) and 0.130(3) and the water occupancy rate to 0.253(9).

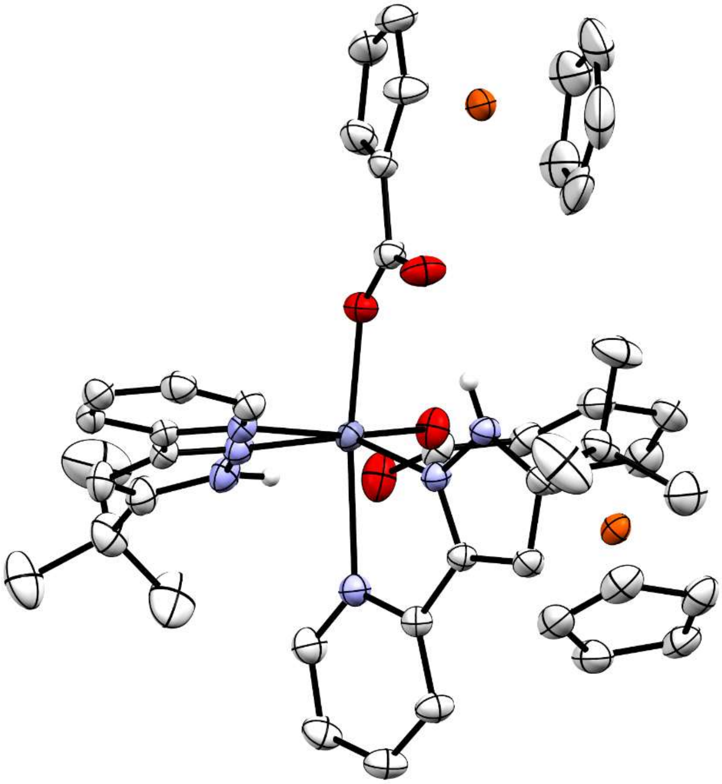

Figure S106. Molecular structure of $(^{H}NN^{tBu})_2Zn(O_2CFc)_2$ (**4**) displayed with 50% probability ellipsoids. Hydrogen atoms not connected to nitrogen are omitted for clarity. Disordered fragments and solvent molecules are omitted for clarity.

Compound: $(^{Me}NN^{tBu})Zn(O_2CFc)_2(H_2O)$ (**4’-hydrate**)
Local Name: CG-53
CCDC Number: 2495157

Table S8. Crystallographic details for $(^{Me}NN^{tBu})Zn(O_2CFc)_2$ (**4’-hydrate**)

**Crystal data**	
Chemical formula	$C_{35}H_{37}Fe_2N_3O_5Zn{\cdot}H_2O$
M_r	774.78
Crystal system, space group	Triclinic, $P\overline{1}$
Temperature (K)	150
a, *b*, *c* (Å)	11.225 (3), 11.723 (3), 12.793 (4)
α, β, γ (°)	84.943 (9), 76.323 (9), 81.994 (9)
V (Å^3)	1617.1 (8)
Z	2
Radiation type	Mo *K*α
μ (mm^{-1})	1.67
Crystal size (mm)	0.09 × 0.07 × 0.03
**Data collection**	
Diffractometer	Bruker AXS D8 Quest
Absorption correction	Multi-scan *SADABS* 2016/2: Krause, L., Herbst-Irmer, R., Sheldrick G.M. & Stalke D., J. Appl. Cryst. 48 (2015) 3-10
T_{min}, T_{max}	0.636, 0.747
No. of measured, independent and observed [$I > 2\sigma(I)$] reflections	54407, 12427, 8215
R_{int}	0.060
$(\sin \theta/\lambda)_{max}$ (Å^{-1})	0.773
**Refinement**	
$R[F^2 > 2\sigma(F^2)]$, $wR(F^2)$, S	0.047, 0.124, 1.02
No. of reflections	12427
No. of parameters	443
No. of restraints	8
H-atom treatment	H atoms treated by a mixture of independent and constrained refinement
$\Delta\rho_{max}$, $\Delta\rho_{min}$ (e Å^{-3})	1.71, -0.67

Computer programs: Apex6 v2024.9-1 (Bruker, 2025), *SAINT* V8.41 (Bruker, 2025), *SHELXT* (Sheldrick, 2015b), *SHELXL2019*/2 (Sheldrick, 2015a, 2019), SHELXLE Rev1737 (Hübschle *et al.*, 2011).

Refinement Details:

A solvate water molecule is H-bonded to its own counterpart by inversion. One of the H atoms was thus refined as 1:1 disordered. Water H atom positions were refined and O-H distances were restrained to 0.84(2) Å. For the disordered hydrogens H...H distances were restrained to 1.36(2) Å, and H atom positions were further restrained based on hydrogen bonding considerations.

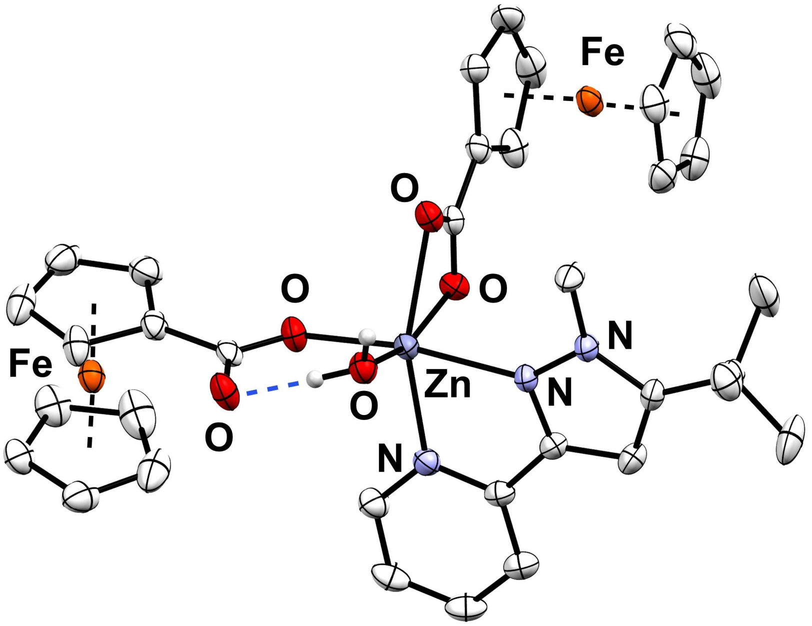

Figure S107. Molecular structure of ($^{Me}NN^{tBu}$)Zn(O_2CFc)$_2$(H_2O) (**4'-hydrate**) displayed with 50% probability ellipsoids. All hydrogen atoms are omitted for clarity.

Compound: $(^{H}NN^{tBu})_2Zn(OTf)_2$ (**5**)
Local Name: JK-1-15 // JK-E-6
CCDC Number: 2395147

Table S9. Crystallographic details for $(^{H}NN^{tBu})_2Zn(OTf)_2$ (**5**)

**Crystal data**	
Chemical formula	$C_{26}H_{30}F_6N_6O_6S_2Zn$
M_r	766.05
Crystal system, space group	Orthorhombic, *Pbcn*
Temperature (K)	150
a, *b*, *c* (Å)	16.4922 (15), 10.0786 (8), 19.1493 (15)
V (Å^3)	3183.0 (5)
Z	4
Radiation type	Mo $K\alpha$
μ (mm^{-1})	0.99
Crystal size (mm)	0.18 × 0.15 × 0.08
**Data collection**	
Diffractometer	Bruker AXS D8 Quest diffractometer with PhotonII charge-integrating pixel array detector (CPAD)
Absorption correction	Multi-scan *SADABS* 2016/2: Krause, L., Herbst-Irmer, R., Sheldrick G.M. & Stalke D., J. Appl. Cryst. 48 (2015) 3-10
T_{min}, T_{max}	0.633, 0.747
No. of measured, independent and observed [$I > 2\sigma(I)$] reflections	35265, 5665, 3954
R_{int}	0.079
$(\sin \theta/\lambda)_{max}$ (Å^{-1})	0.769
**Refinement**	
$R[F^2 > 2\sigma(F^2)]$, $wR(F^2)$, S	0.042, 0.106, 1.02
No. of reflections	5665
No. of parameters	216
H-atom treatment	H-atom parameters constrained
$\Delta\rho_{max}$, $\Delta\rho_{min}$ (e Å^{-3})	0.41, -0.45

Computer programs: Apex3 v2018.7-2 (Bruker, 2018), *SAINT* V8.38A (Bruker, 2018), *SHELXS97* (Sheldrick, 2008), *SHELXL2018*/3 (Sheldrick, 2015, 2018), SHELXLE Rev1030 (Hübschle *et al.*, 2011).

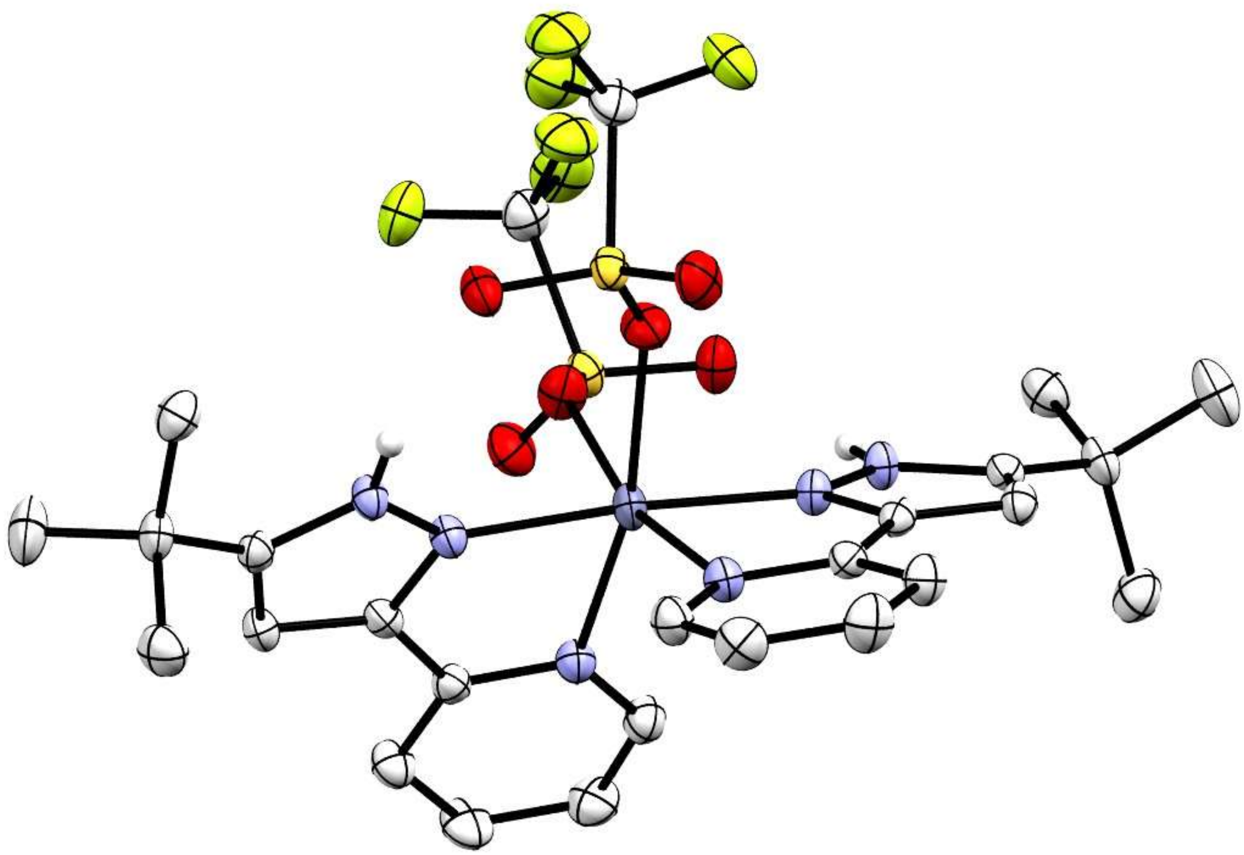

Figure S108. Molecular structure of ($^{H}NN^{tBu}$)$_2$Zn(OTf)$_2$ (**5**) displayed with 50% probability ellipsoids. Hydrogen atoms not connected to nitrogen are omitted for clarity.

Compound: $(^{Me}NN^{tBu})_2Zn(OTf)_2$ (**5'**)
Local Name: CG-68
CCDC Number: 2495156

Table S10. Crystallographic details for $(^{Me}NN^{tBu})_2Zn(OTf)_2$ (**5'**)

**Crystal data**	
Chemical formula	$C_{28}H_{34}F_6N_6O_6S_2Zn$
M_r	794.10
Crystal system, space group	Triclinic, $P\overline{1}$
Temperature (K)	150
a, *b*, *c* (Å)	9.5827 (4), 13.6968 (6), 14.1058 (7)
α, β, γ (°)	90.694 (2), 103.294 (2), 105.323 (2)
V (Å^3)	1732.51 (14)
Z	2
Radiation type	Mo *K*α
μ (mm^{-1})	0.91
Crystal size (mm)	0.59 × 0.27 × 0.10
**Data collection**	
Diffractometer	Bruker AXS D8 Quest
Absorption correction	Multi-scan *SADABS* 2016/2: Krause, L., Herbst-Irmer, R., Sheldrick G.M. & Stalke D., J. Appl. Cryst. 48 (2015) 3-10
T_{min}, T_{max}	0.582, 0.746
No. of measured, independent and observed [$I > 2\sigma(I)$] reflections	71662, 8652, 7698
R_{int}	0.051
$(\sin \theta/\lambda)_{max}$ (Å^{-1})	0.668
**Refinement**	
$R[F^2 > 2\sigma(F^2)]$, $wR(F^2)$, S	0.029, 0.078, 1.04
No. of reflections	8652
No. of parameters	523
No. of restraints	291
H-atom treatment	H-atom parameters constrained
$\Delta\rho_{max}$, $\Delta\rho_{min}$ (e Å^{-3})	0.36, -0.48

Computer programs: Apex6 v2024.9-1 (Bruker, 2025), *SAINT* V8.41 (Bruker, 2025), *SHELXT* (Sheldrick, 2015b), '*SHELXL2025*/1 (Sheldrick, 2025), SHELXLE Rev1737 (Hübschle *et al.*, 2011).

Refinement Details

Disorder was observed for the trifluoromethanesulfonate group coordinated to Zn. The major and minor moieties were restrained to have similar geometry to another not disordered trifluoromethanesulfonate moiety (SAME restrains of Shelxl, esd 0.02 Å). U_{ij} components of ADPs for disordered atoms closer to each other than 2.0 Å were restrained to be similar (SIMU restraints of Shelxl, esd 0.01 Å^2). Subject to these conditions the occupancy ratio refined to 0.609(15) to 0.391(15).

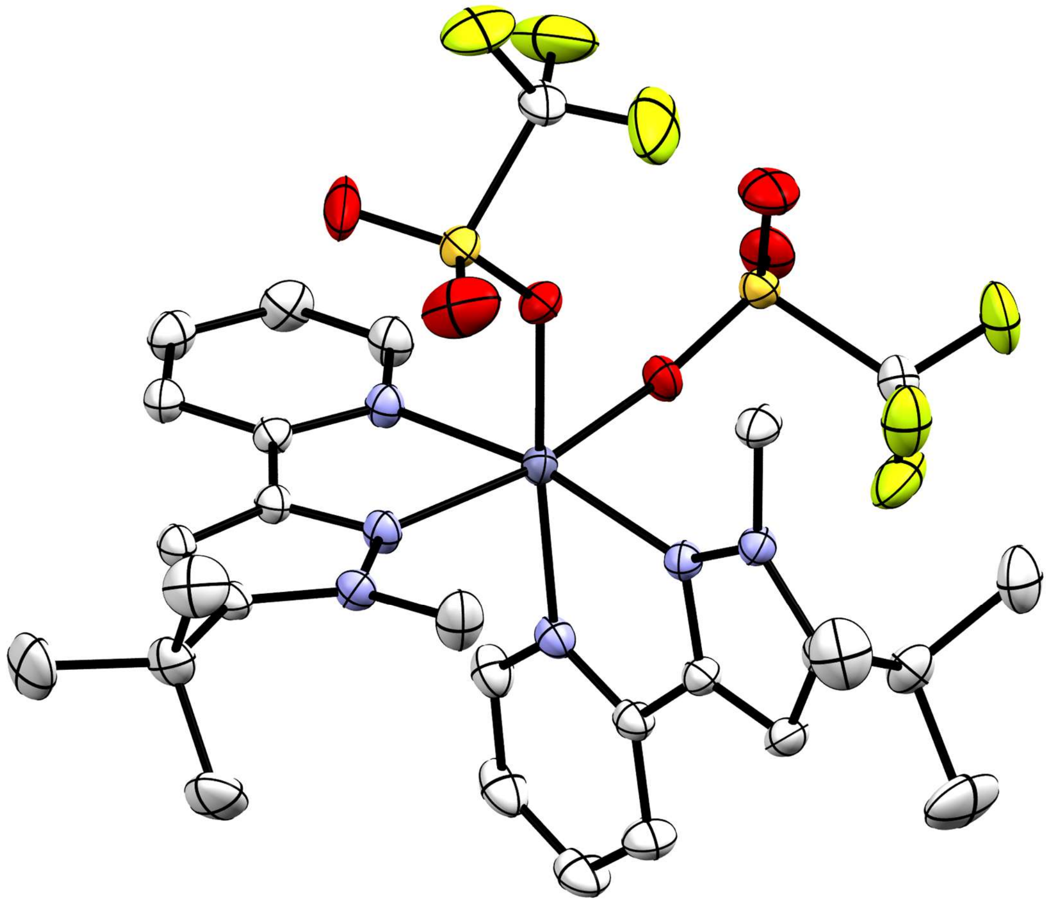

Figure S109. Molecular structure of (^{Me}NN^{tBu})$_2$Zn(OTf)$_2$ (**5'**) displayed with 50% probability ellipsoids. Hydrogen atoms are omitted for clarity.

Compound: $[(^{Me}NN^{tBu})Zn(H_2O)_n][OTf]_2$
Local Name: CG-56_chloroform
CCDC Number: 2495159

Table S11. Crystallographic details for $[(^{Me}NN^{tBu})Zn(H_2O)_n][OTf]_2$

**Crystal data**	
Chemical formula	$0.911(C_{13}H_{25}N_3O_4Zn)\cdot 0.089(C_{13}H_{23}N_3O_3Zn)\cdot 2(CF_3O_3S)$
M_r	649.25
Crystal system, space group	Monoclinic, $P2_1/n$
Temperature (K)	150
a, *b*, *c* (Å)	11.9652 (10), 8.8414 (6), 25.248 (2)
β (°)	102.961 (3)
V (Å^3)	2602.9 (4)
Z	4
Radiation type	Mo *K*α
μ (mm^{-1})	1.20
Crystal size (mm)	0.38 × 0.36 × 0.13
**Data collection**	
Diffractometer	Bruker AXS D8 Quest
Absorption correction	Multi-scan *SADABS* 2016/2: Krause, L., Herbst-Irmer, R., Sheldrick G.M. & Stalke D., J. Appl. Cryst. 48 (2015) 3-10
T_{min}, T_{max}	0.580, 0.747
No. of measured, independent and observed [$I > 2\sigma(I)$] reflections	90319, 9974, 8063
R_{int}	0.059
$(\sin \theta/\lambda)_{max}$ (Å^{-1})	0.772
**Refinement**	
$R[F^2 > 2\sigma(F^2)]$, $wR(F^2)$, S	0.057, 0.116, 1.09
No. of reflections	9974
No. of parameters	463
No. of restraints	305
H-atom treatment	H atoms treated by a mixture of independent and constrained refinement
$\Delta\rho_{max}$, $\Delta\rho_{min}$ (e Å^{-3})	0.78, -0.81

Computer programs: Apex6 v2024.9-1 (Bruker, 2025), *SAINT* V8.41 (Bruker, 2025), *SHELXT* (Sheldrick, 2015b), *SHELXL2019*/2 (Sheldrick, 2015a, 2019), SHELXLE Rev1737 (Hübschle *et al.*, 2011).

Refinement Details:

The cation consists of a mixture of a major tetrahydrate and a minor trihydrate. The absence of one coordinated water induces disorder of two other water molecules and the zinc atom (which is in the less sterically crowded trihydrate is more coplanar with the chelating ligand). Major and minor atoms of O4 and Zn1 are in close proximity and their ADPs were constrained to be each identical. The ADPs of disordered O3 and O3B were freely refined. Water H atom positions were refined and O-H and H...H distances were restrained to 0.84(2) and 1.36(2) Å, respectively. Some water H atom positions were further restrained based on hydrogen bonding considerations. Subject to these conditions the occupancy ratio refined to 0.911(3) to 0.089(3).

One of the triflate anions is independently disordered. The two disordered moieties were restrained to have similar geometries as the other not disordered triflate. U_{ij} components of ADPs for disordered atoms closer to each other than 2.0 Å were restrained to be similar. Subject to these conditions the occupancy ratio refined to 0.414(12) to 0.586(12).

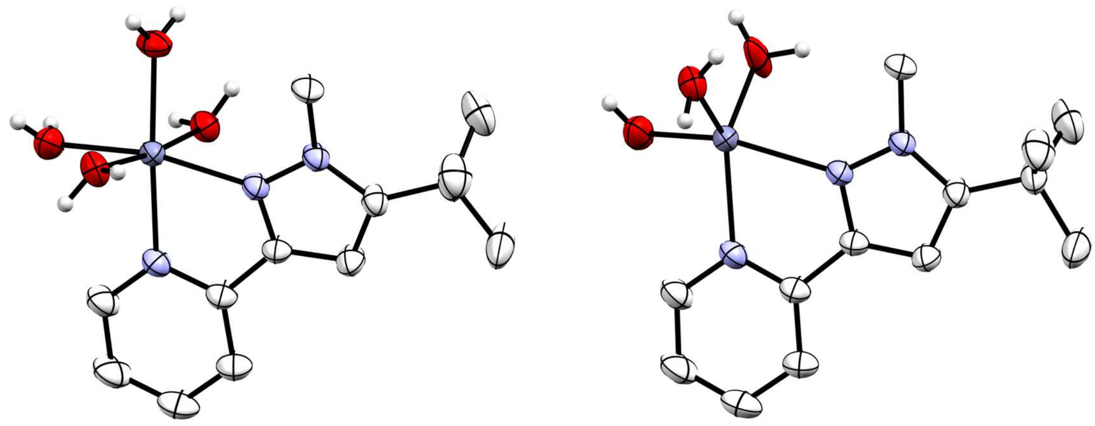

Figure S110. The major moiety (left) and minor moiety (right) of the cationic portion of the molecular structure of $[(^{Me}NN^{tBu})Zn(H_2O)_n][OTf]_2$ displayed with 50% probability ellipsoids (outer-sphere trifluoromethanesulfonate anions omitted). All hydrogen atoms not attached to heteroatoms are omitted for clarity.

Compound: $[(^{Me}NN^{tBu})_2Zn(H_2O)_2][OTf]_2$
Local Name: CG-56_acetone
CCDC Number: 2495158

Table S12. Crystallographic details for $[(^{Me}NN^{tBu})_2Zn(H_2O)_2][OTf]_2$

**Crystal data**	
Chemical formula	$C_{26}H_{38}N_6O_2Zn{\cdot}2(CF_3O_3S)$
M_r	830.13
Crystal system, space group	Orthorhombic, $P2_12_12_1$
Temperature (K)	150
a, *b*, *c* (Å)	9.6062 (2), 16.4951 (5), 23.1922 (5)
V (Å^3)	3674.93 (16)
Z	4
Radiation type	Cu *K*α
m (mm^{-1})	2.75
Crystal size (mm)	0.17 × 0.12 × 0.02
**Data collection**	
Diffractometer	Bruker AXS D8 Quest
Absorption correction	Multi-scan *SADABS* 2016/2: Krause, L., Herbst-Irmer, R., Sheldrick G.M. & Stalke D., J. Appl. Cryst. 48 (2015) 3-10
T_{min}, T_{max}	0.591, 0.754
No. of measured, independent and observed [$I > 2\sigma(I)$] reflections	27547, 7794, 6848
R_{int}	0.090
$(\sin \theta/\lambda)_{max}$ (Å^{-1})	0.639
**Refinement**	
$R[F^2 > 2\sigma(F^2)]$, $wR(F^2)$, S	0.033, 0.083, 1.02
No. of reflections	7794
No. of parameters	700
No. of restraints	969
H-atom treatment	H atoms treated by a mixture of independent and constrained refinement
$\Delta\rho_{max}$, $\Delta\rho_{min}$ (e Å^{-3})	0.28, -0.26
Absolute structure	Flack x determined using 2604 quotients [(I+)-(I-)]/[(I+)+(I-)] (Parsons, Flack and Wagner, Acta Cryst. B69 (2013) 249-259).
Absolute structure parameter	0.001 (13)

Computer programs: Apex6 v2024.9-1 (Bruker, 2025), *SAINT* V8.41 (Bruker, 2025), *SHELXT* (Sheldrick, 2015b), *SHELXL2019*/2 (Sheldrick, 2015a, 2019), SHELXLE Rev1737 (Hübschle *et al.*, 2011).

Refinement Details:

Triflates were refined as disordered. One two-fold, the other three-fold. The disordered moieties were restrained to have similar geometries. U_{ij} components of ADPs for disordered atoms closer to each other than 2.0 Å were restrained to be similar. Subject to these conditions the occupancy ratio refined to 0.902(3) to 0.098(3) and to 0.446(3) to 0.472(3) to 0.083(3), respectively.

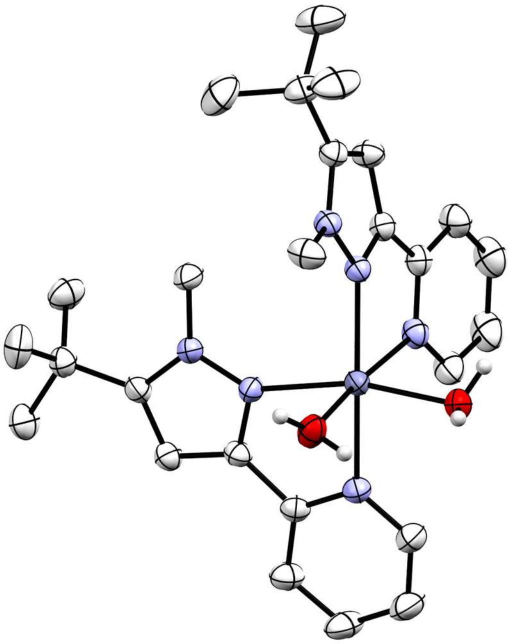

Figure S111. The cationic portion of the molecular structure of $[(^{Me}NN^{tBu})_2Zn(H_2O)_2][OTf]_2$ displayed with 50% probability ellipsoids (outer-sphere trifluoromethanesulfonate anions omitted). All hydrogen atoms not attached to heteroatoms are omitted for clarity.

Compound: $(^{H}NN^{tBu})_2Zn(O_2CAr)_2$ (**6-OMe**; Ar = *p*-C_6H_4OMe)
Local Name: JK-1-27
CCDC Number: 2395150

Table S13. Crystallographic details for $(^{H}NN^{tBu})_2Zn(O_2CAr)_2$ (**6-OMe**; Ar = *p*-C_6H_4OMe)

**Crystal data**	
Chemical formula	$C_{40}H_{44}N_6O_6Zn$
M_r	770.18
Crystal system, space group	Monoclinic, $P2_1/n$
Temperature (K)	150
a, *b*, *c* (Å)	9.0654 (5), 16.2133 (11), 26.5148 (17)
β (°)	96.633 (2)
V ($Å^3$)	3871.1 (4)
Z	4
Radiation type	Mo $K\alpha$
μ (mm^{-1})	0.69
Crystal size (mm)	0.42 × 0.24 × 0.07
**Data collection**	
Diffractometer	Bruker AXS D8 Quest diffractometer with PhotonII charge-integrating pixel array detector (CPAD)
Absorption correction	Multi-scan *SADABS* 2016/2: Krause, L., Herbst-Irmer, R., Sheldrick G.M. & Stalke D., J. Appl. Cryst. 48 (2015) 3-10
T_{min}, T_{max}	0.638, 0.747
No. of measured, independent and observed [$I > 2\sigma(I)$] reflections	134703, 14778, 11613
R_{int}	0.047
$(\sin \theta/\lambda)_{max}$ ($Å^{-1}$)	0.770
**Refinement**	
$R[F^2 > 2\sigma(F^2)]$, $wR(F^2)$, S	0.035, 0.097, 1.02
No. of reflections	14778
No. of parameters	557
No. of restraints	301
H-atom treatment	H atoms treated by a mixture of independent and constrained refinement
$\Delta\rho_{max}$, $\Delta\rho_{min}$ (e $Å^{-3}$)	0.42, -0.49

Computer programs: Apex4 v2022.10-RC10 (Bruker, 2022), *SAINT* V8.40B (Bruker, 2020), *SHELXT* 2014/5 (Sheldrick, 2015), *SHELXL2018*/3 (Sheldrick, 2015, 2018), SHELXLE Rev1275 (Hübschle *et al.*, 2011).

Refinement Details:

A C_6H_4OMe ring was modeled as disordered over two positions. The two disordered moieties were restrained to have similar geometries to the well-defined ligand of the same type and their aromatic rings were restrained to be planar. U_{ij} components of ADPs for disordered atoms closer to each other than 2.0 Å were restrained to be similar. The U_{ij} components of the ADPs and the XYZ coordinates of C2A and C2B were constrained to be identical. Subject to these conditions the occupancy ratio refined to 0.313 (12) to 0.687 (12). One reflection blocked by the beamstop was omitted. Hydrogens on the nitrogen were refined due to hydrogen bonding.

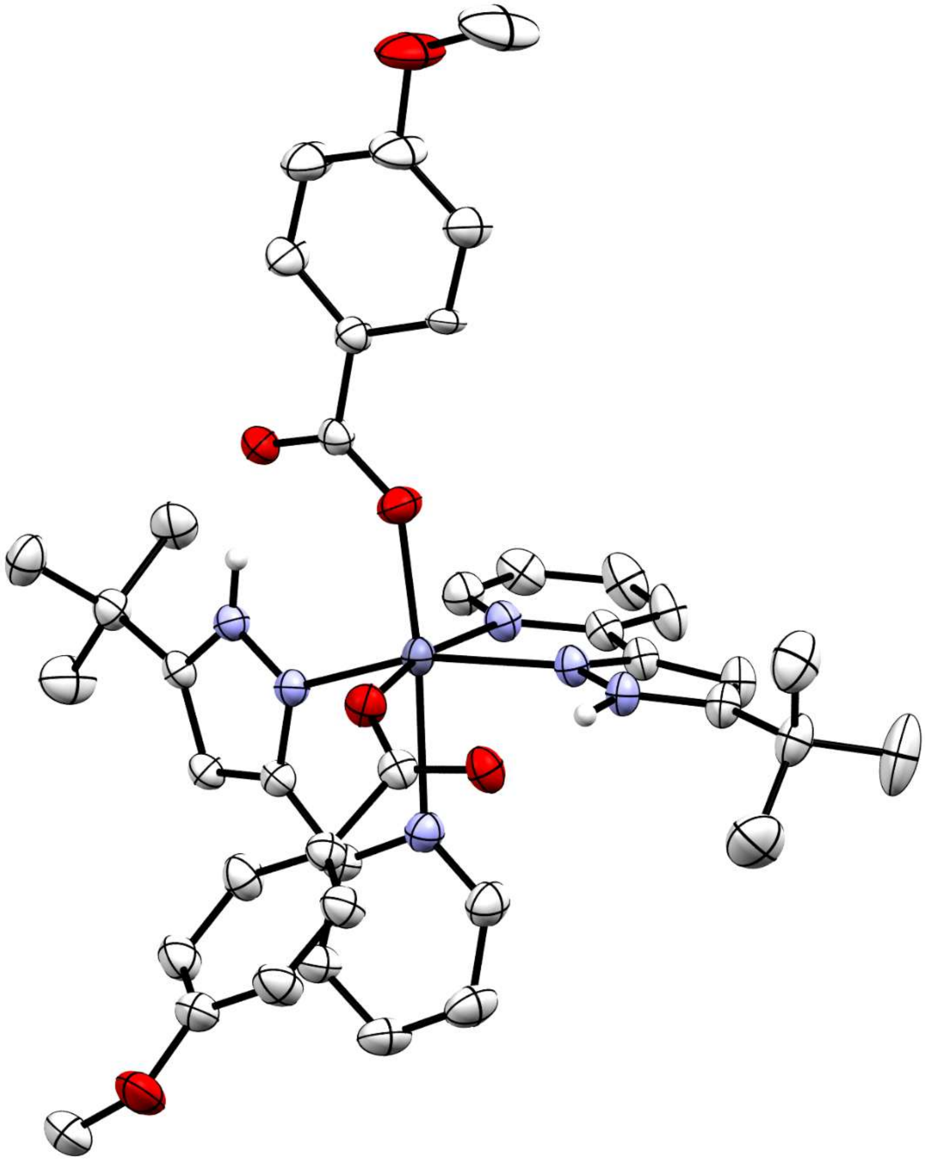

Figure S112. Molecular structure of ($^{H}NN^{tBu}$)$_2$Zn(O_2CAr)$_2$ (**6-OMe**; Ar = *p*-C_6H_4OMe) displayed with 50% probability ellipsoids. Hydrogen atoms not connected to nitrogen are omitted for clarity. Disordered fragments are omitted for clarity.

Compound: $(^{H}NN^{tBu})_2Zn(O_2CPh)_2$ (**6-H**)
Local Name: JK-1-19
CCDC Number: 2395148

Table S14. Crystallographic details for $(^{H}NN^{tBu})_2Zn(O_2CPh)_2$ (**6-H**)

**Crystal data**	
Chemical formula	$C_{38}H_{40}N_6O_4Zn$
M_r	710.13
Crystal system, space group	Monoclinic, $P2_1/c$
Temperature (K)	150
a, *b*, *c* (Å)	10.457 (4), 17.766 (5), 39.82 (2)
β (°)	96.614 (18)
V (Å^3)	7348 (5)
Z	8
Radiation type	Mo $K\alpha$
μ (mm^{-1})	0.72
Crystal size (mm)	0.55 × 0.14 × 0.12
**Data collection**	
Diffractometer	Bruker AXS D8 Quest diffractometer with PhotonII charge-integrating pixel array detector (CPAD)
Absorption correction	Multi-scan *SADABS* 2016/2: Krause, L., Herbst-Irmer, R., Sheldrick G.M. & Stalke D., J. Appl. Cryst. 48 (2015) 3-10
T_{min}, T_{max}	0.685, 0.746
No. of measured, independent and observed [$I > 2\sigma(I)$] reflections	79072, 18032, 12275
R_{int}	0.068
$(\sin \theta/\lambda)_{max}$ (Å^{-1})	0.667
**Refinement**	
$R[F^2 > 2\sigma(F^2)]$, $wR(F^2)$, S	0.042, 0.116, 1.04
No. of reflections	18032
No. of parameters	1015
No. of restraints	444
H-atom treatment	H-atom parameters constrained
$\Delta\rho_{max}$, $\Delta\rho_{min}$ (e Å^{-3})	1.86, -1.04

Computer programs: Apex4 v2021.10-RC6 (Bruker, 2021), *SAINT* V8.40B (Bruker, 2020), *SHELXT* (Sheldrick, 2015), *SHELXL2018*/3 (Sheldrick, 2015, 2018), SHELXLE Rev1275 (Hübschle *et al.*, 2011).

Refinement Details:

Three of four *tert*-butyl groups were refined as disordered by rotation. The disordered moieties were restrained to have a similar geometry as the one not disordered *tert*-butyl group. U_{ij} components of ADPs for disordered atoms closer to each other than 2.0 Å were restrained to be similar. Subject to these conditions the occupancy ratio refined to 0.852(5) to 0.148(5) (residue 1), 0.885(3) to 0.115(3) (residue 2) and 0.591(9) to 0.409(9) (residue 4).

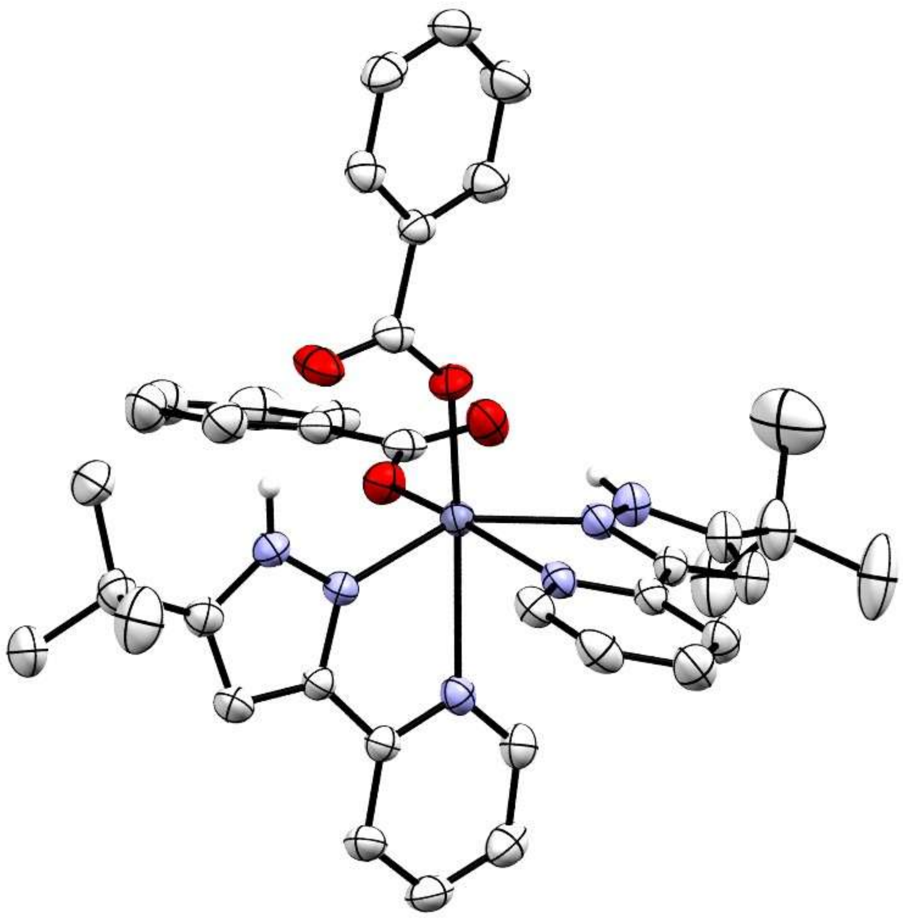

Figure S113. Molecular structure of $(^{H}NN^{tBu})_2Zn(O_2CPh)_2$ (**6-H**) of one of two independent molecules within the unit cell displayed with 50% probability ellipsoids. Hydrogen atoms not connected to nitrogen are omitted for clarity.

Compound: $(^{H}NN^{tBu})_2Zn(O_2CAr)_2$ (**6-Br**; Ar = *p*-C_6H_4Br)
Local Name: JK-1-36
CCDC Number: 2395144

Table S15. Crystallographic details for $(^{H}NN^{tBu})_2Zn(O_2CAr)_2$ (**6-Br**; Ar = *p*-C_6H_4Br)

**Crystal data**	
Chemical formula	$C_{38}H_{38}Br_2N_6O_4Zn{\cdot}2(CH_2Cl_2)$
M_r	1037.79
Crystal system, space group	Monoclinic, *C*2/*c*
Temperature (K)	150
a, *b*, *c* (Å)	25.5001 (17), 9.7209 (5), 19.0141 (13)
β (°)	107.335 (3)
V (Å^3)	4499.2 (5)
Z	4
Radiation type	Mo *K*α
μ (mm^{-1})	2.61
Crystal size (mm)	0.41 × 0.21 × 0.09
**Data collection**	
Diffractometer	Bruker AXS D8 Quest diffractometer with PhotonII charge-integrating pixel array detector (CPAD)
Absorption correction	Multi-scan *SADABS* 2016/2: Krause, L., Herbst-Irmer, R., Sheldrick G.M. & Stalke D., J. Appl. Cryst. 48 (2015) 3-10
T_{min}, T_{max}	0.513, 0.747
No. of measured, independent and observed [$I > 2\sigma(I)$] reflections	30082, 8581, 6498
R_{int}	0.031
$(\sin \theta/\lambda)_{max}$ (Å^{-1})	0.770
**Refinement**	
$R[F^2 > 2\sigma(F^2)]$, $wR(F^2)$, S	0.031, 0.083, 1.07
No. of reflections	8581
No. of parameters	264
H-atom treatment	H atoms treated by a mixture of independent and constrained refinement
$\Delta\rho_{max}$, $\Delta\rho_{min}$ (e Å^{-3})	0.80, -1.00

Computer programs: Apex4 v2022.10-RC10 (Bruker, 2022), *SAINT* V8.40B (Bruker, 2020), *SHELXT* (Sheldrick, 2015), *SHELXL2018*/3 (Sheldrick, 2015, 2018), SHELXLE Rev1275 (Hübschle *et al.*, 2011).

Refinement Details:

Positions of N-bound H-atoms were freely refined.

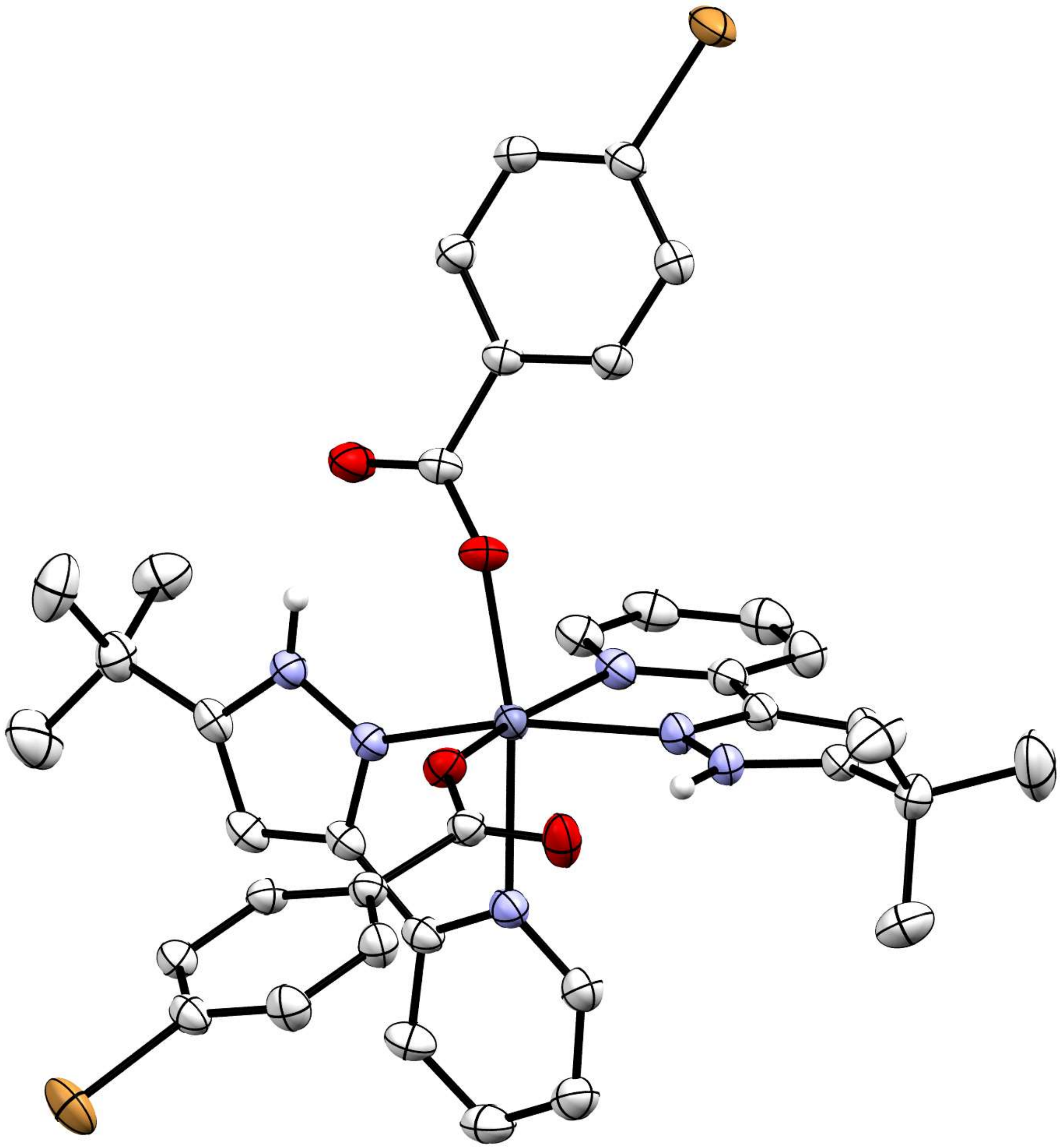

Figure S114. Molecular structure of ($^{H}NN^{tBu}$)$_2$Zn(O_2CAr)$_2$ (**6-Br**; Ar = *p*-C_6H_4Br) displayed with 50% probability ellipsoids. Hydrogen atoms not connected to nitrogen are omitted for clarity. Co-crystallized solvent (CH_2Cl_2) is omitted for clarity.

Compound: (L)$Zn_4O(OAc)_6$
Local Name: JK-1-67
CCDC Number: 2443233

Table S16. Crystallographic details for (L)$Zn_4O(OAc)_6$

**Crystal data**	
Chemical formula	$C_{24}H_{33}N_3O_{13}Zn_4$
M_r	833.01
Crystal system, space group	Triclinic, $P\overline{1}$
Temperature (K)	150
a, *b*, *c* (Å)	10.4868 (7), 10.8215 (7), 17.4833 (12)
α, β, γ (°)	72.488 (2), 88.589 (3), 61.799 (2)
V (Å^3)	1651.10 (19)
Z	2
Radiation type	Mo *K*α
μ (mm^{-1})	2.93
Crystal size (mm)	0.31 × 0.23 × 0.11
**Data collection**	
Diffractometer	Bruker AXS D8 Quest
Absorption correction	Multi-scan *SADABS* 2016/2: Krause, L., Herbst-Irmer, R., Sheldrick G.M. & Stalke D., J. Appl. Cryst. 48 (2015) 3-10
T_{min}, T_{max}	0.501, 0.747
No. of measured, independent and observed [$I > 2\sigma(I)$] reflections	63928, 12538, 9288
R_{int}	0.071
$(\sin \theta/\lambda)_{max}$ (Å^{-1})	0.770
**Refinement**	
$R[F^2 > 2\sigma(F^2)]$, $wR(F^2)$, S	0.040, 0.104, 1.04
No. of reflections	12538
No. of parameters	406
H-atom treatment	H-atom parameters constrained
$\Delta\rho_{max}$, $\Delta\rho_{min}$ (e Å^{-3})	0.72, -0.78

Computer programs: Apex5 v2023.9-2 (Bruker, 2023), *SAINT* V8.40B (Bruker, 2020), *SHELXT* (Sheldrick, 2015b), *SHELXL2019*/2 (Sheldrick, 2015a, 2019), SHELXLE Rev1703 (Hübschle *et al.*, 2011).

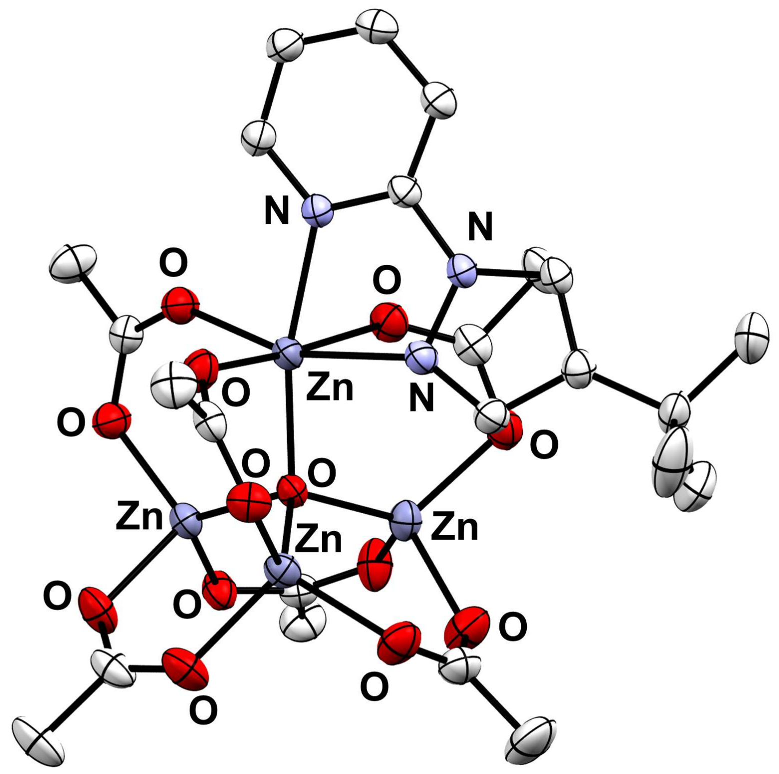

Figure S115. Molecular structure of (L)$Zn_4O(OAc)_6$ displayed with 50% probability ellipsoids. All hydrogen atoms are omitted for clarity.

Table S17. Experimentally determined metrical parameters for tetrahedral zinc complexes. All bond distances reported in Å.

	Zn-N_{pz}	Zn-N_{pyr}	Zn-X	Zn-X	N···X	τ_4
($^{H}NN^{tBu}$)$ZnCl_2$ (**1-Cl**)	2.0373(15)	2.0795(16)	2.2345(6)	2.1959(6)	3.146	0.87
($^{H}NN^{tBu}$)$ZnBr_2$ (**1-Br**)	2.033(3)[a]<br>2.028(3)[b]<br>2.041(3)[c]	2.080(3)[a]<br>2.097(3)[b]<br>2.068(3)[c]	2.3335(8)[a]<br>2.3452(8)[b]<br>2.3653(8)[c]	2.3447(9)[a]<br>2.3337(7)[b]<br>2.3301(9)[c]	3.421[a,b]<br>3.369[a,b]<br>3.396[c]	0.86[a]<br>0.87[b]<br>0.88[c]

[a]For molecule containing Zn1_1
[b]For molecule containing Zn1_2
[c]For molecule containing Zn1_3

Table S18. Experimentally determined metrical parameters for octahedral zinc complexes. All bond distances reported in Å.

	Zn-N_{pz}	Zn-N_{pz}	Zn-N_{pyr}	Zn-N_{pyr}	Zn-O	Zn-O	N···O	N···O
($^{H}NN^{tBu}$)$_2$Zn(OAc)$_2$<br>(**3**)	2.0704(12)[a]<br>2.0414(12)[b]	--<br>--	2.3650(14)[a]<br>2.4043(14)[b]	--<br>--	2.0510(12)[a]<br>2.0742(12)[b]	--<br>--	2.614[a]<br>2.622[b]	--<br>--
($^{H}NN^{tBu}$)$_2$Zn(O_2CFc)$_2$<br>(**4**)	2.0550(14)[a]	2.0562(15)[a]	2.3046(15)[a]	2.4274(16)[a]	2.0196(14)[a]	2.0452(13)[a]	2.692[a]	2.699[a]
($^{Me}NN^{tBu}$)Zn(O_2CFc)$_2$<br>(H_2O)<br>(**4’**)	2.2292(19)	--	2.116(2)	--	2.1564(19)<br>2.2297(18)	2.0902(17)		
($^{H}NN^{tBu}$)$_2$Zn(OTf)$_2$<br>(**5**)	2.0405(15)[a]	--	2.1838(16)[a]	--	2.2267(14)[a]	--	2.799[a]	--
($^{H}NN^{tBu}$)$_2$Zn(O_2CAr)$_2$<br>(Ar = *p*-$C_6H_4OCH_3$)<br>(**6-OMe**)	2.0584(10)	2.0593(10)	2.3782(11)	2.3673(11)	2.0371(9)	2.0585(9)	2.684	2.616
($^{H}NN^{tBu}$)$_2$Zn(O_2CAr)$_2$<br>(Ar = C_6H_5)<br>(**6-H**)	2.0581(17)[a]<br>2.0348(17)[c]	2.0633(18)[a]<br>2.0501(18)[c]	2.353(2)[a]<br>2.429(2)[c]	2.5021(18)[a]<br>2.4821(19)[c]	2.0337(15)[a]<br>2.0255(19)[c]	2.0485(17)[a]<br>2.0577(17)[c]	2.599[a]<br>2.577[c]	2.637[a]<br>2.615[c]
($^{H}NN^{tBu}$)$_2$Zn(O_2CAr)$_2$<br>(Ar = *p*-C_6H_4Br)<br>(**6-Br**)	2.0591(11)[a]	--	2.3012(12)[a]	--	2.0680(10)[a]	--	2.693[a]	--

[a]For molecule containing Zn1
[b]For molecule containing Zn2
[c]For molecule containing Zn1_3

Table S19. Experimentally determined metrical parameters for homoleptic zinc complexes. All bond distances reported in Å.

	Zn-N_{pz}	Zn-N_{pyr}	Zn-NCS	N···X
[($^{H}NN^{tBu}$)$_3$Zn][ClO_4]$_2$<br>(**2-ClO$_4$**)	2.135(4)<br>2.164(4)<br>2.147(4)	2.162(5)<br>2.169(4)<br>2.201(4)	--	2.840[a]<br>2.904[b]<br>2.945[b]
[($^{H}NN^{tBu}$)$_3$Zn][Zn(NCS)$_4$]$_2$<br>(**2-SCN**)	2.17(2)<br>2.129(8)<br>2.181(7)	2.152(8)<br>2.175(7)<br>2.144(8)	1.93(2)[c]<br>1.971(10)<br>1.981(11)<br>2.003(11)	3.459 / 3.486[d]<br>2.948 / 3.169[e]

[a]H-bond between pyrazole N-H and cocrystallized H_2O molecule.
[b]H-bond between pyrazole N-H and perchlorate counteranion.
[c]Moiety occupationally disordered with a chloride anion. Zn-Cl = 2.319(7) Å.
[d]H-bond between pyrazole N-H and sulfur of thiocyanate anion. Pyrazole is disordered over two orientations.
[e]H-bond between pyrazole N-H and nitrogen (2.948 Å) of thiocyanate anion or chloride anion (3.169 Å). The anion is occupationally disordered.

Table S20. Experimentally determined metrical parameters for acetate containing zinc complexes. All bond distances reported in Å.

	Zn-N_{pz}	Zn-N_{pyr}	Zn-$O_{acetate}$	Zn-μ_4O	N···O
$(^{H}NN^{tBu})_2Zn(OAc)_2$ (**3**)	2.0704(12)[a]<br>2.0414(12)[b]	2.3650(14)[a]<br>2.4043(14)[b]	2.0510(12)[a]<br>2.0742(12)[b]	n/a	2.614[a]<br>2.622[b]
$(^{Me}NN^{tBu})Zn(OAc)_2$ (**3'**)	2.1401(9)	2.1009(11)	2.0718(11)<br>2.0921(10)<br>2.2247(10)<br>2.2785(13)	n/a	n/a
$(L_3)Zn_4O(OAc)_6$	2.2443(19)[c]	2.1281(18)[c]	2.0671(17)[c]<br>2.0836(17)[c]<br>2.2634(17)[c]<br>1.9435(18)[d]<br>1.9706(19)[d]<br>1.9771(17)[d]<br>1.9335(17)[e]<br>1.9639(18)[e]<br>1.9860(16)[e]<br>1.9376(17)[f]<br>1.9672(19)[f]<br>1.9728(18)[f]	2.0096(15)[c]<br>1.9304(15)[d]<br>1.9176(15)[e]<br>1.9348(15)[f]	n/a

[a]For molecule containing Zn1
[b]For molecule containing Zn2
[c]For octahedral Zn1
[d]For tetrahedral Zn2
[e]For tetrahedral Zn3
[f]For tetrahedral Zn4

Table S21. Experimentally determined metrical parameters for zinc complexes containing $^{Me}NN^{tBu}$ ligand. All bond distances reported in Å.

	Zn-N_{pz}	Zn-N_{pyr}	Zn-$O_{carboxylate}$ or Zn-$O_{triflate}$	Zn-$O_{carboxylate}$ or Zn-$O_{triflate}$	Zn-O_{H2O}
$(^{Me}NN^{tBu})Zn(OAc)_2$ (**3'**)	2.1401(9)	2.1009(11)	2.2247(10)<br>2.0921(10)	2.0718(11)<br>2.2785(13)	--
$(^{Me}NN^{tBu})Zn(O_2CFc)_2(H_2O)$ (**4'**)	2.2292(19)	2.116(2)	2.1564(19)<br>2.2297(18)	2.0902(17)	2.0344(18)
$(^{Me}NN^{tBu})_2Zn(OTf)_2$ (**5'**)	2.1366(12)<br>2.1254(12)	2.1366(12)<br>2.1844(12)	2.088(13)<br>// 2.086(19)	2.1267(10)	
$[(^{Me}NN^{tBu})Zn(H_2O)_4][OTf]_2$[a]	2.129(2)	2.120(2)	--	--	2.132(2)<br>2.122(2)<br>2.082(2)<br>2.064(2)
$[(^{Me}NN^{tBu})Zn(H_2O)_3][OTf]_2$[a]	2.301(7)	2.176(7)	--	--	2.007(7)<br>1.99(2)<br>1.97(2)
$[(^{Me}NN^{tBu})_2Zn(H_2O)_2][OTf]_2$	2.185(3)<br>2.163(3)	2.166(3)<br>2.154(3)			2.123(2)<br>2.099(2)

[a]These fragments are disordered with one another.

Table S22. Experimentally determined hydrogen bonding distances (crystallography) and N-H resonances (^{1}H NMR) for ^{H}NN^{tBu} ligand containing complexes. All bond distances reported in Å. The distances reported are between heavy atoms (i.e. N···X). ^{1}H NMR recorded in $CDCl_3$ under inert condition and internally referenced versus tetramethylsilane.

	**N···X**	**N···X**	**N-H Resonance**
(^{H}NN^{tBu})$ZnCl_2$<br>(**1-Cl**)	3.146	3.146	
(^{H}NN^{tBu})$ZnBr_2$<br>(**1-Br**)	3.421[a,b]<br>3.369[a,b]<br>3.396[c]	3.421[a,b]<br>3.369[a,b]<br>3.396[c]	
[(^{H}NN^{tBu})$_3$Zn][ClO_4]$_2$<br>(**2-ClO$_4$**)	2.840[f]<br>2.904[g]<br>2.945[g]	2.840[f]<br>2.904[g]<br>2.945[g]	
[(^{H}NN^{tBu})$_3$Zn][Zn(NCS)$_4$]$_2$<br>(**2-SCN**)	3.459 / 3.486[h]<br>2.948 / 3.169[i]	3.459 / 3.486[h]<br>2.948 / 3.169[i]	
(^{H}NN^{tBu})$_2$Zn(OAc)$_2$<br>(**3**)	2.614[d]<br>2.622[e]	--<br>--	15.68 ppm
(^{H}NN^{tBu})$_2$Zn(O_2CFc)$_2$<br>(**4**)	2.692	2.699	12.70 ppm
(^{H}NN^{tBu})$_2$Zn(OTf)$_2$<br>(**5**)	2.799	--	13.18 ppm
(^{H}NN^{tBu})$_2$Zn(O_2CAr)$_2$ (Ar = *p*-$C_6H_4OCH_3$)<br>(**6-OMe**)	2.684	2.616	15.30 ppm
(^{H}NN^{tBu})$_2$Zn(O_2CAr)$_2$ (Ar = C_6H_5)<br>(**6-H**)	2.599[d]<br>2.577[c]	2.637[d]<br>2.615[c]	16.21 ppm
(^{H}NN^{tBu})$_2$Zn(O_2CAr)$_2$ (Ar = *p*-C_6H_4Br)<br>(**6-Br**)	2.693	--	16.36 ppm

[a]For molecule containing Zn1_1
[b]For molecule containing Zn1_2
[c]For molecule containing Zn1_3
[d]For molecule containing Zn1
[e]For molecule containing Zn2
[f]H-bond between pyrazole N-H and cocrystallized H_2O molecule.
[g]H-bond between pyrazole N-H and perchlorate counteranion.
[h]H-bond between pyrazole N-H and sulfur of thiocyanate anion. Pyrazole is disordered over two orientations.
[i]H-bond between pyrazole N-H and nitrogen (2.948 Å) of thiocyanate anion or chloride anion (3.169 Å). The anion is occupationally disordered.

References

(1) Dunaway, L. A.; Davis, A. G.; Carter, V. J.; Korir, A. K.; Zeller, M.; Kiernicki, J. J. Defining the Speciation, Coordination Chemistry, and Lewis Acid Catalysis of Electronically Diverse Zinc Benzoates. *Organometallics* **2025**, *44*, 46-53.

(2) Liu, Y.; Mu, S.; Liu, X.; Ling, Q.; Hang, C.; Ruiz, J.; Astruc, D.; Gu, H. Ferrocenyl Janus mixed-dendron stars and their stabilization of Au and Ag nanoparticles. *Tetrahedron* **2018**, *74*, 4777-4789.

(3) Pearce, B. H.; Ogutu, H. F.; Luckay, R. C. Synthesis of Pyrazole-Based Pyridine Ligands and Their Use as Extractants for Nickel(II) and Copper(II): Crystal Structure of a Copper(II)–Ligand Complex. *Eur. J. Inorg. Chem.* **2017**, *2017*, 1189-1201.

(4) *Bruker Advanced X-ray Solution, Apex3, SAINT, SADABS, Bruker AXS Inc.: Madison (WI), USA, 2018.*; (accessed.

(5) *SHELXTL suite of programs, Version 6.14, 2000-2003, Bruker Advanced X-ray Solutions, Bruker AXS Inc., Madison, Wisconsin: USA*.

(6) Sheldrick, G. A short history of SHELX. *Acta Crystallogr. Section A* **2008**, *64*, 112-122.

(7) Sheldrick, G. Crystal structure refinement with SHELXL. *Acta Crystallogr. Section C* **2015**, *71*, 3-8.

(8) Hübschle, C. B.; Sheldrick, G. M.; Dittrich, B. ShelXle: a Qt graphical user interface for SHELXL. *Journal of Applied Crystallography* **2011**, *44*, 1281-1284.

(9) Su, D.; Liu, Y.; Li, S.; Ding, S.; Jin, Y.; Wang, Z.; Hu, X.; Zhang, L. Selective Extraction of Americium(III) over Europium(III) Ions with Pyridylpyrazole Ligands: Structure–Property Relationships. *Eur. J. Inorg. Chem.* **2017**, *2017*, 651-658.

(10) Waniek, S. D.; Klett, J.; Förster, C.; Heinze, K. Polysubstituted ferrocenes as tunable redox mediators. *Beilstein Journal of Organic Chemistry* **2018**, *14*, 1004-1015.

(11) Kiernicki, John J.; Norwine, Emily E.; Lovasz, Myles A.; Zeller, Matthias; Szymczak, Nathaniel K. Mobility of Lewis Acids within the secondary coordination sphere: toward a model for cooperative substrate binding. *Chem. Commun.* **2020**, *56*, 13105-13108.

(12) Tian, H.; Xue, W; Wu, J.; Yang, Z.; Lu, H; Tang, C. A General and Practical Bifunctional Cobalt Catalytic System for N-Heterocycle Assembly via Acceptorless Dehydrogenation. *Org. Chem. Front.* **2022**, *9*, 4554-4560.

(13) Kim, H. T.; Kang, E.; Kim, M.; Joo, J. M. Synthesis of Bidentate Nitrogen Ligands by Rh-Catalyzed C–H Annulation and Their Application to Pd-Catalyzed Aerobic C–H Alkenylation. *Org. Lett.* **2021**, *23*, 3657-3662.
